# Making the most of your host: the *Metrosideros*-feeding psyllids (Hemiptera, Psylloidea) of the Hawaiian Islands

**DOI:** 10.3897/zookeys.649.10213

**Published:** 2017-01-31

**Authors:** Diana M. Percy

**Affiliations:** 1 Department of Life Sciences, Natural History Museum, Cromwell Road, London, UK, and University of British Columbia, Faculty of Science, University Boulevard, Vancouver, BC, Canada

**Keywords:** Gall biology, jumping plant lice, mitochondrial DNA barcode, morphology, parallel evolution, Pariaconus, species radiation, taxonomic revision, Triozidae

## Abstract

The Hawaiian psyllids (Psylloidea, Triozidae) feeding on Metrosideros (Myrtaceae) constitute a remarkable radiation of more than 35 species. This monophyletic group has diversified on a single, highly polymorphic host plant species, Metrosideros polymorpha. Eleven Metrosideros-feeding species included in the Insects of Hawaii by Zimmerman are redescribed, and an additional 25 new species are described. Contrary to previous classifications that placed the Metrosideros-feeders in two genera, Trioza Foerster, 1848 and Kuwayama Crawford, 1911, all 36 named species are placed in Pariaconus Enderlein, 1926; and the relationship of this genus to other Pacific taxa within the family Triozidae, and other Austro-Pacific taxa feeding on host plants in Myrtaceae is clarified. The processes of diversification in Pariaconus include shifts in galling habit, geographic isolation within and between islands, and preferences for different morphotypes of the host plant. Four species groups are recognized: the bicoloratus and minutus groups are free-living or form pit galls, and together with the kamua group (composing all of the Kauai species) form a basal assemblage; the more derived closed gall species in the ohialoha group are found on all major islands except Kauai. The diversification of Pariaconus has likely occurred over several million years. Within island diversification is exemplified in the kamua group, and within species variation in the ohialoha group, but species discovery rates suggest this radiation remains undersampled. Mitochondrial DNA barcodes are provided for 28 of the 36 species. Genetic divergence, intraspecific genetic structure, and parallel evolution of different galling biologies and morphological traits are discussed within a phylogenetic framework. Outgroup analysis for the genus Pariaconus and ancestral character state reconstruction suggest pit-galling may be the ancestral state, and the closest outgroups are Palaearctic-Australasian taxa rather than other Pacific Metrosideros-feeders.

## Introduction

The Hawaiian Islands are renowned for exemplary species radiations (e.g. Magnacca and Danforth 2006, Rubinoff 2008, Givnish et al. 2009, Lerner et al. 2011, Bennett and O’Grady 2012, Goodman et al. 2014, Magnacca and Price 2015), as well as extraordinary and varied processes of species diversification and evolution (Rivera et al. 2002, Mullen et al. 2007, Rubinoff and Schmitz 2010, Wessel et al. 2013, Gillespie 2016). The primary characteristics of the Hawaiian archipelago that are considered important in speciation processes are: a) multiple islands, b) each island with a geographically concentrated heterogeneity of habitats, c) islands occurring in various degrees of isolation and age in relation to one another, and d) the extreme remoteness of the archipelago. Archipelagos with heterogeneity of habitats and varying geological ages are also found in other island systems, but the extreme isolation of the Hawaiian Islands is unique and may ultimately influence patterns of diversification in ways not found elsewhere (Gillespie et al. 2012, Shaw and Gillespie 2016).

The Hawaiian Islands are home to several endemic psyllid lineages that represent multiple independent colonizations, yet an enduring puzzle is why all of these lineages are from a single psyllid family. The native psyllid fauna is composed entirely of species from the family Triozidae; a single Liviidae specimen (collected in 1925) of an undetermined Paurocephala Crawford, 1913 species (Crawford 1927) remains unconfirmed (Zimmerman 1948). Adjacent continental and Pacific island regions are home to all eight psyllid families (notably Aphalaridae, Carsidaridae, and Psyllidae; Hodkinson 1983, 1986, 1988, Yang and Raman 2007), including the Marquesas and Austral Islands to the south of the Hawaiian Islands. The question therefore remains: why have none of these other psyllid families colonized the Hawaiian archipelago? Perhaps triozid psyllids are somehow more successful colonizers of remote islands. Other Pacific archipelagos are located within 1000 km distance of habitable landmasses (via many small islands), whereas the Hawaiian Islands are more than 3000 km from another landmass, which makes dispersal ability a critical factor (Hembry et al. 2013). However, it is difficult to reason how dispersal ability alone could discriminate between psyllid families. The colonization of remote landmasses containing few familiar host plants could be facilitated by an ability to survive on suboptimal or unfamiliar hosts (Roderick and Percy 2008, Percy 2011), and although the majority of psyllids are mono- or oligophagous (feeding on one or a few plant species within the same genus), the family Triozidae includes the largest number of psyllid species exhibiting atypically broad host preference (Ouvrard et al. 2015). An ancestral preadaptation to expanded host ranges (Janz et al. 2006, Janz and Nylin 2008) may be an advantage allowing successful colonization that may then cycle back to specialization given ecological opportunity (e.g. vacant niches, proximity of alternate hosts) (Percy et al. 2004). This scenario could explain both the imbalance in colonization potential of the different psyllid families and the observed pattern of within archipelago host specialization after establishment. There are also examples from introduced species of host range expansions post colonization of a new region, e.g. Trioza eugeniae Froggatt, 1901, which is associated with Syzygium (Myrtaceae) in its native Australian range, but in California where it is introduced it occurs on cultivated Metrosideros (Percy et al. 2012).

Many of the Hawaiian triozid species have been ascribed to endemic genera rather than placed within more widespread and established generic groupings; exceptions include taxa placed in Kuwayama Crawford, 1911 and Trioza Foerster, 1848, two highly artificial genera found in old and new world regions (Hodkinson 1983, 1986, 1988). The practice of erecting endemic Hawaiian genera does, however, often reflect unusual morphology as well as ambiguous affinities to genera elsewhere. The absence of such conspicuous morphological characters distinguishing Metrosideros-feeding species in the Hawaiian Islands is notable, and this is partly responsible for the placement of these taxa in Kuwayama and Trioza. Trioza, in particular, is a large, poorly defined, polyphyletic genus into which many disparate species have been placed (Burckhardt and Ouvrard 2012, Ouvrard et al. 2015). Kuwayama is also considered problematic and currently includes unrelated taxa from the American continent and Hawaiian Islands. Although both Enderlein (1926) and Crawford (1918) recognized the artifice of placing Hawaiian taxa in Kuwayama, it was Enderlein (1926) who removed three of the Metrosideros-feeding species to a separate, endemic genus, Pariaconus Enderlein, 1926; not based on unique differences, but rather on an absence of distinct affinities with other taxa in Kuwayama (Enderlein 1926). Despite the absence of distinct morphological synapomorphies, endemic generic status for the Metrosideros-feeding species is clearly supported by this study. Pariaconus is not close to the type species of Trioza, Trioza urticae (Linné, 1758), nor to the type species of Kuwayama, Kuwayama medicaginis (Crawford, 1910). However, closer taxonomic affinities outside the Hawaiian Islands remain to be clarified with yet further outgroup sampling.

All Hawaiian psyllid lineages appear to exhibit high host specificity that has been conserved during in situ diversification (e.g. Pariaconus on Metrosideros (Myrtaceae), Swezeyana Caldwell, 1940 on Planchonella (Sapotaceae), Hevaheva Kirkaldy, 1902 on Melicope (Rutaceae), Megatrioza Crawford, 1915 on Pritchardia (Arecaceae)) (Zimmerman 1948, Nishida et al. 1980, Uchida and Beardsley 1988). Pariaconus is an endemic Hawaiian genus with more than 35 species found on a single host plant (Metrosideros polymorpha) (Table 1) and species discovery rates suggest this number will continue to increase. This pattern of within host diversification is atypical for psyllids, as well as for other phytophagous insects (Joy and Crespi 2007). The rarity of speciose radiations on a single host plant is thought to be due, in part, to processes such as competitive exclusion between closely related species (Percy 2003b, 2011, Després and Cherif 2004, Gold et al. 2009). A much more common process associated with speciation in phytophagous insects, including psyllids, is switching to different host plants (Tilmon 2008, and chapters therein, Ouvrard et al. 2015), and a high degree of host specialization in psyllids appears to constrain most switching to closely related plant species with much rarer saltationary leaps to unfamiliar hosts (e.g. Burckhardt and Basset 2000, Percy et al. 2004, Ouvrard et al. 2015). The question is therefore whether diversification in Pariaconus evolved via atypical evolutionary processes, or whether typical evolutionary pathways have been co-opted in atypical ways.

**Table 1.** Pariaconus species. List of all 36 described species with species group affiliation and recognized intraspecific forms, and biological habit if known. Islands: K – Kauai, O – Oahu, L – Lanai, Mo – Molokai, Ma – Maui, H – Hawaii.

species group species	authority	forms	island	habit
**bicoloratus** nigricapitus	(Crawford, 1918)		L, Mo, H	free-living
hina	sp. n.	ovostriatus, orientalis, occidentalis	Ma	?
wyvernus	sp. n.	wyvernus, chimera, gorgonus	H	?
nigrilineatus	sp. n.		H	?
kapo	sp. n.		H	?
proboscideus	sp. n.		H	free-living
poliahu	sp. n.		H	?
lona	sp. n.		Mo	?
liliha	sp. n.		O	?
gracilis	(Crawford, 1918)	gracilis, conconus	O, Mo, Ma	free-living
dorsostriatus	sp. n.	kohalensis, communis	H	pit galls on leaves
namaka	sp. n.		O	pit galls on leaves
**minutus** minutus	(Crawford, 1918)	minutus, kilaueaiensis	H	pit galls on leaves
gibbosus	sp. n.		Ma	?
**kamua** iolani	Kirkaldy, 1902	iolani, scapulus	K	? enclosed galls
hiiaka	sp. n.		K	mainly enclosed galls on leaves: domed or donut type; occasionally stem galls
melanoneurus	sp. n.		K	?
grandis	sp. n.		K	?
caulicalix	sp. n.	brunneis, rubrus	K	thin-walled cup galls on stems
crassiorcalix	sp. n.		K	thick-walled cup galls on stems
lehua	Crawford, 1925		K	?
elegans	sp. n.		K	?
gagneae	sp. n.		K	?
haumea	sp. n.		K	?
**ohialoha** oahuensis	sp. n.	oahuensis, tenuis, latus	O	mainly enclosed galls on stems/buds; occasionally galls leaves: cone type
ohiacola	Crawford, 1918	ohiacola, angustipterus, obtusipterus, waianaiensis	O	enclosed galls on leaves: flat type
lanaiensis	Crawford, 1918		L, Mo	enclosed galls on stems
pullatus	Crawford, 1918		L	?
molokaiensis	Crawford, 1927	molokaiensis, laka	Mo	enclosed galls on stems
hualani	sp. n.		Mo	enclosed galls on leaves
mauiensis	sp. n.	mauiensis, kuula	Ma	?
kupua	sp. n.		Ma	enclosed galls on stems
montgomeri	sp. n.	montgomeri, paliuliensis	Ma	enclosed galls on leaves: flat type
hawaiiensis	Crawford, 1918		H	enclosed galls on stems
pele	sp. n.	pele, kohalensis	H	enclosed galls on leaves: flat or donut type
pyramidalis	sp. n.		H	mainly enclosed galls on leaves: cone type; occasionally stem galls

In order to understand how the Pariaconus radiation has evolved and persisted on Metrosideros polymorpha, it is crucial to appreciate the ecological background over which the speciation processes have played out. Except in the very driest regions, Metrosideros polymorpha is the dominant native shrub in the Hawaiian Islands (Mueller-Dombois 1987, 1992, Dawson and Stemmermann 1999). There are currently eight Metrosideros polymorpha varieties recognized, but these inadequately represent the complex morphological variation observed within and between islands (Percy et al. 2008, Harbaugh et al. 2009, Wright and Ranker 2010, Stacy et al. 2014, 2016). In growth habit, the variation includes statuesque trees (~20 m) and low growing shrubs (< 1 m). Habitats may be wet or dry forest, bogs, or almost soil-less lava flows a few years old (Stacy et al. 2014, 2016). Studies of the morphological variation (e.g. leaf pubescence and thickness), including common garden and genetic studies, have concluded that the diversity of phenotypes is controlled by complex genome-environment interactions, as well as population dynamics (Cordell et al. 1998, Gruner et al. 2005, Wright and Ranker 2010, Stacy et al. 2014). Indeed, the presence of different morphotypes growing side by side in the same environment but exhibiting little or no genetic variation for neutral markers is one of the most striking features of this polymorphic plant species (Percy et al. 2008, Wright and Ranker 2010, Stacy et al. 2016).

The patterns of diversity observed in Pariaconus would not be markedly different from other radiations (e.g. those found in the Atlantic island archipelago of the Canary Islands; Percy 2003a, 2003b) if the variation found in Metrosideros polymorpha was represented by 20 or more distinct species with speciation occurring primarily via a process of switching between hosts. The Canary Islands, although less remote, share many attributes with the Hawaiian Islands, e.g. five major islands formed in an age progressive series over a volcanic hot spot, with similar degrees of habitat heterogeneity and inter-island distances (Neall and Trewick 2008, Bogaard 2013). In the Hawaiian Islands, the high degree of polymorphism in Metrosideros polymorpha may be promoting psyllid speciation processes akin to the effect of multiple host plants. However, understanding the diversification processes is further complicated by shifts in galling biology, and shifts to galling different plant organs.

When considering the origins of Hawaiian Pariaconus, Crawford (1918) thought “the original immigrant to be one inhabiting leaf galls”, and although originally describing different Pariaconus taxa in separate genera, he also stated, “as new species evolved from this, some have retained the gall-making habit … others have taken to living free in the nymphal stages, while still others have gone off to other plants making leaf galls or living free”, in other words he contemplated the scenario that all the Metrosideros-feeders, and even all Hawaiian Triozidae could be a monophyletic group. This illustrates Crawford’s ambiguous approach to the concept of monophyly. He apparently saw no conflict in simultaneously considering evolutionary shifts in situ within the Hawaiian Islands, but at the same time ascribing these island species to different genera based on superficial morphological affiliations with continental groups. Indeed, Crawford noted, when assigning three newly described Hawaiian Metrosideros-feeding species to Kuwayama, that “it seems certain that the species placed in this genus do not represent a common origin at all, but independent or parallel evolution toward the same end” (Crawford 1918). He goes on, “the three species seem almost certainly to have been derived from some Trioza species, probably Trioza ohiacola [Pariaconus ohiacola], or an ancestral type proceeding it”. He is apparently confirming here that he views taxonomic groupings as artificial and more for convenience than necessarily representative of evolutionary origins.

Multiple shifts in galling biology on the same plant species have rarely been documented. One of the few known systems is the gall-inducing Asphondylia Loew, 1850 (Cecidomyiidae) flies found on Larrea tridentata (Joy and Crespi 2007) where diversification has involved numerous shifts between different plant organs (leaves, buds, flowers, and stems) of the same host-plant species, thereby ecologically partitioning the host plant, with additional temporal as well as spatial separation. In a study of oak-gall wasps, Cook et al. (2002) found speciation more likely to involve shifts to galling different plant organs if the host oaks were more closely related (within taxonomic sections) than when host shifts involved oaks in different sections. Another study looking at Eurosta Loew, 1873 (Tephritidae) gall flies on Solidago where sympatric speciation has involved shifting between leaves and stems has suggested gall makers are more likely to speciate sympatrically than non-gall makers (Craig et al. 1994). These findings are consistent with patterns observed in the Metrosideros-psyllid system and thus help to frame hypotheses that may explain diversity in Hawaiian Pariaconus. However, analysis of the processes of speciation in Pariaconus may be additionally complicated by the underlying complexity inherent in the mosaic of plant phenotypes, and the frequent co-occurrence of alternate Metrosideros morphotypes.

The objectives of this study are, a) to describe the diversity of species feeding on Metrosideros in the Hawaiian Islands, b) to identify patterns of diversity within and between islands across the archipelago, c) to test putative outgroup affiliations, and d) to better understand the origins and evolution of Pariaconus.

## Materials, methods and terminology

### Sampling

Over 500 specimens from museum and field collections were examined (Tables 2–3). Field collections in the Hawaiian Islands were made between 2002–2014 as follows: May 2002 (Kauai, Oahu, Hawaii); July 2002 (Maui, Hawaii); August 2003 (Oahu, Molokai, Hawaii); October–November 2005 (Kauai); May–June 2011 (Oahu, Hawaii); July 2013 (Hawaii); March 2014 (Hawaii); July 2014 (Oahu, Maui). Thirty-eight outgroup taxa were sampled from Hawaiian and other island and continental regions. Adults and immatures were collected in the field into 95% ethanol. To associate adults with immature biologies, immatures were sampled whenever possible (e.g. removed from galls, and in some cases adults were reared from galls by placing cut branches in plastic bags). Specimens were preserved in 95% ethanol and stored in -20°C for morphological and DNA analyses.

**Table 2.** Pariaconus type and other material examined (m – male, f – female, i – immature) with GenBank numbers for cytochrome oxidase one (COI) and cytochrome B (cytB).

species group species	material	n	collection information	lat N, long W	GenBank COI/cytB
**bicoloratus**					
nigricapitus	holotype	f	Niulii, Hawaii, USA, ex Ohia Lehua, 22 May 1917, O. Swezey leg. (BPBM)	−	−
	other	6i	same data as holotype		−
	other	1f	Olaa Forest, Hawaii, USA, ex Metrosideros polymorpha var. glaberrima, 24 August 2003, “Hi43-03” D. Percy leg. (BMNH)	19.4603, -155.2467	−
	other	1m	Puu O Umi, Kohala, Hawaii, USA, ex Metrosideros polymorpha var. polymorpha, 2 June 2011, “Hi07-11” D. Percy leg. (BMNH)	20.0701, -155.7240	−
hina	holotype	m	Olinda Flume Road, Makawao, East Maui, USA, ex Metrosideros polymorpha, 3 July 2014, “Hi53-14” D. Percy leg. (BMNH)	20.8106, -156.2398	−
	paratypes	3f	same data as previous except: 25 July 2002, “429-02” D. Percy leg. (BMNH)	20.8047, -156.2608	KY293816/KY294293
	other	1f	Kamakou Preserve, Molokai, USA, on Planchonella sandwicensis, 17 August 2003, “Hi20-03” D. Percy leg. (BMNH)	21.1236, -156.9108	
	other	1m	Puu Kukui, boardwalk trail, West Maui, USA, ex Metrosideros polymorpha, 2 July 2014, “Hi48-14” D. Percy leg. (BMNH)	20.9343, -156.6137	KY293806/KY294281
	other	1f	same data as previous except: “Hi49-14” D. Percy leg. (BMNH)	20.9339, -156.6132	KY293807/KY294282
	other	1m, 1f	Olinda Flume Road, Makawao, East Maui, USA, ex Metrosideros polymorpha, 1 July 2014, “Hi41-14” D. Percy leg. (BMNH)	20.8115, -156.2381	KY293804-05/KY294279-80
	other	3m, 2f	same data as previous except: 3 July 14, “Hi52-14” D. Percy leg. (BMNH)	20.8099, -156.2501	KY293808-12/KY294283-88
	other	1m, 3f	same data as previous except: “Hi53-14” D. Percy leg. (BMNH)	20.8106, -156.2398	KY293813-15/KY294289-92
wyvernus	holotype	m	Upper Hamakua Ditch, Kohala, Hawaii, USA, ex Metrosideros polymorpha var. glaberrima, 5 March 2014, “Hi09-14” D. Percy leg. (BMNH)	20.0714, -155.6711	KY294127/KY294610
	paratype	1f	same data as holotype		KY294126/KY294609
	other	1m	Upper Hamakua Ditch, Kohala, Hawaii, USA, ex Metrosideros polymorpha var. glaberrima, 5 March 2014, “Hi08-14” D. Percy leg. (BMNH)	20.0693, -155.6697	KY294125/KY294608
wyvernus	other	1f	same data as previous except: ex Metrosideros polymorpha var. polymorpha, “Hi10-14” D. Percy leg. (BMNH)	20.0853, -155.6778	KY294128/KY294611
	other	1m, 1f	same data as previous except: ex Metrosideros polymorpha, “Hi11-14” D. Percy leg. (BMNH)	20.0852, -155.6791	KY294129-30/KY294612-13
	other	2m, 3f	same data as previous except: “Hi12-14” D. Percy leg. (BMNH)	20.0841, -155.6752	KY294131-35/KY294614-18
	other	1m	Puu Makaala, Hawaii, USA, ex Metrosideros polymorpha, 6 March 2014, “Hi15-14” D. Percy leg. (BMNH)	19.5495, -155.2312	KY294136/KY294619
	other	1f	Gulch below Puu O Umi, Kohala, Hawaii, USA, ex Metrosideros polymorpha, 17 July 2013, “Hi35-13” D. Percy leg. (BMNH)	20.0630, -155.7189	KY294137/KY294621
	other	1f	Humuula trail, Hawaii, USA, ex Metrosideros polymorpha var. glaberrima, 20 July 2013, “Hi59-13” D. Percy leg. (BMNH)	19.9652, -155.3028	/KY294620
	other	1m	Kehena, 3200ft, Kohala, Hawaii, USA, on Melicope, 12 August 2010, “JG2” J. Giffin leg. (BMNH)	−	−
nigrilineatus	holotype	f	1868 lava flow, near Kahuku Ranch, SW Hawaii, USA, ex Metrosideros polymorpha var. incana, 9 July 2002, “421-02” D. Percy leg. (BMNH)	19.0600, -155.6950	KY293901/KY294377
	paratypes	2f	same data as holotype		−
kapo	holotype	f	Upper Hamakua Ditch, Kohala, Hawaii, USA, ex Metrosideros polymorpha var. polymorpha, 5 March 2014, “Hi10-14” D. Percy leg. (BMNH)	20.0853, -155.6778	KY293821/KY294298
proboscideus	holotype	m	Upper Hamakua Ditch, Kohala, Hawaii, USA, ex Metrosideros polymorpha var. polymorpha, 5 March 2014, “Hi10-14” D. Percy leg. (BMNH)	20.0853, -155.6778	KY294091/KY294568
	paratypes	1m, 2f, 9i	Puu O Umi, Kohala, Hawaii, USA, ex Metrosideros polymorpha var. polymorpha, 2 June 2011, “Hi07-11” D. Percy leg. (BMNH)	20.0701, -155.7240	KY294095-96/KY294573-74
	other	1i	Saddle Rd., 4000ft, Hawaii, USA, ex Metrosideros, 28 August 1973, J. Beardsley leg. (BPBM)	−	−
	other	4i	same data as previous except: 4500-5000ft, 16 April 1973, J. Beardsley leg. (BPBM)	−	−
	other	1f	Olaa Forest, Hawaii, USA, ex Metrosideros polymorpha, 27 July 2002, “434-02” D. Percy leg. (BMNH)	19.4496, -155.2041	−
	other	1f	Kipuka off Saddle Road, Hawaii, USA, ex Metrosideros polymorpha var. polymorpha, 4 March 2014, “Hi02-14” D. Percy leg. (BMNH)	19.6729, -155.3394	KY294090/KY294567
	other	2m, 2f	Koloko Drive, Honuaula, Hualalai, Hawaii, USA, ex Metrosideros polymorpha, 8 March 2014, “Hi30-14” D. Percy leg. (BMNH)	19.7079, -155.9243	KY294092-94/KY294569-72
proboscideus	other	2m, 1f	Kau, Hawaii, USA, ex Metrosideros polymorpha, 12 July 2013, “Hi05-13” D. Percy leg. (BMNH)	19.1883, -155.5850	KY294098-100/KY294576-78
	other	1m	Tree Planting Road, off Saddle Road, Hawaii, USA, ex Metrosideros polymorpha, 3 June 2011, “Hi09-11” D. Percy leg. (BMNH)	19.6840, -155.2927	KY294097/KY294575
	other	1m	same data as previous except: ex Metrosideros polymorpha var. polymorpha, 18 July 2002, “418-02” D. Percy leg. (BMNH)	19.6834, -155.2951	KY294101/KY294579
poliahu	holotype	m	Upper Hamakua Ditch, Kohala, Hawaii, USA, ex Metrosideros polymorpha var. glaberrima, 5 March 2014, “Hi08-14” D. Percy leg. (BMNH)	20.0693, -155.6697	KY294085/KY294562
	paratypes	1m, 2f	same data as holotype		KY294083-84, KY294086/KY294560-61, KY294563
	other	1f	same data as holotype except: ex Metrosideros polymorpha, “Hi11-14” D. Percy leg. (BMNH)	20.0852, -155.6791	KY294087/KY294564
	other	1m	same data as holotype except: ex Metrosideros polymorpha, “Hi12-14” D. Percy leg. (BMNH)	20.0841, -155.6752	KY294088/KY294565
	other	1f	Gulch below Puu O Umi, Kohala, Hawaii, USA, ex Metrosideros polymorpha, 17 July 2013, “Hi35-13” D. Percy leg. (BMNH)	20.0630, -155.7189	KY294089/KY294566
lona	holotype	m	Kamakou Preserve, Molokai, USA, on Polyscias, 17 August 2003, “Hi22-03” D. Percy leg. (BMNH)	21.1236, -156.9108	−
	paratype	1f	same data as holoptype		−
	paratype	1f	same data as holotype except: ex Metrosideros polymorpha var. glaberrima, “Hi21-03” D. Percy leg. (BMNH)	21.1236, -156.9108	−
	paratype	1f	same data as holotype except: ex Metrosideros polymorpha var. polymorpha, “Hi23-03” D. Percy leg. (BMNH)	21.1164, -156.9150	KY293833/KY294308
liliha	holotype	m	Mnt Kaala, boardwalk through bog, Waianae Mnts, Oahu, USA, ex Metrosideros polymorpha, 4 July 2014, “Hi57-14” D. Percy leg. (BMNH)	21.5071, -158.1440	KY293832/KY294307
	paratypes	3f	same data as holotype		KY293829-31/KY294304-06
gracilis	holotype	f	Kuliouou, Oahu, USA, ex Metrosideros polymorpha, 1916, #5524 O. Swezey leg. (BPBM)	−	−
	other	3i	Pahoa Flats, Oahu, USA, ex Metrosideros, 6 September 1973, J. Beardsley leg. (BPBM)	−	−
	other	13m, 6f	Palolo Ridge Trail, Koolau Mnts, Oahu, USA, ex Metrosideros polymorpha var. polymorpha, 14 May 2002, “351-02” D. Percy leg. (BMNH)	21.3260, -157.7790	−
gracilis	other	17m, 26f	Aiea Ridge Trail, Koolau Mnts, Oahu, USA, ex Metrosideros polymorpha var. glaberrima, 15 May 2002, “353/354-02” D. Percy leg. (BMNH)	21.4049, -157.8820	KY293752/KY294231
	other	12m, 11f	Puu Kaeo, Honolua Valley, West Maui, USA, ex Metrosideros polymorpha var. glaberrima, 23 July 2002, “428-02” D. Percy leg. (BMNH)	20.9580, -156.6110	KY293753/KY294232
	other	4m, 3f	Aiea Ridge Trail, Koolau Mnts, Oahu, USA, ex Metrosideros polymorpha var. glaberrima, 13 August 2003, “Hi01-03” D. Percy leg. (BMNH)	21.4050, -157.8819	−
	other	1m, 1f	same data as previous except: ex Metrosideros polymorpha var. polymorpha, “Hi03-03” D. Percy leg. (BMNH)	21.4050, -157.8819	−
	other	2m, 6f	Wiliwilinui Ridge Trail, Koolau Mnts, Oahu, USA, ex Metrosideros polymorpha var. polymorpha, 16 August 2003, “Hi15-03” D. Percy leg. (BMNH)	21.3167, -157.7597	KY293754/KY294233
	other	2m, 1f	same data as previous except: ex Metrosideros polymorpha var. glaberrima, “Hi16/17-03” D. Percy leg. (BMNH)	21.3183, -157.7592	−
	other	1m	Kamakou Preserve, Molokai, USA, ex Metrosideros polymorpha var. glaberrima, 17 August 2003, “Hi21-03” D. Percy leg. (BMNH)	21.1236, -156.9108	−
	other	2m, 4f	same data as previous except: “Hi26-03” D. Percy leg. (BMNH)	21.1164, -156.9150	KY293755/KY294234
	other	1m, 2f	Mnt Kaala road (culvert 55), Waianae Mnts, Oahu, USA, ex Metrosideros polymorpha var. glaberrima, 26 August 2003, “Hi48-03” D. Percy leg. (BMNH)	21.5110, -158.1500	−
	other	2m, 2f	Mnt Kaala summit, Waianae Mnts, Oahu, USA, ex Metrosideros polymorpha var. polymorpha, 26 August 2003, “Hi50-03” D. Percy leg. (BMNH)	21.5083, -158.1439	KY293756/KY294235
	other	2m, 4f	same data as previous except: ex Metrosideros polymorpha var. glaberrima, “Hi51-03” D. Percy leg. (BMNH)	21.5083, -158.1439	−
	other	14m, 17f, 20i	Aiea Ridge Trail, Koolau Mnts, Oahu, USA, ex Metrosideros polymorpha var. polymorpha, 25 May 2011, “Hi01-11” D. Percy leg. (BMNH)	21.4049, -157.8820	KY293750-51, KY293757/KY294229-30, KY294236
	other	11m, 5f, 1i	Mnt Kaala, boardwalk through bog, Waianae Mnts, Oahu, USA, ex Metrosideros polymorpha, 4 July 2014, “Hi61-14” D. Percy leg. (BMNH)	21.5028, -158.1483	KY293758-59/KY294237-38
	other	4m, 1f	Mnt Kaala summit, Waianae Mnts, Oahu, USA, ex Metrosideros polymorpha, 4 July 2014, “Hi62-14” D. Percy leg. (BMNH)	21.5078, -158.1439	KY293760-64/KY294239-43
	other	20m, 20f	Pupukea, N. Koolau Mnts, Oahu, USA, ex Metrosideros polymorpha, 8 July 2014, “Hi78-14” D. Percy leg. (BMNH)	21.6419, -158.0031	KY293765-66/KY294244-45
gracilis	other	4m, 9f	Manoa Cliff trail to Pauoa Flats trail, S. Koolau Mnts, Oahu, USA, ex Metrosideros polymorpha, 10 July 2014, “Hi82-14” D. Percy leg. (BMNH)	21.3455, -157.8062	KY293767-69/KY294246-48
	other	3m, 9f	same data as previous except: “Hi83-14” D. Percy leg. (BMNH)	21.3453, -157.8052	KY293770-71/KY294249-50
	other	1f	Mnt Kaala summit, Waianae Mnts, Oahu, USA, 4 July 2014, “KM02-14” K. Magnacca leg. (BMNH)	21.5036, -158.1476	−
	other	1m	same data as previous except: on Cheirodendron, “KM07-14” K. Magnacca leg. (BMNH)	21.5036, -158.1476	−
dorsostriatus	holotype	m	Puu O Umi NAR, Kohala, Hawaii, USA, ex Metrosideros polymorpha var. polymorpha, 2 June 2011, “Hi07-11” D. Percy leg. (BMNH)	20.0701, -155.7240	−
	paratype	1m	same data as holotype		KY293725/KY294204
	paratypes	3m, 5f, 20i	same data as holotype except: ex Metrosideros polymorpha var. glaberrima, “Hi06-11” D. Percy leg. (BMNH)	20.0701, -155.7240	KY293723-24/KY294202-03
	other	14i	Donkey Mill Rd., Kona, Hawaii, USA, ex Metrosideros, 29 August 1973, J. Beardsley leg. (BPBM)	19.5814, -155.9123	−
		1f	Olaa Forest, Hawaii, USA, ex Metrosideros polymorpha var. glaberrima, 24 August 2003, “Hi43-03” D. Percy leg. (BMNH)	19.4603, -155.2467	−
	other	1f	Puu Pili, 3900ft, Kohala, Hawaii, USA, on Metrosideros, 12 August 2010, “JG7” J. Giffin leg. (BMNH)	−	−
	other	3m	Kau, Hawaii, USA, ex Metrosideros polymorpha, 12 July 2013, “Hi04-13” D. Percy leg. (BMNH)	19.1747, -155.5884	KY293727/KY294206
	other	1m, 1f	Alili Spring trail, Kau, Hawaii, USA, ex Metrosideros polymorpha, 19 July 2013, “Hi47-13” D. Percy leg. (BMNH)	19.2333, -155.5208	KY293726/KY294205
	other	1m, 2f	Humuula trail, Hawaii, USA, ex Metrosideros polymorpha var. glaberrima, 20 July 2013, “Hi59-13” D. Percy leg. (BMNH)	19.9652, -155.3028	KY293728-30/KY294207-09
	other	1f, 3i	Upper Hamakua Ditch, Kohala, Hawaii, USA, ex Metrosideros polymorpha var. glaberrima, 5 March 2014, “Hi09-14” D. Percy leg. (BMNH)	20.0714, -155.6711	KY293731-32/KY294210-11
	other	2f	same data as previous except: ex Metrosideros polymorpha, “Hi11-14” D. Percy leg. (BMNH)	20.0852, -155.6791	KY293733-34/KY294212-13
	other	1f	same data as previous except: “Hi12-14” D. Percy leg. (BMNH)	20.0841, -155.6752	KY293734/KY294214
dorsostriatus	other	1m, 1f	Puu Makaala, Hawaii, USA, ex Metrosideros polymorpha var. glaberrima, 6 March 2014, “Hi15-14” D. Percy leg. (BMNH)	19.5495, -155.2312	KY293736-37/KY294215-16
	other	3m, 3i	same data as previous except: “Hi16-14” D. Percy leg. (BMNH)	19.5516, -155.2308	KY293738-41/KY294217-20
	other	1m	same data as previous except: “Hi17-14” D. Percy leg. (BMNH)	19.5522, -155.2310	KY293742/KY294221
namaka	holotype	m	Mnt Kaala, boardwalk through bog, Waianae Mnts, Oahu, USA, ex Metrosideros polymorpha, 4 July 2014, “Hi57-14” D. Percy leg. (BMNH)	21.5071, -158.1440	KY293899/KY294373
	paratypes	1f, 8i	same data as holotype	−	KY293898-900/KY294372, KY294374-76
**minutus**				−	−
minutus	holotype	m	Kilauea, Hawaii, USA, ex Ohia Lehua, 27 June 1917, O. Swezey leg. (BPBM)	−	−
	other	2m, 1f	Southern Belt road (93 mile marker to Hilo), Hawaii, USA, Metrosideros polymorpha var. polymorpha, 29 May 2002, “387-02” D. Percy leg. (BMNH)	19.2680, -155.8750	−
	other	1m	Saddle Road (near 22 mile marker), Hawaii, USA, ex Metrosideros polymorpha var. polymorpha, 30 May 2002, “391-02” D. Percy leg. (BMNH)	19.6765, -155.3802	−
	other	20m, 20f	Tree Planting Road, off Saddle Road, Hawaii, USA, ex Metrosideros polymorpha var. polymorpha, 18 July 2002, “418-02” D. Percy leg. (BMNH)	19.6834, -155.2951	KY293871/KY294346
	other	1m, 1f	Olaa Forest near Solid Waste Dump, Hawaii, USA, ex Metrosideros polymorpha var. glaberrima, 21 August 2003, “Hi36-03” D. Percy leg. (BMNH)	19.4500, -155.2042	−
	other	1m	same data as previous except: 22 August 2003, “Hi37-03” D. Percy leg. (BMNH)	19.4500, -155.2042	/KY294339
	other	7m, 5f, 3i	Tree Planting Road, off Saddle Road, Hawaii, USA, ex Metrosideros polymorpha var. polymorpha, 25 August 2003, “Hi46-03” D. Percy leg. (BMNH)	19.6833, -155.2950	KY293872/KY294347
	other	1m, 20i	Kilauea Iki Crater, Hawaii, USA, ex Metrosideros polymorpha, 30 May 2011, “Hi02-11” D. Percy leg. (BMNH)	19.4130, -155.2460	KY293865/KY294340
	other	2m, 1f	Puu Oo Trail, off Saddle Road, Hawaii, USA, ex Metrosideros polymorpha, 31 May 2011, “Hi03-11” D. Percy leg. (BMNH)	19.6732, -155.3889	−
	other	10m, 6f, 2i	Tree Planting Road, off Saddle Road, Hawaii, USA, ex Metrosideros polymorpha, 3 June 2011, “Hi09-11” D. Percy leg. (BMNH)	19.6840, -155.2927	KY293866-68/KY294341-43
minutus	other	2m, 2f	Kau, Hawaii, USA, ex Metrosideros polymorpha, 12 July 2013, “Hi05-13” D. Percy leg. (BMNH)	19.1883, -155.5850	KY293869-70/KY294344-45
	other	3i	Tree Planting Road (1850-1880 flows), off Saddle Road, Hawaii, USA, ex Metrosideros polymorpha var. incana and var. glaberrima, 14 July 2013, “Hi17-13” D. Percy leg. (BMNH)	19.6642, -155.2783	−
	other	1m, 1f	Kipuka off Saddle Road, Hawaii, USA, ex Metrosideros polymorpha var. polymorpha, 4 March 2014, “Hi02-14” D. Percy leg. (BMNH)	19.6729, -155.3394	KY293849-50/KY294323-24
	other	10m, 1f	Upper Hamakua Ditch, Kohala, Hawaii, USA, ex Metrosideros polymorpha var. glaberrima, 5 March 2014, “Hi08-14” D. Percy leg. (BMNH)	20.0693, -155.6697	KY293851-54/KY294325-28
	other	4m, 3f	same data as previous except: ex Metrosideros polymorpha var. polymorpha, “Hi10-14” D. Percy leg. (BMNH)	20.0853, -155.6778	KY293855-58/KY294329-32
	other	2m, 3f	same data as previous except: ex Metrosideros polymorpha, “Hi11-14” D. Percy leg. (BMNH)	20.0852, -155.6791	KY293859-60/KY294333-34
	other	1m, 1f	same data as previous except: “Hi12-14” D. Percy leg. (BMNH)	20.0841, -155.6752	KY293861/KY294335
	other	3m, 3i	Puu Makaala, Hawaii, USA, ex Metrosideros polymorpha var. glaberrima, 6 March 2014, “Hi16-14” D. Percy leg. (BMNH)	19.5516, -155.2308	−
	other	3f	Kona Hema TNC, Hawaii, USA, ex Metrosideros polymorpha, 7 March 2014, “Hi26-14” D. Percy leg. (BMNH)	19.2226, -155.8308	KY293862-64/KY294336-38
	other	4i	Donkey Mill Rd., Kona, Hawaii, USA, ex Metrosideros, 29 August 1973, J. Beardsley leg. (BPBM)	19.5814, -155.9123	−
gibbosus	holotype	m	Olinda Flume Road, Makawao, East Maui, USA, ex Metrosideros polymorpha, 3 July 2014, “Hi53-14” D. Percy leg. (BMNH)	20.8106, -156.2398	KY293749/KY294228
	paratypes	1m, 2f	same data as holotype		KY293746-48/KY294225-27
	paratypes	2f	same data as previous except: “Hi52-14” D. Percy leg. (BMNH)	20.8099, -156.2501	KY293744-45/KY294223-24
	paratype	1f	same data as previous except: “Hi41-14” D. Percy leg. (BMNH)	20.8115, -156.2381	KY293743/KY294222
**kamua**					
iolani	holotype	f	Halemanu, Kauai, Sandwich Is., USA, 1933-323, 4000 ft, R. Perkins leg. (BMNH)	−	−
	syntype	f	Kokee, Kauai, USA, on Dodonaea, 12 August 1921, #5518, O. Swezey leg. (BPBM)	−	−
iolani	other	3m, 1f	Discovery Center, Kokee State Park, Kauai, USA, ex Metrosideros polymorpha var. glaberrima, 24 May 2002, “365-02” D. Percy leg. (BMNH)	22.1337, -159.6501	−
	other	1m, 2f	Kalalau Valley (close to 2nd lookout), Kokee State Park, Kauai, USA, ex Metrosideros polymorpha var. glaberrima, 26 May 2002, “375-02” D. Percy leg. (BMNH)	22.1490, -159.6320	−
	other	2m, 3f	Kokee State Park (near NASA Geophysical Observatory, ~14 mile marker), Kauai, USA, ex Metrosideros polymorpha var. glaberrima, 28 May 2002, “385-02” D. Percy leg. (BMNH)	22.1184, -159.6676	KY293819/KY294296
	other	1m	Kokee State Park, Kauai, USA, ex Metrosideros polymorpha, 29 October 2005, “Hi01-05” D. Percy leg. (BMNH)	22.1444, -159.6477	−
	other	1f	Alakai Swamp trail, Kokee State Park, Kauai, USA, on Melicope, 31 October 2005, “Hi07-05” D. Percy leg. (BMNH)	22.1397, -159.6239	KY293820/KY294297
hiiaka	holotype	m	Kalalau Valley (near 2nd lookout), Kokee State Park, Kauai, USA, ex Metrosideros polymorpha var. glaberrima, 26 May 2002, “377-02” D. Percy leg. (BMNH)	22.1490, -159.6320	−
	paratypes	10m, 6f	same data as holotype		−
	other	4i	Kokee State Park (Puu o Kilo, at Kahuamma Flat), Kauai, USA, ex Metrosideros polymorpha var. glaberrima and var. polymorpha, 29 October 2005, “Hi03-05” D. Percy leg. (BMNH)	22.1491, -159.6375	KY293794/KY294273
	other	10i	Alakai Swamp trail, Kokee State Park, Kauai, USA, ex Metrosideros polymorpha var. glaberrima, 31 October 2005, “Hi06-05” D. Percy leg. (BMNH)	22.1359, -159.6257	KY293795-96/KY294274
	other	4i	Alakai Swamp trail (margin of 1st bog, Lehua Maka Noe), Kokee State Park, Kauai, USA, ex Metrosideros polymorpha var. glaberrima, 31 October 2005, “Hi08-05” D. Percy leg. (BMNH)	22.1451, -159.6202	KY293797-99/KY294275-76
	other	12i	same data as previous except: “Hi09-05” D. Percy leg. (BMNH)	22.1469, -159.6152	KY293800-02/KY294277-78
	other	28i	Puu Ka Pele Forest Reserve, Hikimoe valley, Kauai, USA, ex Metrosideros polymorpha var. glaberrima, 1 November 2005, “Hi10-05” D. Percy leg. (BMNH)	22.0948, -159.6953	KY293803/
	other	1m	Awaawapuhi trail, Kokee State Park, Kauai, USA, ex Metrosideros polymorpha var. glaberrima, 02 November 2005, “Hi14-05” D. Percy leg. (BMNH)	22.1426, -159.6536	−
melanoneurus	holotype	m	Kalalau Valley (between 1st and 2nd lookout), Kokee State Park, Kauai, USA, ex Metrosideros polymorpha, 28 May 2002, “384-02” D. Percy leg. (BMNH)	22.1510, -159.6380	−
	paratypes	3m, 3f	same data as holotype		KY293848/KY294322
grandis	holotype	m	Kalalau Valley (between 1st and 2nd lookout), Kokee State Park, Kauai, USA, ex Metrosideros polymorpha, 28 May 2002, “384-02” D. Percy leg. (BMNH)	22.1510, -159.6380	−
grandis	paratype	1f	same data as holotype		−
caulicalix	holotype	m	Kalalau Valley (between 1st and 2nd lookout), Kokee State Park, Kauai, USA, ex Metrosideros polymorpha, 28 May 2002, “384-02” D. Percy leg. (BMNH)	22.1510, -159.6380	−
	paratypes	6m, 13f	same data as holotype		KY293715/KY294194
	other	5m, 4f	Discovery Center, Kokee State Park, Kauai, USA, ex Metrosideros polymorpha var. glaberrima, 24 May 2002, “365-02” D. Percy leg. (BMNH)	22.1337, -159.6501	−
	other	2f	Kalalau Valley (close to 2nd lookout), Kokee State Park, Kauai, USA, ex Metrosideros polymorpha var. glaberrima, 26 May 2002, “375-02” D. Percy leg. (BMNH)	22.1490, -159.6320	−
	other	6m, 9f	Kalalau Valley (near 2nd lookout), Kokee State Park, Kauai, USA, ex Metrosideros polymorpha var. glaberrima, 26 May 2002, “377-02” D. Percy leg. (BMNH)	22.1490, -159.6320	−
	other	9m, 7f	Kokee State Park (near NASA Geophysical Observatory, ~14 mile marker), Kauai, USA, ex Metrosideros polymorpha var. glaberrima, 28 May 2002, “385-02” D. Percy leg. (BMNH)	22.1184, -159.6676	KY293716/KY294195
	other	17i	Kokee State Park (Puu o Kilo, at Kahuamma Flat), Kauai, USA, ex Metrosideros polymorpha var. glaberrima, 29 October 2005, “Hi03-05” D. Percy leg. (BMNH)	22.1491, -159.6375	KY293718-19/KY294197-98
	other	25i	Alakai Swamp trail (margin of 1st bog, Lehua Maka Noe), Kokee State Park, Kauai, USA, ex Metrosideros polymorpha var. glaberrima, 31 October 2005, “Hi09-05” D. Percy leg. (BMNH)	22.1469, -159.6152	KY293717/KY294196
	other	1f	Puu Ka Pele Forest Reserve, Hikimoe valley, Kauai, USA, ex Metrosideros polymorpha var. glaberrima, 1 November 2005, “Hi10-05” D. Percy leg. (BMNH)	22.0948, -159.6953	−
crassiorcalix	holotype	m	Alakai Swamp trail, 1st bog (Lehua Maka Noe), Kauai, USA, ex Metrosideros polymorpha var. pumila, 31 October 2005, “Hi08-05” D. Percy leg. (BMNH)	22.1451, -159.6202	−
	paratypes	8m, 7f, 20i	same data as holotype		KY293720-22/KY294199-201
lehua	holotype	m?	Nualolo, Kauai, USA, ex Ohia Lehua, 1 September 1921, #5520 O. Swezey leg. (BPBM)	−	−
	other	1f	same data as holotype		−
	other	2m, 1f	Alakai Swamp trail, 1st bog (Lehua Maka Noe), Kauai, USA, ex Metrosideros polymorpha var. pumila, 31 October 2005, “Hi08-05” D. Percy leg. (BMNH)	22.1451, -159.6202	−
elegans	holotype	f	Kalalau Valley (near 2nd lookout), Kokee State Park, Kauai, USA, ex Metrosideros polymorpha var. glaberrima, 26 May 2002, “377-02” D. Percy leg. (BMNH)	22.1490, -159.6320	−
gagneae	holotype	f	Kalalau Valley (between 1st and 2nd lookout), Kokee State Park, Kauai, USA, ex Metrosideros polymorpha, 28 May 2002, “384-02” D. Percy leg. (BMNH)	22.1510, -159.6380	−
haumea	holotype	m	Alakai Swamp trail (margin of 1st bog, Lehua Maka Noe), Kokee State Park, Kauai, USA, ex Metrosideros polymorpha var. glaberrima, 31 October 2005, “Hi09-05” D. Percy leg. (BMNH)	22.1469, -159.6152	−
**ohialoha**					
oahuensis	holotype	m	Aiea Ridge Trail, Koolau Mnts, Oahu, USA, ex Metrosideros polymorpha var. glaberrima, 15 May 2002, “353/354-02” D. Percy leg. (BMNH)	21.4049, -157.8820	−
	paratypes	8m, 10f, 1i	same data as holotype		KY293942-43/KY294417-18
	other	3m	Kuliouou Ridge Trail, Koolau Mnts, Oahu, USA, ex Metrosideros polymorpha var. polymorpha, 12 May 2002, “348-02” D. Percy leg. (BMNH)	21.3194, -157.7231	−
	other	4m, 10f, 6i	Palolo Ridge Trail, Koolau Mnts, Oahu, USA, ex Metrosideros polymorpha var. polymorpha, 14 May 2002, “351-02” D. Percy leg. (BMNH)	21.3260, -157.7790	KY293948/KY294423
	other	1m, 1f, 13i	Aiea Ridge Trail, Koolau Mnts, Oahu, USA, ex Metrosideros polymorpha var. polymorpha, 15 May 2002, “355-02” D. Percy leg. (BMNH)	21.4049, -157.8820	KY293946, KY293949-50/KY294421, KY294424-25
	other	7m, 4f	same data as previous except: ex Metrosideros polymorpha var. glaberrima, “356-02” D. Percy leg. (BMNH)	21.4049, -157.8820	−
	other	1m, 1f	Honouliuli Preserve, Waianae Mnts, Oahu, USA, ex Metrosideros polymorpha var. glaberrima, 22 May 2002, “362-02” D. Percy leg. (BMNH)	21.4360, -158.0920	−
	other	12m, 13f	Pupukea, Koolau Mnts, Oahu, USA, ex Metrosideros polymorpha, 23 July 2002, “SLM288-02” S. Montgomery leg. (BMNH)	21.6370, -157.9970	KY293941/KY294416
	other	1m	Aiea Ridge Trail, Koolau Mnts, Oahu, USA, ex Metrosideros polymorpha var. glaberrima, 13 August 2003, “Hi01-03” D. Percy leg. (BMNH)	21.4050, -157.8819	
	other	1i	Pahole NAR, Waianaea Mnts, Oahu, USA, ex Metrosideros polymorpha var. glaberrima, 14 August 2003, “Hi04-03” D. Percy leg. (BMNH)	21.5372, -158.1922	−
	other	1m	same data as previous except: on Planchonella, “Hi06-03” D. Percy leg. (BMNH)	21.5364, -158.1919	−
	other	1m, 1f	same data as previous except: on Melicope, “Hi07-03” D. Percy leg. (BMNH)	21.5364, -158.1919	−
	other	3m, 2f	same data as previous except: ex Metrosideros polymorpha var. glaberrima, “Hi10-03” D. Percy leg. (BMNH)	21.5461, -158.1953	KY293938/KY294413
	other	7m, 7f, 50i	Wiliwilinui Ridge Trail, Koolau Mnts, Oahu, USA, ex Metrosideros polymorpha var. polymorpha, 16 August 2003, “Hi14/15-03” D. Percy leg. (BMNH)	21.3167, -157.7597	KY293947/KY294422
oahuensis	other	1m, 2f	same data as previous except: ex Metrosideros polymorpha var. glaberrima, “Hi16/17-03” D. Percy leg. (BMNH)	21.3183, -157.7592	−
	other	2m, 3f, 42i	Mnt Kaala summit, Waianae Mnts, Oahu, USA, ex Metrosideros polymorpha var. polymorpha, 26 August 2003, “Hi50-03” D. Percy leg. (BMNH)	21.5083, -158.1439	KY293939, KY293944/KY294414, KY294419
	other	2m, 3f	same data as previous except: ex Metrosideros polymorpha var. glaberrima, “Hi51-03” D. Percy leg. (BMNH)	21.5083, -158.1439	−
	other	2m, 1f	Aiea Ridge Trail, Koolau Mnts, Oahu, USA, ex Metrosideros polymorpha var. polymorpha, 25 May 2011, “Hi01-11” D. Percy leg. (BMNH)	21.4049, -157.8820	KY293936-37, KY293945/KY294411-12, KY294420
	other	1f	Mnt Kaala, Waianae Mnts, Oahu, USA, ex Metrosideros polymorpha, 2 February 2011, “SLM2-11” S. Montgomery leg. (BMNH)	−	KY293940/KY294415
	other	2m, 3f	Mnt Kaala, boardwalk through bog, Waianae Mnts, Oahu, USA, ex Metrosideros polymorpha, 4 July 2014, “Hi57-14” D. Percy leg. (BMNH)	21.5071, -158.1440	KY293902-04/KY294378-80
	other	3m, 2f	Mnt Kaala, boardwalk through bog, Waianae Mnts, Oahu, USA, ex Metrosideros polymorpha, 4 July 2014, “Hi61-14” D. Percy leg. (BMNH)	21.5028, -158.1483	KY293905-09/KY294381-85
	other	2m	Mnt Kaala road, Waianae Mnts, Oahu, USA, ex Metrosideros polymorpha, 04 July 2014, “Hi63-14” D. Percy leg. (BMNH)	21.5100, -158.1474	KY293910-11/KY294386-87
	other	2m, 5f	same data as previous except: “Hi64-14” D. Percy leg. (BMNH)	21.5164, -158.1650	KY293912-18/KY294388-94
	other	4m, 3f	Palikea trail, Waianae Mnts, Oahu, USA, ex Metrosideros polymorpha, 05 July 2014, “Hi67-14” D. Percy leg. (BMNH)	21.4105, -158.0987	KY293919-21/KY294395-97
	other	4m, 4f	Mokuleia Forest Reserve, Pahole NAR, Waianae Mnts, Oahu, USA, ex Metrosideros polymorpha, 6 July 2014, “Hi70-14” D. Percy leg. (BMNH)	21.5381, -158.1813	KY293922-27/KY294398-403
	other	1m, 1f	same data as previous except: “Hi72-14” D. Percy leg. (BMNH)	21.5345, -158.1810	−
	other	2f	Pupukea, Koolau Mnts, Oahu, USA, ex Metrosideros polymorpha, 8 July 2014, “Hi76-14” D. Percy leg. (BMNH)	21.6375, -158.0120	−
	other	4m, 7f, 3i	same data as previous except: “Hi78-14” D. Percy leg. (BMNH)	21.6419, -158.0031	KY293928-35/KY294404-10
	other	9m, 5f	Manoa Cliff trail to Pauoa Flats trail, Koolau Mnts, Oahu, USA, ex Metrosideros polymorpha, 10 July 2014, “Hi80-14” D. Percy leg. (BMNH)	21.3374, -157.8111	−
oahuensis	other	9m, 16f	same data as previous except: “Hi83-14” D. Percy leg. (BMNH)	21.3453, -157.8052	−
	other	1m, 1f	Mnt Kaala, Waianae Mnts, Oahu, USA, ex Metrosideros polymorpha, 4 July 2014, “KM08-14” K. Magnacca leg. (BMNH)	21.5141, -158.1614	−
	other	1m	same data as previous except: “KM11-14” K. Magnacca leg. (BMNH)	21.5036, -158.1476	−
ohiacola	holotype	m	Mnt Kaala, 1600ft, Oahu, USA, ex Metrosideros, #5521 Pariaconus Timberlake leg. (BPBM)	−	−
	other	10i	Palolo Ridge Trail, Koolau Mnts, Oahu, USA, ex Metrosideros polymorpha var. polymorpha, 14 May 2002, “351-02” D. Percy leg. (BMNH)	21.3260, -157.7790	−
	other	17m, 18f	Aiea Ridge Trail, Koolau Mnts, Oahu, USA, ex Metrosideros polymorpha var. glaberrima, 15 May 2002, “353/354-02” D. Percy leg. (BMNH)	21.4049, -157.8820	KY294005/KY294482
	other	3m, 3f	same data as previous except: “356-02” D. Percy leg. (BMNH)	21.4049, -157.8820	−
	other	9m, 9f	Honouliuli Preserve, Waianae Mnts, Oahu, USA, ex Metrosideros polymorpha var. glaberrima, 22 May 2002, “361/362-02” D. Percy leg. (BMNH)	21.4360, -158.0920	−
	other	7m, 4f	Pupukea, Koolau Mnts, Oahu, USA, ex Metrosideros polymorpha, 23 July 2002, “SLM288-02” S. Montgomery leg. (BMNH)	21.6370, -157.9970	KY294004/KY294481
	other	15m, 9f	Aiea Ridge Trail, Koolau Mnts, Oahu, USA, ex Metrosideros polymorpha var. glaberrima, 13 August 2003, “Hi01-03” D. Percy leg. (BMNH)	21.4050, -157.8819	−
	other	1m, 1f	same data as previous except: ex Metrosideros polymorpha var. polymorpha, “Hi03-03” D. Percy leg. (BMNH)	21.4050, -157.8819	−
	other	2m, 1f	Pahole NAR, Waianaea Mnts, Oahu, USA, ex Metrosideros polymorpha var. glaberrima, 14 August 2003, “Hi10-03” D. Percy leg. (BMNH)	21.5461, -158.1953	−
	other	18m, 8f	Wiliwilinui Ridge Trail, Koolau Mnts, Oahu, USA, ex Metrosideros polymorpha var. glaberrima, 16 August 2003, “Hi16/17-03” D. Percy leg. (BMNH)	21.3183, -157.7592	−
	other	7m, 18f	Mnt Kaala road (culvert 55), Waianae Mnts, Oahu, USA, ex Metrosideros polymorpha var. glaberrima, 26 August 2003, “Hi48-03” D. Percy leg. (BMNH)	21.5110, -158.1500	KY293952/KY294427-30
	other	67m, 32f	Mnt Kaala summit, Waianae Mnts, Oahu, USA, ex Metrosideros polymorpha var. glaberrima, 26 August 2003, “Hi51-03” D. Percy leg. (BMNH)	21.5083, -158.1439	KY293953-54, KY294009/KY294431, KY294486
	other	5m, 7f, 20i	Aiea Ridge Trail, Koolau Mnts, Oahu, USA, ex Metrosideros polymorpha var. polymorpha, 25 May 2011, “Hi01-11” D. Percy leg. (BMNH)	21.4049, -157.8820	KY293996-4001/KY294473-78
ohiacola	other	8i	Koolau Mnts, Oahu, USA, ex Metrosideros macropus, 18 June 2011, “JL1-11” J. Lau leg. (BMNH)	−	KY294006/KY294483
	other	4m, 3f	Mnt Kaala, Waianae Mnts, Oahu, USA, ex Metrosideros polymorpha, 2 February 2011, “SLM2-11” S. Montgomery leg. (BMNH)	−	KY294002-03/KY294479-80
	other	7i	Palikoa, S. Waianae, Oahu, USA, ex Metrosideros polymorpha var. glaberrima, 23 May 2011, “SLM3-11” S. Montgomery leg. (BMNH)	−	KY294007-08/KY294484-85
	other	1i	Kuliouou, Koolau Mnts, Oahu, USA, ex Metrosideros polymorpha var. glaberrima, 22 February 2012, “ES1-12” E. Stacy leg. (BMNH)	21.3219, -157.7307	KY293951/KY294426
	other	1i	same data as previous except: ex Metrosideros rugosa, “ES2-12” E. Stacy leg. (BMNH)	21.3219, -157.7307	KY293993/KY294470
	other	3i	same data as previous except: ex Metrosideros polymorpha var. incana, “ES5/6-12” E. Stacy leg. (BMNH)	21.3219, -157.7307	KY293994-95/KY294471-72
	other	13m, 1f	Mnt Kaala, boardwalk through bog, Waianae Mnts, Oahu, USA, ex Metrosideros polymorpha, 4 July 2014, “Hi57-14” D. Percy leg. (BMNH)	21.5071, -158.1440	KY293955-58/KY294432-35
	other	4m, 1f	same data as previous except: “Hi59-14” D. Percy leg. (BMNH)	21.5053, -158.1465	KY293959-61/KY294436-38
	other	8m, 3f	Mnt Kaala, boardwalk through bog, Waianae Mnts, Oahu, USA, ex Metrosideros polymorpha, 4 July 2014, “Hi61-14” D. Percy leg. (BMNH)	21.5028, -158.1483	KY293962-63/KY294439-40
	other	1m	Mnt Kaala summit, Waianae Mnts, Oahu, USA, ex Metrosideros polymorpha, 4 July 2014, “Hi62-14” D. Percy leg. (BMNH)	21.5078, -158.1439	KY293964/KY294441
	other	2m, 2f	Mnt Kaala road, Waianae Mnts, Oahu, USA, ex Metrosideros polymorpha, 04 July 2014, “Hi63-14” D. Percy leg. (BMNH)	21.5100, -158.1474	KY293965-68/KY294442-45
	other	7m, 4f	same data as previous except: “Hi64-14” D. Percy leg. (BMNH)	21.5164, -158.1650	KY293969-70/KY294446-47
	other	5m, 4f	Waianae Mnts, Oahu, USA, ex Metrosideros polymorpha, 5 July 2014, “Hi65-14” D. Percy leg. (BMNH)	21.4585, -158.0973	KY293971-73/KY294448-50
	other	9m, 3f	Palikea trail, Waianae Mnts, Oahu, USA, ex Metrosideros polymorpha, 05 July 2014, “Hi67-14” D. Percy leg. (BMNH)	21.4105, -158.0987	KY293974-80/KY294451-57
	other	16m, 20f	Mokuleia Forest Reserve, Pahole NAR, Waianae Mnts, Oahu, USA, ex Metrosideros polymorpha, 6 July 2014, “Hi70-14” D. Percy leg. (BMNH)	21.5381, -158.1813	KY293981-84/KY294458-61
ohiacola	other	2m, 2i	Pupukea, N. Koolau Mnts, Oahu, USA, ex Metrosideros polymorpha, 8 July 2014, “Hi78-14” D. Percy leg. (BMNH)	21.6419, -158.0031	KY293985-86/KY294462-63
	other	12m, 15f	Manoa Cliff trail to Pauoa Flats trail, S. Koolau Mnts, Oahu, USA, ex Metrosideros polymorpha, 10 July 2014, “Hi83-14” D. Percy leg. (BMNH)	21.3453, -157.8052	KY293987-89/KY294464-66
	other	5m, 13f	same data as previous except: “Hi 84-14” D. Percy leg. (BMNH)	21.3452, -157.8048	KY293990-92/KY294467-69
	other	2f	Mnt Kaala, Waianae Mnts, Oahu, USA, ex Metrosideros polymorpha, 4 July 2014, “KM08-14” K. Magnacca leg. (BMNH)	21.5141, -158.1614	−
	other	2m, 1f	same data as previous except: “KM09/11-14” K. Magnacca leg. (BMNH)	21.5036, -158.1476	−
lanaiensis	holotype	1f	3400ft, Lanai, USA, ex Ohia Lehua, 19 January 1917, #5519 W. Giffard leg. (BPBM)	−	−
molokaiensis	holotype	f	Kamiloloa, Molokai, USA, 3200 ft, on Coprosma, 20 December 1925, #1699 O. Swezey leg. (BPBM)	−	−
	other	1m, 1f	Kamakou Preserve, Molokai, USA, on Planchonella, 17 August 2003, “Hi20-03” D. Percy leg. (BMNH)	21.1236, -156.9108	−
	other	3m, 3f	same data as previous except: ex Metrosideros polymorpha var. glaberrima, “Hi21-03” D. Percy leg. (BMNH)	21.1236, -156.9108	−
	other	1f	same data as previous except: on Polyscias, “Hi22-03” D. Percy leg. (BMNH)	21.1236, -156.9108	−
	other	1m, 2f	same data as previous except: ex Metrosideros polymorpha var. polymorpha, “Hi23-03” D. Percy leg. (BMNH)	21.1164, -156.9150	−
	other	7m, 4f	same data as previous except: ex Metrosideros polymorpha var. glaberrima, “Hi26-03” D. Percy leg. (BMNH)	21.1164, -156.9150	KY293873/KY294348
	other	1f	same data as previous except: ex Metrosideros polymorpha var. incana, “Hi28-03” D. Percy leg. (BMNH)	21.1164, -156.9150	−
	other	4m, 4f	same data as previous except: ex Metrosideros waialealae var. fauriei, “Hi30-03” D. Percy leg. (BMNH)	21.1225, -156.9175	KY293874/KY294349
hualani	holotype	m	Kamakou Preserve, Molokai, USA, ex Metrosideros waialealae var. fauriei, 17 August 2003, “Hi30-03” D. Percy leg. (BMNH)	21.1225, -156.9175	−
	paratypes	3f	same data as holotype		KY293817/KY294294
hualani	other	1f	Kamakou Preserve, Molokai, USA, on Planchonella, 17 August 2003, “Hi20-03” D. Percy leg. (BMNH)	21.1236, -156.9108	−
	other	8m, 17f	same data as previous except: ex Metrosideros polymorpha var. glaberrima, “Hi21-03” D. Percy leg. (BMNH)	21.1236, -156.9108	KY293818/KY294295
	other	2m, 1f	same data as previous except: on Polyscias, “Hi22-03” D. Percy leg. (BMNH)	21.1236, -156.9108	−
	other	1f	same data as previous except: ex Metrosideros polymorpha var. polymorpha, “Hi23-03” D. Percy leg. (BMNH)	21.1164, -156.9150	−
mauiensis	holotype	m	Olinda Flume Road, Makawao, East Maui, USA, ex Metrosideros polymorpha, 25 July 2002, “429-02” D. Percy leg. (BMNH)	20.8047, -156.2608	−
	paratypes	6m, 6f	same data as holotype		KY293847/
	other	1m, 1f	Puu Kukui, boardwalk trail, West Maui, USA, ex Metrosideros polymorpha, 2 July 2014, “Hi47-14” D. Percy leg. (BMNH)	20.9342, -156.6142	KY293834-36/KY294309-11
	other	1m	same data as previous except: “Hi49-14” D. Percy leg. (BMNH)	20.9339, -156.6132	KY293837/KY294312
	other	1m	Olinda Flume Road, Makawao, East Maui, USA, ex Metrosideros polymorpha, 3 July 14, “Hi52-14” D. Percy leg. (BMNH)	20.8099, -156.2501	KY293838/KY294313
	other	3m, 5f	same data as previous except: “Hi53-14” D. Percy leg. (BMNH)	20.8106, -156.2398	KY293839-46/KY294314-21
kupua	holotype	m	Puu Kaeo, Honolua Valley, West Maui, USA, ex Metrosideros polymorpha var. glaberrima, 23 July 2002, ”428-02” D. Percy leg. (BMNH)	20.9580, -156.6110	−
	paratypes	4m, 6i	same data as holotype		KY293827-28
	other	1m, 1f	Puu Kukui, boardwalk trail, West Maui, USA, ex Metrosideros polymorpha, 2 July 2014, “Hi47-14” D. Percy leg. (BMNH)	20.9342, -156.6142	KY293822/KY294299
	other	1f	same data as previous except: “Hi48-14” D. Percy leg. (BMNH)	20.9343, -156.6137	KY293823/KY294300
	other	1m	same data as previous except: “Hi49-14” D. Percy leg. (BMNH)	20.9339, -156.6132	−
	other	3m	Olinda Flume Road, Makawao, East Maui, USA, ex Metrosideros polymorpha, 3 July 14, “Hi52-14” D. Percy leg. (BMNH)	20.8099, -156.2501	KY293824/KY294301
kupua	other	1m, 3f	same data as previous except: “Hi53-14” D. Percy leg. (BMNH)	20.8106, -156.2398	KY293825-26/KY294302-03
montgomeri	holotype	m	Puu Kaeo, Honolua Valley, West Maui, USA, ex Metrosideros polymorpha var. glaberrima, 23 July 2002, “428-02” D. Percy leg. (BMNH)	20.9580, -156.6110	−
	paratypes	11m, 11f, 55i	same data as holotype		KY293895-96/KY294370
	other	4m	Olinda Flume Road, Makawao, East Maui, USA, ex Metrosideros polymorpha, 25 July 2002, “429-02” D. Percy leg. (BMNH)	20.8047, -156.2608	KY293897/KY294371
	other	11i	Puu Kukui, boardwalk trail, West Maui, USA, ex Metrosideros polymorpha, 2 July 2014, “Hi45-14” D. Percy leg. (BMNH)	20.9344, -156.6164	KY293875/KY294350
	other	1f, 3i	same data as previous except: “Hi46-14” D. Percy leg. (BMNH)	20.9342, -156.6147	−
	other	7m, 2f	same data as previous except: “Hi47-14” D. Percy leg. (BMNH)	20.9342, -156.6142	KY293876-79/KY294351-54
	other	3m	same data as previous except: “Hi48-14” D. Percy leg. (BMNH)	20.9343, -156.6137	KY293880-82/KY294355-57
	other	1m, 8f	same data as previous except: “Hi49-14” D. Percy leg. (BMNH)	20.9339, -156.6132	KY293883-84/KY294358-59
	other	1m, 2f	same data as previous except: “Hi51-14” D. Percy leg. (BMNH)	20.9297, -156.6096	KY293885-86/KY294360-61
	other	10m, 8f	Olinda Flume Road, Makawao, East Maui, USA, ex Metrosideros polymorpha, 3 July 14, “Hi52-14” D. Percy leg. (BMNH)	20.8099, -156.2501	KY293887-93/KY294362-68
	other	2m, 1f	same data as previous except: “Hi53-14” D. Percy leg. (BMNH)	20.8106, -156.2398	KY293894/KY294369
hawaiiensis	holotype	m (?)	Kilauea, near Volcano, 4000ft, Hawaii, USA, 21 August 1917, #5517 W. Giffard leg. (BPBM)	−	−
	other	3i	Kilauea, Hawaii, USA, ex Metrosideros, July 1973, J. Beardsley leg. (BPBM)	−	−
	other	1m, 2f	Southern Belt road (93-95 mile marker to Hilo), Hawaii, USA, Metrosideros polymorpha var. polymorpha, 29 May 2002, “386/387-02” D. Percy leg. (BMNH)	19.2680, -155.8750	KY293777/KY294256
hawaiiensis	other	1m, 3f	Southern Belt road (91 mile marker to Hilo), Hawaii, USA, Metrosideros polymorpha var. polymorpha, 29 May 2002, “388-02” D. Percy leg. (BMNH)	19.2400, -155.8770	KY293772/KY294251
	other	5f	Kipuka Puaulu (Bird Park), Hawaii, USA, ex Metrosideros polymorpha var. polymorpha, 17 July 2002, “Hi415-02” D. Percy leg. (BMNH)	19.4373, -155.3032	−
	other	2m, 2f	Tree Planting Road, off Saddle Road, Hawaii, USA, ex Metrosideros polymorpha var. polymorpha, 18 July 2002, “418-02” D. Percy leg. (BMNH)	19.6834, -155.2951	−
	other	1f	Southern Belt road (34 mile marker to Hilo), Hawaii, USA, Metrosideros polymorpha var. incana, 19 July 2002, “420-02” D. Percy leg. (BMNH)	19.2950, -155.4200	−
	other	3m, 3f	1868 lava flow, near Kahuku Ranch, SW Hawaii, USA, ex Metrosideros polymorpha var. incana, 9 July 2002, “421-02” D. Percy leg. (BMNH)	19.0600, -155.6950	−
	other	1f	Olaa Forest, Hawaii, USA, ex Metrosideros polymorpha, 27 July 2002, “434-02” D. Percy leg. (BMNH)	19.4496, -155.2041	−
	other	2f	Kipuka Alani, Hawaii, USA, on Cheirodendron trigynum, 20 August 2003, “Hi32-03” D. Percy leg. (BMNH)	19.4403, -155.3078	−
	other	1m	Kipuka Ki, Hawaii, USA, on Metrosideros polymorpha var. incana, 20 August 2003, “Hi33-03” D. Percy leg. (BMNH)	19.4433, -155.3174	−
	other	1m, 1f	Kipuka Puaulu (Bird Park), Hawaii, USA, ex Metrosideros polymorpha var. incana, 21 August 2003, “Hi34-03” D. Percy leg. (BMNH)	19.4372, -155.3031	−
	other	1m	Olaa Forest, Hawaii, USA, ex Metrosideros polymorpha var. glaberrima, 24 August 2003, “Hi43-03” D. Percy leg. (BMNH)	19.4603, -155.2467	−
	other	1f	Tree Planting Road, off Saddle Road, Hawaii, USA, ex Metrosideros polymorpha var. polymorpha, 25 August 2003, “Hi46-03” D. Percy leg. (BMNH)	19.6833, -155.2950	−
	other	2m, 2f	Kehena, 3200ft, Kohala, Hawaii, USA, on Melicope, 12 August 2010, “JG2” J. Giffin leg. (BMNH)	−	−
	other	1m, 1f	Puu Oo Trail, off Saddle Road, Hawaii, USA, ex Metrosideros polymorpha, 31 May 2011, “Hi03-11” D. Percy leg. (BMNH)	19.6732, -155.3889	KY293773/KY294252
	other	2m, 1f	Puu O Umi NAR, Kohala, Hawaii, USA, ex Metrosideros polymorpha var. glaberrima, 2 June 2011, “Hi06-11” D. Percy leg. (BMNH)	20.0701, -155.7240	KY293774/KY294253
	other	1m, 2f	Waiakamali Gulch, Kohala, Hawaii, USA, ex Metrosideros polymorpha var. incana, 2 June 2011, “Hi08-11” D. Percy leg. (BMNH)	20.0660, -155.7260	KY293775/KY294254
hawaiiensis	other	1m	RIG Site (E. Stacy), off Saddle Road, Hawaii, USA, ex Metrosideros polymorpha var. incana and var. glaberrima, 3 June 2011, “Hi10-11” D. Percy leg. (BMNH)	19.6938, -155.2573	KY293776/KY294255
	other	1m	Kau, Hawaii, USA, ex Metrosideros polymorpha, 12 July 2013, “Hi05-13” D. Percy leg. (BMNH)	19.1883, -155.5850	−
	other	1f	Gulch below Puu O Umi, Kohala, Hawaii, USA, ex Metrosideros polymorpha, 17 July 2013, “Hi35-13” D. Percy leg. (BMNH)	20.0630, -155.7189	−
	other	1f	Olaa Forest (small unit off Wright Rd), Hawaii, USA, ex Metrosideros polymorpha var. incana and var. glaberrima, 18 July 13, “Hi37-13” D. Percy leg. (BMNH)	19.4620, -155.2480	−
	other	2f	Upper Hamakua Ditch, Kohala, Hawaii, USA, ex Metrosideros polymorpha, 5 March 2014, “Hi11-14” D. Percy leg. (BMNH)	20.0852, -155.6791	KY293778/KY294257
	other	4m, 3f	same data as previous except: “Hi13-14” D. Percy leg. (BMNH)	20.0797, -155.6701	KY293779-80/KY294258-59
	other	1m, 7f	Kona Hema, Hawaii, USA, ex Metrosideros polymorpha, 7 March 2014, “Hi23-14” D. Percy leg. (BMNH)	19.2092, -155.7771	KY293781-84/KY294260-63
	other	1m	same data as previous except: “Hi25-14” D. Percy leg. (BMNH)	19.2277, -155.8041	−
	other	3f	same data as previous except: “Hi26-14” D. Percy leg. (BMNH)	19.2226, -155.8308	KY293785-87/KY294264-66
	other	2m, 1f	same data as previous except: 9 March 2014, “Hi32-14” D. Percy leg. (BMNH)	19.2019, -155.7810	−
	other	2m, 4f	same data as previous except: “Hi33-14” D. Percy leg. (BMNH)	19.1774, -155.8208	KY293788-91/KY294267-70
	other	2m, 2f	same data as previous except: “Hi34-14” D. Percy leg. (BMNH)	19.1960, -155.8037	KY293792-93/KY294271-72
	other	1m, 1f	Wright Rd (common garden), Volcano, Hawaii, USA, ex Metrosideros polymorpha, 11 March 2014, “Hi35-14” D. Percy leg. (BMNH)	19.4756, -155.2602	−
pele	holotype	m	Kipuka Puaulu (Bird Park), Hawaii, USA, ex Metrosideros polymorpha var. incana, 21 August 2003, “Hi34-03” D. Percy leg. (BMNH)	19.4372, -155.3031	−
	paratypes	9m, 4f	same data as holotype		−
	other	2m, 3f	Southern Belt road (93 mile marker to Hilo), Hawaii, USA, Metrosideros polymorpha var. polymorpha, 29 May 2002, “387-02” D. Percy leg. (BMNH)	19.2680, -155.8750	−
pele	other	3m, 5f, 35i	Southern Belt road (91 mile marker to Hilo), Hawaii, USA, Metrosideros polymorpha var. polymorpha, 29 May 2002, “388-02” D. Percy leg. (BMNH)	19.2400, -155.8770	KR108099-100/
	other	1m, 3f	Kipuka Puaulu (Bird Park), Hawaii, USA, ex Metrosideros polymorpha var. polymorpha, 30 May 2002, “389-02” D. Percy leg. (BMNH)	19.4373, -155.3032	−
	other	6m, 12f	Southern Belt road (near entrance to HVNP), Hawaii, USA, ex Metrosideros polymorpha var. incana, 19 July 2002, “419-02” D. Percy leg. (BMNH)	19.3550, -155.8100	−
	other	2m	Southern Belt road (34 mile marker to Hilo), Hawaii, USA, Metrosideros polymorpha var. incana, 19 July 2002, “420-02” D. Percy leg. (BMNH)	19.2950, -155.4200	−
	other	3m, 3f	1868 lava flow, near Kahuku Ranch, SW Hawaii, USA, ex Metrosideros polymorpha var. incana, 9 July 2002, “421-02” D. Percy leg. (BMNH)	19.0600, -155.6950	−
	other	1m, 1f	Olaa Forest, Hawaii, USA, ex Metrosideros polymorpha, 27 July 2002, “434-02” D. Percy leg. (BMNH)	19.4496, -155.2041	−
	other	2m, 2f	Kilauea Crater area, HVNP, Hawaii, USA, ex Metrosideros polymorpha var. incana, 19 August 2003, “Hi31-03” D. Percy leg. (BMNH)	19.4247, -155.2928	−
	other	3f	Kipuka Alani, Hawaii, USA, on Cheirodendron trigynum, 20 August 2003, “Hi32-03” D. Percy leg. (BMNH)	19.4403, -155.3078	−
	other	2m, 2f	Kipuka Puaulu (Bird Park), Hawaii, USA, ex Metrosideros polymorpha var. polymorpha, 21 August 2003, “Hi35-03” D. Percy leg. (BMNH)	19.4372, -155.3031	−
	other	5m, 2f	Olaa Forest, Hawaii, USA, ex Metrosideros polymorpha var. glaberrima, 21 August 2003, “Hi36-03” D. Percy leg. (BMNH)	19.4500, -155.2042	−
	other	1f	same data as previous except: 22 August 2003, “Hi37-03” D. Percy leg. (BMNH)	19.4500, -155.2042	−
	other	5m, 2f	Manuka NAR, Hawaii, USA, ex Metrosideros polymorpha var. incana, 23 August 2003, “Hi38/39-03” D. Percy leg. (BMNH)	19.1097, -155.8256	−
	other	1m, 2f	Kipahoehoe NAR, Hawaii, USA, ex Metrosideros polymorpha var. incana, 23 August 2003, “Hi40-03” D. Percy leg. (BMNH)	19.2669, -155.8750	−
	other	2m, 1f	same data as previous except: “Hi42-03” D. Percy leg. (BMNH)	19.2467, -155.8778	−
	other	10m, 10f	Olaa Forest, Hawaii, USA, ex Metrosideros polymorpha var. glaberrima, 24 August 2003, “Hi43-03” D. Percy leg. (BMNH)	19.4603, -155.2467	KR108067/KR108113
pele	other	5m, 5f	Tree Planting Road, off Saddle Road, Hawaii, USA, ex Metrosideros polymorpha var. polymorpha, 25 August 2003, “Hi46-03” D. Percy leg. (BMNH)	19.6833, -155.2950	−
	other	2f	Kehena, 2800ft, Kohala, Hawaii, USA, on Antidesma, 23 September 2010, “JG9” J. Giffin leg. (BMNH)	−	−
	other	3m	Puu Oo Trail, off Saddle Road, Hawaii, USA, ex Metrosideros polymorpha, 31 May 2011, “Hi03-11” D. Percy leg. (BMNH)	19.6732, -155.3889	KR108084/KR108130
	other	1f	East of Waimea (off Hwy 19), Kohala, Hawaii, USA, ex Metrosideros polymorpha, 1 June 2011, “Hi04-11” D. Percy leg. (BMNH)	20.0621, -155.5352	KR108063/KR108109
	other	8m, 3f	Waiakamali Gulch, Kohala, Hawaii, USA, on Planchonella, 2 June 2011, “Hi05-11” D. Percy leg. (BMNH)	20.0631, -155.7285	−
	other	3m, 5f	Puu O Umi NAR, Kohala, Hawaii, USA, ex Metrosideros polymorpha var. glaberrima, 2 June 2011, “Hi06-11” D. Percy leg. (BMNH)	20.0701, -155.7240	KR108078/KR108124
	other	1m	same data as previous except: ex Metrosideros polymorpha var. polymorpha, “Hi07-11” D. Percy leg. (BMNH)	20.0701, -155.7240	−
	other	17m, 11f, 20i	Waiakamali Gulch, Kohala, Hawaii, USA, ex Metrosideros polymorpha var. incana, 2 June 2011, “Hi08-11” D. Percy leg. (BMNH)	20.0660, -155.7260	KR108079-82/KR108125-28
	other	8m, 4f	Tree Planting Road, off Saddle Road, Hawaii, USA, ex Metrosideros polymorpha, 3 June 2011, “Hi09-11” D. Percy leg. (BMNH)	19.6840, -155.2927	KR108074, KR108085/KR108120, KR108131
	other	3m, 2f	RIG Site (E. Stacy), off Saddle Road, Hawaii, USA, ex Metrosideros polymorpha var. incana and var. glaberrima, 3 June 2011, “Hi10-11” D. Percy leg. (BMNH)	19.6938, -155.2573	KR108075-76/KR108121-22
	other	19m, 1f	Hamakua Coast (21 mile marker), Hawaii, USA, ex Metrosideros polymorpha, 3 June 2011, “Hi11-11” D. Percy leg. (BMNH)	19.9542, -155.1903	KR108064-66, KR108087/KR108110-12, KR108133
	other	3m	Laupahoehoe, Hawaii, USA, Metrosideros polymorpha, 13 July 2013, “Hi07-13” D. Percy leg. (BMNH)	19.9301, -155.2890	KR108061-62/KR108107-08
	other	1m, 1i	Tree Planting Road (1850-1880 flows), off Saddle Road, Hawaii, USA, ex Metrosideros polymorpha var. incana and var. glaberrima, 14 July 2013, “Hi17-13” D. Percy leg. (BMNH)	19.6642, -155.2783	−
	other	7m, 2f	Puu Lae Lae, Kohala, Hawaii, USA, ex Metrosideros polymorpha, 16 July 2013, “Hi22-13” D. Percy leg. (BMNH)	20.0448, -155.6870	KR108086/KR108132
	other	1m	Kohala Forest Preserve, Hawaii, USA, on Melicope, 16 July 2013, “Hi24-13”	20.0496, -155.6875	−
pele	other	3i	Gulch below Puu O Umi, Kohala, Hawaii, USA, ex Metrosideros polymorpha, 17 July 2013, “Hi35-13” D. Percy leg. (BMNH)	20.0630, -155.7189	KR108083/KR108129
	other	2m, 2f	Olaa Forest (small unit off Wright Rd), Hawaii, USA, ex Metrosideros polymorpha var. incana and var. glaberrima, 18 July 13, “Hi37-13” D. Percy leg. (BMNH)	19.4620, -155.2480	KR108072, KR108077/KR108118, KR108123
	other	1m	Alili Spring trail, Kau, Hawaii, USA, ex Metrosideros polymorpha, 19 July 2013, “Hi47-13” D. Percy leg. (BMNH)	19.2333, -155.5208	KR108073/KR108119
	other	8m, 9f, 3i	Humuula trail, Hawaii, USA, ex Metrosideros polymorpha var. glaberrima, 20 July 2013, “Hi59-13” D. Percy leg. (BMNH)	19.9652, -155.3028	KR108069-70/KR108114-17
	other	6m, 4f	Kipuka off Saddle Road, Hawaii, USA, ex Metrosideros polymorpha var. polymorpha, 4 March 2014, “Hi03-14” D. Percy leg. (BMNH)	19.6734, -155.3396	KY294010-13/KY294487-89
	other	20m, 20f	Upper Hamakua Ditch, Kohala, Hawaii, USA, ex Metrosideros polymorpha var. glaberrima, 5 March 2014, “Hi08-14” D. Percy leg. (BMNH)	20.0693, -155.6697	KY294014-19/KY294490-95
	other	2m	same data as previous except: “Hi09-14” D. Percy leg. (BMNH)	20.0714, -155.6711	KY294020-21/KY294496-97
	other	2m	same data as previous except: ex Metrosideros polymorpha, “Hi11-14” D. Percy leg. (BMNH)	20.0852, -155.6791	KY294022-23/KY294498-99
	other	4m, 5f	same data as previous except: “Hi12-14” D. Percy leg. (BMNH)	20.0841, -155.6752	KY294024-25/KY294500-01
	other	1m, 2f	same data as previous except: “Hi13-14” D. Percy leg. (BMNH)	20.0797, -155.6701	KY294026-27/KY294502-03
	other	1f	Puu Makaala, Hawaii, USA, ex Metrosideros polymorpha, 6 March 2014, “Hi15-14” D. Percy leg. (BMNH)	19.5495, -155.2312	KY294028/KY294504
	other	3m, 5f	same data as previous except: “Hi17-14” D. Percy leg. (BMNH)	19.5522, -155.2310	KY294029-32/KY294505-08
	other	2m, 1f	Kona Hema, Hawaii, USA, ex Metrosideros polymorpha, 7 March 2014, “Hi21-14” D. Percy leg. (BMNH)	19.2047, -155.8123	KY294033-35/KY294509-11
	other	5m, 3f	same data as previous except: “Hi22-14” D. Percy leg. (BMNH)	19.2054, -155.7880	−
	other	10m, 10f	same data as previous except: “Hi23-14” D. Percy leg. (BMNH)	19.2092, -155.7771	KY294036-41/KY294512-17
pele	other	10m, 10f	same data as previous except: “Hi24-14” D. Percy leg. (BMNH)	19.2301, -155.7818	KY294042-48/KY294518-24
	other	7m, 7f	same data as previous except: “Hi25-14” D. Percy leg. (BMNH)	19.2277, -155.8041	KY294049-53/KY294525-29
	other	9m, 4f	same data as previous except: “Hi26-14” D. Percy leg. (BMNH)	19.2226, -155.8308	KY294054-57/KY294530-33
	other	2m, 1f	same data as previous except: “Hi27-14” D. Percy leg. (BMNH)	19.2153, -155.8294	−
	other	9m, 3f	same data as previous except: 9 March 2014, “Hi31-14” D. Percy leg. (BMNH)	19.2158, -155.7767	−
	other	3m, 1f	same data as previous except: “Hi32-14” D. Percy leg. (BMNH)	19.2019, -155.7810	−
	other	10m, 10f	same data as previous except: “Hi33-14” D. Percy leg. (BMNH)	19.1774, -155.8208	KY294075-81/KY294551-57
	other	5m, 3f	same data as previous except: “Hi34-14” D. Percy leg. (BMNH)	19.1960, -155.8037	−
	other	10m, 10f	Honuaula FR, Makaula Ooma Tract, Hualalai, Hawaii, USA, ex Metrosideros polymorpha, 8 March 2014, “Hi28-14” D. Percy leg. (BMNH)	19.7180, -155.9487	KY294058-63/KY294534-39
	other	4f	same data as previous except: “Hi29-14” D. Percy leg. (BMNH)	19.7197, -155.9459	KY294064-66/KY294540-42
	other	9m, 3f	Koloko Drive, Honuaula, Hualalai, Hawaii, USA, ex Metrosideros polymorpha, 8 March 2014, “Hi30-14” D. Percy leg. (BMNH)	19.7079, -155.9243	KY294067-74/KY294543-50
	other	1m, 1f	Wright Rd (common garden), Volcano, Hawaii, USA, ex Metrosideros polymorpha, 11 March 2014, “Hi35-14” D. Percy leg. (BMNH)	19.4756, -155.2602	−
	other	1f	Tree Planting Rd, off Saddle Rd, Hawaii, USA, ex Metrosideros polymorpha var. polymorpha, 11 March 2014, “Hi37-14” D. Percy leg. (BMNH)	19.6828, -155.2946	KY294082/KY294558
pyramidalis	holotype	m	Kipuka Puaulu (Bird Park), Hawaii, USA, ex Metrosideros polymorpha var. incana, 21 August 2003, “Hi34-03” D. Percy leg. (BMNH)	19.4372, -155.3031	−
	paratypes	3m, 2f	same data as holotype		−
	other	5m, 5f, 30i	Southern Belt road (95 mile marker to Hilo), Hawaii, USA, Metrosideros polymorpha var. polymorpha, 29 May 2002, “386-02” D. Percy leg. (BMNH)	19.2680, -155.8750	KR108102-03/KY294580-81
pyramidalis	other	1m, 1f, 45i	Southern Belt road (91 mile marker to Hilo), Hawaii, USA, Metrosideros polymorpha var. polymorpha, 29 May 2002, “388-02” D. Percy leg. (BMNH)	19.2400, -155.8770	KR108101/KY294582
	other	78i	Kipuka Puaulu (Bird Park), Hawaii, USA, ex Metrosideros polymorpha var. polymorpha, 17 July 2002, “Hi415-02” D. Percy leg. (BMNH)	19.4373, -155.3032	KR108092/KR108138
	other	6m, 6f	Southern Belt road (near entrance to HVNP), Hawaii, USA, ex Metrosideros polymorpha var. incana, 19 July 2002, “419-02” D. Percy leg. (BMNH)	19.3550, -155.8100	−
	other	1m	Southern Belt road (34 mile marker to Hilo), Hawaii, USA, Metrosideros polymorpha var. incana, 19 July 2002, “420-02” D. Percy leg. (BMNH)	19.2950, -155.4200	−
	other	2m, 2f	1868 lava flow, near Kahuku Ranch, SW Hawaii, USA, ex Metrosideros polymorpha var. incana, 9 July 2002, “421-02” D. Percy leg. (BMNH)	19.0600, -155.6950	−
	other	1m, 1f	Olaa Forest, Hawaii, USA, ex Metrosideros polymorpha, 27 July 2002, “434-02” D. Percy leg. (BMNH)	19.4496, -155.2041	−
	other	1m, 1f	Mountain View, Hawaii, USA, Metrosideros polymorpha var. polymorpha, 27 July 2002, “436-02”	19.5300, -155.1020	−
	other	1f	Puuwaawaa, Hawaii, USA, ex Metrosideros polymorpha, 29 July 2002, “440-02”	19.7840, -155.8330	−
	other	38i	Kipuka Puaulu (Bird Park), Hawaii, USA, ex Metrosideros polymorpha var. polymorpha, 27 July 2002, “Hi441-02” D. Percy leg. (BMNH)	19.4373, -155.3032	KR108104-05/KY294583
	other	30i	same data as previous except: “Hi442-02” D. Percy leg. (BMNH)	19.4373, -155.3032	KR108106/KY294584
	other	2m, 2f, 10i	Kilauea Crater area, HVNP, Hawaii, USA, ex Metrosideros polymorpha var. incana, 19 August 2003, “Hi31-03” D. Percy leg. (BMNH)	19.4247, -155.2928	−
	other	2m, 1f	Kipuka Puaulu (Bird Park), Hawaii, USA, ex Metrosideros polymorpha var. polymorpha, 21 August 2003, “Hi35-03” D. Percy leg. (BMNH)	19.4372, -155.3031	−
	other	5m, 2f	Olaa Forest, Hawaii, USA, ex Metrosideros polymorpha var. glaberrima, 21 August 2003, “Hi36-03” D. Percy leg. (BMNH)	19.4500, -155.2042	−
	other	1m, 1f	Manuka NAR, Hawaii, USA, ex Metrosideros polymorpha var. incana, 23 August 2003, “Hi39-03” D. Percy leg. (BMNH)	19.1097, -155.8256	−
	other	1m, 1f	Kipahoehoe NAR, Hawaii, USA, ex Metrosideros polymorpha var. incana, 23 August 2003, “Hi40-03” D. Percy leg. (BMNH)	19.2669, -155.8750	−
pyramidalis	other	10m, 10f	Olaa Forest, Hawaii, USA, ex Metrosideros polymorpha var. glaberrima, 24 August 2003, “Hi43-03” D. Percy leg. (BMNH)	19.4603, -155.2467	−
	other	1f	Kehena, 3150ft, Kohala, Hawaii, USA, on Tetraplasandra, 5 August 2010, “JG8” J. Giffin leg. (BMNH)	−	−
	other	1i	Kilauea Iki Crater, Hawaii, USA, ex Metrosideros polymorpha, 30 May 2011, “Hi02-11” D. Percy leg. (BMNH)	19.4130, -155.2460	KR108097/KR108143
	other	2f, 1i	East of Waimea (off Hwy 19), Kohala, Hawaii, USA, ex Metrosideros polymorpha, 1 June 2011, “Hi04-11” D. Percy leg. (BMNH)	20.0621, -155.5352	KR108090-91, KR108098/KR108136-37, KR108144
	other	2i	Keauohana FR, Hawaii, USA, ex Metrosideros polymorpha var. glaberrima, 1 March 2012, “ES3/4-12” E. Stacy leg. (BMNH)	19.4211, -154.9560	KR108095-96/KR108141-42
	other	6i	KMC military camp, HVNP, Hawaii, USA, Metrosideros polymorpha var. incana, 12 July, 2013, “Hi03-13” D. Percy leg. (BMNH)	19.4341, -155.2739	KR108093/KR108139
	other	5i	Kohala Forest Preserve, Hawaii, USA, ex Metrosideros polymorpha var. glaberrima, 16 July 2013, “Hi26-13” D. Percy leg. (BMNH)	20.0538, -155.6828	KR108088-89/KR108134-35
	other	1f	Humuula trail, Hawaii, USA, ex Metrosideros polymorpha var. glaberrima, 20 July 2013, “Hi59-13” D. Percy leg. (BMNH)	19.9652, -155.3028	KR108094/KR108140
	other	1f	Kona Hema, Hawaii, USA, ex Metrosideros polymorpha, 7 March 2014, “Hi22-14” D. Percy leg. (BMNH)	19.2054, -155.7880	KY294102/KY294585
	other	14m, 13f	same data as previous except: “Hi26-14” D. Percy leg. (BMNH)	19.2226, -155.8308	KY294103-06/KY294586-89
	other	4m, 16f	same data as previous except: “Hi27-14” D. Percy leg. (BMNH)	19.2153, -155.8294	−
	other	10m, 10f	same data as previous except: 9 March 2014, “Hi33-14” D. Percy leg. (BMNH)	19.1774, -155.8208	KY294120-24/KY294603-07
	other	2m, 1f	same data as previous except: “Hi34-14” D. Percy leg. (BMNH)	19.1960, -155.8037	−
	other	20m, 20f	Honuaula FR, Makaula Ooma Tract, Hualalai, Hawaii, USA, ex Metrosideros polymorpha, 8 March 2014, “Hi28-14” D. Percy leg. (BMNH)	19.7180, -155.9487	KY294107-12/KY294590-95
	other	17m, 10f	same data as previous except: “Hi29-14” D. Percy leg. (BMNH)	19.7197, -155.9459	KY294113-19/KY294596-602
	other	1m, 1f	Wright Rd (common garden), Volcano, Hawaii, USA, ex Metrosideros polymorpha, 11 March 2014, “Hi35-14” D. Percy leg. (BMNH)	19.4756, -155.2602	−

**Table 3.** Outgroup material examined with GenBank numbers for cytochrome oxidase one (COI) and cytochrome B (cytB).

Family Species	n	Collection information	lat, long	COI	cytB
**Carsidaridae** Mesohomotoma hibisci (Froggatt, 1901)	1m	Motu Mautaro, Tubuai, Austral Islands, French Polynesia, ex Hibiscus tiliaceus, 15 November 2003, “FP26-03” D. Percy leg. (BMNH)	-23.3421S, -149.4151W	KY294172	KY294656
Mesohomotoma hibisci	30m, 30f	north of airport, Moorea, Society Islands, French Polynesia, ex Hibiscus tiliaceus, 7 June 2002, “DMP-401A-02” D. Percy leg. (BMNH)	-17.4871S, -149.7708W	KY294174	KY294658
Mesohomotoma hibisci	1f	below Ati Ati Peak, Moorea, Society Islands, French Polynesia, ex Hibiscus tiliaceus, 15 March 2009, “FP32B-09” D. Percy leg. (BMNH)	-17.5282S, -149.8584W	KY294173	KY294657
Mesohomotoma hibisci	4m, 2f	coastal road, western Raiatea, Society Islands, French Polynesia, ex Hibiscus tiliaceus, 8 March 2009, “FP18-09” D. Percy leg. (BMNH)	-16.76312S, -151.49031W	KY294171	KY294655
Mesohomotoma hibisci	5m, 5f	Pouanlotch River, ca. 20km N of Voh, New Caledonia, ex Hibiscus tiliaceus, “DMP-464A-02” D. Percy leg. (BMNH)	-20°51’29”S, 164°36’44”E	KY294170	KY294654
Mesohomotoma hibisci	2m	Airport, Norfolk Island, Australia, ex Hibiscus tiliaceus, 21 December 2012, “LAM5675” L. Mound leg. (BMNH)	-29.042S, 167.94E	KY294175	KY294659
Mesohomotoma hibisci	1m, 2f	Berlayer Creek, Labrador Nature Reserve, Singapore, ex Hibiscus tiliaceus, 9 November 2012, “SING06A-12” D. Percy leg. (BMNH)	1.267N, 103.803E	KY294176	KY294660
**Triozidae** Anomocephala unica Tuthill, 1942	1m	Rapa Island, French Polynesia, ex Metrosideros, 17 December 2004, “EC-Aunica-04” E. Claridge leg. (BMNH)	−	KY293698	KY294177
Bactericera cockerelli (Šulc, 1909)	−	Coahuila, Mexico [DNAs sample supplied by Trumble Lab, University of California, Riverside]	−	KY293699	KY294178
Bactericera cockerelli	−	Orange Co., California, USA [DNAs sample supplied by Trumble Lab, University of California, Riverside]	−	KY011201	KY011296
Baeoalitriozus diospyri (Ashmead, 1881)	5m, 5f	Bladen Lakes State Forest, North Carolina, USA, ex Diospyros virginiana, 2 June 2005, “NC02_Tdiospyri” D. Percy leg. (BMNH)	−	KY293700	KY294179
Baeoalitriozus diospyri	1f	Louisiana, USA, ex Diospyros virginiana, 8 August 2004, “Tdios-Louisiana” D. Percy leg. (BMNH)	−	KY293701	KY294180
Hevaheva maculata Caldwell, 1940	9m, 11f	Nualolo Trail, Kokee State Park, Kauai, USA, ex Melicope anisata (leaf edge curl), 25 May 2002, “HI369-02” D. Percy leg. (BMNH)	22.13N, -159.67W	KY293702	KY294181
Hevaheva minuta Crawford, 1925	12m, 8f	Nualolo Trail, Kokee State Park, Kauai, USA, ex Melicope barbigera, 25 May 2002, “HI366B-02” D. Percy leg. (BMNH)	22.13N, -159.67W	KY293703	KY294182
Hevaheva perkinsi Kirkaldy, 1902	2m, 2f	Mnt Kaala, Waianae Mnts, Oahu, USA, ex Melicope christophersenii, 4 July 2014, “Hi58-14” D. Percy leg. (BMNH)	21.5061N, -158.1457W	KY293704	KY294183
Hevaheva silvestris Kirkaldy, 1908	2m, 2f, 1i	Mnt Kaala, Waianae Mnts, Oahu, USA, ex Melicope christophersenii, 4 July 2014, “Hi58-14” D. Percy leg. (BMNH)	21.5061N, -158.1457W	KY293705	KY294184
Hevaheva sp.	17m, 14f	TNC Honouliuli Preserve, Waianae Mnts, Oahu, USA, ex Melicope sp., 22 May 2002, “HI363-02” D. Percy leg. (BMNH)	21.436N, -158.092W	KY293706	KY294185
Kuwayama minutura (Caldwell, 1940)	15m, 22f	Pahole NAR, Waianae Mnts, Oahu, USA, ex Pisonia sandwicensis, 14 August 2003, “Hi5Bmin-03” D. Percy leg. (BMNH)	21°32’14”N, -158°11’32”W	KY293707	KY294186
Kuwayama pisonia Caldwell, 1940	10m, 5f	Pahole NAR, Waianae Mnts, Oahu, USA, ex Pisonia sandwicensis, 14 August 2003, “Hi5Bpis-03” D. Percy leg. (BMNH)	21°32’14”N, -158°11’32”W	KY293708	KY294187
Leptynoptera sulfurea Crawford, 1919	1f	Motu Motihia, Tubuai, Austral Islands, French Polynesia, 15 November 2003, “FP24-03” D. Percy leg. (BMNH)	-23.3702S, -149.39604W	KY293711	KY294190
Leptynoptera sulfurea	24m, 18f, 9i	Forêt Sèche, Parc Forestier, Noumea, New Caledonia, ex Calophyllum caledonicum, 20 August 2002, “DMP-451A-02” D. Percy leg. (BMNH)	-22.2590S, 166.4590E	KY293709	KY294188
Leptynoptera sulfurea	2m, 1f	Singapore Botanical Garden, Singapore, ex Calophyllum inophyllum, 10 November 2012, “SING08-12” D. Percy leg. (BMNH)	1.3086N, 103.8181E	KY293712	KY294191
Leptynoptera sulfurea	7m, 25f	National Cheng Kung University campus, Tainan, Taiwan, ex Calophyllum inophyllum, 28 January 2010, “DPTAI-73-10” D. Percy leg. (BMNH)	22.9973N, 120.2202E	KY293710	KY294189
Megatrioza kauaiensis Uchida & Beardsley, 1988	11m, 6f, 7i	Kalalau Valley (close to 2nd lookout), Kokee State Park, Kauai, USA, ex Pritchardia minor, 26 May 2002, “DMP-378-02” D. Percy leg. (BMNH)	22.149N, -159.632W	KY293713	KY294192
Megatrioza zanthoxyli Uchida & Beardsley, 1992	1m, 2f, 6i	Pohakuloa Training Area, Hawaii, USA, ex Zanthoxylum hawaiiense, 25 August 2003, “Hi45-03” D. Percy leg. (BMNH)	19°45’03”N, -155°37’57”W	KY293714	KY294193
Powellia vitreoradiata Maskell, 1879	2m, 2f	Burnt Pine, Norfolk Island, Australia, ex Pittosporum undulatum, 22 December 2012, “LAM5681” L. Mound leg. (BMNH)	-29.033N, 167.95W	KY294138	KY294622
Schedotrioza apicobystra Taylor, 1990	3m, 3f	Adelaide Hills, South Australia, Australia, reared from galls ex Eucalytpus cosmophylla, 10 September 2001, “S35apic-01” G. Taylor leg. (BMNH)	−	KY294139	KY294623
Schedotrioza marginata Taylor, 1987	3m, 3f	Adelaide Hills, South Australia, Australia, reared from galls ex Eucalytpus obliqua, 10 September 2001, “S32marg-01” G. Taylor leg. (BMNH)	−	KY294140	KY294624
Schedotrioza multitudinea (Maskell, 1898)	3m, 3f	Adelaide Hills, South Australia, Australia, reared from galls ex Eucalytpus obliqua, 10 September 2001, “S29mult-01” G. Taylor leg. (BMNH)	−	KY294141	KY294625
Swezeyana elongagena Caldwell, 1940	1m, 2f	South Mohiakea, Waianae Mnts, Oahu, USA, ex Planchonella sandwicensis, 29 January 2014, “KM16-14” K. Magnacca leg. (BMNH)	21.4821N, -158.1247W	KY294142	KY294626
Swezeyana elongagena	1m, 2f	Mnt Kaala road (culvert 32), Waianae Mnts, Oahu, USA, ex Planchonella sandwicensis, 26 August 2003, “Hi57-03” D. Percy leg. (BMNH)	−	KY294143	KY294627
Swezeyana reticulata Caldwell, 1940	1m, 1f	Mokuleia Forest Reserve, Pahole NAR, Waianae Mnts, Oahu, USA, ex Planchonella sandwicensis, 6 July 2014, “Hi74-14” D. Percy leg. (BMNH)	21.5321N, -158.1786W	KY294144	KY294628
Swezeyana reticulata	1m, 1f	Puu Hapapa, Waianae Mnts, Oahu, USA, ex Planchonella sandwicensis, 17 May 2014, “KM15-14” K. Magnacca leg. (BMNH)	21.4666N, -158.1029W	KY294145	KY294629
Trioza adventicia Tuthill, 1952	20m, 20f	Auckland, New Zealand, ex Syzygium paniculatum, 11 March 2002, “NZtri1” Pariaconus Dale leg. (BMNH)	−	KY294146	KY294630
Trioza alipellucida Klyver, 1932	4m, 7f, 10i	Mt. Ootua, Hiva Oa, Marquesas, French Polynesia, ex Metrosideros collina, 16 June 2002, “FP411-02” D. Percy leg. (BMNH)	−	KY294147	KY294631
Trioza anceps Tuthill, 1944	3m, 3f	Dur-West Farm, Sumpango, Sacatepequez, Guatemala, ex Persea americana ‘Hass’, 14 March 2008, “GT-2007-41” M. Hoddle leg. (BMNH)	14°40.292N, -90°43.195W	KY294148	KY294632
Trioza anceps	1f	Las Cuevas, Chiquibul Forest Reserve, Cayo District, Belize, ex Persea americana, 11 June 2002, “JHM7670” J. Martin leg. (BMNH)	−	KY294149	KY294633
Trioza eugeniae Froggatt, 1901	1f	Santa Cruz, California, USA, ex Syzygium paniculatum, 2003, “DPTeug2” D. Percy leg. (BMNH)	−	KY294150	KY294634
Trioza eugeniae	1m, 7f	Santa Monica hills, Los Angeles, California, USA, 29 April 2006, “LA3A-06” D. Percy leg. (BMNH)	34.1072N, -118.4278W	KY294151	KY294635
Trioza eugeniae	3i	Queensland, Australia, ex Syzygium smithii, 2002, “LGC02-149” L. Cook leg. (BMNH)	−	KY294152	KY294636
Trioza eugeniae	1f	University of California, Berkeley, California, USA, ex Syzygium, 2003, “Teug1-03” D. Percy leg. (BMNH)	37.8730N, -122.2629W	KY294153	KY294637
Trioza kuwayamai Enderlein, 1914	5m, 3f	Kenting National Park, Pingtung, Taiwan, ex Planchonella obovata, 30 January 2010, “DPTAI-78A-10” D. Percy leg. (BMNH)	21.9501N, 120.8231E	KY294154	KY294638
Trioza magnoliae (Ashmead, 1881)	3m, 3f	Jonathon Dickson State Park, Florida, USA, ex Persea borbonia, 22 May 2005, “FL11-05” D. Percy leg. (BMNH)	−	KY294155	KY294639
Trioza malloticola (Crawford, 1928)	2m, 6i	Rafting Ground Reserve, Queensland, Australia, ex Mallotus philippensis (leaf tip galls), 1 April 2002, “TrioA” C. Burwell leg. (BMNH)	-27°31’17”S, 152°55’30”E	KY294156	KY294640
Trioza obunca Fang & Yang, 1986	7m, 17f	Pingtung, Taiwan, ex Syzygium buxifolium, 30 January 2010, “DPTAI-81A-10” D. Percy leg. (BMNH)	22.0603N, 120.8611E	KY294157	KY294641
Trioza outeiensis Yang, 1984		Pingtung, Taiwan, ex Syzygium buxifolium, 30 January 2010, “DPTAI-81B-10” D. Percy leg. (BMNH)	22.0603N, 120.8611E	KY294159	KY294643
Trioza pallida (Uichanco, 1919)	6i	Rafting Ground Reserve, Queensland, Australia, ex Mallotus philippensis (pit depressions on leaf underside), 1 April 2002, “TrioB”, C. Burwell leg. (BMNH)	-27°31’17”S, 152°55’30”E	KY294160	KY294644
Trioza percyae Taylor, 2013 (in Taylor et al. 2013)	7m, 13f	West of Murray Bridge, South Australia, Australia, ex Allocasuarina verticillata, 3 October 2001, “S41tri2-01” D. Percy leg. (BMNH)	−	KY294161	KY294645
Trioza remota Foerster, 1848	3m, 3f	Cawdor, Scotland, ex Quercus, 21 September 1998, “283-TremoSI”, D. Percy leg. (BMNH)	−	KY294163	KY294647
Trioza remota	1m, 1f	Bookham Common, Surrey, UK, on Juncus, 14 October 2012, “BOOK011-12” D. Percy leg. (BMNH)	51.2913N, -0.3825W	KY294162	KY294646
Trioza sp.	3m, 4f, 1i	Cradle Mnt, Tasmania, Australia, ex Banksia marginata, 4 January 2003, “Tas489-03” D. Percy leg. (BMNH)	−	KY294164	KY294648
Trioza tricornuta Taylor, 2013 (in Taylor et al. 2013)	2m, 5f	West of Murray Bridge, South Australia, Australia, ex Allocasuarina verticillata, 3 October 2001, “S41tri1-01” D. Percy leg. (BMNH)	−	KY294165	KY294649
Trioza urticae (Linné, 1758)	5m, 5f	Kew, London, UK, ex Urtica dioica, 10 June 2003, “TurticSI” D. Percy leg.	−	KY294167	KY294651
Trioza urticae	17m, 5f	Bookham Common, Surrey, UK, ex Urtica dioica, 18 July 2012 “BOOK01-12Turt3” D. Percy leg. (BMNH)	51.2913N, -0.3825W	KY294166	KY294650
Trioza vitiensis Kirkaldy, 1907	1f	Mouaputa cascade, Vaioro River, Moorea, Society Islands, French Polynesia, ex Syzygium malaccense, 9 November 2003, “FP2-03” D. Percy leg. (BMNH)	-17.5380S, -149.7975W	KY294168	KY294652
Trioza zimmermani Tuthill, 1942	1m, 9f	ridge between Tonarutu and Tavaetu, Tubuai, Austral Islands, French Polynesia, ex Metrosideros collina var. fruticosa, 11 November 2003, “FP10-03” D. Percy leg. (BMNH)	-23.3861S, -149.5078W	KY294169	KY294653
Pauropsylla triozoptera Crawford, 1913	22i	Pingtung, Taiwan, dissected from galls ex Ficus cf. ampelas, 30 January 2010, “TAI83-10” D. Percy leg. (BMNH)	22.0499N, 120.8576E	KT588303	KT588309

### Molecular analysis

The molecular analysis includes 537 individuals, 479 in Pariaconus and 58 in outgroup taxa. To confirm adult/immature/gall types and taxon group associations, DNA barcodes were sequenced from two mitochondrial gene regions, cytochrome oxidase I (COI), and cytochrome B (cytB). Protocols for DNA extraction, PCR, and sequencing follow those described in Percy (2003b), except for annealing temperature, 56°C for cytB versus 50°C for COI; PCR primers for COI and cytB respectively are given in Simon et al. (1994), and Timmermans et al. (2010). Distance neighbour-joining (NJ) analyses were performed using program PAUP* (Swofford 2003), and maximum likelihood (ML) with RAxML (v.8.2.4) on CIPRES (Miller et al. 2010, Stamatakis 2014). Genetic distances reported use uncorrected (p) distances estimated in PAUP* (Swofford 2003). The molecular phylogenetic analyses reported here are intended as confirmation of morphological species concepts, as well as to compare genetic variation with geographic and morphological variation. Further phylogenetic analyses will focus on assessing different rates of evolution in this group. Genomic DNA was extracted from whole specimens, adult or immature, and post-DNA extraction voucher specimens retained in ethanol or slide mounted. The DNA sequences are deposited in GenBank (Tables 2–3). Thirty-seven outgroup taxa in the family Triozidae were included in the phylogenetic analyses, with a focus on taxa putatively ancestral to the Hawaiian Metrosideros-feeders, including Metrosideros-feeders from French Polynesia and New Zealand, other Myrtaceae-feeders from the Pacific-Australasian region, taxa producing galls from continental regions on both sides of the Pacific, the type species of Trioza, and some of the other endemic genera in the Hawaiian Islands. To provide a root for Triozidae, Mesohomotoma hibisci (Froggatt, 1901) (Carsidaridae) was included.

To interpret the putative ancestral biology (i.e. galling versus non-galling) for Pariaconus, an ancestral character state analysis was performed using Mesquite (v.3.11) (Madison and Madison 2016) with both parsimony (unordered) and maximum likelihood (Mk1 model) reconstructions. A single three state character, states: free-living, open galling, closed galling, plus “unknown”, was traced onto the majority-rule consensus topology of the maximum likelihood analysis using a single representative for each Pariaconus species, and the two Trioza species (that are strongly supported as the nearest outgroups) either as monophyletic or paraphyletic.

### Morphological analysis

Ethanol preserved material was cleared in 10% potassium hydroxide followed by clove oil and slide mounted in Canada balsam as described in Hodkinson and White (1979). In some instances, 1^st^-2^nd^ instar immatures were mounted directly into Euparal from 95% ethanol without clearing. Post-DNA extraction voucher specimens were either placed directly into clove oil from 100% ethanol, or were first cleared in 10% potassium hydroxide. DNA voucher specimens not cleared before mounting often retain red pigmented ocular tissue after DNA extraction (visible in some Figures). Morphological terminology follows Hodkinson and White (1979), Hollis (1984), White and Hodkinson (1985), and Percy (2003a). Type and other material is deposited in the Natural History Museum, London, UK (BMNH), and in the Bishop Museum, Honolulu, USA (BPBM) (Tables 2–3).

As there is a large degree of size variation in some Pariaconus species (particularly ohialoha group), averages (av.) across all individuals measured are used in descriptions and keys with the measured range given in Tables 4–7. Male parameres are illustrated as outlines (without setation added) because the shape is in most cases sufficient for differentiation, where shape is similar between species, the setation is relatively uniform, and not as informative as other characters illustrated. Suppl. material 1 illustrates adult characters and measurements referred to in the text. Abbreviations used in the descriptions and Tables 4–7 are as follows (all measurements are recorded in mm): Adults: WL, fore wing length; WW, fore wing width; HW, head width; AL, antennal length; GP, genal process length; PB, distal proboscis segment length; WL:WW, ratio fore wing length:width; CUR, ratio fore wing cell cu_1_ width:height; MR, ratio fore wing cell m_2_ width:height; HW:VW, ratio head width:vertex width; VL:GP, ratio vertex length:genal process length; VW:VL, ratio vertex width:length; AL:HW ratio antennal length:head width; HW:HT ratio head width:hind tibia length. Adult male terminalia: MP, proctiger length; PL, paramere length; AEL, distal aedeagus segment length; PL:HW, ratio paramere length:head width; MP:PL, ratio proctiger length:paramere length; PL:AEL, ratio paramere length:distal aedeagus segment length; AEL:AELH, ratio distal aedeagus segment length:aedeagus apical head length; PL:SH, ratio paramere length:subgenital plate height. Adult female terminalia: FP, proctiger length; FSP, subgenital plate length; RL, anal ring length; OVH, ovipositor valvulae dorsalis height; EL, egg length; EW, egg width; FP:RL, ratio female proctiger length:anal ring length; FP:HW, ratio female proctiger length:head width; FP:SP: ratio female proctiger length:subgenital plate length; EL:EW, ratio egg length:egg width. Immatures: BL, body length; BW, body width; WPL, fore wing pad length; CPL, caudal plate length; CPW, caudal plate width; RW, circumanal ring width; HW, head width; AL, antennal length; BL:BW ratio body length:width; HW:AL ratio head width:antennal length; CPW:RW ratio caudal plate width:circumanal ring width.

Data resources. The collections and specimen data underpinning the analyses reported in this paper are deposited in the Natural History Museum Data Portal as Diana Percy (2017) Dataset: Metrosideros-feeding psyllids of the Hawaiian Islands. http://dx.doi.org/10.5519/0097165

**Table 4.** Adult Pariaconus measurements (mm).

Group	Species	n	WL	WW	HW	AL	GP	PB	MP	PL	AEL	FP	FSP	RL	OVH	EL
**bicoloratus**	nigricapitus	1m, 3f	1.7–2.30	0.7–0.97	0.47–0.52	0.56–0.80	0.03–0.05	0.06–0.08	0.19	0.16	0.16	0.30–0.38	0.20–0.28	0.14–0.20	0.06–0.08	0.26
	hina	5m, 9f	1.61–2.21	0.67–0.91	0.46–0.55	0.62–0.73	0.03–0.05	0.05–0.06	0.14	0.12–0.13	0.12	0.28–0.34	0.18–0.26	0.13–0.15	0.06–0.07	0.26–0.28
	wyvernus	5m, 8f	1.70–2.38	0.76–1.12	0.48–0.55	0.61–0.82	0.03–0.06	0.06–0.08	0.17–0.20	0.16–0.18	0.16–0.20	0.34–0.42	0.23–0.30	0.15–0.22	0.07–0.09	0.30–0.34
	nigrilineatus	0m, 3f	1.85–1.94	0.77–0.82	0.48–0.52	0.58–0.61	0.03–0.05	0.10–0.11	−	−	−	0.42–0.45	0.30–0.33	0.11–0.13	0.08–0.09	0.27–0.28
	kapo	0m, 1f	2.67	1.12	0.58	0.94	0.08	0.11	−	−	−	0.62	0.54	0.18	0.10	−
	proboscideus	5m, 3f	1.61–2.11	0.58–0.89	0.43–0.49	0.64–0.76	0.04–0.07	0.12–0.15	0.18–0.21	0.17–0.19	0.16–0.18	0.55–0.58	0.37–0.50	0.18–0.20	0.08–0.10	0.28
	poliahu	3m, 4f	1.67–2.24	0.68–0.95	0.47–0.52	0.61–0.73	0.03–0.05	0.05–0.06	0.16–0.17	0.13–0.14	0.17–0.19	0.34–0.36	0.23–0.26	0.17–0.18	0.06–0.08	0.26
	lona	1m, 3f	1.74–2.03	0.68–0.83	0.47–0.52	0.71–0.73	0.05–0.08	0.11–0.12	0.19	0.17	0.18	0.51–0.55	0.40–0.42	0.15–0.16	0.09	0.22
	liliha	1m, 3f	1.89–2.51	0.71–0.94	0.52–0.55	0.74–0.76	0.03–0.06	0.05–0.08	0.16	0.14	0.13	0.36–0.37	0.25	0.18–0.19	0.11	0.30
	gracilis	12m, 14f	1.24–1.92	0.53–0.80	0.46–0.58	0.50–0.59	0.03–0.07	0.06–0.08	0.14–0.17	0.12–0.14	0.12–0.17	0.31–0.42	0.26–0.34	0.14–0.18	0.06–0.10	0.20–0.26
	dorsostriatus	7m, 4f	2.03–2.65	0.83–1.03	0.47–0.55	0.53–0.73	0.05–0.08	0.06–0.08	0.17–0.20	0.16–0.20	0.14–0.16	0.32–0.37	0.36–0.40	0.12–0.14	0.08	0.24–0.28
	namaka	3m, 1f	2.30–2.58	0.82–0.97	0.44–0.48	0.85	0.05–0.08	0.06–0.08	0.22–0.23	0.19–0.20	0.16	0.39	0.44	0.13	0.08	−
**minutus**	minutus	9m, 9f	1.42–1.98	0.62–0.88	0.41–0.50	0.45–0.59	0.05–0.07	0.07–0.11	0.14–0.17	0.14–0.17	0.14–0.17	0.38–0.45	0.28–0.39	0.14–0.18	0.06–0.07	0.19–0.23
	gibbosus	1m, 5f	1.71–2.01	0.73–0.86	0.48–0.52	0.48–0.55	0.02–0.04	0.07–0.08	0.16	0.16	0.18	0.31–0.40	0.26–0.35	0.13–0.16	0.06–0.07	−
**kamua**	iolani	3m, 4f	2.62–3.66	1.15–1.60	0.67–0.75	1.26–1.40	0.12–0.14	0.08–0.11	0.22–0.25	0.21–0.23	0.23–0.24	0.43–0.45	0.38–0.41	0.10–0.12	0.13–0.14	0.29
	hiiaka	3m, 3f	1.96–2.93	0.88–1.28	0.60–0.68	0.95–1.20	0.10–0.13	0.09–0.11	0.25–0.26	0.25–0.27	0.25–0.26	0.47–0.48	0.39–0.44	0.10–0.12	0.09	0.25–0.27
	melanoneurus	3m, 3f	2.73–3.60	1.17–1.53	0.69–0.78	1.17–1.45	0.12–0.15	0.09–0.11	0.26–0.29	0.25–0.27	0.20–0.25	0.46–0.52	0.51–0.55	0.11	0.09–0.12	0.29
	grandis	1m, 1f	2.95–4.08	1.28–1.78	0.74–0.79	1.40–1.55	0.13	0.10–0.12	0.31	0.27	0.27	0.85	0.84	0.10	0.11	0.34
	caulicalix	6m, 6f	1.61–2.24	0.54–0.90	0.45–0.59	0.60–0.81	0.05–0.08	0.09–0.12	0.21–0.24	0.19–0.21	0.18–0.23	0.46–0.55	0.37–0.47	0.15–0.19	0.08–0.09	0.18–0.24
	crassiorcalix	4m, 5f	1.62–2.18	0.64–0.85	0.45–0.55	0.64–0.76	0.06–0.08	0.08–0.11	0.20–0.22	0.18–0.20	0.18–0.20	0.46–0.48	0.42–0.45	0.10–0.16	0.08–0.09	0.24–0.26
	lehua	2m, 1f	1.94–2.09	0.79–0.82	0.50	0.73–0.89	0.08–0.09	0.08–0.09	0.21	0.19	0.18	0.50	0.43	0.14	0.08	0.25
	elegans	0m, 1f	2.15	0.89	0.58	0.82	0.08	0.12	−	−	−	0.73	0.62	0.18	0.09	0.26
	gagneae	0m, 1f	2.38	1.00	0.55	0.75	0.05	0.09	−	−	−	0.50	0.41	0.19	0.08	0.24
	haumea	1m, 0f	1.70	0.67	0.44	0.55	0.08	0.06	0.15	0.14	0.15	−	−	−	−	−
**ohialoha**	oahuensis	18m, 15f	2.38–3.55	0.94–1.52	0.62–0.73	1.16–1.46	0.14–0.27	0.08–0.14	0.15–0.28	0.22–0.30	0.20–0.27	0.60–0.98	0.49–0.68	0.08–0.13	0.08–0.10	0.23–0.28
	ohiacola	20m, 17f	1.86–3.42	0.67–1.30	0.47–0.70	0.78–1.26	0.10–0.23	0.07–0.09	0.17–0.26	0.18–0.28	0.16–0.25	0.47–0.88	0.36–0.56	0.09–0.17	0.08–0.11	0.17–0.24
	molokaiensis	6m, 5f	2.50–3.40	1.00–1.45	0.64–0.66	1.09–1.60	0.15–0.28	0.09–0.12	0.22–0.26	0.26–0.29	0.22–0.27	0.57–0.78	0.49–0.69	0.09–0.13	0.10–0.11	0.23–0.30
	hualani	4m, 4f	1.94–2.68	0.76–1.06	0.52–0.63	0.94–1.16	0.15–0.25	0.07–0.09	0.17–0.18	0.18–0.20	0.15–0.17	0.38–0.43	0.29–0.31	0.09–0.12	0.11–0.12	0.18–0.20
	mauiensis	3m, 4f	2.70–3.20	1.04–1.27	0.60–0.67	1.25–1.45	0.24–0.31	0.09–0.11	0.22–0.24	0.25–0.26	0.20–0.21	0.55–0.59	0.41–0.50	0.11–0.14	0.12–0.13	0.24–0.26
	kupua	4m, 2f	2.68–3.39	1.03–1.30	0.61–0.71	1.18–1.48	0.20–0.32	0.09–0.12	0.25–0.26	0.28–0.32	0.25–0.27	0.62–0.75	0.55–0.68	0.14–0.19	0.10–0.11	0.25
	montgomeri	7m, 5f	2.08–2.88	0.80–1.15	0.53–0.65	0.90–1.21	0.08–0.13	0.08–0.09	0.20–0.23	0.19–0.23	0.18–0.22	0.55–0.70	0.41–0.50	0.10–0.15	0.09–0.11	0.20–0.22
	hawaiiensis	6m, 6f	2.61–3.73	1.03–1.50	0.53–0.78	1.21–1.50	0.18–0.27	0.10–0.12	0.23–0.31	0.24–0.28	0.21–0.25	0.54–0.65	0.43–0.63	0.10–0.17	0.10–0.12	0.26–0.28
	pele	11m, 12f	1.70–2.98	0.67–1.15	0.43–0.65	0.76–1.23	0.11–0.22	0.07–0.11	0.17–0.25	0.18–0.24	0.16–0.22	0.37–0.54	0.31–0.44	0.09–0.15	0.10–0.12	0.19–0.22
	pyramidalis	5m, 5f	1.83–2.93	0.73–1.18	0.43–0.65	0.93–1.20	0.09–0.14	0.08–0.11	0.22–0.24	0.24–0.30	0.21–0.25	0.55–0.73	0.49–0.63	0.10–0.13	0.09	0.23–0.24

**Table 5.** Adult Pariaconus ratios.

Group	species	WL:WW	CUR	MR	HW:VW	VL:GP	VL:VW	AL:HW	HW:HT
**bicoloratus**	nigricapitus	2.38–2.50	0.96–1.19	0.86–0.93	1.82–1.89	3.61–6.00	0.68–0.78	1.19–1.56	0.96–1.11
	hina	2.35–2.54	1.04–1.30	0.78–1.07	1.78–2.00	3.33–7.00	0.56–0.75	1.28–1.48	0.94–1.13
	wyvernus	1.92–2.52	0.81–1.20	0.77–1.00	1.78–2.00	3.25–6.50	0.63–0.78	1.14–1.59	0.95–1.21
	nigrilineatus	2.37–2.40	1.00	0.79–1.00	1.60–1.73	4.33–6.50	0.65–0.71	1.12–1.19	1.06–1.10
	kapo	2.38	1.19	0.92	2.13	2.80	0.78	1.61	0.87
	proboscideus	2.36–3.05	0.95–1.15	0.79–0.95	1.72–1.82	2.95–4.67	0.69–0.82	1.47–1.58	0.86–0.94
	poliahu	2.35–2.45	0.95–1.09	0.76–0.95	1.82–1.94	4.00–6.00	0.61–0.75	1.30–1.55	1.00–1.11
	lona	2.24–2.56	1.00–1.12	0.78–0.96	1.81–1.88	2.20–4.33	0.58–0.76	1.37–1.40	0.89–1.01
	liliha	2.66–2.73	1.14–1.44	0.75–0.89	1.79	3.67–5.50	0.56–0.63	1.47	1.09–1.16
	gracilis	2.22–2.54	0.86–1.17	0.83–1.00	1.70–1.95	3.25–8.00	0.61–0.84	0.94–1.10	1.16–1.45
	dorsostriatus	2.44–2.63	1.08–1.24	0.78–0.92	1.74–2.00	2.40–5.33	0.58–0.89	1.13–1.33	0.85–0.97
	namaka	2.66–2.81	1.43–1.68	0.43–0.88	1.82–1.93	2.20–2.50	0.59–0.73	−	0.78
**minutus**	minutus	2.21–2.48	0.95–1.12	0.67–1.00	1.79–2.07	2.17–4.33	0.59–0.86	1.00–1.26	0.87–1.13
	gibbosus	2.24–2.36	0.95–1.05	0.86–1.06	1.88–2.00	5.42–11.00	0.65–0.88	0.94–1.13	1.06–1.14
**kamua**	iolani	2.28–2.40	1.08–1.19	0.71–0.83	1.71–1.87	1.88–2.20	0.58–0.70	1.70–2.00	0.94–1.10
	hiiaka	2.15–2.30	1.10–1.27	0.75–0.92	1.73–1.86	1.80–2.25	0.60–0.71	1.58–1.85	0.84–0.98
	melanoneurus	2.31–2.41	1.37–1.62	0.88–0.95	1.72–1.87	1.67–2.13	0.56–0.67	1.66–2.07	0.85–0.89
	grandis	2.30–2.31	1.22–1.25	0.72–0.79	1.70–1.79	1.90–2.20	0.58–0.59	1.90–1.97	0.83–0.89
	caulicalix	2.44–3.21	1.00–1.26	0.78–0.95	1.82–2.05	2.33–4.00	0.57–0.78	1.20–1.63	0.95–1.16
	crassiorcalix	2.47–2.71	1.11–1.29	0.79–1.13	1.72–1.94	2.20–3.64	0.67–0.80	1.22–1.61	1.18–1.45
	lehua	2.37–2.65	1.32–1.33	0.83–0.85	1.74	2.00–2.60	0.63–0.68	1.45–1.79	1.14–1.32
	elegans	2.41	1.09	0.96	1.90	3.00	0.75	1.42	0.93
	gagneae	2.38	1.26	0.88	2.00	3.50	0.64	1.36	0.94
	haumea	2.55	1.12	0.86	1.93	2.00	0.67	1.24	1.04
**ohialoha**	oahuensis	2.28–2.79	0.97–1.29	0.73–1.27	1.68–1.93	1.13–2.12	0.60–0.86	1.79–2.25	0.80–1.00
	ohiacola	2.48–3.41	1.08–1.57	0.55–1.04	1.71–2.08	1.07–2.25	0.58–0.83	1.63–1.88	0.86–1.24
	molokaiensis	2.05–2.58	1.05–1.43	0.83–1.06	1.76–2.00	0.90–1.63	0.67–0.89	1.85–2.38	0.85–1.02
	hualani	2.45–2.56	1.10–1.28	0.81–1.09	1.81–2.00	1.05–1.50	0.72–0.90	1.73–1.94	0.96–1.17
**ohialoha**	mauiensis	2.51–2.60	1.09–1.24	0.74–0.91	1.76–1.93	0.80–1.05	0.71–0.83	1.92–2.23	0.96–1.05
	kupua	2.51–2.61	1.18–1.30	0.82–0.93	1.82–1.93	0.86–1.13	0.64–0.77	1.86–2.11	0.90–1.00
	montgomeri	2.47–2.66	1.12–1.52	0.74–1.00	1.80–2.09	1.90–2.86	0.73–0.86	1.71–2.07	0.95–1.11
	hawaiiensis	2.42–2.63	1.00–1.24	0.70–0.96	1.50–1.93	0.94–1.43	0.65–0.80	1.90–2.22	0.84–1.07
	pele	2.40–2.65	0.96–1.36	0.77–0.95	1.67–2.00	1.04–2.00	0.64–0.92	1.63–1.95	0.96–1.19
	pyramidalis	2.48–2.62	1.06–1.27	0.81–0.94	1.70–2.10	1.64–2.25	0.69–0.90	1.64–1.85	0.92–1.02

**Table 6.** Adult Pariaconus ratios for male and female terminalia and eggs.

Group	Species	PL:HW	MP:PL	PL:AEL	AEL:AELH	PL:SH	FP:HW	FP:RL	FP:SP	EL:EW
**bicoloratus**	nigricapitus	0.31	1.20	1.00	2.00	0.95	0.63–0.72	1.88–2.53	1.34–1.48	1.88–2.29
	hina	0.26	1.16–1.17	0.97–1.03	1.88–2.21	0.75–0.78	0.54–0.67	1.84–2.24	1.28–1.70	2.23–3.05
	wyvernus	0.31–0.35	0.96–1.10	0.84–1.00	2.08–2.27	0.89–1.05	0.66–0.73	1.92–2.24	1.35–1.57	2.85
	nigrilineatus	−	−	−	−	−	0.87	3.31–3.71	1.33–1.37	3.18–3.78
	kapo	−	−	−	−	−	1.06	3.50	1.15	−
	proboscideus	0.37–0.40	1.04–1.21	0.95–1.12	2.28–2.63	0.91–1.05	1.12–1.19	2.82–3.09	1.16–1.50	2.12
	poliahu	0.29	1.21–1.24	0.72–0.79	2.10–2.68	0.76–0.77	0.67–0.73	1.87–2.14	1.30–1.55	3.00
	lona	0.36	1.14	0.91	2.30	0.84	1.00–1.06	3.37–3.45	1.27–1.33	2.33
	liliha	0.26	1.18	1.03	2.20	0.76	0.66–0.67	1.92–2.05	1.48	3.36
	gracilis	0.25–0.27	1.12–1.38	0.78–0.97	2.35–3.57	0.69–0.76	0.63–0.74	2.05–2.82	1.11–1.48	2.78–3.57
	dorsostriatus	0.33–0.39	0.90–1.07	1.15–1.31	1.90–2.27	0.92–1.07	0.60–0.71	2.50–2.80	0.84–1.02	2.67–2.92
	namaka	0.44	1.13	1.17	2.41	1.00	0.83	3.06	0.89	−
**minutus**	minutus	0.33–0.39	0.86–1.11	0.98–1.09	2.27–2.73	0.87–1.00	0.85–0.97	2.55–2.78	1.08–1.35	1.81–3.11
	gibbosus	0.32	1.03	0.89	2.75	0.85	0.75–0.78	2.50–2.53	1.09–1.16	−
**kamua**	iolani	0.31–0.34	0.97–1.15	0.87–1.00	2.10–2.31	0.60–0.73	0.59–0.60	3.83–4.50	1.06–1.18	2.16–2.40
	hiiaka	0.41–0.42	0.96	1.02–1.04	1.95–2.07	1.00–1.04	0.70–0.74	4.00–4.67	1.09–1.23	1.91–2.07
	melanoneurus	0.36–0.39	1.00–1.07	1.04–1.32	1.92–2.66	0.93–1.05	0.67–0.72	4.27–4.82	0.90–0.95	1.84–1.93
	grandis	0.37	1.14	1.00	2.14	0.93	1.08	8.70	1.01	2.14
	caulicalix	0.35–0.42	1.08–1.20	0.89–1.08	2.32–3.13	0.95–1.08	0.82–1.02	2.76–3.20	1.07–1.24	2.60–3.37
	crassiorcalix	0.38–0.40	1.09–1.22	0.94–1.02	2.33–2.67	1.05–1.05	0.85–0.97	3.33–4.62	1.04–1.15	2.13
	lehua	0.38	1.08	1.04	2.30	0.96–1.04	1.01	3.50	1.17	2.07
	elegans	−	−	−	−	−	1.26	3.96	1.17	2.36
	gagneae	−	−	−	−	−	0.91	2.68	1.21	2.94
	haumea	0.31	1.12	0.92	2.18	0.77	−	−	−	−
**ohialoha**	oahuensis	0.35–0.43	0.61–1.02	1.06–1.30	2.13–3.00	1.00–1.19	0.87–1.31	5.71–9.38	1.12–1.46	1.45–2.06
	ohiacola	0.33–0.44	0.78–0.96	1.00–1.29	2.01–2.68	1.04–1.26	0.78–1.32	3.18–6.47	1.18–1.67	1.20–1.51
	molokaiensis	0.42–0.44	0.79–0.93	1.04–1.20	1.94–2.37	0.92–1.07	0.95–1.12	5.27–7.50	1.14–1.17	1.66–1.80
**ohialoha**	hualani	0.33–0.34	0.88–0.95	1.12–1.26	1.95–2.21	0.92–1.10	0.64–0.73	3.58–4.56	1.26–1.43	1.23–1.53
	mauiensis	0.41–0.42	0.86–0.94	1.21–1.29	1.93–2.21	1.00–1.04	0.83–0.89	4.21–5.09	1.10–1.40	1.68–1.94
	kupua	0.46–0.50	0.76–0.94	1.13–1.24	2.07–2.32	1.03–1.14	0.98–1.06	3.92–4.59	1.11–1.13	1.91
	montgomeri	0.35–0.41	0.87–1.03	1.00–1.10	2.45–2.89	1.00–1.24	1.02–1.07	4.47–6.00	1.30–1.40	1.32–1.54
	hawaiiensis	0.36–0.52	0.88–1.10	1.09–1.26	2.01–2.32	0.94–1.17	0.83–0.93	3.72–5.46	0.97–1.31	1.83–1.92
	pele	0.33–0.43	0.88–1.14	0.95–1.19	2.10–2.50	0.94–1.09	0.71–0.93	3.64–4.82	1.19–1.30	1.44–1.80
	pyramidalis	0.47–0.55	0.76–0.92	1.09–1.25	1.96–2.45	1.04–1.15	0.95–1.17	5.15–7.30	1.12–1.23	1.76–1.94

**Table 7.** Pariaconus immatures: 5^th^ instar measurements (mm) and ratios.

Group	Species	n	BL	BW	WPL	CPL	CPW	RW	HW	AL	BL:BW	HW:AL	CPW:RW
**bicoloratus**	nigricapitus	2	1.36–1.48	1.15	0.79	0.42–0.48	0.97–1.03	0.19–0.21	0.56–0.57	0.15–0.16	1.18–1.29	3.55–3.68	4.95–5.05
	proboscideus	5	1.41–1.55	0.98–1.12	0.73–0.76	0.58–0.73	0.73–0.88	0.21–0.22	0.51–0.64	0.21–0.23	1.32–1.45	2.24–3.08	3.37–4.08
	gracilis	2	1.36–1.70	0.82–1.00	0.61–0.76	0.55–0.65	0.70–0.85	0.15–0.20	0.50–0.56	0.16–0.18	1.67–1.70	3.15–3.18	4.24–4.58
	dorsostriatus	6	1.33–1.52	1.03–1.27	0.76–1.00	0.55–0.64	0.91–1.06	0.22–0.25	0.47–0.54	0.17–0.19	1.15–1.32	2.68–2.86	4.06–4.42
	namaka	5	1.30–1.48	1.03–1.15	0.76–0.88	0.50–0.55	0.88–0.97	0.21–0.24	0.46–0.50	0.18–0.21	1.26–1.35	2.31–2.59	3.85–4.22
**minutus**	minutus	5	1.00–1.18	0.85–0.94	0.61–0.64	0.36–0.45	0.64–0.73	0.13–0.16	0.42–0.45	0.16–0.17	1.13–1.39	2.52–2.75	4.55–4.97
**kamua**	hiiaka	6	1.45–2.00	1.03–1.36	0.61–0.88	0.76–1.06	0.82–1.15	0.03–0.05	0.53–0.70	0.22–0.32	1.41–1.50	2.15–2.50	20.45–35.98
	caulicalix	1	1.18	1.00	0.64	0.48	0.85	0.14	0.51	0.17	1.18	3.05	5.89
	crassiorcalix	5	1.15–1.18	0.79–0.88	0.61–0.64	0.45–0.45	0.70–0.76	0.14–0.16	0.44–0.49	0.16–0.18	1.34–1.46	2.39–2.95	4.73–5.26
**ohialoha**	oahuensis	6	1.85–2.33	1.33–1.52	0.82–0.91	0.91–1.24	1.12–1.36	0.04–0.06	0.72–0.78	0.39–0.44	1.36–1.60	1.70–1.94	20.02–28.79
	ohiacola	3	1.21–1.61	0.97–1.09	0.61–0.70	0.61–0.79	0.73–0.91	0.03–0.06	0.54–0.56	0.34–0.36	1.25–1.47	1.56–1.67	15.15–28.41
	kupua	1	1.82	1.18	0.82	0.76	1.03	0.06	0.70	0.39	1.54	1.80	18.40
	montgomeri	5	1.70–2.00	1.12–1.30	0.70–0.85	0.76–1.06	0.94–1.12	0.16–0.17	0.62–0.72	0.32–0.42	1.42–1.58	1.54–2.25	5.59–7.01
	hawaiiensis	2	2.33–2.61	1.55–1.73	0.94–1.06	0.97–1.09	1.09–1.36	0.06	0.80	0.46–0.48	1.51	1.67–1.75	19.48–21.30
	pele	7	1.45–1.88	1.00–1.24	0.67–0.85	0.70–0.97	0.73–1.03	0.03–0.06	0.56–0.70	0.36–0.44	1.43–1.51	1.54–1.82	15.15–32.19
	pyramidalis	6	1.45–2.12	1.06–1.39	0.73–0.91	0.64–1.06	0.76–1.06	0.05–0.06	0.58–0.78	0.32–0.42	1.37–1.56	1.76–1.92	11.84–22.09

## Results

### Taxonomic placement of Pariaconus and outgroup analysis

**Figure 1.**
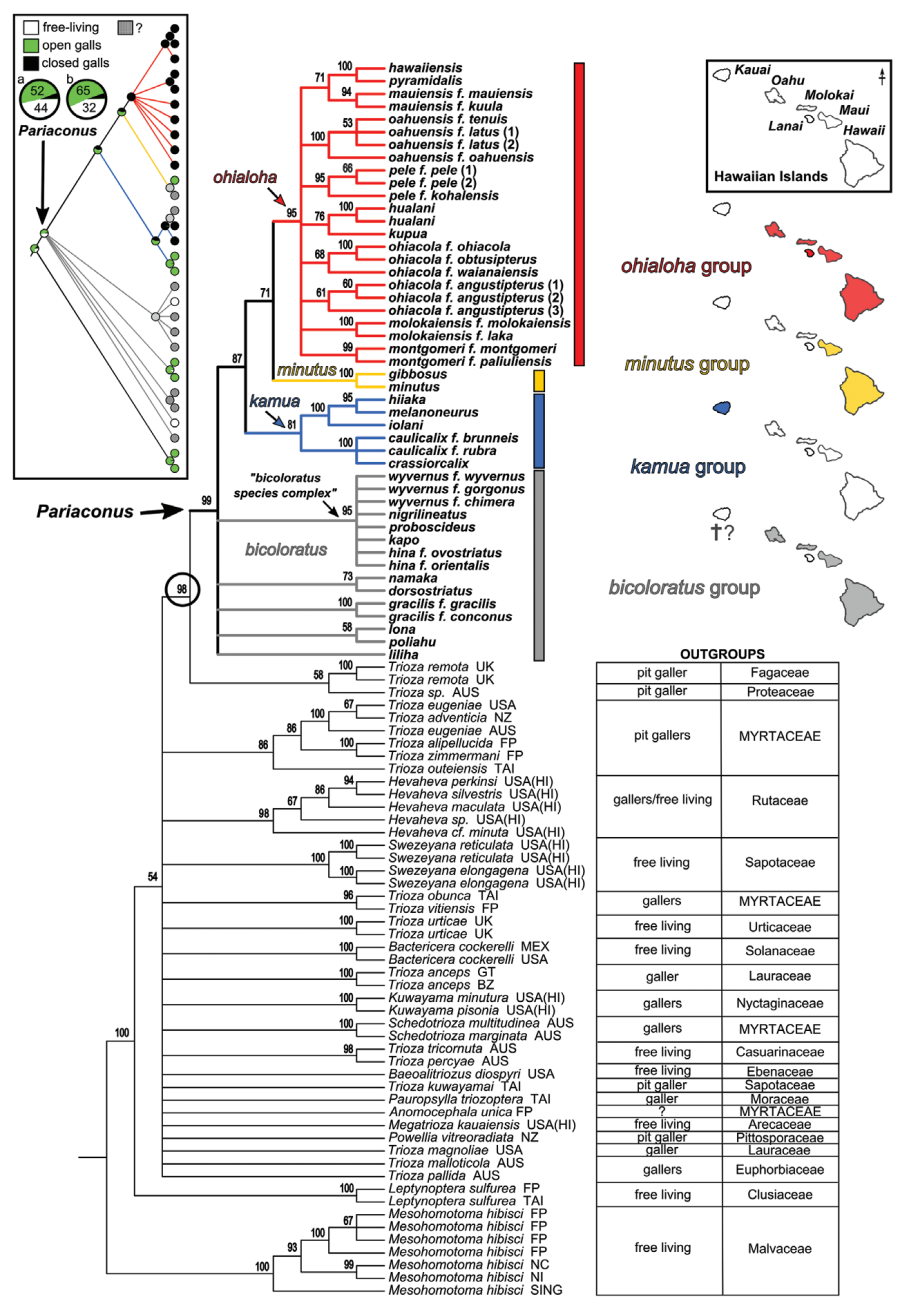
Results from outgroup sampling. Majority-rule consensus of maximum likelihood analysis (combined COI and cytB data using RAxML with 1000 bootstrap replicates) including Pariaconus (28 species) and putative outgroup taxa (36 species). The four recognized species groups within Pariaconus are indicated and their distribution in the Hawaiian Islands is shown. Outgroup biology and host plant family is given, with other Myrtaceae-feeding species highlighted. Strong support for Trioza remota ex Quercus (Palaearctic) and Trioza sp. ex Banksia (Australia) as the closest outgroups is indicated with a black circle. Inset top left, maximum likelihood ancestral character state reconstruction (character -lnL = -16.48) showing proportional likelihoods, and the ancestral state at the root node of Pariaconus with a) pit galling outgroup taxa monophyletic, and b) pit galling outgroup taxa paraphyletic.

Combined analysis of the two mitochondrial regions provides two robustly supported key results: a) confirmation of the monophyly of the genus Pariaconus, b) strong support for a sister relationship with pit-galling species from Asia-Australasia as the closest known relatives of Pariaconus (Fig. 1). There is strong support for the monophyly of Pariaconus (i.e. both taxa previously placed in Trioza and Kuwayama) (ML: 99%, NJ: 100%), and robust support for an ancestral outgroup (ML: 98%, NJ: 98%), which may be suggestive of a leaf pit-galling ancestry (see results of ancestral character state reconstruction below). The most unexpected discovery is the identity of the closest outgroup taxa, which are not among other Pacific Metrosideros- or Myrtaceae-feeders, but include Trioza remota Foerster, 1848, a Palaearctic species making barely noticeable pit galls on the leaves of oaks (Quercus spp.; Fagaceae), and an undescribed species from Australia making deep pit galls on Banksia (Proteaceae) leaves. Other Myrtaceae-feeders, including those on Metrosideros, from the Australasian and Pacific regions do not cluster close to Hawaiian Pariaconus. The French Polynesian taxa on Metrosideros are a separate radiation of species related to Myrtaceae-feeders from Asia and Australia (Trioza outeiensis Yang, 1984, Trioza eugeniae Froggatt, 1901) and also cluster with taxa galling Eucalyptus and other Myrtaceae (Schedotrioza spp., Trioza obunca Fang & Yang, 1986, Trioza vitiensis Kirkaldy, 1907). Trioza eugeniae is an Australian Myrtaceae-feeder associated with Syzygium in its native Australian range, but in its introduced range in California it occurs on cultivated Metrosideros (Percy et al. 2012); this species was therefore considered another putative outgroup for Pariaconus. The phylogenetic analyses revealed it is not related to Pariaconus, but rather groups with the other Pacific-Australasian Myrtaceae-feeders described above. Some doubts about the correct identity of the Californian specimens came to light during this study. Specimens of introduced Trioza eugeniae sampled from three localities in California are in fact closer to a species described from New Zealand, Trioza adventicia Tuthill, 1952 (specimens supplied by Pam Dale, Plant Protection Centre, MAF, Auckland) which feeds on Angophora floribunda, Syzygium smithii, and Syzygium paniculatum (all Myrtaceae) in New Zealand (Martoni et al. 2016, Pam Dale pers. comm.). Tuthill (1952) recognized Trioza adventicia as introduced to New Zealand, and based on a morphological examination as well as the molecular analyses, both the Californian and New Zealand specimens sampled here are likely the same species. Despite Tuthill (1952) examining the type material of Trioza eugeniae when he described Trioza adventicia, he mentioned only one distinguishing character between them, apical metatibial spurs 2+1 (Trioza adventicia) versus 3+1 (Trioza eugeniae). I have examined six specimens (3 male, 3 female) of Trioza eugeniae sourced from a single California population, and six specimens (5 male, 1 female) of Trioza adventicia from a single New Zealand population. The California material has four individuals with 2+1 spurs, one with 3+1, and one with both 2+1 and 3+1 (on right and left metatibia). The New Zealand material has five individuals with 2+1 and another with 2+1 and 3+1 (on right and left metatibia). There appear to be no distinguishing morphological characters to separate the Californian and New Zealand material. In conclusion, Californian and New Zealand specimens are likely the same species and may have been introduced from Australia over similar time periods, and possibly from a similar source area in Australia (based on minimal genetic distance). I have not examined sufficient material of Australian Trioza eugeniae, or type material of either species, which would be needed to confirm synonymization of Trioza adventicia with Trioza eugeniae, but molecular divergence between the Australian, and the Californian/New Zealand specimens is well within typical intraspecific distances for psyllids (Percy 2003b, Taylor et al. 2016). Froggatt (1901) mentions different forms of Trioza eugeniae, which he infers may be different species, suggesting more extensive sampling within Australia is needed to clarify the taxonomy of Trioza eugeniae. The illustration Froggatt (1901) provides of the male genitalia of Trioza eugeniae is dissimilar to that of the Californian and New Zealand material I have examined, and as Tuthill was convinced, based on his examination of Trioza eugeniae type material, of the distinctiveness of Trioza adventicia, this issue is here flagged as needing further investigation.

### Ancestral character state reconstruction: a galling or non-galling origin?

Parsimony reconstruction analysis resolved the ancestral state for Pariaconus as open galling (e.g. pit/cup gallers) with four steps between states within Pariaconus, but character consistency and retention scores were relatively low (CI: 0.5, RI 0.71). The maximum likelihood analysis also recovered open galling as the ancestral state but the margin in proportional likelihoods between open galling and free living is not large (Pariaconus root node with pit galling outgroup taxa monophyletic: 0.52 open galling versus 0.44 free living, and with pit galling outgroup taxa paraphyletic: 0.65 open galling versus 0.32 free living) (see inset in Fig. 1). In all analyses, closed galling is derived within Pariaconus with no reversals. The ancestral trait analysis results remain somewhat equivocal due to the number of unknown biologies within Pariaconus, as well as the lack of firm resolution at the base of the Pariaconus radiation. Finally, lack of knowledge of ancestors and tree topology beyond the two outgroup pit gallers hinders confidence in resolving the question of the original biology, and therefore the results remain only suggestive of the ancestral state and potential evolutionary transitions in galling biology.

### Divergence within Pariaconus

Maximum inter-specific molecular divergence within Pariaconus as a whole (across all four species groups) is 16.9%. This can be compared with divergence within Swezeyana and Hevaheva, which, although not as comprehensively sampled, provide maximum divergence estimates of 18% and 17.5% respectively. These comparative divergences suggest Swezeyana and Hevaheva may be marginally older genera that established earlier in the Hawaiian Islands. Mitochondrial rates of divergence vary across different organismal lineages, even among insects (Shapiro et al. 2006, Magnacca and Danforth 2007, Goodman et al. 2012, Haines et al. 2014), and rate heterogeneity has been reported within psyllid lineages (e.g. Percy et al. 2004). However, assuming a mitochondrial molecular clock can still provide a reasonable range of ages in many cases (Papadopoulou et al. 2010, Wessel et al. 2013, Parmakelis et al. 2015). If we assume a range of mtDNA rates between 2.5–5% divergence per million years, then the length of time Pariaconus has been diversifying in the Hawaiian Islands is likely between 3.5–6.5 Myr. This range is also consistent with estimates determined from observed divergence and island age. For instance, if the maximum divergence found in Pariaconus (16.9%) is a product of diversification since the emergence of Kauai (~5 Myr), then divergence rate is estimated at ~3.4% per million years, however, the ohialoha group, which only occurs on the younger islands (with island ages of ~3–3.5 Myr and younger) would suggest that the divergence rate is at least as high as 4.5–5.3% per million years.

**Figure 2.**
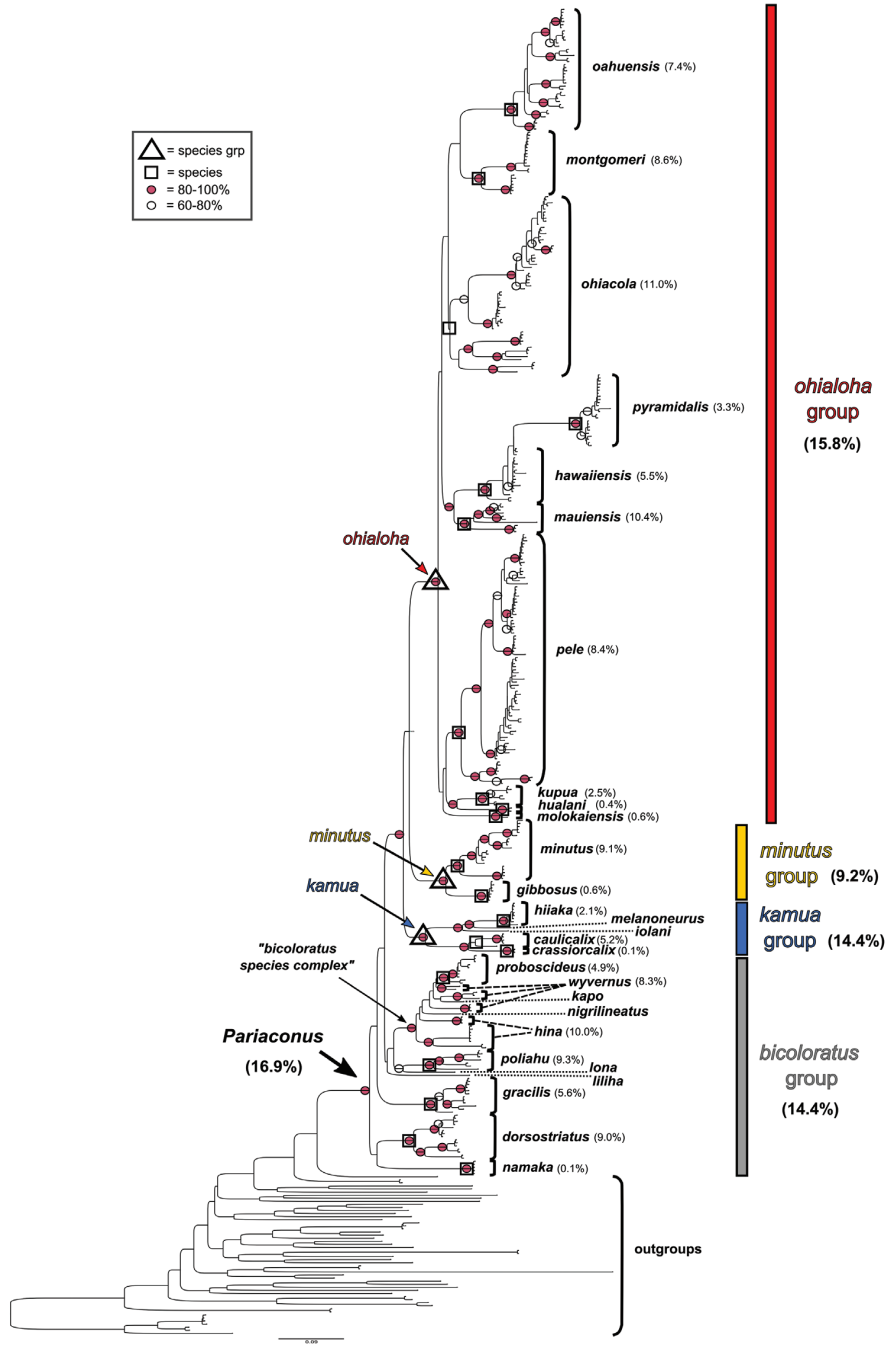
Best maximum likelihood topology (-lnL = -33769.25, combined COI and cytB data using RAxML with 1000 bootstrap replicates). Included are 432 unique haplotypes (381 within Pariaconus and 51 representing outgroups) recovered from 537 individuals sampled. The four recognized species groups within Pariaconus are indicated as well as the “bicoloratus species complex” and 28 described species. Moderate to strong support for nodes within Pariaconus is indicated. Dotted lines indicate species either with only one individual sampled or several individuals with only one unique haplotype. Dashed lines indicate two species (Pariaconus hina, Pariaconus wyvernus) for which intraspecific haplotypes were not recovered as monophyletic, both species are within the “bicoloratus species complex”. Two taxa (Pariaconus ohiacola, Pariaconus caulicalix) are recovered as topologically monophyletic (in both ML and NJ analysis) but without bootstrap support. Two species are recovered as sister taxa in NJ analysis (Pariaconus hawaiiensis, Pariaconus pyramidalis), but here the ML analysis places one (Pariaconus pyramidalis) on a long branch nested within the other (Pariaconus hawaiiensis), reflecting a likely evolutionary gall shift in situ on Hawaii. All other taxa, including recognized intraspecific forms and variations, are reasonably to strongly supported as monophyletic (80-100% support). Maximum genetic distance within species groups and maximum intraspecific genetic distances (p-distance in PAUP*) are shown in parenthesis for all taxa with more than one haplotype.

Based on morphological and molecular analyses, four species groups are recognized (Figs 1–2). The bicoloratus and minutus groups are free-living or form pit galls (formerly Kuwayama spp.). These taxa are challenging because they include most of the small sized species, which are often less abundant than those in the ohialoha group; and they are often more difficult to detect in the field, particularly the free-living immatures, because there are few or no visible indications of presence on the plant. In addition, when pit galls are shallow they are relatively inconspicuous compared to enclosed galls.

The bicoloratus group is one of the oldest, and possibly the ancestral group in the Hawaiian radiation, but interestingly the greatest extant species diversity in this group is found on the youngest island of Hawaii, although subfossil remains closely resembling extant bicoloratus-type immatures have recently been found on Kauai (Nicholas Porch pers. comm.). The kamua and minutus groups are also likely older than the ohialoha group, but currently the minutus group is only known from Maui and Hawaii. The kamua group is the only single island lineage and includes all of the known Kauai species. The range of morphologies and galling biologies, and the restriction to the oldest extant island of Kauai, also make this group a plausible ancestral group in the radiation. However, of the four larger islands (Kauai, Oahu, Maui, Hawaii), Kauai is the least well sampled with more collecting needed to determine both the habits and variety of galling biologies, as well as the potential presence of bicoloratus and minutus group taxa on Kauai. The ohialoha group includes all of the closed galling species not in the kamua group, and is found on all islands except Kauai; it appears to be the most derived group and exhibits dynamic patterns of variation suggestive of ongoing speciation processes.

Recognized morphological forms provide information about the extent and distribution of morphological variation within, in some cases, relatively broad species concepts, and a number of the more distinct forms may eventually require recognition at species level.

### Divergence within the bicoloratus group

There are 12 species in the bicoloratus group and maximum interspecific divergence is 14.4% (maximum intraspecific divergence 10%). This is the only species group not resolved as monophyletic, rather it constitutes a basal grade of taxa likely representing early divergence in Pariaconus (Figs 1–2). The recent discovery of subfossils on Kauai from this group (Nicholas Porch pers. comm.) also supports its ancestral position in the radiation of the genus, although the absence thus far of any extant species from Kauai remains puzzling. There is a subgroup within the bicoloratus group, namely the “bicoloratus species complex”, which encompasses complex patterns of morphological and genetic variation not easily interpreted, including two taxa not resolved as monophyletic in either ML or NJ analyses (Pariaconus hina, Pariaconus wyvernus). Notably, many species are only known from one or few localities, which contrasts with the widespread distributions of many of the closed gallers in the ohialoha group (Figs 53–55)

### Divergence within the minutus group

There are two species in the minutus group, and both are well represented in the molecular dataset with 9.2% maximum interspecific divergence (maximum intraspecific divergence 9.1%). Within group divergence appears modest compared to other species groups, and currently most of the genetic divergence is found within Pariaconus minutus on Hawaii (Fig. 2), however, the species on Maui, Pariaconus gibbosus, is likely undersampled.

### Divergence within the kamua group

There are 10 species in the kamua group, with maximum interspecific divergence 14.4%, but molecular data are only available for five species and insufficient individuals were sampled to gauge typical intraspecific divergence, except perhaps in Pariaconus caulicalix with 5.2% intraspecific divergence. The kamua group exemplifies within island diversification encompassing a variety of biologies: closed and open galls, and a large range of body sizes. All sampled species were collected within a small geographic area on Kauai (Fig. 54) emphasising the co-occurrence of taxa and the likely discovery of more diversity with broader geographic sampling on Kauai.

### Divergence within the ohialoha group

There are 12 species in the ohialoha group; molecular data are presented for 10 species and maximum interspecific divergence is 15.8% (maximum intraspecific divergence 11%). Slightly greater genetic distances in the younger ohialoha group are likely a result of more comprehensive sampling but could also suggest an accelerated molecular divergence rate related to a shift to the closed galling biology. All species, with the exception of Pariaconus ohiacola, are well supported, but there is a notable lack of support at the base of the ohialoha group suggesting a rapid radiation occurred after a shift to the closed galling biology (Figs 1–2); subsequent shifts to galling different plant parts then promoted further diversification within the group. Notably, a strongly supported sister relationship between the stem/bud galler, Pariaconus hawaiiensis, and cone leaf galler, Pariaconus pyramidalis, on Hawaii, provides support for in situ (within island) galling shifts. A sister relationship between a stem galler, Pariaconus kupua on Maui, and a leaf galler, Pariaconus hualani on Molokai, also suggest these biological shifts happened repeatedly, both within and between islands.

**Figure 3.**
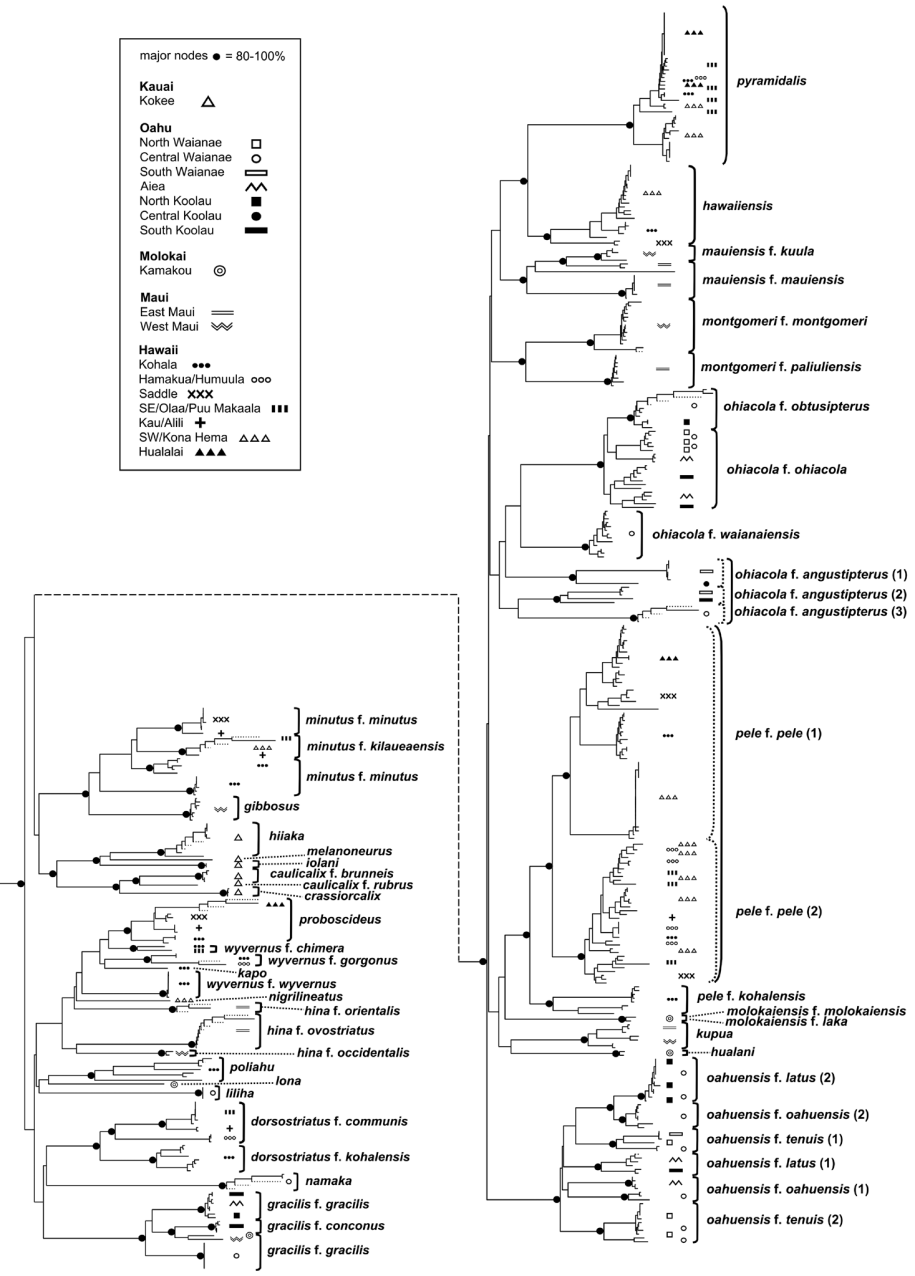
Neighbour-joining analysis (combined COI and cytB data with 1000 bootstrap replicates in PAUP*). The analysis included all 537 sampled individuals, shown here are the 479 individuals within Pariaconus. Described species and recognized forms are indicated as well genotypic clusters within forms. Regional locations for sampling sites within islands are shown to give an idea of geographic clustering and isolation (e.g. Pariaconus pele form pele cluster 1 has regionally distinct clusters, whereas Pariaconus pele form pele cluster 2 is composed of regionally mixed clusters). Support (80-100%) is indicated for major nodes only.

Notable morphological variation is evident for several species, which has resulted in the recognition, in this treatment, of a number of morphological forms. These forms are usually also identifiable in genotypic clusters, but in two widespread species on Oahu, Pariaconus oahuensis and Pariaconus ohiacola, divergent genetic clusters exist within the same form (Fig. 3). On Hawaii, a widespread species and form, Pariaconus pele form pele has two distinct genetic clusters which exhibit strikingly different geographic patterns (Pariaconus pele f. pele cluster 1 is composed of regionally distinct clusters, whereas Pariaconus pele f. pele cluster 2 is composed of regionally mixed clusters) despite both cluster 1 and cluster 2 occurring across a similar geographic breadth (Fig. 3); a second form, Pariaconus pele form kohalensis, is restricted to the Kohala region, and genetic divergence within this form is almost as great as within the much more widespread form pele distributed across the island; greater divergence in form kohalensis could reflect an older and relictual population in Kohala, which is the geologically oldest region of Hawaii. A number of other studies have reported higher genetic diversity in this region, including for the host plant, Metrosideros (Stacy et al. 2014).

Repeated patterns of intraspecific morphological variation in the ohialoha group suggests there may be substantial “standing morphological variation” underlying polymorphism in the genus as a whole. Whether this variation, and the apparent convergences in the ohialoha group, result from a process essentially akin to “morphological drift”, or whether selection is involved is not clear. If produced from standing allelic variation (Barrett and Schluter 2008), such polymorphisms may result from rapid adaptation. In the ohialoha group, parallel within species variation in body size, head shape, wing shape, and even genitalic characters, such as size and shape of female terminalia, and shape of male paramere and aedeagus results in some forms from different species appearing superficially similar. In contrast, the “bicoloratus species complex” exhibits something more akin to “morphological stasis” (e.g. Pariaconus wyvernus and Pariaconus hina), with comparatively little morphological divergence despite similarly high levels of genetic divergence.

## Discussion

### Origins of Pariaconus and other Myrtaceae-feeders

Crawford (1918) thought that the galling habit in itself indicated an affiliation between Hawaiian and Asian species as there is an unusually high percentage of gallers in some Asian psyllid faunas: in Taiwan and Japan 50% or more of the described psyllid species are gall making, and notably these oriental galling faunas are dominated by taxa in the family Triozidae (Yukawa and Masuda 1996; Yang and Raman 2007; Yang et al. 2013; Percy et al. 2015). What is particularly remarkable in Pariaconus is that within this lineage and over a relatively short period of evolutionary time, there have evolved many different galling habits as well as completely free-living species, and these shifts have all occurred within an insular radiation over a few million years. This makes Pariaconus unique among psyllids.

When considering the age of Pariaconus in the Hawaiian archipelago, Crawford (1918) remarked: “At what time after the establishment of the Ohia lehua [Metrosideros] here the gall psyllids came in is impossible to say, because of the absence of fossils”. The kamua group is endemic to the oldest island, Kauai, and recent discovery of subfossils of bicoloratus-type can place these putatively older groups on Kauai with both groups appearing to have diverged early in the evolutionary history of Pariaconus. However, similar degrees of mtDNA divergence within all species groups, as well as the lack of backbone resolution in the genus, implies there was rapid diversification soon after colonization of the Hawaiian Islands. The same range of dates for colonization and diversification by Pariaconus and Metrosideros (Percy et al. 2008) raises the possibility of concurrent in situ progression down the island chain, however this will need to be tested more rigorously with further double-dating analyses (Percy et al. 2004). Different pre-Hawaiian biogeographies: Metrosideros colonizing from the Marquesas (Wright et al. 2001, Percy et al. 2008), and Pariaconus apparently colonizing directly from Asia-Australasia via long distance dispersal (Gillespie et al. 2012), precludes a joint colonization event, nevertheless the date ranges suggest Pariaconus arrived in the Hawaiian Islands contemporaneously or soon after Metrosideros.

The closest sister group of the Hawaiian Metrosideros-feeding psyllids remains unknown, but this study confirms a phylogenetic outgroup association with pit-galling species from Australasia and the Palaearctic region on non-Myrtaceae hosts. Rather surprisingly, the pit-galling Metrosideros-feeders from French Polynesia, Fiji, Samoa, and Australasia, as well as other species galling Myrtaceae from Asia and Australasia represent a separate radiation on Myrtaceae. There may yet be an ancestral Myrtaceae-feeding sister taxon to Pariaconus still to be discovered, but it is clear that the Austro-Pacific Myrtaceae-feeders that have been sampled for this study are not close to Pariaconus. Based on the outgroup analysis and character reconstruction, it is an ancestral galling habit that is more likely to have been conserved during the colonization of the Hawaiian Islands, not the association with Myrtaceae. The data presented here strongly support a close relationship with two species in particular, although these are unlikely to be the closest relatives: the Palaearctic pit-galler of oaks, Trioza remota, which has a native distribution range from the UK to Japan (Hodkinson and White 1979, Burckhardt 1989, Önuçar and Ulu 1991, Ossiannilsson 1992, Malumphy et al. 2009), and an undescribed species making pit galls on Banksia in Australia (Tasmania). Trioza remota makes shallow pit galls on the leaves of Quercus; the Australian species makes deep pit galls on the leaves of Banksia, with the immatures situated at the base of the pit, in a similar manner to the cup gallers from Kauai (kamua group). The Banksia-feeder is likely related to two other described Australian species from Banksia (Taylor and Moir 2014), and one of these species, Trioza banksiae Froggatt, 1901, was noted as having free-living immatures (Froggatt 1901). If the Hawaiian lineage is derived from an ancestral lineage that also exhibits within lineage lability in biology, including galling and non-galling taxa, then this ancestral lability could partly explain the propensity for biological shifts in Pariaconus. The pit galling habit and reduced genal processes of these outgroup taxa also support the interpretation of bicoloratus, minutus, and kamua groups as ancestral to the closed galling ohialoha group, with the development of longer genal processes repeatedly derived in Pariaconus.

The question of long distance dispersal to the Hawaiian Islands, whether from another Pacific archipelago or from a continental source, remains an enduring enigma for many insect groups with no obvious means of trans-ocean dispersal. Crawford (1918) envisioned storms as a means of dispersal, stating: “It is conceivable that once in several million years a windstorm might have carried a leaf with galls containing nymphal psyllids and dropped the leaf in an Hawaiian forest of the same kind of trees – an exceedingly rare chance! – whereupon the insect might establish itself”. This dispersal scenario seems implausible for immatures as a leaf separated from a plant would soon desiccate resulting in mortality of developing immatures. However, such storms may have delivered adult psyllids to these remote islands; weather system dispersal has been proposed for other organisms, including Metrosideros (Wright et al. 2001, Gillespie et al. 2012).

### Does Pariaconus diversification parallel Metrosideros?

Some aspects of Pariaconus diversification suggest parallel and sequential evolutionary progression down the island chain that may mirror patterns found in Metrosideros (Percy et al. 2008). One parallel pattern in plant and insects is the basal position of the oldest island of Kauai and higher levels of divergence on this older island versus groups restricted to younger islands. Although there is no evidence for greater molecular divergence on Kauai, there is greater morphological diversity with some taxa more clearly differentiated. Kauai may also have the broadest range of galling habits, though interestingly, the younger island of Hawaii has a similarly high diversity. It should be noted that overall conclusions are limited by the relatively poor sampling on Kauai. Other similarities between diversification patterns of Metrosideros and Pariaconus lineages include the degree of within island ecological and geographic variation (e.g. bog and forest plant morphotypes, and bog and forest psyllid species around Alakai swamp on Kauai); distinct variation found between Waianae and Koolau Mountain ranges on Oahu and between east and west Maui; and the complex patterns, including apparent incipient diversity, on younger islands. These are all suggestive of parallel diversity in plant and insect lineages, which may be an important evolutionary process in this system.

**Figure 4.**
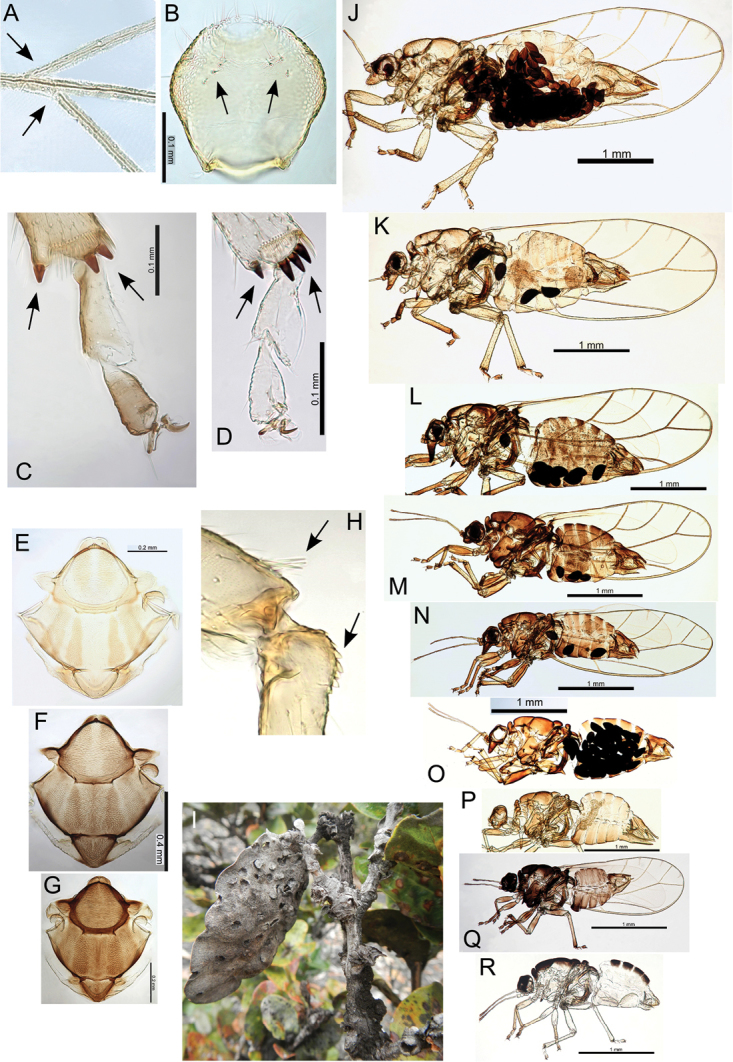
Examples of variability in Pariaconus adults, and an example of severe host damage. **A** fore wing detail showing a common trait variation with R branching slightly anterior of M and Cu_1_ bifurcation (Pariaconus pele) **B** posterior surface of male proctiger showing setae on interior of each lobe (Pariaconus pyramidalis) **C** typical 1+2 arrangement of sclerotized apical metatibia spurs, the single spur typically more stalked in ohialoha group (Pariaconus molokaiensis) **D** common variation of 1+3 arrangement of sclerotized apical metatibia spurs, the single spur typically less stalked in bicoloratus group (Pariaconus namaka) **E–G** thorax (dorsal view): **E** Pariaconus hawaiiensis **F** Pariaconus ohiacola **G** Pariaconus dorsostriatus **H** metafemur with several stout setae apically, and basal metatibia with cluster of genual spines (Pariaconus molokaiensis) **I** example of severe damage to a host plant with local necrosis caused by two closed gallers: Pariaconus pele (galls on leaf) and Pariaconus hawaiiensis (galls on stem and petiole) (Kona Hema, Hawaii) **J–R** adult size variation (females): **J–O** ohialoha group (abdomens with dark coloured eggs), **P** kamua group, **Q** minutus group, **R** bicoloratus group, **J** Pariaconus oahuensis (enclosed galls on stem/bud/leaf cones) **K** Pariaconus hawaiiensis (enclosed galls on stem/bud) **L** Pariaconus mauiensis (unknown gall biology) **M** Pariaconus montgomeri (enclosed flat gall on leaf) **N** Pariaconus ohiacola (enclosed flat gall on leaf) **O** Pariaconus pele (enclosed flat gall on leaf) **P** Pariaconus caulicalix (open cup gall on stem) **Q** Pariaconus gibbosus (unconfirmed, likely open pit gall on leaf) **R** Pariaconus hina (abdomen with pale eggs) (unconfirmed, likely free-living).

There still remain many questions regarding the underlying mechanisms responsible for morphological variation in Metrosideros polymorpha, despite genetic studies using both nuclear and chloroplast data, and genotype-ecotype analyses (Percy et al. 2008, Harbaugh et al. 2009, Wright and Ranker 2010, Stacy et al. 2014, 2016). It is likely that the patterns of diversity seen in the psyllid lineage, including species specific preference for one or another host morphotype, are driven in part by the varied phenotypic landscape of the host plant. But whether this effect is unidirectional (i.e. host plant effect on psyllids), or whether the impact and abundance of psyllids could influence shifts in the phenotypic landscape of Metrosideros polymorpha (e.g. via frequency dependent selection; Garrido et al. 2016) is an intriguing question. Certainly, the extremely heavy herbivore load on some individual plants caused by one or more Pariaconus species and resulting in tissue necrosis and leaf or bud abscission (Nishida et al. 1980, Gruner et al. 2005) (Fig. 4I), could impact plant fitness and/or reproductive success and thereby influence local ratios of different morphotypes (Bagchi et al. 2014). Plants have evolved a diverse array of anti-herbivory traits, including the evolution of specific defences to specific herbivores (Futuyma and Agrawal 2009), and an increasing number of studies show alternate phenotypes in plants can be driven by interactions with herbivores, such as defensive chemicals or leaf trichomes occurring as a result of herbivory (Agrawal et al. 2012, Hare 2012). In addition, there are a number of studies that have looked at the mechanistic control of alternate phenotypes, for instance via balancing selection and epigenetic functions (Bräutigam et al. 2013, Delph and Kelly 2014, Quintana-Murci 2016). The interplay of plant-insect interactions across the ecotypic-genotypic landscape of the Metrosideros-Pariaconus system could be a new model system that is only just starting to be investigated (e.g. Bailey et al. 2015).

### Intra-island divergence in Pariaconus

There are many examples suggesting a strong role for localized isolation by distance in Pariaconus populations, implying limited and localised dispersal within islands. However, it is also possible that migration rates may be higher than observed, but involve a high likelihood of failure to establish within already well established populations (Nosil et al. 2005), which would reinforce population structure and promote local adaptation. High levels of localized population structure on small geographic scales, such as we see in Pariaconus, is not unusual for specialist herbivores versus generalist predators (Rominger et al. 2015), and altitudinal clines in Pariaconus species distributions are partly linked to distributions of preferred host morphotypes (Nishida et al. 1980, Gruner et al. 2005). Even within the same host morphotype, local variation in occurrence and abundance of Pariaconus species is evident where individual Metrosideros plants bear many more galls than neighbouring plants. There are many factors that could influence local insect-plant interactions at this scale (Nishida et al. 1980, Gruner et al. 2005, Stacy et al. 2016), including stochastic processes of ecological turnover, particularly on younger, geologically volatile islands, which may disproportionately impact specialist taxa (Gruner et al. 2007, Hardy and Otto 2014), and contribute to both irregular distributions and rarity. One factor that may explain the mosaic patterns of distribution, at least for galling Pariaconus species, is intraspecific facilitation, whereby gall maker aggregations act as physiological sinks for photosynthate originating elsewhere in the plant (Heard and Buchanan 1998), competition thereafter may promote the utilization of different plant parts (Joy and Crespi 2007) and thus ultimately explain the ecological packing of multiple psyllid species on individual plants. Under this scenario, ecological packing results where tradeoffs are balanced between a) host sharing of favourably modified/induced plant phenotypes, and b) within plant spatial competition. It is possible that the absence of induced resource aggregating effects by the free-living taxa may explain why these species appear to be rarer and more scattered in distribution, although free-living species also frequently co-occur.

Complex patterns of diversification observed on the youngest island of Hawaii (e.g. “bicoloratus species complex”) can also be found in a number of other invertebrate groups (e.g. Wessel et al. 2013). One factor that likely plays an important role in promoting species complexes is the instability and fluctuation in the geological landscape on Hawaii, with ongoing volcanic activity and shifting habitats (Goodman et al 2012). What Futuyma (2010) calls “ephemeral divergence”, where localized spatial and temporal divergence may be relatively short lived and unstable population structure prevents longer term adaptive signatures becoming established, could be contributing to the disruption of species boundaries and/or maintenance of within species complexes (e.g. Pariaconus wyvernus and Pariaconus pele). However, similarly complex patterns are observable on most of the older islands that are less volatile geologically (e.g. in Pariaconus ohiacola and Pariaconus oahuensis on Oahu), thus requiring a broader hypothesis to explain the morphological variability evident within taxa. Crawford (1918) also described notable intraspecific variation in several species, some of which he maintained were incipient species, such as Trioza lanaiensis, which he considered to be “an incipient species not yet clearly marked from [Pariaconus oahuensis]”.

A comparison of the patterns of local divergence in Pariaconus with two other Pacific-wide “tramp” species sampled as outgroups: Leptynoptera sulfurea Crawford, 1919 (Triozidae) and Mesohomotoma hibisci (Froggatt, 1901) (Carsidaridae), highlights how different historical processes can generate marked differences in genetic-geographic structure in psyllids. These Pacific-wide psyllid species feed on widespread coastal host plants, Calophyllum inophyllum (Clusiaceae) and Hibiscus tiliaceus (Malvaceae) respectively, and although neither psyllid is native to the Hawaiian Islands, they do have wide native distributions reflecting the wide distribution of their host plants from Asia across the Pacific. The data presented here confirm that specimens of Leptynoptera sulfurea from Taiwan, Singapore, New Caledonia, and French Polynesia have virtually no genetic divergence, whereas specimens of Mesohomotoma hibisci from Singapore, New Caledonia, Norfolk Island, and French Polynesia exhibit considerably greater divergence suggestive of stronger isolation by distance. The geographically wide distributions of both plants are considered native, but anthropogenic influences can not be ruled out, particularly in the case of Calophyllum inophyllum, and therefore it is difficult to interpret the differences observed, but these examples nevertheless serve to illustrate that a similar breadth of geographic range may be accompanied by very different genetic structure.

### Parallel diversification in Pariaconus

The diversity in Pariaconus is striking considering speciation is not associated with shifts to different plant species, but rather shifts to different biological niches on a single plant species: galling to non-galling, different gall structures and placement of galls on the plant. Crawford (1925) was the first to report rearing adults from galls, and thereby identified species making enclosed galls on leaves as different from those making enclosed galls on the same plants but on stems and buds. Even more striking are characters associated with apparently independent parallel shifts to the same galling habit, and these convergences have led to much taxonomic confusion. An example is the large sized, yellow-green species that gall stems and buds on all major islands (Kauai, Oahu, Maui, and Hawaii). These were previously considered to be the same species or sister taxa. However, in each case where sister taxon relationships are well supported, they are more closely related to a species on the same island with a different galling habit, often a smaller, dark coloured species galling leaves. These shifts within islands appear key to understanding diversification in this system.

An ancestral pit-galling habit may have acted as an evolutionary springboard to both other galling and non-galling habits, with potentially multiple shifts to closed galling or free-living biologies. Independent shifts to closed galling biologies in the kamua and ohialoha groups has resulted in similar changes in immature morphology and chaetotaxy. Among the closed gallers there is evidence of numerous minor parallel evolutionary shifts, and even within species there is some galling lability. In a number of cases, species that predominantly exhibit one galling habit (stem gall, flat leaf gall, cone leaf gall), were found on at least three islands (Kauai, Oahu, Hawaii) to exhibit some galling lability (leaf gallers were galling stems and vice versa). In each of these cases, gall phenotype is faithful to gall position, and the identity of the galler was only detected by DNA sequencing immatures removed from galls. The apparently complex interaction between gall shifts and speciation among these closed gallers reinforces the importance of understanding the “interactome” or “cecidome” (Nabity 2016), in other words the insect-plant-gene interactions and factors influencing plant responses (Nabity et al. 2013, Bailey et al. 2015). In one example on Oahu, the predominantly stem/bud galler (Pariaconus oahuensis) produces cone leaf galls in a local population in the northern Koolau Mountains with similar gall structure (except for a different gall opening mechanism) to the cone leaf galler from Hawaii (Pariaconus pyramidalis) (compare cone galls in Fig. 50 and Fig. 52), yet these species are not sister taxa; interestingly, on the older island of Oahu it appears to be a more recent and population level shift (from stem galling to galling leaves); whereas on the younger island of Hawaii, the shift is older resulting in a divergent sister taxon from the stem galler (Pariaconus hawaiiensis) that produces cone leaf galls (Pariaconus pyramidalis). Thus, what likely began with similar local shifts, progressed further on Hawaii to produce two distinct sister taxa. What factors, other than time, promote the evolutionary progression to complete reproductive isolation remains unknown. These examples provide discrete parallel evolutionary systems with which to investigate plant gall shifts. In addition, local lability in galling habit does mean that using gall type alone for identification of taxa (particularly in the ohialoha species group) can be problematic.

As mentioned above, stem/bud gallers have generally larger body size and paler body colour than leaf gallers in the ohialoha group, but there are no other obvious macromorphological traits that are specifically associated with the shifts between leaf and stem galling habits. Variation in ovipositor size between related stem- and leaf-galling cecidomyid flies is associated with placement of eggs into plant tissues and deeper implantation on stems (Joy and Crespi 2007), but similarly marked ovipositor differences between pairs of stem- and leaf-galling sister taxa in Pariaconus is unlikely because eggs are deposited on the plant surface, and it is not until 1^st^ instar feeding commences that gall development, first as a pit gall, is initiated, with completed gall enclosure by the 2^nd^ instar. This generational progression, from 1st instars in shallow pits before gall enclosure, is also evident on examination of the 1^st^ instar exuviae, which have typical pit galler morphology (i.e. marginal sectasetae), and can often be found inside closed galls with extant 2^nd^ instars which no longer retain those traits. It is therefore apparent that galls result in response to psyllid feeding, rather than to oviposition, and preliminary work on the transcriptional landscape of galls using dual psyllid and plant RNA sequence analysis suggests Pariaconus taxa may influence gall development by synthesizing the plant growth hormone auxin (Bailey et al. 2015).

Because gall position is one of the factors determining gall structure, maternal selection of an oviposition site will be important in determining gall type and potentially promoting diversification (Janz et al. 2005, Kato et al. 2010). The cues influencing oviposition site selection may be a critical factor determining rates and direction of diversification in Pariaconus, and oviposition “mistakes” may be an important route to repeated parallel shifts to different plant organs (Joy and Crespi 2007), as well as preference for particular Metrosideros morphotypes (Gruner et al. 2005). Notably, the highly variable egg morphologies remain unexplained, but appear to be relatively phylogenetically conserved, in contrast to homoplasy in overall body size/colour. Further investigation of the more subtle shifts in egg morphology and biology within species (e.g. Pariaconus oahuensis) is needed to fully understand the cause and effect of the unusual range of egg types in Pariaconus.

## Conclusions

Species diversity in Pariaconus provides a unique example of a psyllid radiation on a single, highly polymorphic host plant. The extraordinary diversity of biologies and morphology found in Pariaconus have emerged within the geological period of the current high islands of the Hawaiian chain, and diversification of psyllid and host plant lineages have occurred within a similar time frame. This raises many questions for future investigation regarding patterns of parallel and convergent evolution, and ecotype-genotype interactions between plant and insect systems over time. Extensive and focused study using a variety of molecular approaches will be needed to explore and understand the complex evolutionary processes in Pariaconus. In this study, the basic patterns of variation in this fascinating group are presented in order to provide a baseline for future investigations.

## Taxonomic treatment

### Triozidae Löw, 1879

#### Pariaconus

Taxon classificationAnimaliaHemipteraTriozidae

Enderlein, 1926

Trioza Foerster, 1848: 82, in part.
Kuwayama Crawford, 1911: 503, in part.
Pariaconus Enderlein, 1926: 401. Type species: Kuwayama nigricapita Crawford, 1918, by original designation.

##### Adult colour and structure.

General body colour either entirely dark (black, brown, or red), entirely or mostly pale (cream, yellow or orange), or distinctly bicolored (pale/dark) (e.g. Fig. 4R). Overall size variable from ~1.5–4.5 mm in length (Fig. 4E–G, J–R). Fore wing broadest either in the middle or in the apical third, membrane with or without distinct pattern of pigmentation, if without pattern either clear or fuscous (whiteish opaque in newly emerged adults); veins brown and either with trifurcation of R, M and Cu_1_, or vein R branching slightly anterior (Fig. 4A, this character can vary within populations and even between left and right wings of individuals and is not considered diagnostic for species); vein Rs relatively short, reaching fore wing margin at or proximal to M fork; long to minute setae on fore wing margins and veins; fore wing membrane either with spinules distributed densely throughout all cells or sparsely distributed and limited to a few cells; a cluster of marginal radular spines present in cells cu_1_, m_1_ and m_2_; fore wing apices either acute, bluntly acute, or rounded. Head moderately deflexed downwards, vertex more or less flat dorsally, with lateral ocelli lying on small tubercles, medial epicranial suture distinct; genal processes extremely short to long, and either concolorous, darker, or lighter than general body colour. Antennae short to long; antennal segments 10, with terminal 3(-6) segments usually darker; a single rhinaria apically on segments 4, 6, 8, 9; terminal segment with two unequal length setae. Distal proboscis segment short to long, darker apically. Thorax moderately arched. Minute to long setae on dorsum of vertex and thorax. Legs moderately short and robust to long and slender, tibia longer than femur; hind leg with meracanthus well developed and straight; metafemur with several stout setae apically; metatibia with a cluster of genual spines basally and typically 1+2 (occasionally a single or 1+3) sclerotized apical spurs and a comb of stout unsclerotized setae; tarsi subequal in length (Fig. 4C–D, H). Male terminalia with more or less rounded subgenital plate; proctiger with moderate or more pronounced posterior lobe medially (interior surface of lobes bearing 4–5 simple setae, Fig. 4B), length shorter, subequal or longer than paramere; paramere variable; distal aedeagus segment apex either hooked or blunt. Female terminalia with proctiger short or long, dorsal surface straight, convex, or medially concave, subequal, shorter or longer than the subgenital plate, long dorsal setae, a simple anal ring composed of a continuous or interrupted double row of cells, and apex acute to bluntly acute; subgenital plate ventral surface either concave or convex, medium to long ventral setae, apex acute to bluntly acute or truncate; ovipositor either with or without serrations. Eggs highly variable, broadly ovoid or slender, pale or dark, with or without microsculpturing and striations, and with or without distinct pedicel and tail.

##### Immatures and biology.

Extremely variable immature morphology reflects variation in biologies and galling habits. Immatures may be free-living or gall forming (open and closed galls). Galling species appear to have one immature per gall/gall chamber, but in some galling species dense aggregations of galls result in clusters of chambers in close proximity. Morphologically consistent characters for 5^th^ instars are antennae with 8-9 segments bearing 4 rhinaria (on segments 4, 6, 8 and 9, or 2 on segment 8) and two short to medium long terminal simple setae, tarsi with broad crescent arolia and each terminal tarsus bearing a long simple or weakly capitate seta, and anus situated ventrally.

##### Host plant.

The host plant of all Pariaconus species is considered to be Metrosideros polymorpha (Myrtaceae). However, there are five described species of Metrosideros in the Hawaiian Islands, and Pariaconus species and galls have occasionally been found on other Metrosideros taxa (e.g. Metrosideros macropus, Metrosideros rugosa, and Metrosideros waialealae) (see Table 2), but in all cases, these Pariaconus species are predominantly on Metrosideros polymorpha. Extensive interspecies gene flow among Hawaiian Metrosideros suggests taxonomic concepts in Metrosideros may not reflect discrete genotypic or phenotypic units; the extensive morphotypic variation in Metrosideros polymorpha is therefore considered more influential in driving divergence than occurrence on different species of Metrosideros. Nevertheless, more detailed examination of population divergence in Pariaconus species found on multiple Metrosideros species remains to be undertaken.

##### Comments.

Enderlein (1926) erected this genus in order to rectify, as he saw it, the incorrect inclusion of three Hawaiian species (Pariaconus nigricapitus, Pariaconus minutus, Pariaconus gracilis) by Crawford (1918) in the predominantly new world genus Kuwayama based on the absence of genal processes (also referred to as genal cones or genae); Enderlein named this new genus Pariaconus, with the intention of highlighting this homoplasy: ‘similarly without cones’, yet different from Kuwayama. The name Pariaconus has therefore existed since 1926 as the nomenclaturally correct name for these three Hawaiian taxa, but Pariaconus was not used in subsequent publications (e.g. Zimmerman 1948, Swezey 1954, Nishida et al. 1980), and the use of Kuwayama persisted until noted in a revisionary classification of the Psylloidea by Burckhardt and Ouvrard (2012).

The original placement of some taxa in Kuwayama by Crawford (who also originally described the genus Kuwayama) was done with acknowledged reservations; characters used to define Kuwayama, such as the absence of genal processes (but with swellings below the bases of the antennae), enlarged clypeus, thorax as broad or broader than the head, and wing subacute to acute, are either not found at all, or not consistently found in Pariaconus. The reduced genal processes that are characteristic of the bicoloratus and minutus species groups are usually still visible between the bases of the antennae, and there are no distinct swellings below the antennae. Furthermore, the development of the genal processes in Pariaconus is highly variable and can even vary considerably within species (notably Pariaconus gracilis, Pariaconus oahuensis, Pariaconus ohiacola, and Pariaconus pele). Diminutive genal processes are considered the ancestral condition based on data presented here, with development of longer genal processes in more recently derived species (e.g. the ohialoha group).

Pariaconus is a monophyletic genus endemic to the Hawaiian Islands. Four species groups within Pariaconus are recognized: the bicoloratus, minutus, kamua, and ohialoha groups. The taxa are morphologically remarkably diverse, making ancestral outgroup affiliations difficult to interpret, but they are neither allied to the type species of the genus Trioza, nor to Kuwayama. Nor are those members of Pariaconus that were originally assigned to Kuwayama by Crawford (1918) related to other Hawaiian taxa currently placed in Kuwayama that feed on Pisonia (Nyctaginaceae) and Sideroxylon (Sapotaceae) (Caldwell 1940, Uchida and Beardsley 1992), nor are they related to other Hawaiian genera feeding on other plant families in the Hawaiian Islands. The Pariaconus species are the only psyllids that feed on the family Myrtaceae in the Hawaiian Islands, but they are not closely related to other Myrtaceae-feeding taxa in the Pacific or Australasia.

The original placement of some of the Pariaconus species in Trioza reflects the use of the genus Trioza as a default placement for triozid taxa with no clear affiliations. The width of the head in Pariaconus is typically greater (bicoloratus and minutus groups) than that of the thorax, or subequal (kamua and ohialoha groups), but the fore wings do not have the long sinuous Rs vein present in the type species of Trioza, Trioza urticae (Linné, 1758), and Kuwayama, Kuwayama medicaginis (Crawford, 1910). The basal metatibial spur arrangement is typically 2+1 in Pariaconus, which is the same as Kuwayama medicaginis, but differs from the 3+1 in Trioza urticae. However, this character, normally considered fixed and diagnostic of species or even genera, can be variable within populations in Pariaconus (Fig. 4C–D), with one population of Pariaconus hawaiiensis including individuals with a single spur, or 2+1, or 3+1. Similar variation was noted by Crawford (1918) for Pariaconus oahuensis and Pariaconus lanaiensis, see also note on this character in relation to Trioza eugeniae and Trioza adventicia.

In this study, broad species concepts are combined with the recognition of morphological forms (infrasubspecific names as per ICZN) to convey the extent and distribution of morphological variation. Some forms recognized here may warrant subsequent recognition at species level.

##### Adult key to Pariaconus species groups

**Table:** 

1	On Kauai	**See key to kamua species group**
–	On other islands	**2**
2	Generally smaller species (often bicolored cream and black, or entirely black, or entirely pale) with short genal processes (ratio VL:GP >2), antennal length less than 1 mm (ratio AL:HW ≤1.6), female anal ring relatively large (ratio FP:RL usually <3.5), eggs more narrow and elongate (ratio EL:EW usually ≥2) and typically unpigmented or light brown	**See key to bicoloratus and minutus species groups**
–	Generally larger species (often red-brown or yellow-green), genal processes either long (ratio VL:GP <2) or if shorter (ratio VL:GP >2) (e.g. Pariaconus oahuensis, Pariaconus ohiacola, Pariaconus montgomeri, Pariaconus pyramidalis), antennal length usually longer than 1 mm (ratio AL:HW >1.6), female anal ring relatively small (ratio FP:RL usually >3.5), eggs more broad and ovoid (ratio EL:EW usually <2) and typically mid- to dark brown	**See key to ohialoha species group**

##### Adult key to Pariaconus species in the bicoloratus and minutus species groups (found on Oahu, Molokai, Maui, Hawaii)

**Table:** 

1	Male with paramere shape triangular, broad at base and tapering to narrow apex, female subgenital plate truncate apically, ovipositor valvulae dorsalis strongly convex dorsally	**2**
–	Male with paramere more or less parallel sided before constricting below apex, or tapering to apex, female subgenital plate not truncate either acute or bluntly acute, ovipositor valvulae dorsalis typically moderately or not convex dorsally, rarely strongly convex dorsally (e.g. Pariaconus gibbosus)	**6**
2	Fore wing narrow (ratio WL:WW >2.60) with apex bluntly acute, shape of male and female terminalia as in Fig. 14 (on Oahu)	**Pariaconus liliha sp. n.**
–	Fore wing broader (ratio WL:WW <2.60) with apex typically rounded, rarely bluntly acute (on Molokai, Lanai, Maui, Hawaii)	**3**
3	Male with longer paramere (PL ≥0.16 mm, ratio MP:PL <1.15), shape of male and female terminalia as in Fig. 8 (on Hawaii)	**Pariaconus wyvernus sp. n.**
–	Male with shorter paramere (PL ≤0.16 mm, ratio MP:PL >1.15)	**4**
4	Male with longer distal aedeagus segment (AEL ≥0.17 mm, ratio PL:AEL <0.80), shape of male and female terminalia as in Fig. 12 (on Hawaii)	**Pariaconus poliahu sp. n.**
–	Male with shorter distal aedeagus segment (AEL <0.17 mm, ratio PL:AEL >0.80)	**5**
5	Male with paramere length less than 0.80 height of subgenital plate, shape of male and female terminalia as in Fig. 7 (on Molokai, Maui)	**Pariaconus hina sp. n.**
–	Male with paramere length greater than 0.80 height of subgenital plate, shape of male and female terminalia as in Fig. 5 (on Molokai, Lanai, Hawaii)	**Pariaconus nigricapitus (Crawford, 1918)**
6	Female terminalia with relatively small anal ring (ratio FP:RL >3.20), male paramere (only known for Pariaconus lona) apex with bipartite sclerotization and length >0.15 mm (as in Fig. 13)	**7**
–	Female terminalia with relatively large anal ring (ratio FP:RL <3.20), male paramere apex typically terminating in hook, if with bipartite sclerotization then paramere length <0.15 mm (e.g. Pariaconus gracilis)	**9**
7	Larger species (WL >2.20 mm, HW >0.55 mm), genae more developed, female terminalia longer (FP >0.60 mm), shape of female terminalia as in Fig. 10 (on Hawaii)	**Pariaconus kapo sp. n.**
–	Smaller species (WL <2.20 mm, HW <0.55 mm), genae less developed, female terminalia shorter (FP <0.60 mm)	**8**
8	Antenna longer (>0.70 mm), female proctiger longer (FP >0.50 mm), shape of male and female terminalia as in Fig. 13 (on Molokai)	**Pariaconus lona sp. n.**
–	Antenna shorter (<0.70 mm), female proctiger shorter (FP <0.50 mm), shape of female terminalia as in Fig. 9 (on Hawaii)	**Pariaconus nigrilineatus sp. n.**
9	Length of female proctiger and subgenital plate subequal (ratio FP:SP <1.05), male with shorter distal aedeagus segment (ratio PL:AEL ≥1.15)	**10**
	Female proctiger longer than subgenital plate (ratio FP:SP >1.05), male with longer distal aedeagus segment (ratio PL:AEL <1.15)	**11**
10	Fore wing narrower (WL:WW >2.65) and more acute apically, genae more acute, shape of male and female terminalia as in Fig. 18 (makes pit galls) (on Oahu)	**Pariaconus namaka sp. n.**
–	Fore wing broader (WL:WW <2.65) and more rounded apically, genae more rounded, shape of male and female terminalia as in Fig. 17 (makes pit galls) (on Hawaii)	**Pariaconus dorsostriatus sp. n.**
11	Long distal proboscis segment (PB >0.11 mm), female terminalia long (FP >0.50 mm) with proctiger longer than head width (ratio FP:HW >1), antennae relatively long (>0.60 mm, ratio AL:HW >1.30), male proctiger and paramere typically longer (MP ≥0.18, PL ≥0.17), shape of male and female terminalia as in Fig. 11 (free-living immatures) (on Hawaii)	**Pariaconus proboscideus sp. n.**
–	Shorter distal proboscis segment (PB ≤0.11 mm), female terminalia shorter (FP <0.50 mm) with proctiger shorter than head width (ratio FP:HW <1), antennae relatively short (<0.60 mm, ratio AL:HW <1.30), male proctiger and paramere typically shorter (MP <0.18, PL ≤0.17)	**12**
12	Male with short paramere (ratios PL:SH <0.80 and PL:HW <0.30) with bipartite sclerotization at apex, relatively short hind tibiae (ratio HW:HT >1.15), female proctiger <0.75 × head width, egg with surface striations, shape of male and female terminalia as in Fig. 16 (free-living immatures) (on Oahu, Molokai, Maui)	**Pariaconus gracilis (Crawford, 1918)**
–	Male with longer paramere (ratios PL:SH >0.80 and PL:HW >0.30) with hook at apex, and relatively long hind tibiae (ratio HW:HT <1.15), female proctiger ≥0.75× head width, egg without surface striations	**13**
13	Male with paramere apical hook inward pointing, longer distal aedeagus segment (ratio PL:AEL <0.95) with well developed hook, female proctiger shorter (ratios FP:RL <2.55 and FP:HW <0.80), shape of male and female terminalia as in Fig. 20 (on Maui)	**Pariaconus gibbosus sp. n.**
–	Male with paramere apical hook upward pointing, shorter distal aedeagus segment (ratio PL:AEL >0.95) with apex not hooked, female proctiger longer (ratios FP:RL ≥2.55 and FP:HW >0.80), shape of male and female terminalia as in Fig. 19 (makes pit galls) (on Hawaii)	**Pariaconus minutus (Crawford, 1918)**

#### bicoloratus species group

A group of mostly diminutive species, though the largest are similar in size to the smaller members of other groups (Fig. 4). This group includes two of the taxa previously placed in Kuwayama (Pariaconus nigricapitus, Pariaconus gracilis). The immatures develop either in open galls as shallow pits on the leaf surface, or are free-living on the leaf surface. The adults are characterized by reduced genal processes, short antennae, and often distinct bicoloration, with combinations of dark and pale colouration: usually brown/black with yellow/cream, or with individuals entirely dark or entirely pale. Immature morphology is extremely variable. The eggs are typically slender and either with distinct striations and ridges present or entirely lacking, and either with or without a short, laterally positioned pedicel. Currently the greatest diversity of extant species is found on Hawaii, particularly the Kohala region. Subfossils from Kauai and two recently discovered species on Oahu suggest that this group may have been much more diverse on older islands.

##### Pariaconus nigricapitus

Taxon classificationAnimaliaHemipteraTriozidae

(Crawford, 1918)

Figures 5 , 45A–E

Kuwayama nigricapita Crawford, 1918: 446.
Kuwayama nigrocapita wrong spelling of Kuwayama nigricapita Crawford in Crawford (1925): 424.
Pariaconus nigricapitus (Crawford), Enderlein (1926): 401; Burckhardt and Ouvrard (2012): 22.
Pariaconus nigricapatus wrong spelling of Pariaconus nigricapitus (Crawford) in Enderlein (1926): 401.

###### Adult colour.

Typically bicoloured, generally pale cream-yellow to green on thorax and abdomen, head darker, with a dark dorsal stripe from the head extending part or all the length of the body. Fore wing membrane clear or slightly fuscous.

###### Adult structure.

**Figure 5.**
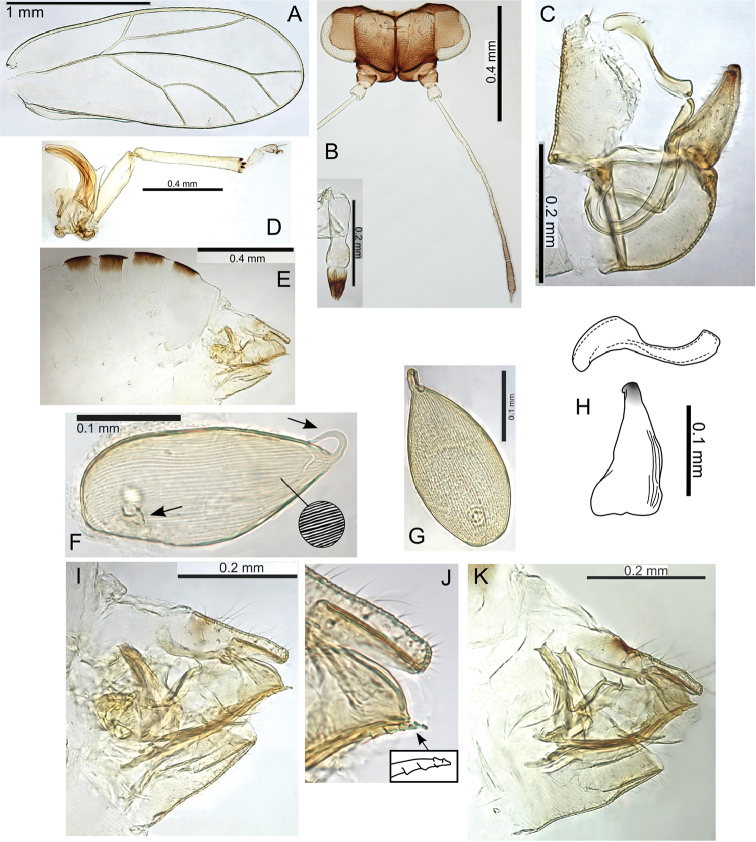
Pariaconus nigricapitus. **A** fore wing **B** head with antenna, and proboscis (inset) **C** male terminalia **D** hind leg **E** female abdomen and terminalia (holotype) **F, G** eggs (pedicel, tail, and striations indicated; holotype) **H** aedeagus and paramere **I** female terminalia (holotype) **J** ovipositor (serrations indicated) **K** female terminalia (Olaa, Hawaii).

Fore wing apex rounded; surface spinules dispersed, usually in all cells but may be reduced or absent in r_1_ and c+sc; setae on margins and veins short to minute (Fig. 5A). Antennae short (av. length 0.68; ratio AL:HW av. 1.38); genal processes extremely short (ratio VL:GP av. 4.81); short to minute setae on vertex and thorax; distal proboscis segment short (av. length 0.07); hind tibia subequal to head width (ratio HW:HT av. 1.03) (Fig. 5B, D). Male terminalia (Fig. 5C, H): paramere shorter than proctiger (ratio MP:PL 1.20), broad at base, tapering to narrow neck below apex with short interiorly directed hook; distal aedeagus segment length subequal to paramere (ratio PL:AEL 1.00), base slightly angular and moderately inflated, apex developed into a dorsally flattened, bluntly rounded hook (ratio AEL:AELH 2.00). Female terminalia (Fig. 5E, I-K): proctiger dorsal surface more or less straight, apex blunt, longer than subgenital plate (ratio FP:FSP av. 1.41), anal ring long (ratio FP:RL av. 2.20); subgenital plate with no or slight medial bulge ventrally, apex truncate; ovipositor apex with serrations (3 pronounced lower, and 2 pronounced upper), valvulae dorsalis strongly convex dorsally.

###### Egg.

Unpigmented or light brown, broad and moderately long with uninterrupted striations over entire surface, short pedicel 1/4 length from base, tail moderately long (Fig. 5F–G).

###### Immature

(note: immature association for Pariaconus nigricapitus is based on immatures with the same collection data as the holotype, but given the co-occurrence of taxa, this remains to be confirmed with DNA analysis). Colour and structure 5^th^ instar: Mid to dark brown. Broadly ovoid in outline with more or less uninterrupted circumference, and the entire dorsal surface raised into a sclerotized dome resulting in a smooth lacquer-like casing (Fig. 45A–C, E). Fore wing buds with distinct humeral lobes. Tarsi with small reduced claws (Fig. 45D). Circumanal ring broad and shallowly v-shaped, with a single row of elongate cells (Fig. 45A). Four protruding mounds of tissue each terminating in a cluster of distinctly enlarged cells are situated ventro-anterior to the meso and meta coxae (Fig. 45A; also present in open gallers, e.g. Fig. 48N), similar structures were found in the same position on Pariaconus minutus instars within pit galls, and these likely aid either in attachment to the plant surface, or in positional shifting within a gall but are usually no longer visible after preparation for slide mounting and so are illustrated here for the first time using the imprint left in the casing of Pariaconus nigricapitus. Chaetotaxy: The entire outer margin is ringed with fused setae resembling reduced sectasetae (Fig. 45A). Dorsum without setae, or with sparse minute simple setae.

###### Host plant notes.

Unconfirmed, but may prefer more glabrous morphotypes.

###### Island.

Molokai, Lanai, Hawaii.

###### Distribution notes.

Although treated broadly as occurring on three islands (based on previous records summarized in Zimmerman 1948), further sampling from Molokai and Lanai is required to confirm this distribution. Only two species from the bicoloratus group were collected on Molokai during this study (Pariaconus hina, Pariaconus lona).

###### Biology.

The immatures described here are considered free-living but develop under a domed casing which remains after the adult has enclosed (see note on need to confirm adult-immature association).

###### Comments.

Belongs to a complex of species within the bicoloratus group (“bicoloratus species complex” Figs 1–2) for which there is scant biological knowledge, but all the taxa may be non-galling. The species complex includes Pariaconus nigricapitus, Pariaconus hina, and Pariaconus wyvernus with notably truncate female genitalia, and three other species (Pariaconus nigrilineatus, Pariaconus proboscideus, Pariaconus kapo). All species appear to have a scattered distribution, occur at low abundance and are infrequently collected; immatures are rarely encountered, and the adult morphology is often cryptic (close examination of slide mounted specimens is required). A complete taxonomic concept of Pariaconus nigricapitus remains somewhat uncertain due to the fact that the type material consists of a single entire female, and a partial male specimen with the abdomen missing. Because female morphology is similar among three species in the species complex, the association of the type female specimen with conspecific male counterparts is challenging. Similarly, due to the sympatric co-occurrence of taxa in this species complex, the association of the immature described here, which was collected by O. Swezey in May 1917 with the adult type material of Pariaconus nigricapitus, remains to be confirmed.

###### Type material.

Holotype, female (slide mounted, BPBM). See Table 2 for details of type and other material examined for this study.

##### Pariaconus hina

Taxon classificationAnimaliaHemipteraTriozidae

Percy sp. n.

http://zoobank.org/F97011C2-83EF-419D-9D4E-7C8A337E9CC5

Figures 6 , 7

###### Adult colour.

**Figure 6.**
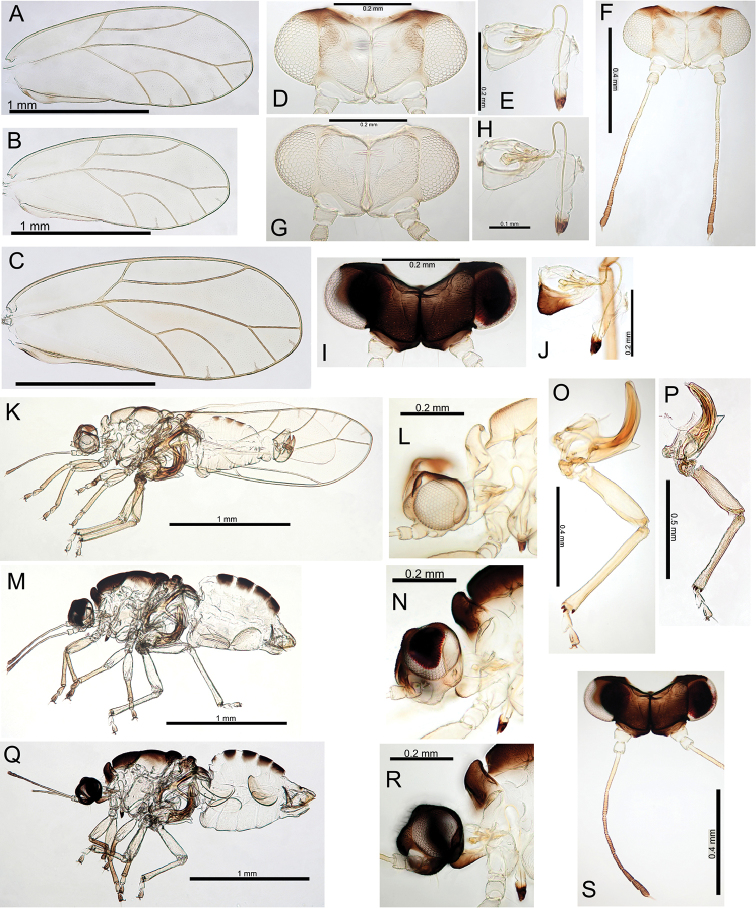
Pariaconus hina sp. n. **A, B, C** fore wing: **A** form ovostriatus **B** form occidentalis **C** form orientalis (female) **D, E, F** head, proboscis, head and antennae, form ovostriatus **G, H** head, proboscis, form occidentalis **I, J** head, proboscis, form orientalis (female) **K, L** male, form ovostriatus **O** hind leg, form ovostriatus **P** hind leg, form occidentalis **M, N** female, form ovostriatus **Q, R, S** female, head and antenna (female), form orientalis.

Typically bicoloured, generally pale cream-yellow to green on thorax and abdomen, head darker, with or without a dark dorsal stripe from the head extending part or all the length of the body (Fig. 6K, M, Q). Fore wing membrane slightly to moderately fuscous.

###### Adult structure.

**Figure 7.**
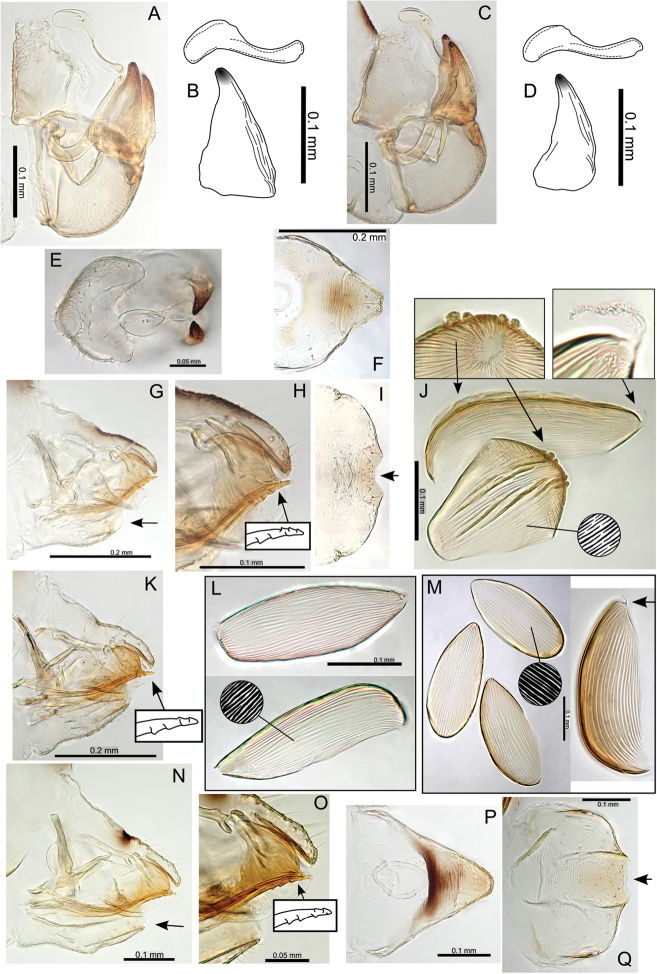
Pariaconus hina sp. n. **A, B**, male terminalia, aedeagus and paramere, form ovostriatus **C, D** male terminalia, aedeagus and paramere, form occidentalis **E, F, G, H, I, J** form ovostriatus: **E** male terminalia (dorsal view) **F** female proctiger (dorsal view) **G** female terminalia (truncate subgenital plate indicated) **H** ovipositor (serrations indicated) **I** female subgenital plate (ventral view, concave apex indicated) **J** eggs (tail and striations indicated) **K, L** form occidentalis: **K** female terminalia (ovipositor serrations indicated) **L** eggs (striations indicated) **M, N, O, P, Q** form orientalis: **M** female terminalia (truncate subgenital plate indicated) **O** ovipositor (serrations indicated) **P** female proctiger (dorsal view) **Q** female subgenital plate (ventral view, concave apex indicated).

Fore wing apex rounded to bluntly acute; surface spinules well dispersed, present in all cells but reduced coverage in r_1_ and c+sc; setae on margins and veins short to minute (Fig. 6A–C). Antennae short (av. length 0.67; ratio AL:HW av. 1.38); genal processes extremely short (ratio VL:GP av. 5.17); short to minute setae on vertex and thorax; vertex comparatively wide (ratio HW:VW av. 1.89); distal proboscis segment short (av. length 0.06); hind tibia subequal to head width (ratio HW:HT av. 1.03) (Fig. 6D–J, L, N–P, R–S). Male terminalia (Fig. 7A–E): paramere shorter than proctiger (ratio MP:PL av. 1.16), broad at base, tapering to narrow neck below apex with short, inward pointing hook; distal aedeagus segment length subequal to paramere (ratio PL:AEL av. 1.00), base slightly angular and moderately inflated, apex developed into a bluntly rounded hook (ratio AEL:AELH av. 2.04). Female terminalia (Fig. 7F–I, K, N–Q): proctiger dorsal surface more or less straight but moderately to strongly convex apically, apex bluntly rounded, longer than subgenital plate (ratio FP:FSP av. 1.49), anal ring long (ratio FP:RL av. 2.04); subgenital plate with slight to more pronounced medial bulge ventrally, apex truncate and concave (more pronounced in form ovostriatus and only slightly in form orientalis); ovipositor apex with serrations (3 pronounced lower, and either 3 pronounced upper in form ovostriatus, or 1-2 reduced upper in forms occidentalis and orientalis), valvulae dorsalis strongly convex dorsally.

###### Egg.

Unpigmented or light brown, long to moderately long with striations over entire surface, but in form ovostriatus these are more widely spaced and interrupted, with the addition of distinctly raised dorsal ridges, in forms occidentalis and orientalis they are uninterrupted; pedicel absent, tail long in form ovostriatus (and composed of a curious structure of nodules), short in form orientalis, and apparently lacking in form occidentalis (Fig. 7J, L–M).

###### Immature.

Unknown.

###### Host plant notes.

Morphotype preference unknown.

###### Island.

Molokai, Maui.

###### Distribution notes.

Of the three recognized forms, form ovostriatus and form orientalis are currently only known from east Maui, and form occidentalis is only known from west Maui. Only one, diminutive sized female from Molokai was collected, and the distribution and form on this island remain to be confirmed.

###### Biology.

Unknown.

###### Etymology.

Named after Hina, a goddess of the moon in Hawaiian mythology (noun in the nominative singular standing in apposition to the generic name).

###### Comments.

Three forms are recognized (Figs 6–7): form ovostriatus (based on the type has a broader paramere, female subgenital plate more truncate and more notably concave apically, and distinct dorsal ridges on the egg), form occidentalis (smallest form, with more slender paramere), and form orientalis (largest from), both the latter have finely striated eggs lacking dorsal ridges. These forms are highly divergent for molecular data (to the extent that there is no support for a monophyletic Pariaconus hina) (Fig. 3) but extremely similar morphologically and therefore may represent morphologically cryptic divergence.

###### Type material.

Holotype male (slide mounted, BMNH). See Table 2 for details of type and other material examined for this study.

##### Pariaconus wyvernus

Taxon classificationAnimaliaHemipteraTriozidae

Percy sp. n.

http://zoobank.org/419BF25F-6552-4CC0-A742-57701EDDB9FC

Figure 8

###### Adult colour.

**Figure 8.**
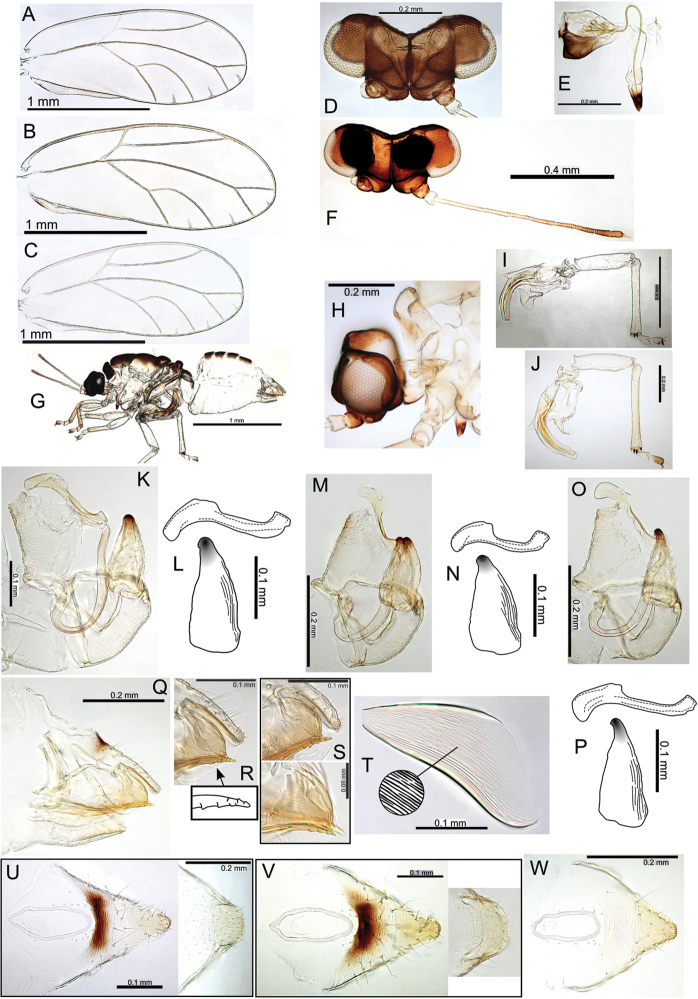
Pariaconus wyvernus sp. n. **A, B, C** fore wing: **A** form wyvernus **B** form chimera **C** form gorgonus **D, E** head, proboscis, form wyvernus **F** head and antenna (uncleared ocular tissue), form chimera **G** female, form wyvernus **H** male head, form wyvernus **I, J** hind legs: **I** form wyvernus **J** form gorgonus **K, L, M, N, O, P** male terminalia, aedeagus and paramere: **K, L** form wyvernus **M, N** form chimera **O, P** form gorgonus **Q** female terminalia, form wyvernus **R, S** ovipositors: **R** form wyvernus (serrations indicated) **S** form gorgonus (above), form chimera (below) **T** egg (striations indicated), form wyvernus **U, V, W** female proctigers and subgential plates: **U** form wyvernus **V** form chimera **W** form gorgonus.

Variable, often strikingly bicoloured with black or dark brown head and pale cream or yellow-green thorax and abdomen, with or without a dark dorsal stripe from the head extending part or all the length of the body, but can also be completely pale throughout (Fig. 8G). Fore wing membrane clear or fuscous.

###### Adult structure.

Fore wing apex rounded; surface spinules well dispersed in all cells but reduced or absent in r_1_ and c+sc; setae on margins and veins short to minute (Fig. 8A–C). Antennae short (av. length 0.71; ratio AL:HW av. 1.36); genal processes extremely short (ratio VL:GP av. 4.88); short to minute setae on vertex and thorax; distal proboscis segment short (av. length 0.07); hind tibia subequal to head width (ratio HW:HT av. 1.08) (Fig. 8D–F, H–J). Male terminalia (Fig. 8K–P): paramere length subequal to proctiger (ratio MP:PL av. 1.03), broad at base, tapering to anteriorly directed apex with short, interiorly directed hook; distal aedeagus segment length subequal to paramere (ratio PL:AEL av. 0.92), base angular and moderately inflated, apex developed into a dorsally flattened, bluntly rounded hook (ratio AEL:AELH av. 2.18). Female terminalia (Fig. 8Q–S, U–W): proctiger dorsal surface moderately to strongly convex apically, apex bluntly acute, longer than subgenital plate (ratio FP:FSP av. 1.46), anal ring long (ratio FP:RL av. 2.08); subgenital plate with slight medial bulge ventrally, apex truncate; ovipositor apex with serrations (2-3 upper and 3-4 lower), valvulae dorsalis strongly convex dorsally.

###### Egg.

(only known for form wyvernus) Unpigmented or light brown, large, with both continuous and interrupted striations over entire surface, apparently lacking pedicel and tail (Fig. 8T).

###### Immature.

Unknown.

###### Host plant notes.

Unconfirmed, but may prefer more glabrous morphotypes.

###### Island.

Hawaii.

###### Distribution notes.

All three forms are found in Kohala, with form wyvernus only known from this region.

###### Biology.

Unknown.

###### Etymology.

Named after “wyvern”, a mythical winged creature in Medieval mythology, in reference to the rarity and acknowledged taxonomic puzzle this taxon presents (noun in the nominative singular).

###### Comments.

Three forms are recognized (Fig. 8): form wyvernus (based on the type has a longer paramere and larger aedeagus hook), form chimera (larger form has the shortest paramere), and form gorgonus (longer, more slender tibiae, more slender paramere, and bulbous tip to aedeagus hook). The current genetic analyses suggest this taxon may actually be composed of two or more cryptic species that are polyphyletic. Further work is needed, particularly more sampling, to resolve this and therefore recognising this variation with forms is the best option at present.

###### Type material.

Holotype male (slide mounted, BMNH). See Table 2 for details of type and other material examined for this study.

##### Pariaconus nigrilineatus

Taxon classificationAnimaliaHemipteraTriozidae

Percy sp. n.

http://zoobank.org/051ECEA8-0E89-4EBA-A4C1-A30EAD88B32E

Figure 9

###### Adult colour.

**Figure 9.**
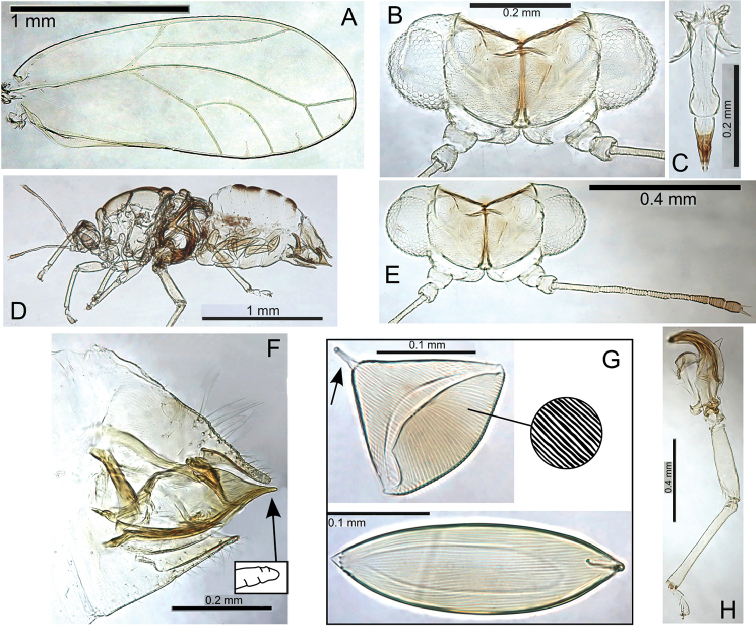
Pariaconus nigrilineatus sp. n. (female) **A** fore wing **B** head **C** proboscis **D** habitus **E** head and antenna **F** terminalia (ovipositor serrations indicated) **G** eggs (tail and striations indicated) **H** hind leg.

Typically bicoloured, generally pale cream-yellow thorax and abdomen, head darker and a dark dorsal stripe from the head extending part or all the length of the body (Fig. 9D). Fore wing membrane slightly fuscous.

###### Adult structure.

Fore wing apex rounded; surface spinules dispersed in all cells but reduced coverage in r_1_ and c+sc; setae on margins and veins short to minute (Fig. 9A). Antennae short (av. length 0.59; ratio AL:HW av. 1.15); genal processes short and bluntly rounded (ratio VL:GP av. 5.42); short setae on vertex, minute on thorax; vertex comparatively wide (ratio HW:VW av. 1.67); distal proboscis segment moderately long (av. length 0.10); hind tibia slender, shorter than head width (ratio HW:HT av. 1.08) (Fig. 9B–C, E, H). Female terminalia (Fig. 9F): proctiger dorsal surface more or less straight, longer than subgenital plate (ratio FP:FSP av. 1.35), apex acute, anal ring moderately long (ratio FP:RL av. 3.51); subgenital plate with no or slight medial bulge ventrally, apex acute; ovipositor apex with no or reduced serrations (2 above and below), valvulae dorsalis not strongly convex dorsally.

###### Egg.

Unpigmented or light brown, narrow, slender, entire surface with narrowly spaced uninterrupted striations no visible pedicel, tail short (Fig. 9G).

###### Immature.

Unknown.

###### Host plant notes.

Collected from semi-glabrous morphotype growing on lava flow (dated to 1880s) in south western Hawaii.

###### Island.

Hawaii.

###### Distribution notes.

Known from only one locality.

###### Biology.

Unknown, but may be free-living as for Pariaconus nigricapitus and Pariaconus proboscideus.

###### Etymology.

In reference to the dark dorsal stripe that is frequently present in this species and signifies affiliation with the bicoloratus group (adjective in the nominative singular).

###### Comments.

Currently known only from females; females appear particularly similar to Pariaconus lona from Molokai, however DNA analysis does not even place these two taxa in the same subgroup of the bicoloratus group, and this exemplifies the need for additional efforts to sample species diversity more completely.

###### Type material.

Holotype female (slide mounted, BMNH). See Table 2 for details of type and other material examined for this study.

##### Pariaconus kapo

Taxon classificationAnimaliaHemipteraTriozidae

Percy sp. n.

http://zoobank.org/C44CE816-AD40-4189-9064-7F1CDE2BA235

Figure 10

###### Adult colour.

General body colour yellow to brown. Head darker than the rest of the body, apparently not distinctly bicoloured (e.g. without distinct dorsal stripe). Fore wing membrane clear, or slightly fuscous.

###### Adult structure.

**Figure 10.**
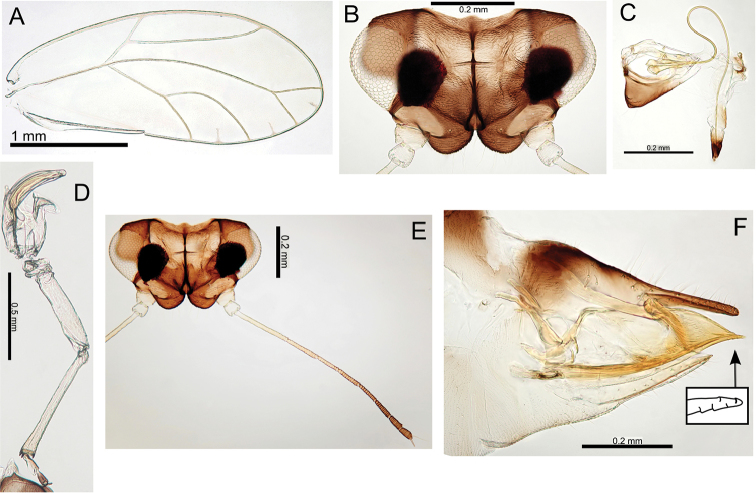
Pariaconus kapo sp. n. (female) **A** fore wing **B** head (uncleared ocular tissue) **C** proboscis **D** hind leg **E** head and antenna **F** terminalia (ovipositor serrations indicated).

Fore wing apex rounded; surface spinules dispersed in all cells but reduced or none in r_1_ and c+sc; setae on margins and veins short to minute (Fig. 10A). Antennae moderately long (av. length 0.94; ratio AL:HW av. 1.61); genal processes atypically well developed and bluntly rounded (ratio VL:GP av. 2.80); short to minute setae on vertex and thorax; distal proboscis segment moderately long (av. length 0.11); hind tibia slender, and longer than head width (ratio HW:HT av. 0.87) (Fig. 10B–E). Female terminalia (Fig. 10F): proctiger dorsal surface more or less straight, longer than subgenital plate (ratio FP:FSP av. 1.15), apex acute, anal ring moderately long (ratio FP:RL av. 3.50); subgenital plate with slight medial bulge ventrally, apex acute; ovipositor apex with reduced serrations (2 above and 2-3 below), valvulae dorsalis not strongly convex dorsally.

###### Egg.

Unknown.

###### Immature.

Unconfirmed, but 1^st^ instars recovered on the surface of leaves at the collection locality have a setal arrangement similar to that illustrated for Pariaconus oahuensis (Fig. 50F), with narrow, blunt sectasetae: anterior margin of the head with simple setae only, a single pair of post ocular sectasetae, a single pair of sectasetae on the apices of each wing bud, and the margin of the abdomen with 8 pairs of sectasetae.

###### Host plant notes.

Collected from pubescent morphotypes.

###### Island.

Hawaii.

###### Distribution notes.

Only known from Kohala.

###### Biology.

Unconfirmed, but this species was collected from low growing pubescent forms in upland bog; eggs and 1^st^ instar immatures were recovered from the plant surface among the trichomes along the mid-rib (upper leaf surface) and petiole, these eggs have widely spaced interrupted surface striations, a short pedicel and a long tail, however, two other bicoloratus species (Pariaconus proboscideus, and Pariaconus wyvernus form gorgonus) were collected at the same site and therefore association of this egg type remains uncertain.

###### Etymology.

Named after Kapo, a goddess of fertility in Hawaiian mythology (noun in the nominative singular standing in apposition to the generic name).

###### Comments.

Currently known from only one female; this is the largest species in the bicoloratus group and is unusual for the more well developed genae.

###### Type material.

Holotype female (slide mounted, BMNH). See Table 2 for details of type material examined for this study.

##### Pariaconus proboscideus

Taxon classificationAnimaliaHemipteraTriozidae

Percy sp. n.

http://zoobank.org/796D0199-5D77-4DD2-A4A5-B64E04BA5E8C

Figures 11 , 45F–K

###### Adult colour.

Typically bicoloured, generally pale cream-yellow thorax and abdomen, head brown or black, apparently lacking dorsal stripe. Fore wing membrane slightly fuscous.

###### Adult structure.

**Figure 11.**
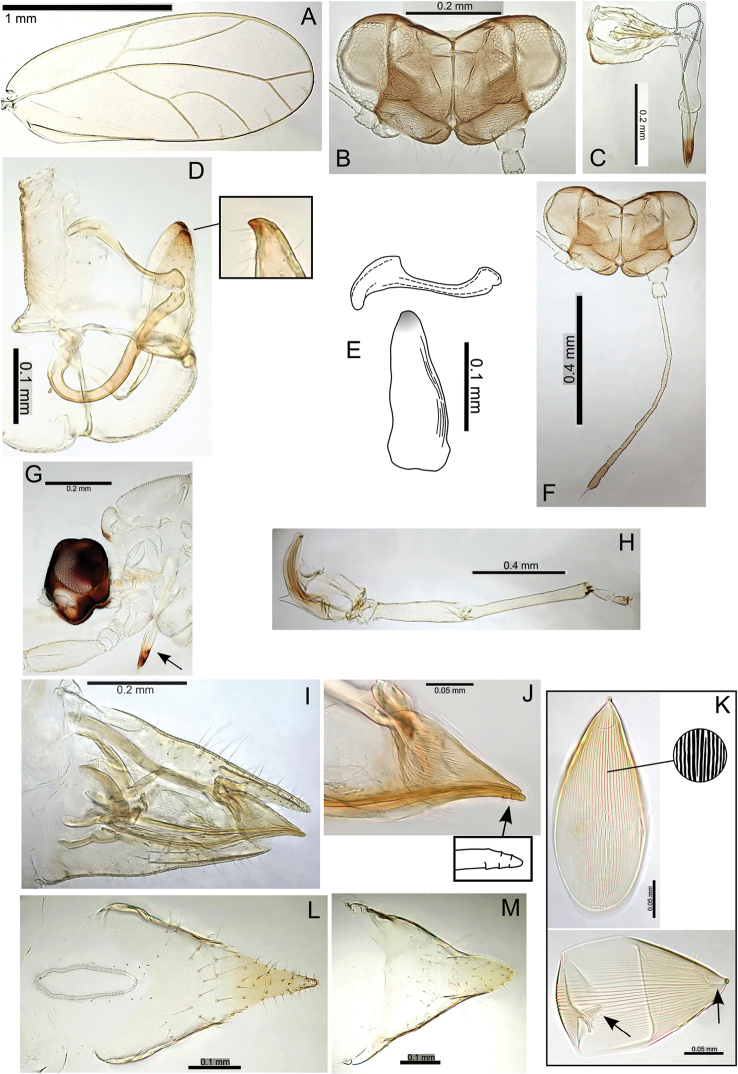
Pariaconus proboscideus sp. n. **A** fore wing **B** head **C** proboscis **D** male terminalia with dorsal view of paramere apex (inset) **E** aedeagus and paramere **F** head and antenna **G** head (with long distal proboscis segment indicated) **H** hind leg **I** female terminalia **J** ovipositor (serrations indicated) **K** eggs (pedicel, tail, and striations indicated) **L** female proctiger (dorsal view) **M** female subgenital plate (ventral view).

Fore wing apex rounded; surface spinules dispersed, usually in all cells except may be reduced or absent from cell r_1_ and c+sc; short setae on margins and veins (Fig. 11A). Antennae short (av. length 0.70; ratio AL:HW av. 1.52); genal processes short (ratio VL:GP av. 3.81); short to minute setae on vertex and thorax; distal proboscis segment atypically long (av. length 0.14); hind tibia longer than width of head (ratio HW:HT av. 0.90) (Fig. 11B–C, F–H). Male terminalia (Fig. 11D–E): paramere shorter than proctiger (ratio MP:PL av. 1.13), but slender and slightly sinusoidal, small interior directed hook at apex (Fig. 11E); distal aedeagus segment length subequal to paramere (ratio PL:AEL av. 1.04), base angular and moderately inflated, apex developed into a hook with bluntly acute apex (Fig. 11E) (ratio AEL:AELH av. 2.45). Female terminalia (Fig. 11I–J, L-M): proctiger long, slender, dorsal surface more or less straight, longer than subgenital plate (ratio FP:FSP av. 1.33), apex acute, anal ring short (ratio FP:RL av. 2.95); subgenital plate with slight medial bulge ventrally, apex acute; ovipositor apex with reduced serrations (0-2 above, 2-3 below), valvulae dorsalis not strongly convex dorsally.

###### Egg.

Unpigmented, broad, surface covered with long uninterrupted striations, short pedicel positioned 1/4 length from base, tail moderately long (Fig. 11K).

###### Immature.

Colour and structure 5^th^ instar: Appearance is white and spikey (hedgehog-like) due to coverage of stiff white filaments produced from sectasetae (Fig. 45F). Narrowly ovoid in outline with wing buds protruding and distinct humeral lobes (Fig. 45I–J). Tarsi with small reduced claws (Fig. 45H). Circumanal ring moderately wide (CPW:RW av. 3.72), and shallowly v-shaped, with a single row of uninterrupted elongate cells (Fig. 45K). Chaetotaxy 5^th^ instar: Entire dorsal surface and margins covered with pointed sectasetae (Fig. 45G).

###### Host plant notes.

On pubescent and tomentose morphotypes.

###### Island.

Hawaii.

###### Distribution notes.

Widespread on Hawaii: DNA analysis indicates distinct clusters of individuals from (a) Kohala as basal and sister to (b) Kau, (c) Saddle Road, and (d) Hualalai.

###### Biology.

This species is free-living on the undersides of pubescent leaves.

###### Etymology.

Named for the distinctly longer distal proboscis segment (adjective in the nominative singular).

###### Type material.

Holotype male (slide mounted, BMNH). See Table 2 for details of type and other material examined for this study.

##### Pariaconus poliahu

Taxon classificationAnimaliaHemipteraTriozidae

Percy sp. n.

http://zoobank.org/A819F0B0-CAC3-46B5-8FA1-0C7BFC3B124F

Figure 12

###### Adult colour.

Typically bicoloured, generally pale yellow to green on thorax and abdomen, head darker black or brown, and a dark dorsal stripe extending part or all the length of the body. Fore wing membrane slightly fuscous.

###### Adult structure.

**Figure 12.**
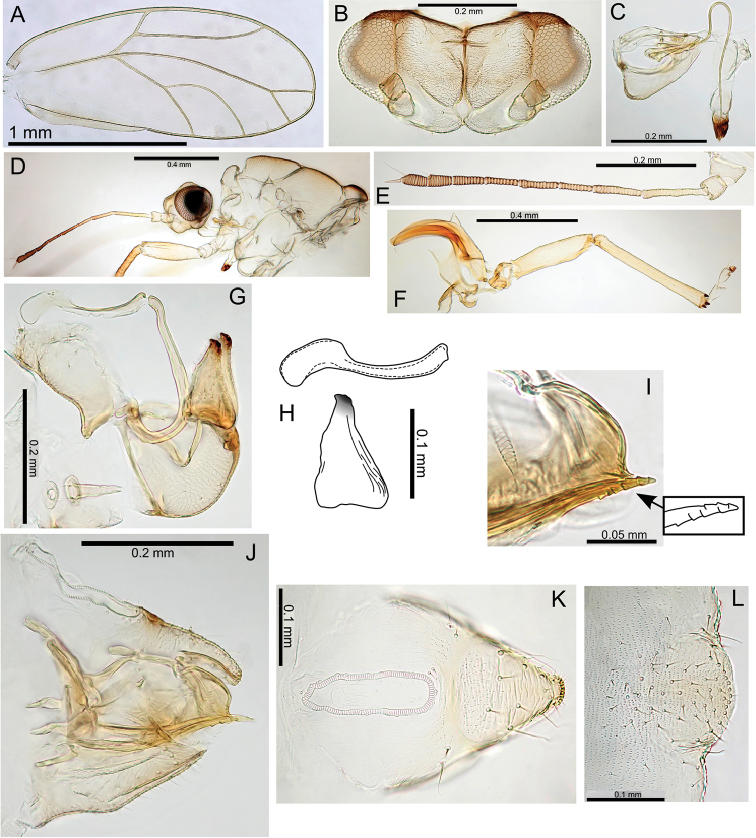
Pariaconus poliahu sp. n. **A** fore wing **B** head (female) **C** proboscis (female) **D** head and thorax (female) **E** antenna (female) **F** hind leg **G** male terminalia **H** aedeagus and paramere **I** ovipositor (serrations indicated) **J** female terminalia **K** female proctiger (dorsal view) **L** female subgenital plate (ventral view).

Fore wing apex rounded; surface spinules distributed in all cells except few or none in r_1_ and c+sc; short setae on margins and veins. Antennae short (av. length 0.67; ratio AL:HW av. 1.43); genal processes short and bluntly rounded (ratio VL:GP av. 5.00); short to minute setae on vertex and thorax; distal proboscis segment short (av. length 0.06); hind tibia slender, length subequal to head width (ratio HW:HT av. 1.05). Male terminalia: paramere shorter than proctiger (ratio MP:PL av. 1.22), broad at base and tapering to apex with anteriorly directed hook; distal aedeagus segment longer than paramere (ratio PL:AEL av. 0.75) with base rounded or slightly angular and slightly inflated, and a large, broadly rounded, hooked apex (Fig. 12H) (ratio AEL:AELH av. 2.39). Female terminalia: proctiger short, dorsal surface convex apically, apex bluntly rounded, anal ring extremely long (ratio FP:RL av. 2.01); subgenital plate with moderate medial bulge ventrally, apex truncate; ovipositor apex with serrations (2-3 above, and below), valvulae dorsalis strongly convex dorsally.

###### Egg.

Unpigmented, slender, and apparently without striations, pedicel or tail.

###### Immature.

Unknown.

###### Host plant notes.

Collected from mixed glabrous and pubescent morphotypes.

###### Island.

Hawaii.

###### Distribution notes.

Only known from the Kohala region of Hawaii.

###### Biology.

Unknown.

###### Etymology.

Named for Poliahu, a goddess of snow in Hawaiian mythology, in reference to a concept that each snowflake is unique, as many individuals sampled for this species have highly divergent genetic haplotypes (noun in the nominative singular standing in apposition to the generic name).

###### Type material.

Holotype male (slide mounted, BMNH). See Table 2 for details of type and other material examined for this study.

##### Pariaconus lona

Taxon classificationAnimaliaHemipteraTriozidae

Percy sp. n.

http://zoobank.org/3FEA62EA-DC69-4961-BF94-73DB68D708EB

Figure 13

###### Adult colour.

Typically bicoloured, generally pale yellow to green thorax and abdomen, head darker black or brown, and a dark dorsal stripe extending from head down the thorax. Fore wing membrane slightly fuscous.

###### Adult structure.

**Figure 13.**
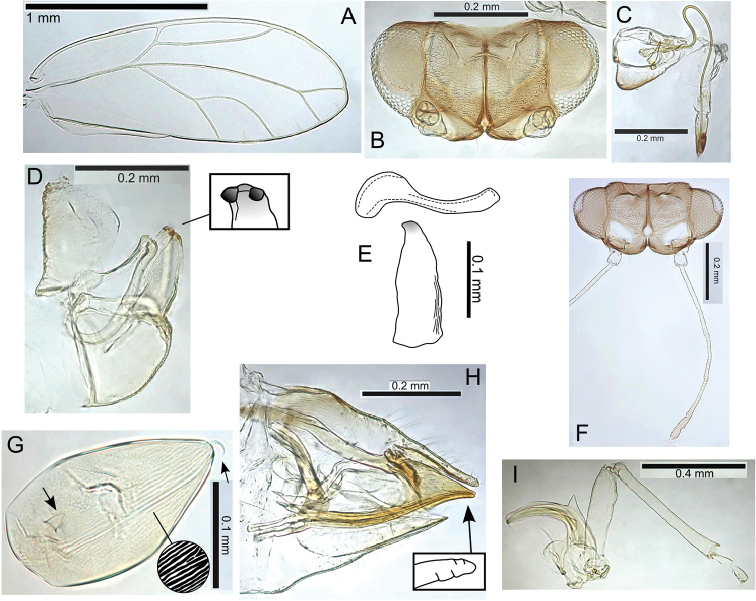
Pariaconus lona sp. n. **A** fore wing **B** head **C** proboscis **D** male terminalia with interior view of paramere apex (inset) **E** aedeagus and paramere **F** head and antenna **G** egg (pedicel, tail, and striations indicated) **H** female terminalia (ovipositor serrations indicated) **I** hind leg.

Fore wing apex bluntly acute; surface spinules dispersed, usually in all cells, but may be limited or absent from cells c+sc and r_1_; short setae on margins and veins (Fig. 13A). Antennae short (av. length 0.72; ratio AL:HW av. 1.38); genal processes extremely short and bluntly rounded (ratio VL:GP av. 3.27); short setae on vertex and thorax; distal proboscis segment moderately long (av. length 0.11); hind tibia slender, subequal or longer than head width (ratio HW:HT av. 0.95) (Fig. 13B–C, F, I). Male terminalia (Fig. 13D–E): paramere shorter than proctiger (ratio MP:PL 1.14), more or less parallel sided before tapering below dorsally flattened apex with bipartite sclerotization; distal aedeagus segment longer or subequal to paramere (ratio PL:AEL av. 0.91) with base angular and slightly inflated, and a large, broadly rounded, hooked apex (ratio AEL:AELH av. 2.30). Female terminalia (Fig. 13H): proctiger moderately long, dorsal surface more or less straight, apex acute, anal ring long (ratio FP:RL 3.41); subgenital plate long (ratio FP:FSP av. 1.48), with no or slight medial bulge ventrally, apex acute; ovipositor apex with reduced serrations (2 above, 2 below), valvulae dorsalis not strongly convex dorsally.

###### Egg.

Unpigmented to light brown, short, broad and with striations, mostly uninterrupted, on the dorsal surface, pedicel and tail short (Fig. 13G).

###### Immature.

Unknown.

###### Host plant notes.

Collected from glabrous morphotype.

###### Island.

Molokai.

###### Distribution notes.

Known from only one location in Kamakou Preserve.

###### Biology.

Unknown.

###### Etymology.

Named for Lona, a lunar deity in Hawaiian mythology (noun in the nominative singular standing in apposition to the generic name).

###### Type material.

Holotype male (slide mounted, BMNH). See Table 2 for details of type and other material examined for this study.

##### Pariaconus liliha

Taxon classificationAnimaliaHemipteraTriozidae

Percy sp. n.

http://zoobank.org/CD46756E-103B-430F-AF0C-449ABD7C6941

Figure 14

###### Adult colour.

Typically bicoloured, generally pale cream-yellow to green thorax and abdomen, head darker, with a dark dorsal stripe from the head extending part or all the length of the body. Fore wing membrane fuscous.

###### Adult structure.

**Figure 14.**
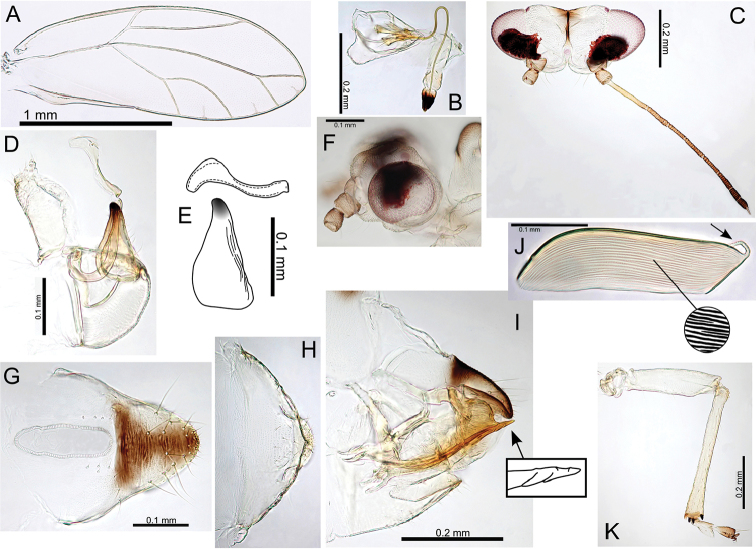
Pariaconus liliha sp. n. **A** fore wing **B** proboscis **C** head and antenna (uncleared ocular tissue) **D** male terminalia **E** aedeagus and paramere **F** head (uncleared ocular tissue) **G** female proctiger (dorsal view) **H** female subgenital plate (ventral view) **I** female terminalia (ovipositor serrations indicated) **J** egg (tail and striations indicated) **K** hind leg.

Fore wing moderately narrow, apex bluntly acute; surface spinules distributed in all cells, but limited in c+sc; short setae on margins and veins (Fig. 14A). Antennae short (av. length 0.75; ratio AL:HW av. 1.47); genal processes extremely short and bluntly rounded (ratio VL:GP av. 4.58); short to minute setae on vertex and thorax; distal proboscis segment short (av. length 0.07); hind tibia subequal or shorter than head width (ratio HW:HT av. 1.13) (Fig. 14B–C, F, K). Male terminalia (Fig. 14D–E): paramere shorter that proctiger (ratio MP:PL 1.18), broad at base and tapering to narrow neck below rounded apex with interiorly directed hook; distal aedeagus segment length subequal to paramere (ratio PL:AEL av. 1.03) with base angular and not inflated, and a broadly rounded, hooked apex (ratio AEL:AELH av. 2.20). Female terminalia (Fig. 14G–I): proctiger short, dorsal surface convex apically, apex bluntly rounded, anal ring extremely long (ratio FP:RL 1.98); subgenital plate extremely short (ratio FP:FSP av. 1.48), with slight medial bulge ventrally, apex truncate; ovipositor apex with shallow serrations (2 above, 2 below), valvulae dorsalis strongly convex dorsally (Fig. 14I).

###### Egg.

Light brown, extremely long, slender and with striations, mostly uninterrupted, over entire surface, no pedicel apparent, tail moderately long (Fig. 14J).

###### Immature.

Unknown.

###### Host plant notes.

Collected from glabrous morphotypes.

###### Island.

Oahu.

###### Distribution notes.

Only known from one locality, the high elevation bog area on Mnt Kaala.

###### Biology.

Unknown.

###### Etymology.

Named for Kuini Liliha, a High Chiefess who served the Kingdom of Hawaii as royal governor of Oahu (noun in the nominative singular standing in apposition to the generic name).

###### Type material.

Holotype male (slide mounted, BMNH). See Table 2 for details of type and other material examined for this study.

##### Pariaconus gracilis

Taxon classificationAnimaliaHemipteraTriozidae

(Crawford, 1918)

**Figure 15.**
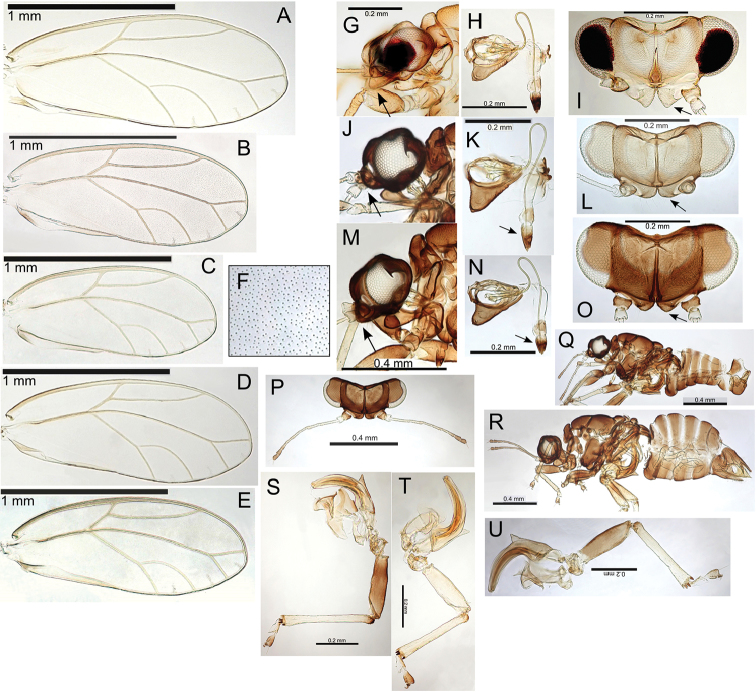
Pariaconus gracilis. **A, B, C, D, E** fore wing: **A** form gracilis (Waianae, Oahu) **B** form conconus (Koolau, Oahu) **C** form gracilis (Koolau, Oahu) **D** form gracilis (Molokai) **E** form gracilis (Maui) **F** detail of fore wing spinule density, form gracilis (Molokai) **G, H, I** form conconus (Koolau, Oahu): **G** head (uncleared ocular tissue) **H** proboscis **I** head (uncleared ocular tissue) **J, K, L** form gracilis (Aiea, Oahu): **J** head **K** proboscis **L** head **M, N, O** form gracilis (Maui): **M** head **N** proboscis **O** head **P, Q, R** form gracilis (Oahu): **P** head and antennae, **Q** male **R** female **S, T, U** hind legs: **S** form gracilis (Koolau, Oahu) **T** form conconus (Koolau, Oahu) **U** form gracilis (Maui).

**Figure 16.**
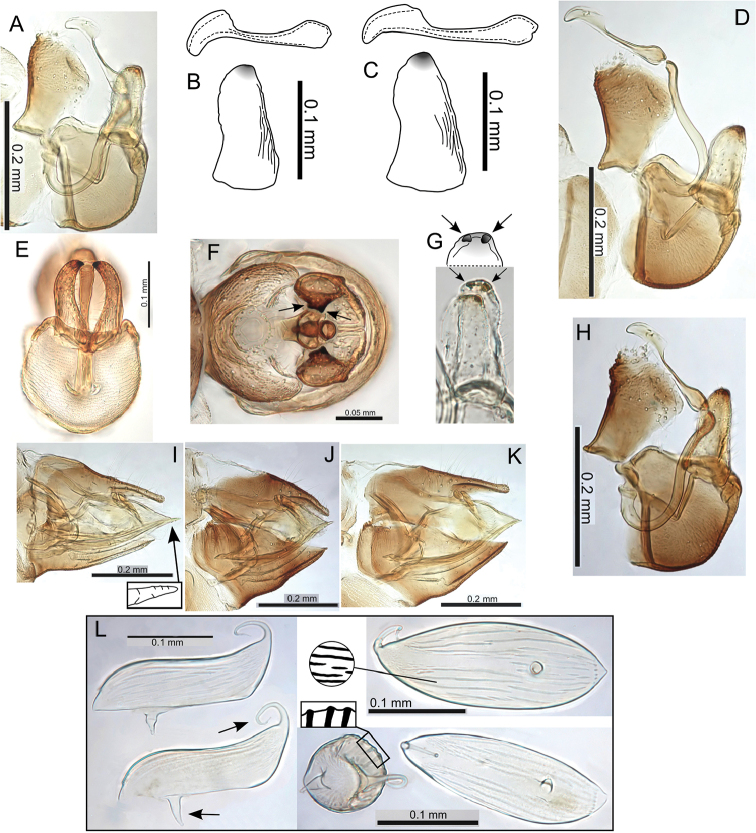
Pariaconus gracilis. **A, B** form gracilis (Aiea, Oahu): **A** male terminalia **B** aedeagus and paramere, **C, D** form gracilis (Molokai): **C** aedeagus and paramere **D** male terminalia **E** male terminalia (posterior view), form conconus (Koolau, Oahu) **F, G** form gracilis (Koolau, Oahu): **F** male terminalia (dorsal view, bipartite sclerotization indicated) **G** male paramere with interior view of paramere apex (bipartite sclerotization indicated) **H** male terminalia, form gracilis (Maui) **I, J** form gracilis (Oahu): **I** female terminalia (ovipositor serrations indicated) (Koolau) **J** female terminalia (Waianae) **K** female terminalia, form gracilis (Molokai) **L** eggs (pedicel, tail, and striations indicated), form gracilis (Oahu).

Figures 15 , 16 , 46A–F

Kuwayama gracilis Crawford, 1918: 447.
Pariaconus gracilis (Crawford), Enderlein (1926): 401.

###### Adult colour.

General body colour typically dark brown to black throughout, but occasionally partly or entirely pale cream or yellow throughout (Fig. 15Q–R). Head often darker than the rest of the body. Individuals are not distinctly bicoloured (e.g. without distinct dorsal stripe). Fore wing membrane clear, or slightly fuscous.

###### Adult structure.

Fore wing apex rounded; surface spinules densely distributed throughout all cells; short to minute setae on margins and veins (Fig. 15A–F). Antennae short (av. length 0.54; ratio AL:HW av. 1.02); genal processes extremely short (ratio VL:GP av. 5.63); short to minute setae on vertex and thorax; distal proboscis segment short (av. length 0.07); hind tibia short relative to head width (ratio HW:HT av. 1.31) Fig. 15G–P, S–U). Male terminalia (Fig. 16A–H): paramere shorter than proctiger (ratio MP:PL av. 1.25), broad at the base and tapering only slightly towards the apex, sometimes slightly constricted medially, no apical hook but apex with bipartite sclerotization (Fig. 16G); distal aedeagus segment longer than paramere (ratio PL:AEL av. 0.88) with inflated base, apex hooked but flattened dorsally, hook apex blunt (ratio AEL:AELH av. 2.96). Female terminalia (Fig. 16I–K): proctiger dorsal surface either straight, or with slight medial depression (Koolau), anal ring long (ratio FP:RL av. 2.44), apex bluntly acute; subgenital plate with slight or more pronounced medial bulge ventrally, apex bluntly acute; ovipositor apex with no or very reduced serrations above, two reduced serrations below, valvulae dorsalis not strongly convex dorsally.

###### Egg.

Unpigmented, elongate, slender, slightly sinusoidal, dorsal surface with widely spaced interrupted striations, medium-short pedicel positioned 1/4-1/3 length from base, tail long (Fig. 16L).

###### Immature.

Colour and structure: Orange or cream. 5^th^ instar: Narrowly ovoid in outline with wing buds protruding and with distinct humeral lobes (Fig. 46A, D). Tarsi with small reduced claws (Fig. 46D). Circumanal ring shallowly v-shaped, with a single row of elongate cells (Fig. 46C, E). Chaetotaxy: 5^th^ instar: Margin with medially expanded and overlapping diamond-shaped setae, dorsal surface rugose with ridges but otherwise only minute simple setae (Fig. 46B–C). 1^st^ instar (Fig. 46F): anterior margin of the head with long simple setae, otherwise marginal setae are narrow, blunt sectasetae (a single pair post ocular, a single pair on the apices of each wing bud, and the margin of the abdomen with approximately 9-10 pairs); by the 2^nd^ instar, the characteristic diamond-shaped sectasetae are evident around the entire margin.

###### Host plant notes.

Predominantly on pubescent and tomentose morphotypes.

###### Island.

Oahu, Molokai, Maui.

###### Distribution notes.

A common species on Oahu. Four clusters can be recognized in the DNA analysis: (a) individuals from Molokai and Maui; these in turn group with (b) a population from Oahu’s southern Koolau Mnts that have more developed genal processes (Fig. 15G, I). Another cluster includes (c) individuals from both southern and northern Koolau Mnts with moderately reduced genal processes (Fig. 15J, L), and (d) a fourth cluster comprises all individuals from Mnt Kaala, Waianae Mnts, with much reduced genal processes. It appears that this Kaala population is ancestral with increasing development of genal processes being a derived characteristic in Koolau populations; Maui and Molokai specimens have moderately reduced genal processes and appear to be immigrants from the Koolau region of Oahu.

###### Biology.

The immatures are free-living, usually on the lower leaf surface of pubescent and tomentose morphotypes, this host morphotype preference is also noted on slide specimens collected in 1973 by Beardsley (BISH). Eggs appear to be laid mostly singly and sparsely distributed amongst the leaf trichomes.

###### Comments.

One of the most commonly encountered species in the bicoloratus group. Two forms are recognized (Figs 15–16): form gracilis (based on the type is the more common form, with short rounded genae) (Fig. 15J, L), and form conconus (has more developed, apically acute genae) (Fig. 15G, I). Both these forms can be found sympatrically in the Koolau Mountains (Oahu) and form distinct genetic clusters, suggesting some reproductive isolation.

###### Type material.

Holotype female (dry mounted, BPBM). See Table 2 for details of type and other material examined for this study.

##### Pariaconus dorsostriatus

Taxon classificationAnimaliaHemipteraTriozidae

Percy sp. n.

http://zoobank.org/2DCB25CC-D170-41BB-8769-2F05C92EF7B9

Figures 17 , 47A–J

###### Adult colour.

Variable, usually bicoloured with orange-brown, cream or yellow to greenish-yellow thorax and abdomen, and a dark dorsal stripe from the head extending part or all the length of the body. Fore wing fuscous, especially around anal margin.

###### Adult structure.

**Figure 17.**
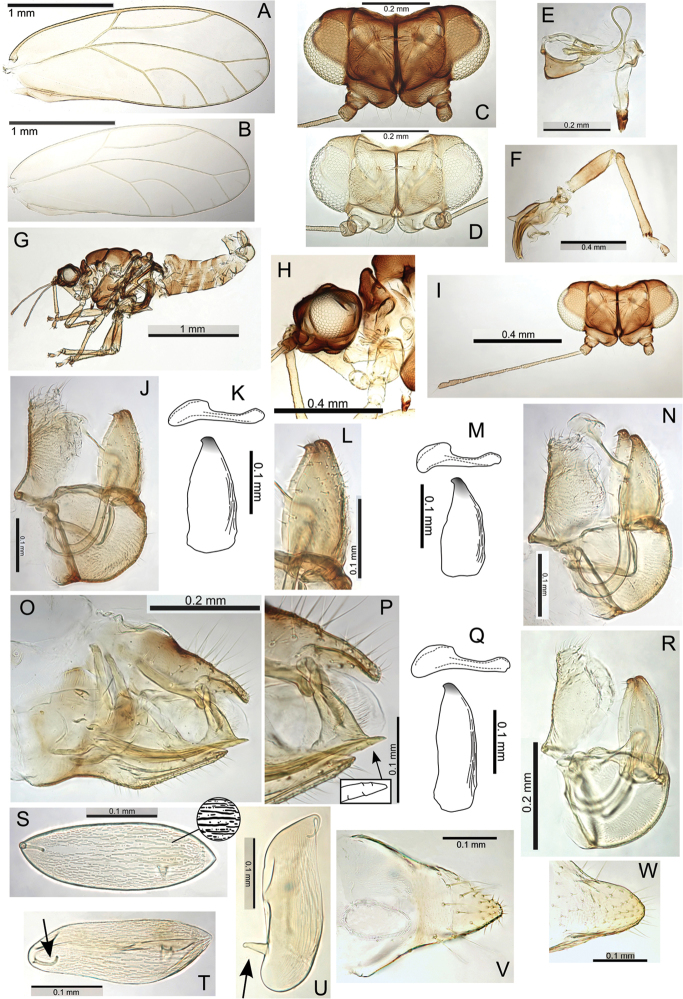
Pariaconus dorsostriatus sp. n. **A, B** fore wing: **A** (Kohala, Hawaii) **B** (Olaa, Hawaii) **C, D** head: **C** (Kohala, Hawaii) **D** (Olaa, Hawaii) **E** proboscis **F** hind leg **G** male **H** head **I** head and antennae **J, K, L** form kohalensis variation 1 (Kohala, Hawaii): **J** male terminalia **K** aedeagus and paramere **L** paramere **M, N** form kohalensis variation 2 (Kohala, Hawaii): **M** aedeagus and paramere **N** male terminalia **O** female terminalia **P** ovipositor (serrations indicated) **Q, R** form communis (Olaa, Hawaii): **Q** aedeagus and paramere **R** male terminalia **S, T, U** eggs (pedicel, tail, and striations indicated): **S, T** (Kohala, Hawaii) **U** (Olaa, Hawaii) **V** female proctiger (dorsal view) **W** female subgenital plate (ventral view).

Fore wing apex bluntly acute to rounded; dispersed spinules present in all cells, but reduced or absent in cell r_1_; setae on margins and veins short to minute (Fig. 17A–B). Antennae short (av. length 0.63; ratio AL:HW av. 1.23); genal processes short (ratio VL:GP av. 3.87); short setae on vertex, minute on thorax; distal proboscis segment short (av. length 0.07); hind tibia longer than head width (ratio HW:HT av. 0.91) (Fig. 17C–I). Male terminalia (Fig. 17J–N, Q–R): paramere length subequal to proctiger (ratio MP:PL av. 1.01), broad at base, not sinusoidal, tapering evenly to small apical hook appearing flat topped from lateral aspect; distal aedeagus segment shorter than paramere (ratio PL:AEL av. 1.23), base rounded and slightly inflated, apex triangular not developed into a hook (ratio AEL:AELH av. 2.19). Female terminalia (Fig. 17O–P, V–W): proctiger short, subequal to subgenital plate, usually depressed medially (ratio FP:FSP av. 0.93), anal ring short (ratio FP:RL av. 2.65), apex bluntly acute; subgenital plate with slight medial bulge ventrally, apex bluntly acute; ovipositor apex with no or very reduced serrations above, two reduced serrations below, valvulae dorsalis not strongly convex dorsally.

###### Egg.

Unpigmented, narrowly oval, marginally sinusoidal, entire surface with interrupted striations, medium-short pedicel positioned 1/4 length from base, tail short (Fig. 17S–U).

###### Immature.

Colour and structure: Pale cream, yellow to green. 5^th^ instar: Broadly ovoid and ventro-dorsally flattened wing buds only slightly protruding and distinct humeral lobes (Fig. 47A–B, J). Tarsi with small reduced claws. Circumanal ring wide (CPW:RW av. 4.24), and more or less straight, with a single row of uninterrupted elongate cells (Fig. 47A). Chaetotaxy: 5^th^ instar: Continuous marginal ring of blunt, weakly bisected sectasetae (Fig. 47C). Dorsal surface rugose and either without setae or with scattered minute simple setae. 1^st^ instar (Fig. 47I): Margin with broadly fan-shaped sectasetae (9 pairs anterior margin of head, 1 pair postocular, 1 pair on each wing bud, 12 pairs abdominal); by the 2^nd^ instar there is a continuous marginal ring of sectasetae (Fig. 47H).

###### Host plant notes.

Apparently prefers glabrous morphotypes, with pit galls mostly on the lower leaf surface, occasionally on the upper leaf surface.

###### Island.

Hawaii.

###### Distribution notes.

Appears to be widespread; collected from four regions that group into distinct clusters in the DNA analysis: (a) Puu Makaala, (b) Alili plus Kau, (c) Humuula (Hamakua Coast), and (d) the Kohala region (Fig. 3).

###### Biology.

Immatures make pit galls, typically on the lower leaf surface that are initially shallow, becoming deeper with older instars (Fig. 47D–G).

###### Etymology.

Named for the dark dorsal stripe that is frequently present in this species and signifies its affiliation with the bicoloratus group (adjective in the nominative singular).

###### Comments.

One of the largest species in the bicoloratus group. Two forms are recognized (Fig. 17): form kohalensis (based on the type is found in the Kohala region, with broader, shorter paramere, and shorter genae) (Fig. 17J–N), and form communis (more common and widespread, has a more slender, longer paramere, e.g. Fig. 17Q–R [with more round apex in Kau/Alili] and more well developed genae).

###### Type material.

Holotype male (slide mounted, BMNH). See Table 2 for details of type and other material examined for this study.

##### Pariaconus namaka

Taxon classificationAnimaliaHemipteraTriozidae

Percy sp. n.

http://zoobank.org/538455FD-5F50-4CA2-810C-63E4A923A424

Figures 18 , 47K–U

###### Adult colour.

Variable, either entirely pale cream or yellow to greenish-yellow, or with head darker; a partial or weakly marked dorsal stripe extends from the head part or all the length of the body. Fore wing membrane clear or slightly fuscous.

###### Adult structure.

**Figure 18.**
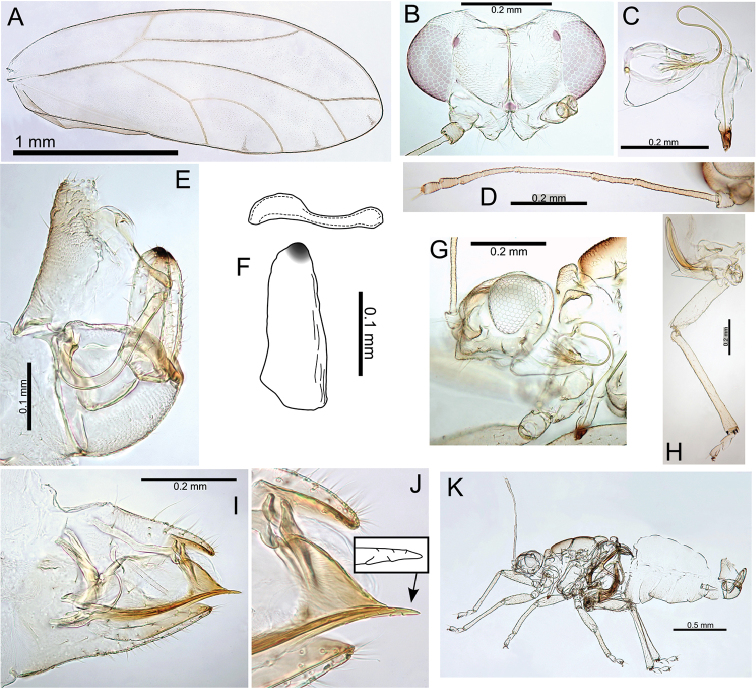
Pariaconus namaka sp. n. **A** fore wing **B** head **C** proboscis **D** antenna **E** male terminalia **F** aedeagus and paramere **G** head **H** hind leg **I** female terminalia **J** ovipositor (serrations indicated) **K** male.

Fore wing narrow, apex bluntly acute; surface spinules distributed in all cells, but limited in c+sc; short setae on margins and veins (Fig. 18A). Antennae short (av. length 0.85); genal processes moderately short and acute (ratio VL:GP av. 2.35); short to minute setae on vertex and thorax; distal proboscis segment short (av. length 0.07); hind tibia long and slender, longer than head width (ratio HW:HT av. 0.78) (Fig. 18B–D, G–H, K). Male terminalia (Fig. 18E–F): paramere shorter that proctiger (ratio MP:PL 1.13), more or less parallel sided before tapering below a rounded apex with interiorly directed hook; distal aedeagus segment shorter than paramere (ratio PL:AEL av. 1.17) with base rounded and slightly inflated, and a rounded, shallow hooked apex (ratio AEL:AELH av. 2.41). Female terminalia (Fig. 18I–J): proctiger moderately short, dorsal surface more or less straight, apex acute, anal ring long (ratio FP:RL 3.06); subgenital plate long (ratio FP:FSP av. 0.89), without or with very slight medial bulge ventrally, apex acute; ovipositor apex with reduced serrations (2 above, 0-2 below), valvulae dorsalis not strongly convex dorsally.

###### Egg.

Unknown.

###### Immature.

Colour and structure: Pale cream to orange-yellow. 5^th^ instar: Ovoid and ventro-dorsally flattened wing buds only slightly protruding and distinct humeral lobes (Fig. 47K-L, N). Tarsi with small reduced claws (Fig. 47M). Circumanal ring wide (CPW:RW av. 4.04), and more or less straight, with a single row of uninterrupted elongate cells (Fig. 47P). Chaetotaxy: 5^th^ instar: Continuous marginal ring of blunt, weakly bisected sectasetae (Fig. 47O). Dorsal surface is rugose and either without setae or with scattered minute simple setae. 1^st^ instar (Fig. 47Q): Margin with broadly fan-shaped sectasetae (anterior of head with 9 pairs, 1 pair postocular, 1 pair on apices of each wing bud, 14 pairs on the abdomen).

###### Host plant notes.

Collected on glabrous morphotypes.

###### Island.

Oahu.

###### Distribution notes.

Only known from one locality, the high elevation bog area on Mnt Kaala.

###### Biology.

Immatures make pit galls on the lower leaf surface (Fig. 47R–U), after eclosion the remaining pits are generally shallower than the likely sister taxon on Hawaii, Pariaconus dorsostriatus.

###### Etymology.

Named after Namaka, a sea goddess or water spirit in Hawaiian mythology, in reference to the type locality in the wet bog on top of Mnt Kaala (noun in the nominative singular standing in apposition to the generic name).

###### Comments.

A pit-galling habit on Oahu was only recently discovered, previously pit-gallers were only known from younger islands and their presence on Oahu together with subfossils on Kauai (see Discussion) supports an older and more widespread status for the bicoloratus group.

###### Type material.

Holotype male (slide mounted, BMNH). See Table 2 for details of type and other material examined for this study.

#### minutus species group

Currently this group includes only two species. These are small species, similar to those in the bicoloratus group, but usually entirely one colour, not bicoloured. The eggs are slender and the immatures develop in shallow pits on the upper leaf surface, and occasionally lower leaf surface of pubescent morphotypes of the host plant. This group consistently clusters as sister to the kamua species group rather than together with other pit gallers and free-living species in the bicoloratus group in the molecular analyses.

##### Pariaconus minutus

Taxon classificationAnimaliaHemipteraTriozidae

(Crawford, 1918)

Figures 19 , 46G–P

Kuwayama minuta Crawford, 1918: 447.
Pariaconus minutus (Crawford), Enderlein (1926): 401.

###### Adult colour.

Variable, typically mid- to dark brown throughout, recently emerged adults can be completely pale cream, head often darker than the rest of the body. A population in the Kilauea Iki caldera (form kilaueaiensis) is typically yellow-orange or dark orange throughout, or occasionally with blue-green abdomens. Fore wing membrane slightly to noticeably fuscous.

###### Adult structure.

**Figure 19.**
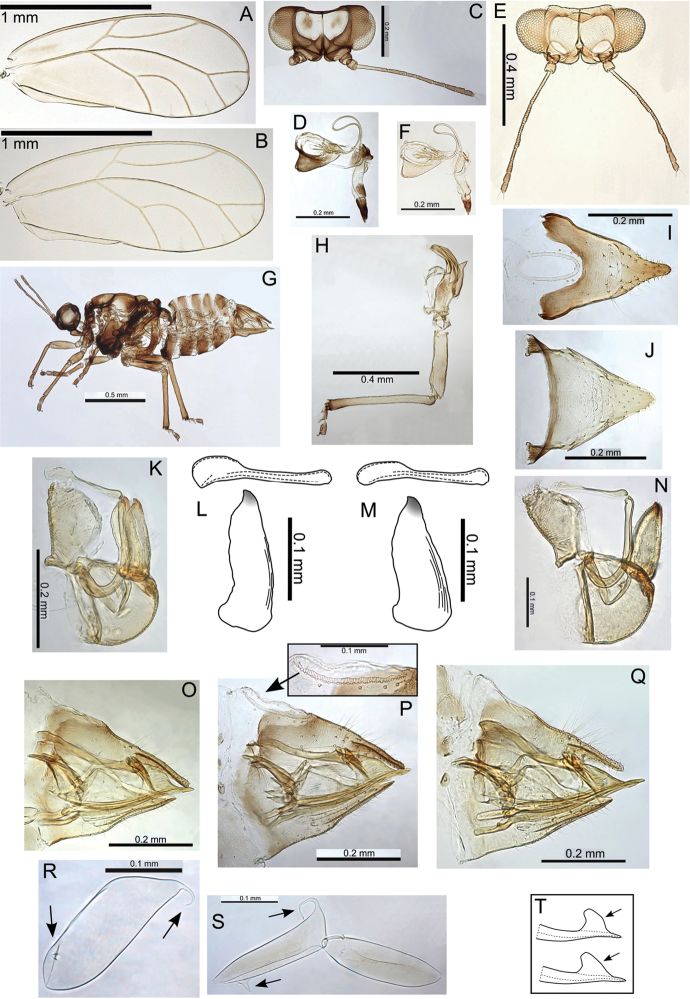
Pariaconus minutus. **A, B** fore wing: **A** form minutus **B** form kilaueaiensis **C, D** form minutus: **C** head and antenna **D** proboscis **E, F** form kilaueaiensis: **E** head and antenna **F** proboscis **G** female **H** hind leg **I** female proctiger (dorsal view) **J** female subgenital plate (ventral view) **K, L** form minutus: **K** male terminalia **L** aedeagus and paramere **M, N** form kilaueaiensis: **M** aedeagus and paramere **N** male terminalia **O, P** female terminalia, form minutus: **O** (Olaa, Hawaii) **P** (Saddle Rd., Hawaii) **Q** female terminalia, form kilaueaiensis **R, S** eggs (pedicel and tail indicated): **R** form minutus **S** form kilaueaiensis **T** ovipositors (shape variation indicated), form minutus (above) form kilaueaiensis (below).

Fore wing apex rounded; surface spinules fairly densely distributed in all cells; setae on margins and veins minute (Fig. 19A–B). Antennae short (av. length 0.52; ratio AL:HW av. 1.13); genal processes short (ratio VL:GP av. 3.25); minute setae on vertex and thorax; distal proboscis segment short (av. length 0.09); hind tibia length subequal to head width (ratio HW:HT av. 1.00) (Fig. 19C–H). Male terminalia (Fig. 19K–N): length of paramere and proctiger subequal (ratio MP:PL av. 0.98), paramere slender, somewhat sinuous (curving posteriorly then anteriorly at the apex), apex with upwardly directed hook; length of distal aedeagus segment and paramere subequal (ratio PL:AEL av. 1.03), base rounded and slightly inflated, apex blunt, somewhat flattened dorsally, not developed into a hook (ratio AEL:AELH av. 2.50). Female terminalia (Fig. 19I–J, O–Q, T): proctiger dorsal surface slightly undulating, anal ring long (ratio FP:RL av. 2.66), apex acute; subgenital plate with slight medial bulge ventrally, apex acute; ovipositor apex with two reduced serrations above and below, valvulae dorsalis slightly to moderately convex dorsally (Fig. 19T).

###### Egg.

Unpigmented to light brown, elongate ovoid, not sinusoidal, smooth, without striations, short pedicel 1/5 length from base, tail short to moderately long (Fig. 19R–S).

###### Immature.

Colour and structure: Smaller immatures are orange and cream, or yellow-brown, larger become blue-green or remain orange (e.g. Kilauea Iki population). 5^th^ instar: Broadly ovoid in outline and ventro-dorsally flattened with wing buds protruding and distinct humeral lobes (Fig. 46G). Tarsi with small reduced claws (Fig. 46I). Anal ring moderately wide (ratio CPW:RW av. 4.76) and shallowly v-shaped, with a single row of uninterrupted elongate cells (Fig. 46G). Chaetotaxy: 5^th^ instar: Continuous marginal ring of blunt, weakly bisected sectasetae. Dorsal surface either scattered with small pointed sectasetae (form minutus, ﻿Fig. 46G), or small to minute simple setae (form kilaueaiensis). 1^st^ instar (Fig. 46J): Marginal ring of broad, weakly bisected fan-shaped sectasetae (anterior of head with 11-12 pairs, 1 pair postocular, 1 pair on apices of each wing bud, and 11-12 pairs on abdomen). Apparently only in form kilaueaiensis (Kilauea Iki population) are three pairs of narrow sectasetae present on the dorsal surface of 1^st^ instars (1 pair on each of head, thorax, abdomen). By the 2^nd^ instar there is a continuous marginal ring of sectasetae (Fig. 46H).

###### Host plant notes.

Usually on thick leaved, pubescent or semi-pubescent morphotypes.

###### Island.

Hawaii.

###### Distribution notes.

Widespread on Hawaii. The DNA analysis indicates distinct population clusters, but also more dispersal than for other taxa: two distinct clusters in Kohala, a mixed cluster from Olaa, Kilauea, Kau, and Kona Hema, and another cluster almost entirely from Saddle Road with a single Kau sample.

###### Biology.

Makes pit galls on upper leaf surface. The leaf tissue often forms into a thickened rim of red or yellow around the immature (Fig. 46L–P). The Kilauea Iki population makes somewhat shallower pit galls on the upper leaf surface of low growing shrubs (~1m) in the bottom of the caldera. Eggs are laid individually or in small clusters long the upper leaf margin (Fig. 46K).

###### Comments.

Two forms are recognized (Fig. 19): form minutus (based on the type, with mature adults usually brown, is a smaller form with more slender paramere), and form kilaueaiensis (with adults typically orange, is larger with a shorter, broader paramere).

###### Type material.

Holotype, male (slide mounted, BPBM). See Table 2 for details of type and other material examined for this study.

##### Pariaconus gibbosus

Taxon classificationAnimaliaHemipteraTriozidae

Percy sp. n.

http://zoobank.org/B1C332D6-23F3-4F47-95AF-6F4043CFC630

Figure 20

###### Adult colour.

Most specimens examined are almost entirely dark brown to black, however, as with Pariaconus minutus, it is likely there are paler forms, such as when newly emerged. Fore wing membrane clear to moderately fuscous.

###### Adult structure.

**Figure 20.**
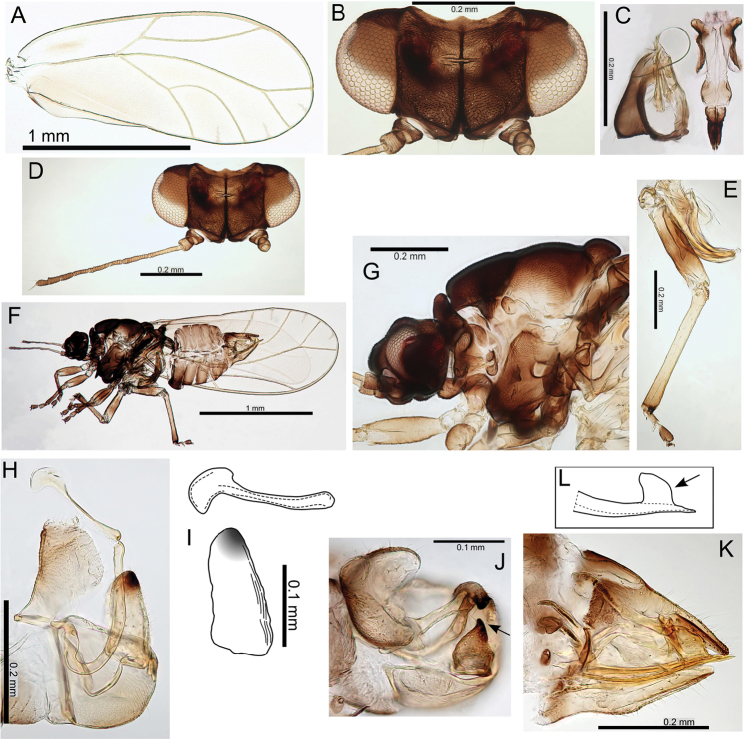
Pariaconus gibbosus sp. n. **A** fore wing **B** head **C** proboscis **D** head and antenna **E** hind leg **F** female **G** head and thorax **H** male terminalia **I** aedeagus and paramere **J** male terminalia (dorsal view, inward directed apex indicated) **K** female terminalia **L** ovipositor (convex shape of valvulae dorsalis indicated).

Fore wing apex rounded; surface spinules fairly densely distributed in all cells; setae on margins and veins minute (Fig. 20A). Antennae short (av. length 0.52; ratio AL:HW av. 1.03); genal processes extremely short (ratio VL:GP av. 8.21); minute setae on vertex and thorax; distal proboscis segment short (av. length 0.07); hind tibia shorter than head width (ratio HW:HT av. 1.10) (Fig. 20B–G). Male terminalia (Fig. 20H–J): length of paramere and proctiger subequal (ratio MP:PL 1.03), paramere broad, slightly sinuous (curving posteriorly at the apex), apex hook interiorly directed; length of distal aedeagus segment longer than paramere (ratio PL:AEL 0.89), base rounded and slightly inflated, apex developed into broadly rounded hook with acute apex (ratio AEL:AELH 2.75). Female terminalia (Fig. 20K–L): proctiger dorsal surface more or less straight, anal ring long (ratio FP:RL av. 2.51), apex acute; subgenital plate with slight medial bulge ventrally, apex acute; ovipositor apex with two reduced serrations above and below, valvulae dorsalis strongly convex dorsally (Fig. 20L).

###### Egg.

Unknown.

###### Immature.

Unknown.

###### Host plant notes.

Collected from pubescent morphotypes.

###### Island.

Maui.

###### Distribution notes.

Only known from eastern Maui, in the Makawao area.

###### Biology.

Unknown, but likely to be pit galling given the biology of the sister taxon, Pariaconus minutus.

###### Etymology.

Named for the more dorsally humped (gibbosus) shape of paramere apex, aedeagus apex, and ovipositor valvulae dorsalis that distinguishes this species from the sister taxon, Pariaconus minutus (adjective in the nominative singular).

###### Comments.

Adults of this species are easily confused with Pariaconus gracilis in the field. However, Maui is currently the only island where both species occur. Both species are often almost entirely dark brown to black, and similar in overall size.

###### Type material.

Holotype male (slide mounted, BMNH). See Table 2 for details of type and other material examined for this study.

#### kamua species group

The kamua species group is a monophyletic group of at least 10 species endemic to the island of Kauai. It includes the largest and some of the smallest species in Pariaconus, as well as the only species in the genus with a distinct fore wing colour pattern (Pariaconus melanoneurus), and the only species with a more pronounced lobe on the posterior of the male proctiger, particularly in the largest species in the group (e.g. Pariaconus hiiaka). It encompasses perhaps a larger degree of adult morphological variation than found in other species groups, but the immatures are known for only three species, and many of the species are known from only one locality. There is a diversity of galls, including enclosed leaf and stem galls similar to those in the ohialoha group, and a unique type of open gall that is produced on the side of plant stems whereby the inner cambium extrudes out to produce a cup gall. Further study of the different biologies of this species group is required to establish the full range of galling behaviours. Both the endemism of the kamua group on the oldest island of Kauai, and the molecular data, support the interpretation of independent origins of closed galling on Kauai and on the younger islands.

#### Adult key to Pariaconus species found on Kauai – kamua species group

**Table:** 

1	Fore wing membrane with distinct brown patches bordering wing veins, shape of male and female terminalia as in Fig. 23	**Pariaconus melanoneurus sp. n.**
–	Fore wing membrane without distinct brown patches bordering wing veins, membrane either clear or slightly fuscous	**2**
2	Male with paramere extremely broad, with prominent posterior shoulder below apex, female terminalia short (≤0.60 × head width), female subgenital plate strikingly concave ventrally, shape of male and female terminalia as in Fig. 21	**Pariaconus iolani (Kirkaldy, 1902), comb. n.**
–	Male with paramere narrower, more or less parallel sided or tapering, without or with only moderate posterior shoulder below apex, female terminalia ≥0.70 × head width, female subgenital plate more or less straight or slightly convex ventrally	**3**
3	Large species (antennal length >1.30 mm, head width >0.70 mm, wing length ≥2.95), female terminalia long (>0.80 mm) with apex acute and extremely small anal ring (ratio FP:RL >8), shape of male and female terminalia as in Fig. 24	**Pariaconus grandis sp. n.**
–	Smaller species (antennal length <1.30 mm, head width <0.70 mm, wing length <2.95), female terminalia shorter (<0.80 mm) with apex either acute, blunt or truncate, but ring relatively large (ratio FP:RL <5)	**4**
4	Larger species (antennal length >0.90 mm, head width ≥0.60 mm), with broader wings (ratio WL:WW <2.35), male terminalia with paramere longer than proctiger, aedeagus hook large (ratio AEL:AELH <2.15), female terminalia short (ratio FP:HW <0.75) and subgenital plate truncate, ovipositor apex with numerous distinct serrations, shape of male and female terminalia as in Fig. 22	**Pariaconus hiiaka sp. n.**
–	Smaller species (antennal length <0.90 mm, head width ≤0.60 mm), with narrower wings (ratio WL:WW >2.35), male terminalia with paramere shorter than proctiger, aedeagus hook smaller (ratio AEL:AELH >2.15), female terminalia relatively long and acute (ratio FP:HW <0.75), ovipositor apex with few reduced serrations	**5**
5	Male with short proctiger and paramere (both ≤0.15 mm, ratio PL:SH <0.90), but aedeagus hook relatively large (ratio AEL:AELH <2.25) with blunt apex, shape of male terminalia as in Fig. 30	**Pariaconus haumea sp. n.**
–	Male with longer proctiger and paramere (both >0.15 mm, ratio PL:SH >0.90), but aedeagus hook relatively small (ratio AEL:AELH >2.25) with acute apex	**6**
6	Female with shorter terminalia (FP <0.60 mm, ratio FP:HW <1.20), male (where known) either with paramere tapering to slender neck below apex, or constricting just below apex	**7**
–	Female with long terminalia (FP >0.60 mm, ratio FP:HW >1.20), shape of female terminalia as in Fig. 28 (male unknown)	**Pariaconus elegans sp. n.**
7	Female anal ring relatively small (ratio FP:RL >2.7), egg without surface striations, male (where known) either with paramere tapering to slender neck below apex, or constricting just below apex	**8**
–	Female anal ring relatively large (ratio FP:RL <2.7), egg with surface striations, shape of female terminalia as in Fig. 29 (male unknown)	**Pariaconus gagneae sp. n.**
8	General body colour brown to red, paramere tapering to slender neck below apex, egg with long sinuous tail, shape of male and female terminalia as in Fig. 25 (makes thin-walled cup galls)	**Pariaconus caulicalix sp. n.**
–	General body colour orange, yellow or yellow-brown, paramere tapering to apex or more parallel sided and constricted just below apex, egg with short bulbous tail	**9**
9	Male with paramere tapering gradually to apex, aedeagus hook less well developed, shape of male and female terminalia as in Fig. 26 (makes thick-walled cup galls)	**Pariaconus crassiorcalix sp. n.**
–	Male with paramere more parallel side, constricting just below apex, aedeagus hook more well developed, shape of male and female terminalia as in Fig. 27	**Pariaconus lehua (Crawford, 1925), comb. n.**

##### Pariaconus iolani

Taxon classificationAnimaliaHemipteraTriozidae

(Kirkaldy, 1902) comb. n.

Figure 21

Trioza iolani Kirkaldy, 1902: 114 in part (Kauai specimens, nec Oahu specimens), type designated by lectotypification in 1908: 206; non Trioza iolani Crawford, 1918, nec Zimmerman, 1948.
Trioza kauaiensis Crawford, 1925: 29, **syn. n.**

###### Adult colour.

General body colour green, yellow-green or yellow-orange, often with brown on legs, thorax and abdomen. Females may have a darker abdomen due to darkly pigmented egg load. Fore wing membrane clear.

###### Adult structure.

**Figure 21.**
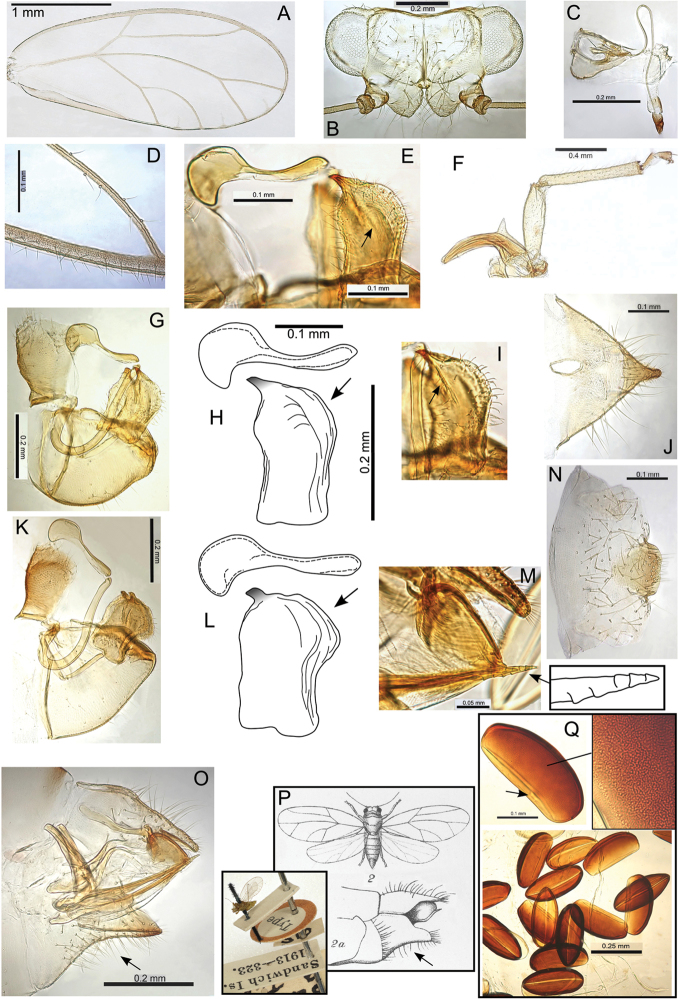
Pariaconus iolani. **A** fore wing **B** head **C** proboscis **D** fore wing detail **E** aedeagus and paramere (central ridge indicated), form iolani **F** hind leg **G, H, I** form iolani: **G** male terminalia **H** aedeagus and paramere (posterior shoulder indicated) **I** paramere (interior view, central ridge indicated) **J** female proctiger (dorsal view) **K, L** form scapulus: **K** male terminalia **L** aedeagus and paramere (posterior shoulder indicated) **M** ovipositor (serrations indicated) **N** female subgenital plate (ventral view) **O** female terminalia (concave subgenital plate indicated) **P** original illustration (Kirkaldy 1902) (concave subgenital plate indicated) and female lectotype (inset) **Q** eggs (pedicel and microsculpturing indicated).

Fore wing apex rounded; surface spinules sparsely distributed, usually in all cells except limited or absent from cell r_1_; long setae on margins and particularly dense on the ventral margin, sparse long setae on veins (Fig. 21A, D). Antennae long (av. length 1.33; ratio AL:HW av. 1.85); genal processes length short-medium and bluntly acute (ratio VL:GP av. 2.04); medium to long setae on vertex and thorax; distal proboscis segment short (av. length 0.09); hind tibia thick, length shorter or subequal to head width (ratio HW:HT av. 0.95) (Fig. 21B–C, F). Male terminalia (Fig. 21E, G–I, K–L): paramere length subequal to proctiger (ratio MP:PL av. 1.06), paramere extremely broad, with a prominent central ridge and posterior bulge or “shoulder”, apex directed anteriorly; subgenital plate extending posteriorly to give a somewhat triangular rather than rounded shape; distal aedeagus segment length subequal to paramere (ratio PL:AEL av. 0.93) with base rounded and slightly inflated, and a large hooked apex (ratio AEL:AELH av. 2.20). Female terminalia (Fig. 21J, M–O): proctiger short, dorsal surface straight, apex constricted in dorsal view and bluntly acute, anal ring short (ratio FP:RL av. 4.17); subgenital plate strikingly concave ventrally and apex truncate (ratio FP:FSP av. 1.12); ovipositor apex with distinct serrations (3 above, 4 below), valvulae dorsalis not strongly convex dorsally.

###### Egg.

Mid- to light brown, elongate oval, with longitudinal medial suture entire length of egg (coffee bean-like), surface with microsculpturing and granular in appearance, extremely short pedicel 1/3 length from base, tail lacking (Fig. 21Q).

###### Immature.

Unknown.

###### Host plant notes.

Collected predominantly from glabrous and semi-pubescent morphotypes.

###### Island.

Kauai.

###### Distribution notes.

Collected in several locations in Kokee State Park, including Alakai, Kalalau and Nu Alolo.

###### Biology.

Based on phylogenetic closeness to Pariaconus hiiaka, and the large body size, this species may have a similar closed gall biology, but further study is required to confirm.

###### Comments.

The female lectotype (dry mounted, BMNH) has been examined and compared with the female syntype (dry mounted, BPBM) of Trioza kauaiensis Crawford, 1925. In publications after 1908, the name Trioza iolani has been almost exclusively associated with a common Oahu species (here designated Pariaconus oahuensis). There is no way to avoid this unfortunate synonymization given the following circumstances: Kirkaldy (1902) referred to two specimens in his original description, one from Kauai and one from Oahu, but the two specimens are not from the same species. In the original description, he illustrates only the specimen from Kauai but does not publish a designated type. However, in 1908 he validly lectotypified the Kauai specimen: “The type was from Kauai”, and this specimen also bears a hand written label (apparently in Kirkaldy’s handwriting), “male type” (though the lectotype is in fact female) (Fig. 21P). The name iolani must therefore be considered to apply to the Kauai specimen, rather than the Oahu specimen, and the Kauai specimen belongs to the same species as that described by Crawford in 1925 as Trioza kauaiensis. Kirkaldy probably had a relatively broad concept of psyllid species (not being a specialist in this group), and he may not have had a good understanding of psyllid morphology because both the specimens and the illustration accompanying the original description (Plate IV, Fig. 2; and shown here in Fig. 21P) are females, not males as Kirkaldy thought them to be. Kirkaldy’s other female specimen clearly fits the concept of Trioza iolani *sensu* Crawford (1918, 1925) and Zimmerman (1948). Neither Crawford nor Zimmerman appear to have examined the BMNH type material, and Crawford only examined additional material collected by Kirkaldy from Oahu. Nevertheless, Zimmerman (1948) correctly noted that the specimen from Kauai was the type for iolani, and with typical astuteness discerned, “…some confusion regarding the identity of this species. It is generally thought of as one of the commonest psyllids on Oahu, yet the holotype was designated as a Kauai specimen. Further study might reveal that the Oahu form is a distinct species from the Kauai form”.

Two forms are recognized on Kauai (Fig. 21): form iolani (based on the type is more common, with paramere apex more extended and posterior shoulder rounded) (Fig. 21H), and form scapulus (with paramere apex short and distinctly extended posterior shoulder) (Fig. 21L). More specimens and an investigation of the biology is required to establish if these are distinct species.

###### Type material.

Lectotype, female (dry mounted, BMNH). Syntype, female (dry mounted, BPBM). See Table 2 for details of type and other material examined for this study.

##### Pariaconus hiiaka

Taxon classificationAnimaliaHemipteraTriozidae

Percy sp. n.

http://zoobank.org/DBEBA3F9-A990-45F2-8E2B-0A0061BA9D7A

Figures 22 , 48A–L

###### Adult colour.

General body colour red-brown or brown. Fore wing membrane clear.

###### Adult structure.

**Figure 22.**
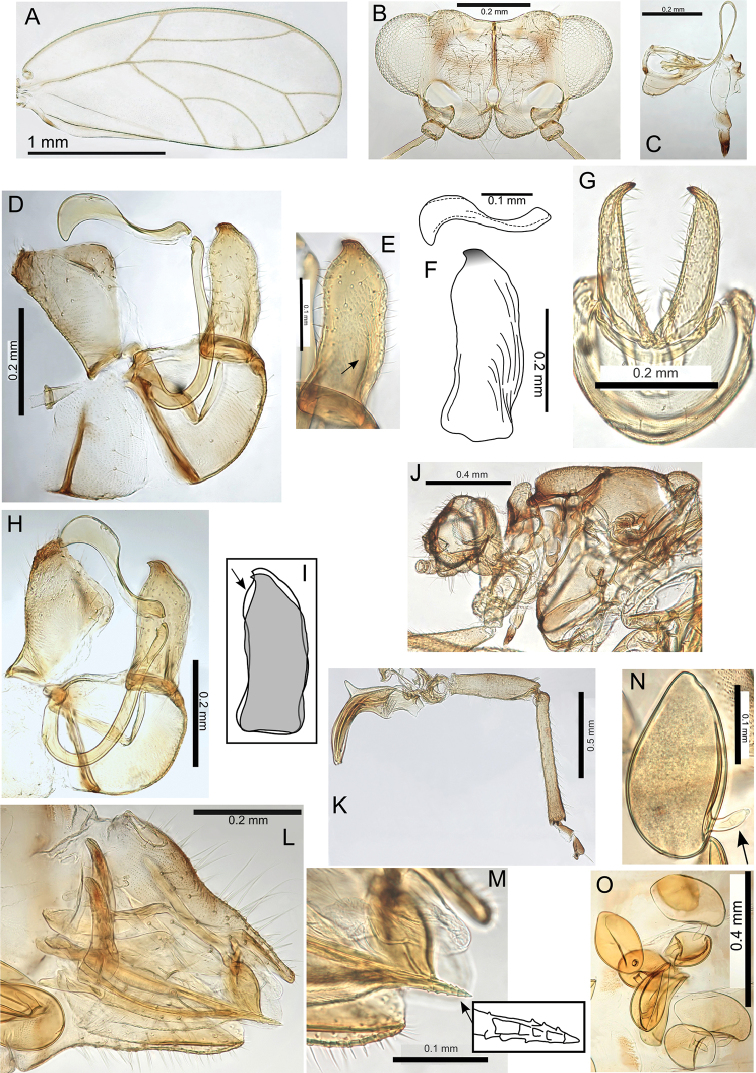
Pariaconus hiiaka sp. n. **A** fore wing **B** head **C** proboscis **D** male terminalia **E** paramere **F** aedeagus and paramere **G** male terminalia (posterior view) **H** male terminalia (variation) **I** paramere shape variation comparison **J** head and thorax **K** hind leg **L** female terminalia **M** ovipositor (serrations indicated) **N, O** eggs (pedicel indicated).

Fore wing apex rounded; surface spinules with limited distribution, few or none in cells r_1_, r_2_, m_2_; long setae on margins and veins (Fig. 22A). Antennae long (av. length 1.08; ratio AL:HW av. 1.71); genal processes length short-medium, converging, and bluntly acute (ratio VL:GP av. 2.03); medium to long setae on vertex and thorax; distal proboscis segment short (av. length 0.10); hind tibia thick, longer than or subequal to head width (ratio HW:HT av. 0.91) (Fig. 22B–C, J–K). Male terminalia (Fig. 22D–I): paramere length subequal to proctiger (ratio MP:PL av. 0.96), paramere broad, more or less parallel-sided, apex almost flat-topped in profile with slight anteriorly directed hook; distal aedeagus segment marginally shorter than paramere (ratio PL:AEL av. 1.03) with base angular and slightly inflated, and a large hooked apex (ratio AEL:AELH av. 2.01). Female terminalia (Fig. 22L–M): proctiger dorsal surface slightly medial depressed, apex bluntly acute, anal ring short (ratio FP:RL av. 4.33); subgenital plate with slight medial bulge ventrally and apex blunt and slightly truncate (ratio FP:FSP av. 1.16); ovipositor apex with distinct serrations (3 above, 3-4 below), valvulae dorsalis moderately convex dorsally.

###### Egg.

Light brown, smooth, apparently without microsculpturing but with a granular appearance, short pedicel 1/4 length from base, tail lacking (Fig. 22N–O).

###### Immature.

Colour and structure: Smaller instars orange, larger becoming yellow or blue-green with grey thorax and head. 5^th^ instar ovoid in outline with wing buds protruding and nondistinct humeral lobes (Fig. 48A, C). Tarsi with large claws (Fig. 48B). Circumanal ring small, u-shape with a single row of often interrupted cells (Fig. 48D). Younger instars are ovate (egg-shaped) with broad head and narrowing abdomen (Fig. 48E). Chaetotaxy: 1^st^-5^th^ instars: Head, thorax and abdomen with scattered short to long simple setae. 1^st^ instar (Fig. 48E): anterior margin of the head with simple setae, a single pair of short simple setae post ocular, a single pair of short simple setae on the apices of each wing bud, and the margin of the abdomen apparently lacking setae.

###### Host plant notes.

Collected predominantly from glabrous and semi-pubescent morphotypes.

###### Distribution.

Kauai.

###### Distribution notes.

Collected in two locations in Kokee State Park.

###### Biology.

This species forms enclosed galls on leaves that resemble flat leaf galls in the ohialoha group, but are typically more domed (Fig. 48F–H), and in some cases either convex or concave in the centre (e.g. resembling Pariaconus pele form kohalensis, see Fig. 52Z). The galls open by a hinged circular door (Fig. 48I–K) in similar fashion to Pariaconus pyramidalis on Hawaii (see Fig. 52W–X). Gall density can severely deform leaves, and cause whole leaf necrosis (Fig. 48F, L). In one location an immature dissected from a stem gall also DNA barcoded to this species (see discussion on galling lability). This species frequently co-occurs with other galling taxa (Fig. 48M).

###### Etymology.

Named after Hiiaka in Hawaiian mythology, the favoured sister of Pele, who dwelled in a sacred Lehua grove and journeyed to Kauai (noun in the nominative singular standing in apposition to the generic name).

###### Comments.

Some variation in paramere shape, particularly in development of anterior shoulder, is illustrated in Fig. 22H-I.

###### Type material.

Holotype male (slide mounted, BMNH). See Table 2 for details of type and other material examined for this study.

##### Pariaconus melanoneurus

Taxon classificationAnimaliaHemipteraTriozidae

Percy sp. n.

http://zoobank.org/387701FA-EDDE-4DC5-8652-94CE6DF94019

Figure 23

###### Adult colour.

**Figure 23.**
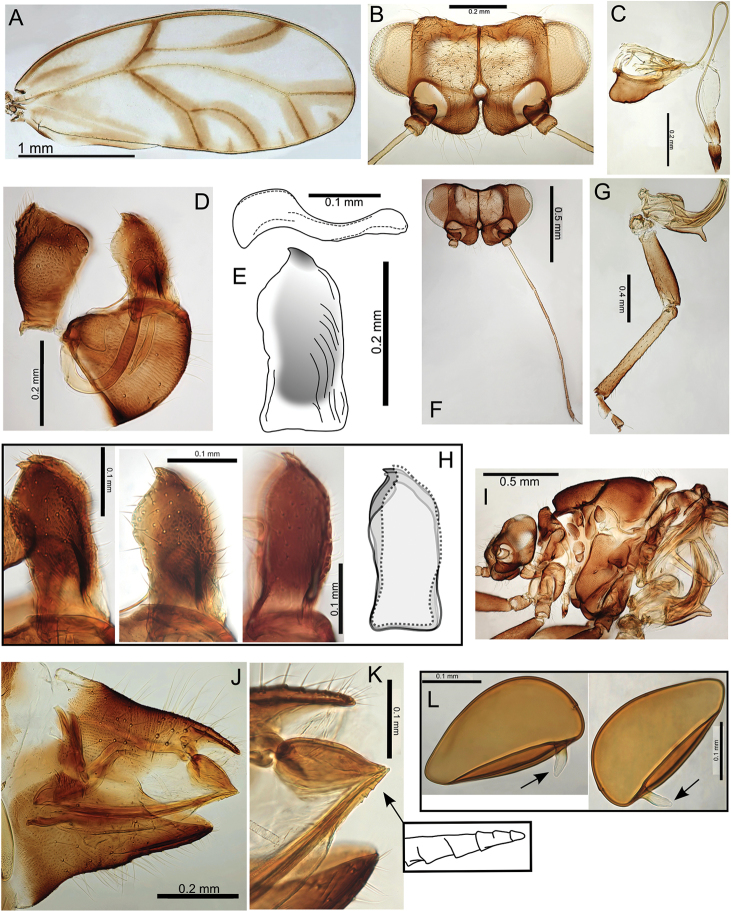
Pariaconus melanoneurus sp. n. **A** fore wing **B** head **C** proboscis **D** male terminalia **E** aedeagus and paramere **F** head and antenna **G** hind leg **H** male parameres and shape variation comparison **I** head and thorax **J** female terminalia **K** ovipositor (serrations indicated) **L** eggs (pedicel indicated).

General body colour mid- to dark brown. Fore wing membrane with brown pigmentation around wing base and patches of brown pigmentation bordering veins resulting in a distinct wing pattern (Fig. 23A).

###### Adult structure.

Fore wing apex rounded; surface spinules with limited distribution in cells c+sc and cu_2_, and absent or very limited in all other cells; long setae on margins and veins (Fig. 23A). Antennae long (av. length 1.31; ratio AL:HW av. 1.87); genal processes length short-medium, converging, and bluntly acute (ratio VL:GP av. 1.90); long setae on vertex and thorax; distal proboscis segment short (av. length 0.10); hind tibia thick, longer than head width (ratio HW:HT av. 0.87) (Fig. 23B–C, F–G, I). Male terminalia (Fig. 23D–E, H): paramere length subequal to proctiger (ratio MP:PL av. 1.04), paramere broad, more or less parallel-sided but broadening in apical 1/3 just below constriction to apex, apex with acute point directed anteriorly; distal aedeagus segment shorter than paramere (ratio PL:AEL av. 1.18) with base rounded and slightly inflated, and a large bluntly hooked apex (ratio AEL:AELH av. 2.29). Female terminalia (Fig. 23J–K): proctiger dorsal surface slightly medial depressed, apex bluntly acute, anal ring short (ratio FP:RL av. 4.55); subgenital plate with slight medial bulge ventrally and apex blunt and slightly truncate, marginally longer than proctiger (ratio FP:FSP av. 0.93); ovipositor apex with distinct serrations (3 above, 3 below), valvulae dorsalis slightly convex dorsally.

###### Egg.

Light brown, smooth, apparently without microsculpturing but with a slight granular appearance, short pedicel 1/4 length from base, tail lacking (Fig. 23L).

###### Immature.

Unknown.

###### Host plant notes.

Morphotype preference unknown, adults collected on both glabrous and pubescent types.

###### Island.

Kauai.

###### Distribution notes.

Known from only one location in Kokee State Park.

###### Biology.

Unknown, but its close relationship with Pariaconus iolani and Pariaconus hiiaka suggest it is likely to make closed galls.

###### Etymology.

Named for the dark pigmentation around the fore wing veins (adjective in the nominative singular).

###### Comments.

This species is the only member of Pariaconus to have a distinctly patterned fore wing. Variation in paramere shape is illustrated in Fig. 23H.

###### Type material.

Holotype male (slide mounted, BMNH). See Table 2 for details of type and other material examined for this study.

##### Pariaconus grandis

Taxon classificationAnimaliaHemipteraTriozidae

Percy sp. n.

http://zoobank.org/5ACCE084-1CC0-4E65-A8A3-1B0455D9D066

Figure 24

###### Adult colour.

General body colour is either brown to yellow-brown, or green. Fore wing membrane clear.

###### Adult structure.

**Figure 24.**
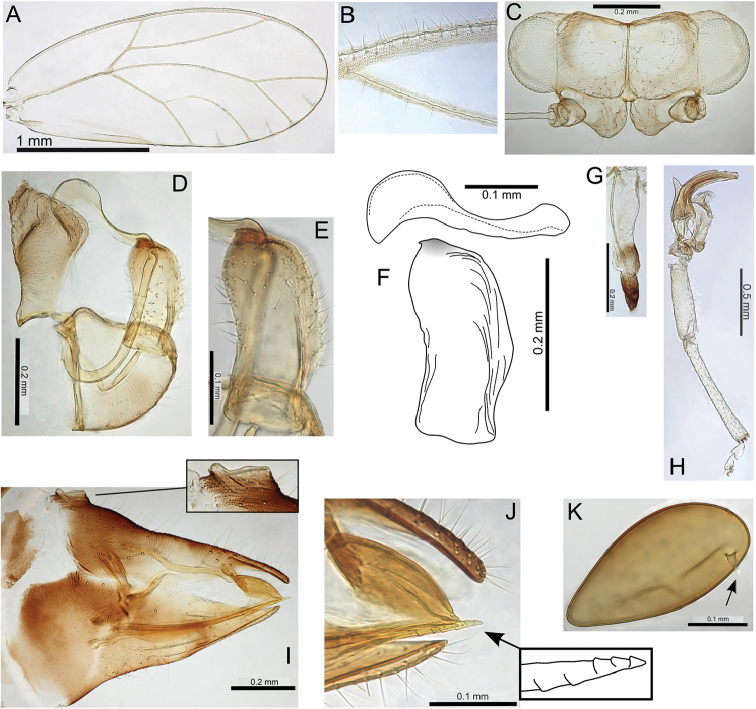
Pariaconus grandis sp. n. **A** fore wing **B** fore wing detail **C** head **D** male terminalia **E** paramere **F** aedeagus and paramere **G** proboscis **H** hind leg **I** female terminalia showing raised anal ring collar (inset) **J** ovipositor (serrations indicated) **K** egg (pedicel indicated).

Fore wing apex rounded; surface spinules with limited distribution in all cells except absent from r_1_; long setae on margins and medium long on veins (Fig. 24A–B). Antennae long (av. length 1.48; ratio AL:HW av. 1.93); genal processes length short-medium, converging, and bluntly acute (ratio VL:GP av. 2.05); long setae on vertex and thorax; distal proboscis segment short (av. length 0.11); hind tibia thick, longer than head width (ratio HW:HT av. 0.86) (Fig. 24C, G–H). Male terminalia (Fig. 24D–F): paramere shorter than proctiger (ratio MP:PL 1.14), broad, more or less parallel-sided but broadening medially, and whole curving anteriorly from middle, apex moderately constricted with acute point directed anteriorly; distal aedeagus segment length subequal to paramere (ratio PL:AEL 1.00) with base rounded and slightly inflated, and a large bluntly hooked apex (ratio AEL:AELH 2.14). Female terminalia (Fig. 24I–J): proctiger long, dorsal surface slightly medial depressed, apex bluntly acute, anal ring extremely short and on a raised collar (ratio FP:RL 8.70); subgenital plate with slight medial bulge ventrally and apex acute, length subequal to proctiger (ratio FP:FSP 1.01); ovipositor apex with distinct serrations (3 above, 2 below), valvulae dorsalis moderately convex dorsally.

###### Egg.

Light brown, smooth, apparently without microsculpturing, short pedicel 1/4 length from base, tail lacking (Fig. 24K).

###### Immature.

Unknown.

###### Host plant notes.

Morphotype preference unknown, adults collected on both glabrous and pubescent types.

###### Island.

Kauai.

###### Distribution notes.

Known from only one location, Kalalau Valley in Kokee State Park.

###### Biology.

Unknown, but morphological affinities with Pariaconus hiiaka suggest may make closed galls.

###### Etymology.

The name refers to the large size of the species, it is the largest of the Metrosideros-feeding psyllids in the Hawaiian Islands (adjective in the nominative singular).

###### Comments.

This is the largest of the Metrosideros-feeding species in the Hawaiian Islands, but it is only marginally larger than some of the other large taxa (e.g. Pariaconus iolani, Pariaconus oahuensis, Pariaconus mauiensis, Pariaconus hawaiiensis) that are generally yellow-green and probably predominantly stem gallers.

###### Type material.

Holotype male (slide mounted, BMNH). See Table 2 for details of type and other material examined for this study.

##### Pariaconus caulicalix

Taxon classificationAnimaliaHemipteraTriozidae

Percy sp. n.

http://zoobank.org/9D1E6BD8-0659-4890-A204-F33F86423EFD

Figures 25 , 49A–J

###### Adult colour.

General body colour is either brown to dark brown, or light red to red-brown. Fore wing membrane clear or slightly fuscous.

###### Adult structure.

**Figure 25.**
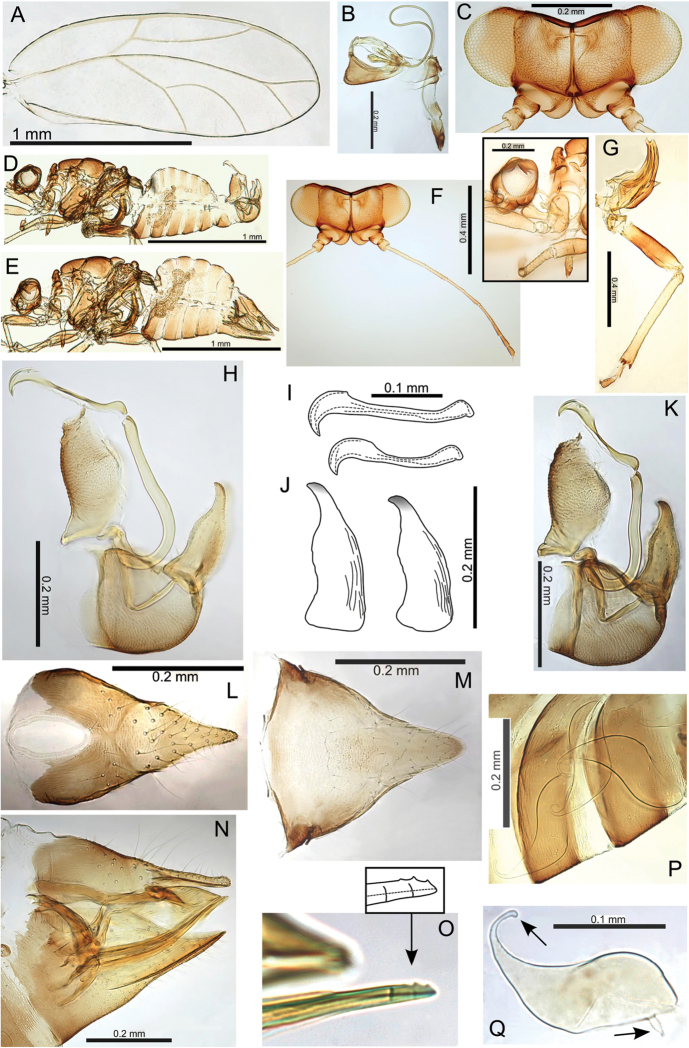
Pariaconus caulicalix sp. n. **A** fore wing **B** proboscis **C** head **D** male **E** female **F** head and antenna and head (lateral view, inset) **G** hind leg **H** male terminalia, form brunneis **I** aedeagi: form brunneis (above), form rubrus (below) **J** parameres: form brunneis (left), form rubrus (right) **K** male terminalia, form rubrus **L** female proctiger (dorsal view) **M** female subgenital plate (ventral view) **N** female terminalia **O** ovipositor (serrations indicated) **P, Q** eggs (pedicel and tail indicated).

Fore wing apex bluntly acute; surface spinules distributed in all cells except few or none in r_1_; short setae on margins and veins (Fig. 25A). Antennae short (av. length 0.71; ratio AL:HW av. 1.41); genal processes short (ratio VL:GP av. 3.17); short to minute setae on vertex and thorax; distal proboscis segment short (av. length 0.11); hind tibia length subequal to head width (ratio HW:HT av. 1.06) (Fig. 25B–G). Male terminalia (Fig. 25H–K): paramere shorter than proctiger (ratio MP:PL av. 1.14), broader at the base and tapering to an elongate neck below a somewhat flat-topped apex with anteriorly directed hook; distal aedeagus segment length subequal to paramere (ratio PL:AEL av. 0.99) with base rounded or slightly angular and slightly inflated, and a moderately large acutely hooked apex (ratio AEL:AELH av. 2.73). Female terminalia (Fig. 25L–O): proctiger short, dorsal surface more or less straight, apex acute, anal ring long (ratio FP:RL av. 2.98); subgenital plate with very slight medial bulge ventrally, apex acute to bluntly acute; ovipositor apex with reduced serrations (2 above, 0-2 below), valvulae dorsalis not strongly convex dorsally.

###### Egg.

Unpigmented to light brown, elongate and sinusoidal, no microsculpturing, short pedicel 1/4 length from base, long tail with slightly inflated tip (Fig. 25P–Q).

###### Immature.

Colour and structure: Black or brown dorsally, cream to pale orange ventrally. 5^th^ instar: Broadly ovoid in outline, wing buds only slightly protruding with distinct humeral lobes (Fig. 49A–B). Dorsal surface with ridges (Fig. 49D). Tarsi with moderately small claws (not extending beyond arolia) (Fig. 49B). Circumanal ring moderately wide (CPW:RW 5.89), shallowly v-shaped, with a single row of elongate cells (Fig. 49A). Chaetotaxy: 5^th^ instar: Continuous marginal ring of blunt sectasetae (Fig. 49C). Dorsal surface with intermittent minute simple setae. 1^st^ instar (Fig. 49F): Margin with broad blunt sectasetae (8 pairs anterior margin of head, 1 pair postocular, 1 pair on each wing bud, 12 pairs on abdomen), and with distinct arrangement of large to medium large, acutely pointed sectasetae dorsally (4 pairs head, 4 pairs thorax, 2-5 pairs abdomen), which are lost by the 3^rd^-4^th^ instars; by the 2^nd^ instar there is a continuous marginal ring of sectasetae (Fig. 49E).

###### Host plant notes.

Found predominantly on glabrous and semi-pubescent morphotypes.

###### Island.

Kauai.

###### Distribution notes.

The two recognized forms (brunneis and rubrus) of Pariaconus caulicalix are found sympatrically (although form brunneis is more widespread), which, given the molecular differentiation, suggests that, in addition to colour and general size differences that are noticeable in the field, there may be some reproductive isolation.

###### Biology.

This species forms thin-walled cup galls on stems, often clustered together, with one immature per gall chamber (Fig. 49G–J). The gall tissue of the cup is green or yellow-green. Immatures are seated in the base of the cup gall and the ridged sclerotized dorsal surface forms a plug under which is the soft unsclerotized body (Fig. 49G–H).

###### Etymology.

The name refers to the gall type which is a cup (*calix*) -shaped cambial outgrowth on plant stems (*caulae*) (adjective in the nominative singular).

###### Comments.

Two forms are recognized (Fig. 25): form brunneis (more common, larger and generally brown, with a longer paramere), and form rubrus (smaller, generally orange-red, and shorter paramere); there are few other morphological characters to distinguish these forms, however there is notable genetic divergence (Fig. 3). This is one of the more common species on Kauai and is closely related to Pariaconus crassiorcalix, which makes a thick-walled cup gall on stems of more pubescent morphotypes such as bog ohia in Alakai Swamp. Morphologically, both these species are close to Pariaconus lehua (Crawford, 1925).

###### Type material.

Holotype male (slide mounted, BMNH). See Table 2 for details of type and other material examined for this study.

##### Pariaconus crassiorcalix

Taxon classificationAnimaliaHemipteraTriozidae

Percy sp. n.

http://zoobank.org/74C0156B-542E-453C-9290-5CC69FB7A33B

Figures 26 , 49K–R

###### Adult colour.

**Figure 26.**
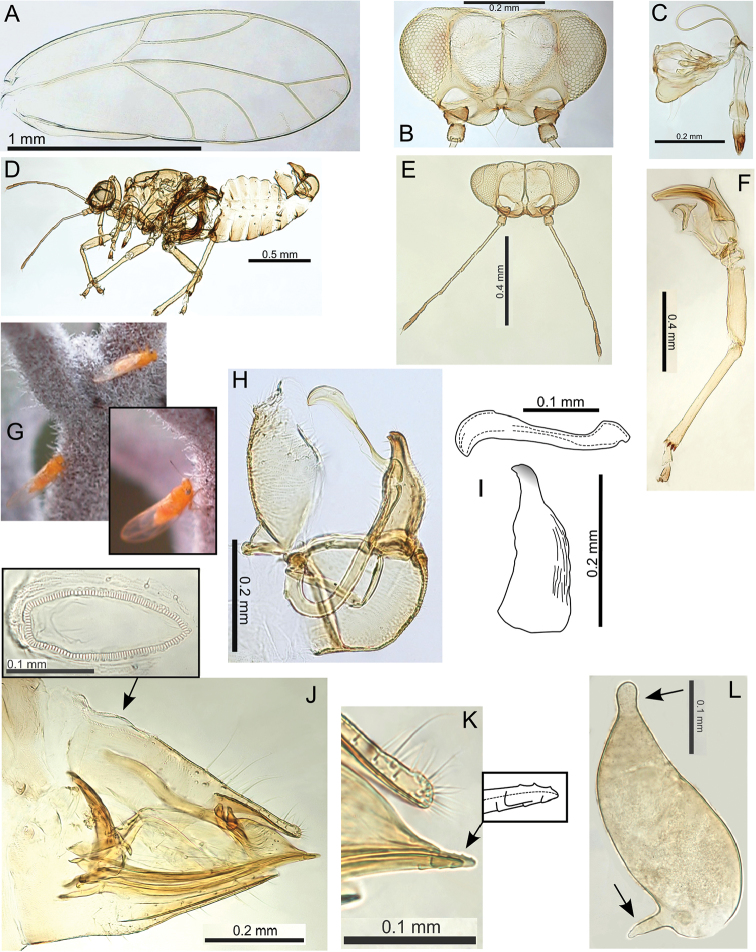
Pariaconus crassiorcalix sp. n. **A** fore wing **B** head **C** proboscis **D** male **E** head and antenna **F** hind leg **G** adults on pubescent host morphotype **H** male terminalia **I** aedeagus and paramere **J** female terminalia showing anal ring cells (inset) **K** ovipositor (serrations indicated) **L** egg (pedicel and tail indicated).

General body colour pale yellow to orange (Fig. 26G). Fore wing membrane clear or slightly fuscous.

###### Adult structure.

Fore wing apex bluntly acute; surface spinules dispersed, usually present in all cells but may be limited or absent; short setae on margins and veins (Fig. 26A). Antennae short (length av. 0.70; ratio AL:HW av. 1.42); genal processes short (ratio VL:GP av. 2.92) and rounded apically; medium long setae on vertex and short setae on thorax; distal proboscis segment short (av. length 0.09); hind tibia slender, length subequal to head width (ratio HW:HT av. 1.02) (Fig. 26B–F). Male terminalia (Fig. 26H–I): paramere shorter than proctiger (ratio MP:PL av. 1.15), broad and parallel-sided before tapering to an elongate neck below a somewhat flat-topped apex with anteriorly directed hook; distal aedeagus segment length subequal to paramere (ratio PL:AEL av. 0.98) with base slightly angular and inflated, and a moderately large hooked apex (ratio AEL:AELH av. 2.5). Female terminalia (Fig. 26J–K): proctiger dorsal surface more or less straight, apex bluntly acute, anal ring long (ratio FP:RL av. 3.97); subgenital plate with slight medial bulge ventrally, bluntly acute apically; ovipositor apex with reduced serrations (2-3 above, 2-3 below), valvulae dorsalis not strongly convex dorsally.

###### Egg.

Light brown, short, slightly sinusoidal, surface granular in appearance, no microsculpturing, short pedicel 1/4 length from base, tail bulbous (Fig. 26L).

###### Immature.

Colour and structure: Brown to orange-brown dorsally, cream to orange ventrally. 5^th^ instar: Broadly ovoid in outline (but narrower than Pariaconus caulicalix), wing buds only slightly protruding with distinct humeral lobes (Fig. 49K–L). Dorsal surface with round tubercle like scales (as opposed to ridges in Pariaconus caulicalix) (Fig. 49N). Tarsi with medium large claws (as wide as arolia) (Fig. 49M). Circumanal ring moderately wide (CPW:RW av. 5.00), shallowly v-shaped, with a single row of elongate cells (Fig. 49K). Chaetotaxy: 5^th^ instar: Continuous marginal ring of blunt sectasetae (Fig. 49O). Dorsal surface with intermittent minute simple setae. 1^st^ instar (similar to that illustrated for Pariaconus caulicalix): Margin with broad blunt sectasetae (10 pairs anterior margin of head, 1 pair postocular, 1 pair each wing bud, 10 pairs abdominal), and with distinct arrangement of acutely pointed small sectasetae dorsally (2-4 pairs on head, 2-4 pairs on thorax, 2-5 pairs on the abdomen), mostly lost by 3^rd^-4^th^ instars, but minute sectasetae are scattered dorsally in some older instars.

###### Host plant notes.

Only known from one locality where it galls densely pubescent bog morphotypes.

###### Island.

Kauai.

###### Distribution notes.

Known only from Alakai Swamp, Kokee State Park.

###### Biology.

Forms thick-walled cup galls on stems, galls are often clustered together, with one individual per gall chamber (Fig. 49P–R). The depression forming the cup gall is not as deep as for Pariaconus caulicalix, the cup walls are much thicker and the gall tissue is typically dark red or orange-brown. The immature is lodged tightly in the base of the cup with the sclerotized dorsal surface forming a plug under which is the soft unsclerotized body (Fig. 49R). The different structure of the dorsal surface, scaly in Pariaconus crassiorcalix and ridged in Pariaconus caulicalix, may be related to adaptation to different moisture levels, with Pariaconus crassiorcalix found in more humid, wet bog habitat.

###### Etymology.

The name refers to the gall type which is a thick (*crassior*)-walled and cup (*calix*)-shaped cambial outgrowth on the plant stems (adjective in the nominative singular).

###### Comments.

This species is a sister taxon to the other known cup gall maker, Pariaconus caulicalix, and, together with Pariaconus elegans, Pariaconus gagneae, Pariaconus haumea, ﻿and Pariaconus lehua, for which biologies are currently unknown, may constitute a sub-clade of cup gallers. A similar type of thick walled cup gall (Fig. 48N–O, referred to as “a raised button gall on the stem”, Russell Messing pers. comm.) is produced by an immature with long waxy dorsal filaments, which may be the immature of one of the described species here, or an as yet undescribed species.

###### Type material.

Holotype male (slide mounted, BMNH). See Table 2 for details of type and other material examined for this study.

##### Pariaconus lehua

Taxon classificationAnimaliaHemipteraTriozidae

(Crawford, 1925) comb. n.

Figure 27

Trioza lehua Crawford, 1925: 29

###### Adult colour.

General body colour yellow or orange. Fore wing membrane clear.

###### Adult structure.

**Figure 27.**
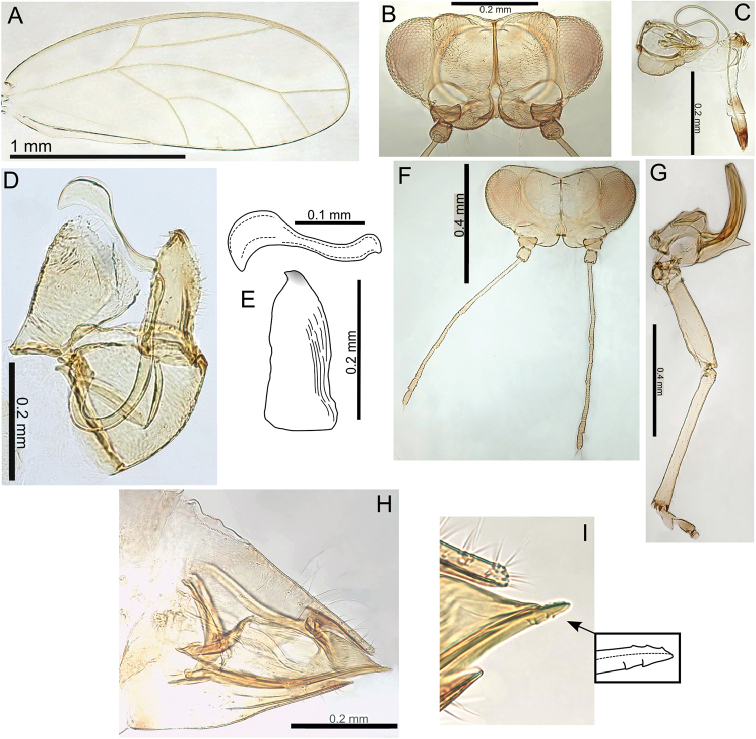
Pariaconus lehua. **A** fore wing **B** head **C** proboscis **D** male terminalia **E** aedeagus and paramere **F** head and antennae **G** hind leg **H** female terminalia **I** ovipositor (serrations indicated).

Fore wing apex rounded; surface spinules distributed in all cells; short setae on margins and veins (Fig. 27A). Antennae short (length av. 0.81; ratio AL:HW av. 1.62); genal processes short (ratio VL:GP av. 2.30) and rounded apically; medium short setae on vertex and short setae on thorax; distal proboscis segment short (av. length 0.08); hind tibia slender, length subequal to head width (ratio HW:HT av. 1.02) (Fig. 27B–C, F–G). Male terminalia (Fig. 27D–E): paramere slightly shorter than proctiger (ratio MP:PL av. 1.08), broad and parallel-sided before tapering to a somewhat flat-topped apex with anteriorly directed hook; distal aedeagus segment length subequal to paramere (ratio PL:AEL av. 1.04) with base slightly angular and inflated, and a large hooked apex (ratio AEL:AELH av. 2.30). Female terminalia (Fig. 27H–I): proctiger dorsal surface more or less straight, apex bluntly acute, anal ring long (ratio FP:RL av. 3.50); subgenital plate with slight medial bulge ventrally, and acute apex; ovipositor apex with reduced serrations (2 above, 2 below), valvulae dorsalis not strongly convex dorsally.

###### Egg.

Unpigmented, not sinusoidal, smooth, no microsculpturing, short pedicel 1/4 length from base, tail lacking.

###### Immature.

Unknown (see comment under Pariaconus crassiorcalix).

###### Host plant notes.

Unknown.

###### Island.

Kauai

###### Distribution notes.

The type location is recorded only as “Nualolo”.

###### Biology.

The biology of this species is unknown, it may form cup galls on stems as morphologically it is close to the two stem cup-gallers, Pariaconus caulicalix and Pariaconus crassiorcalix.

###### Type material.

Holotype, male (?) (dry mounted, damaged, abdomen and fore wings missing, BPBM). See Table 2 for details of type and other material examined for this study.

##### Pariaconus elegans

Taxon classificationAnimaliaHemipteraTriozidae

Percy sp. n.

http://zoobank.org/0DE38EDF-A0E9-4CE4-A89D-EC9E0533DF74

Figure 28

###### Adult colour.

General body colour brown. Fore wing membrane clear.

###### Adult structure.

**Figure 28.**
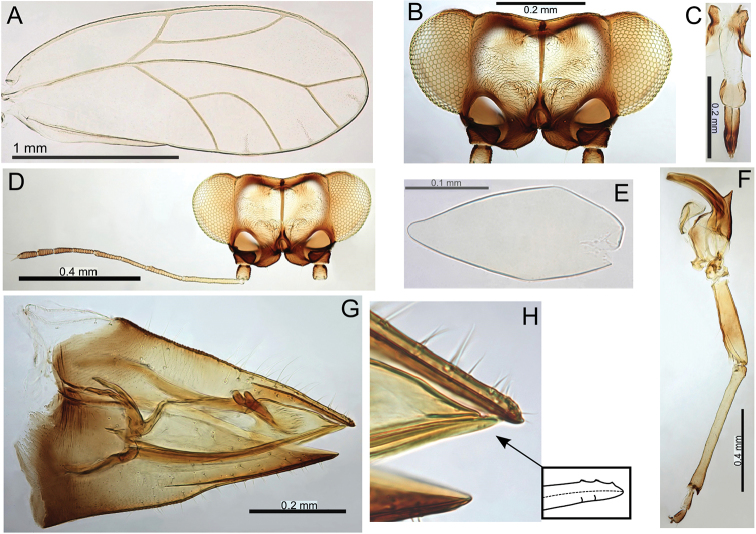
Pariaconus elegans sp. n. (female) **A** fore wing **B** head **C** proboscis **D** head and antenna **E** egg **F** hind leg **G** female terminalia **H** ovipositor (serrations indicated).

Fore wing apex bluntly acute; surface spinules sparsely distributed, few or none in cells r_1_, cu_2_, c+sc; short setae on margins and veins (Fig. 28A). Antennae short (length 0.74; ratio AL:HW 1.28); genal processes short (ratio VL:GP 3.00) and rounded apically; medium short setae on vertex and short setae on thorax; distal proboscis segment medium-short (length 0.12); hind tibia slender and longer than head width (ratio HW:HT 0.92) (Fig. 28B–D, F). Female terminalia (Fig. 28G–H): proctiger long, dorsal surface more or less straight, apex acute, anal ring long (ratio FP:RL 3.89); subgenital plate with slight medial bulge ventrally, acute apically; ovipositor apex with very reduced serrations (0-2 above, 0-2 below), valvulae dorsalis not strongly convex dorsally.

###### Egg.

Unpigmented, short, not sinusoidal, no microsculpturing, pedicel not visible, tail lacking (Fig. 28E).

###### Immature.

Unknown.

###### Host plant notes.

Collected from glabrous morphotype.

###### Island.

Kauai.

###### Distribution notes.

Only known location is Kalalau Valley, Kokee State Park.

###### Biology.

Unknown.

###### Etymology.

The name refers to the small and elegant appearance with slender elongate female terminalia and long, slender tibiae (adjective in the nominative singular).

###### Comments.

Known from only one female specimen; the distinctly long, slender terminalia is unlike any other described species.

###### Type material.

Holotype female (slide mounted, BMNH). See Table 2 for details of type material examined for this study.

##### Pariaconus gagneae

Taxon classificationAnimaliaHemipteraTriozidae

Percy sp. n.

http://zoobank.org/975FCBC7-1668-49A3-9EA9-F4CEEEF807C3

Figure 29

###### Adult colour.

General body colour yellow with darker yellow-brown dorsally. Fore wing membrane clear or slightly fuscous basally.

###### Adult structure.

**Figure 29.**
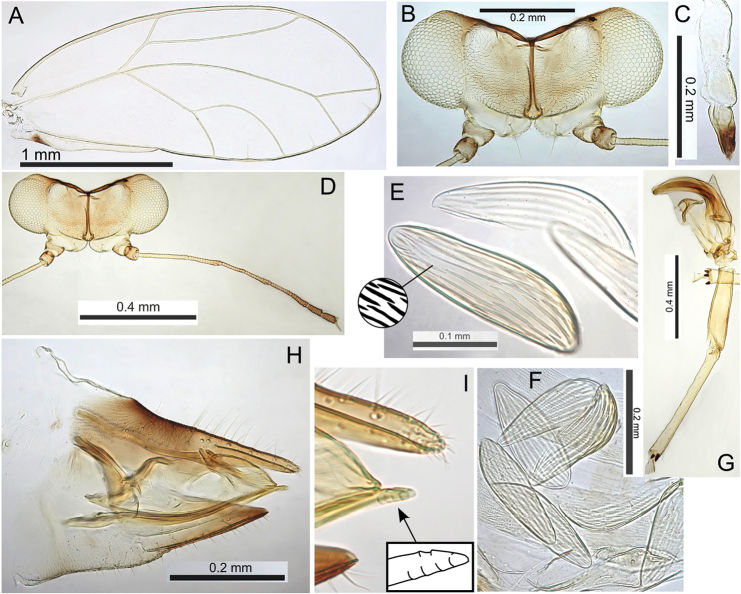
Pariaconus gagneae sp. n. (female) **A** fore wing **B** head **C** proboscis **D** head and antenna **E, F** eggs (striations indicated) **G** hind leg **H** female terminalia **I** ovipositor (serrations indicated).

Fore wing apex bluntly acute; surface spinules sparsely distributed, absent from r_1_ and c+sc; short to minute setae on margins and veins (Fig. 29A). Antennae short (length 0.75; ratio AL:HW 1.36); genal processes short (ratio VL:GP 3.50) and rounded apically; short to minute setae on vertex and thorax; distal proboscis segment short (length 0.09); hind tibia slender and longer than head width (ratio HW:HT 0.94) (Fig. 29B–D, G). Female terminalia (Fig. 29H–I): proctiger long, dorsal surface depressed posterior to anal ring and then more or less straight, apex acute, anal ring long (ratio FP:RL 2.68); subgenital plate with slight medial bulge ventrally, acute apically; ovipositor apex with very reduced serrations (2–3 above, 3 below), valvulae dorsalis moderately convex dorsally.

###### Egg.

Unpigmented to light brown, long and narrow, not sinusoidal, surface with broadly spaced longitudinal striations that are either continuous or interrupted, pedicel appears to be absent, tail lacking (Fig. 29E–F).

###### Immature.

Unknown.

###### Host plant notes.

Morphotype preference unknown.

###### Island.

Kauai.

###### Distribution notes.

Only known location is Kalalau Valley, Kokee State Park.

###### Biology.

Unknown.

###### Etymology.

Named after Betsy Gagné to honour her role in promoting biodiversity research, entomology, and conservation in the Hawaiian Islands (noun in the genitive case).

###### Comments.

Known from only one female specimen; the distinctly shaped female terminalia and egg characteristics are not found in other species.

###### Type material.

Holotype female (slide mounted, BMNH). See Table 2 for details of type material examined for this study.

##### Pariaconus haumea

Taxon classificationAnimaliaHemipteraTriozidae

Percy sp. n.

http://zoobank.org/60513D00-EE6F-471C-8FD6-B614311F41E5

Figure 30

###### Adult colour.

General body colour yellow to pale brown. Fore wing membrane clear or slightly fuscous.

###### Adult structure.

**Figure 30.**
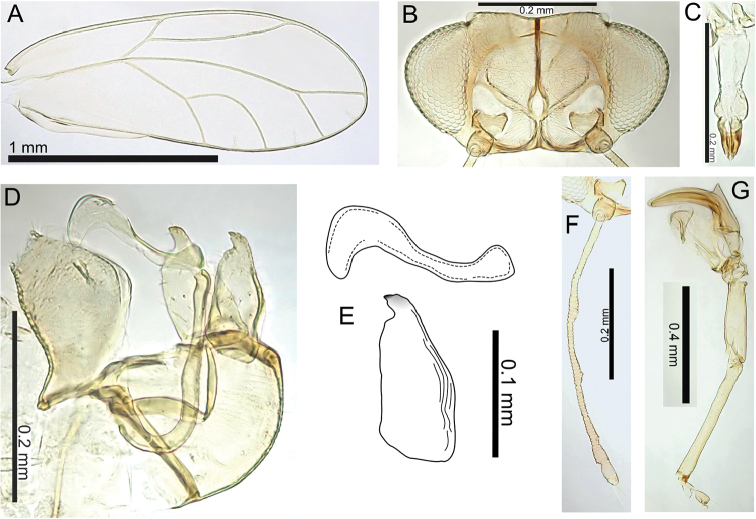
Pariaconus haumea sp. n. **A** fore wing **B** head **C** proboscis **D** male terminalia **E** aedeagus and paramere **F** antenna **G** hind leg.

Fore wing apex bluntly acute to almost rounded; surface spinules sparsely distributed, absent from r_1_ and c+sc; short to minute setae on margins and veins (Fig. 30A). Antennae very short (length 0.55; ratio AL:HW 1.24); genal processes short (ratio VL:GP 2.00) and rounded apically; short setae on vertex and thorax; distal proboscis segment very short (length 0.06); hind tibia slender, length subequal to head width (ratio HW:HT 1.04) (Fig. 30B–C, F–G). Male terminalia (Fig. 30D–E): paramere shorter than proctiger (ratio MP:PL 1.12), broad at base and tapering to a short neck below an apex with anteriorly directed hook; distal aedeagus segment longer than paramere (ratio PL:AEL av. 0.92) with base angular and inflated, and a large hooked apex (ratio AEL:AELH av. 2.18).

###### Egg.

Unknown.

###### Immature.

Unknown.

###### Host plant notes.

Collected from glabrous forest tree.

###### Island.

Kauai.

###### Distribution notes.

Only known location is Alakai, Kokee State Park, in forest near Alakai bog area.

###### Biology.

Unknown.

###### Etymology.

Named after Haumea in Hawaiian mythology, the Hawaiian goddess of fertility and mother of Pele (noun in the nominative singular standing in apposition to the generic name).

###### Comments.

Known from only one male specimen; the distinctly shaped male paramere is not found in other species; morphologically it appears most closely related to Pariaconus lehua.

###### Type material.

Holotype male (slide mounted, BMNH). See Table 2 for details of type material examined for this study.

#### ohialoha species group

The ohialoha species group is a monophyletic clade of species that makes enclosed galls on leaves, stems, and buds. This species group is found on all major islands except Kauai. The group is characterized by typically longer, more acute genal cones (with the exception of Pariaconus pyramidalis), longer and usually more slender parameres, and eggs that are distinct for being short, broad, darkly pigmented, lacking surface striations but with microsculpturing, and with pedicels usually long and only slightly off set from base; female abdomens, especially of paler species, can appear much darker when carrying egg loads. Extensive sampling for some of the species in this group reveals that overall body size can be highly variable within species. The immatures are remarkably homogenous with few species specific distinguishing characters. A broad taxonomic approach at species level is combined with an attempt to illustrate the variation within species in this evolutionarily dynamic group.

Due to a high degree of intraspecific variation and inter-island convergence in the ohialoha species group, keys are provided for each island independently (with the exception of Lanai for which insufficient material is available).

#### Adult key to Pariaconus species in the ohialoha species group found on Oahu

**Table:** 

1	Generally larger species (WL av. 2.96 mm), fore wing generally broader (ratio WL:WW av. 2.54) with broadly rounded apex, male with broader paramere (width av. 0.10 mm), shape of male and female terminalia as in Figs 32–33 (makes closed galls usually on stems and buds, occasionally leaves)	**Pariaconus oahuensis sp. n.**
–	Generally smaller species (WL av. 2.64 mm), fore wing generally narrower (ratio WL:WW av. 2.94) with typically more acute apex, male with more slender paramere (width av. 0.06 mm), shape of male and female terminalia as in Fig. 35 (makes flat leaf galls)	**Pariaconus ohiacola (Crawford, 1918), comb. n.**

#### Adult key to Pariaconus species in the ohialoha species group found on Molokai

**Table:** 

1	Generally larger species, male with longer, broader paramere (ratio PL:HW >0.40) and aedeagus hook not developed, female with longer terminalia (ratios FP:HW >0.85 and FP:RL >5), shape of male and female terminalia as in Fig. 36	**Pariaconus molokaiensis (Crawford, 1927), comb. n.**
–	Generally smaller species, male with shorter, narrower paramere (ratio PL:HW <0.40) and aedeagus hook well developed, female with shorter terminalia (ratios FP:HW <0.85 and FP:RL <5), shape of male and female terminalia as in Fig. 37	**Pariaconus hualani sp. n.**

#### Adult key to Pariaconus species in the ohialoha species group found on Maui.

**Table:** 

1	Smaller species with short genal processes (GP <0.15 mm, ratio VL:GP >1.8), male with shorter paramere (PL <0.24 mm, ratios PL:HW ≤0.41 and PL:AEL >2.40), shape of male and female terminalia as in Fig. 40	**Pariaconus montgomeri sp. n.**
	Larger species with long genal processes (GP >0.15 mm, ratio VL:GP <1.2), male with longer paramere (PL >0.24 mm, ratios PL:HW ≥0.41 and PL:AEL <2.40)	**2**
2	Female terminalia shorter (FP <0.60 mm, ratio FP:HW <0.90), shape of male and female terminalia as in Fig. 38	**Pariaconus mauiensis sp. n.**
	Female terminalia longer (FP >0.60 mm, ratio FP:HW >0.90), shape of male and female terminalia as in Fig. 39	**Pariaconus kupua sp. n.**

#### Adult key to Pariaconus species in the ohialoha species group found on Hawaii

**Table:** 

1	Intermediate sized species, male with shorter paramere (PL ≤0.24 mm), distal aedeagus segment apex distinctly hooked and hook with acute apex, female proctiger shorter (FP ≤0.54 mm), shape of male and female terminalia as in Fig. 43 (makes flat leaf galls)	**Pariaconus pele sp. n.**
	Larger or smaller species, male with longer paramere (PL ≥0.24 mm), distal aedeagus segment apex shallowly or barely hooked and hook with blunt apex, female proctiger longer (FP ≥0.54 mm)	**2**
2	Larger species, longer antennae (AL >1.20 mm, ratio AL:HW ≥1.9) with longer genal processes (GP >0.15 mm, ratio VL:GP <1.50), female proctiger shorter (ratio FP:HW <0.95), shape of male and female terminalia as in Fig. 41 (galls stems and buds)	**Pariaconus hawaiiensis (Crawford, 1918), comb. n.**
	Smaller species, shorter antennae (AL ≤1.20 mm, ratio AL:HW <1.9) with shorter genal processes (GP <0.15 mm, ratio VL:GP >1.50), female proctiger longer (ratio FP:HW ≥0.95), shape of male and female terminalia as in Fig. 44 (makes cone leaf galls, occasionally stem galls)	**Pariaconus pyramidalis sp. n.**

##### Pariaconus oahuensis

Taxon classificationAnimaliaHemipteraTriozidae

Percy sp. n.

http://zoobank.org/9C81B080-8746-424B-88B3-DC9DDBFD986A

**Figure 31.**
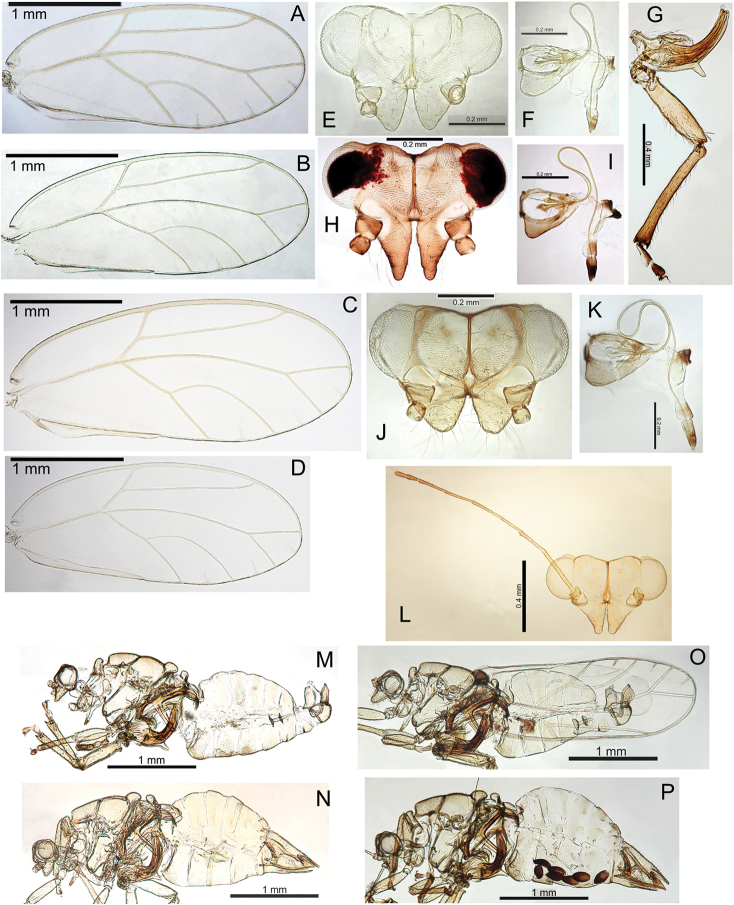
Pariaconus oahuensis sp. n. **A, B, C, D** fore wing: **A** form oahuensis **B** form tenuis **C** form latus (Waianae, Oahu) **D** form latus (Koolau, Oahu) **E, F** form oahuensis (Northern Waianae, Oahu): **E** head **F** proboscis **G, H, I** form oahuensis (Central Waianae, Oahu): **G** hind leg **H** head (uncleared ocular tissue) **I** proboscis **J, K** form latus (Waianae, Oahu): **J** head **K** proboscis **L** head and antenna, form oahuensis (Aiea, Oahu) **M** male, form oahuensis (Aiea, Oahu) **N** female, form tenuis (Koolau, Oahu) **O** male, form latus (Koolau, Oahu) **P** female, form latus (Aiea, Oahu).

**Figure 32.**
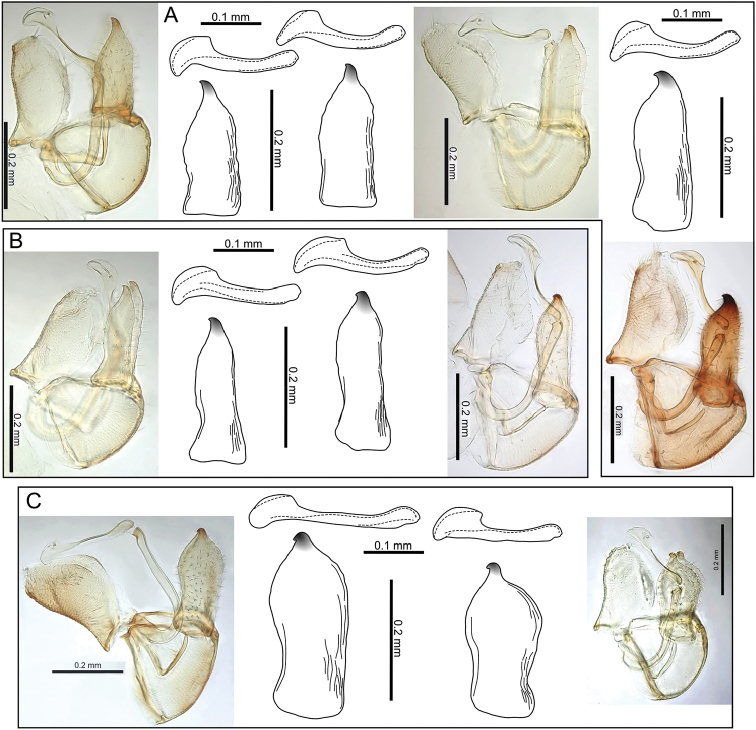
Pariaconus oahuensis sp. n. (males) **A, B, C** terminalia, aedeagus and paramere: **A** form oahuensis 3 variations: left and centre (Aiea, Oahu), right (Waianae, Oahu) **B** form tenuis 2 variations (Waianae, Oahu) **C** form latus 2 variations: left (Waianae, Oahu), right (Koolau, Oahu).

**Figure 33.**
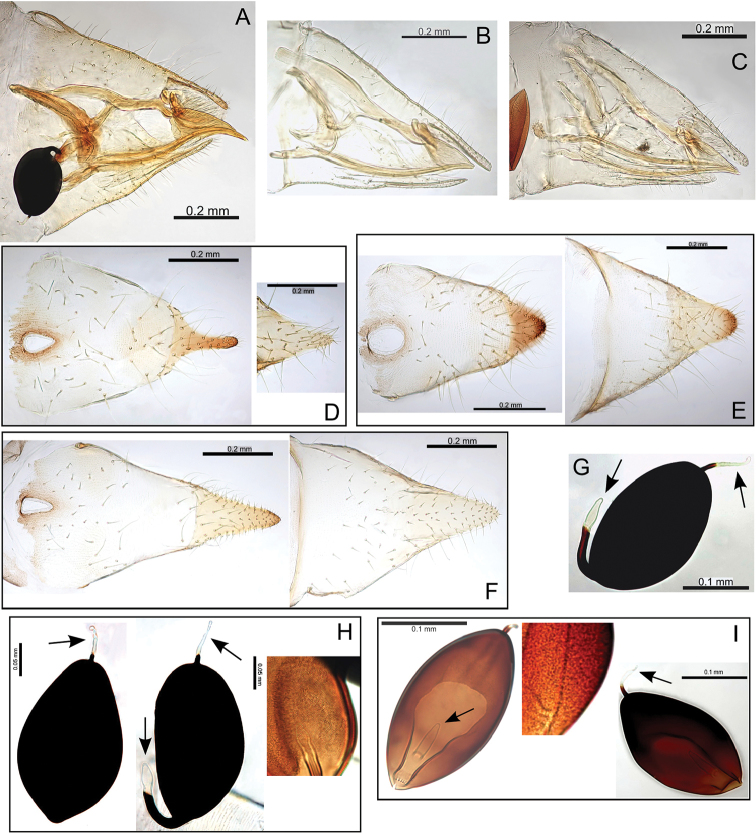
Pariaconus oahuensis sp. n. (females) **A, B, C** terminalia: **A** form oahuensis **B** form tenuis **C** form latus **D, E, F** proctiger (dorsal view) and subgenital plate (ventral view) **D** form tenuis (subgenital plate apex only) **E** form oahuensis **F** form latus **G, H, I** eggs (pedicel and tail indicated, microsculpturing detailed): **G** form tenuis **H** form oahuensis **I** form latus.

Figures 31 , 32 , 33 , 50A–D, F, I–L

Trioza iolani Kirkaldy, 1902: 114, in part (Oahu specimens, nec Kauai specimens)
Trioza iolani sensu Crawford, 1918: 441, 1925: 27
Trioza iolani sensu Zimmerman, 1948: 21

###### Adult colour.

General body colour yellow-green to yellow-brown. Females often appear to have a dark abdomen due to darkly pigmented egg load. Fore wing membrane clear.

###### Adult structure.

Fore wing apex rounded; spinules distributed in all cells, but few in r_1_; medium to long setae on margins and veins (Fig. 31A–D). Antennae long (av. length 1.31; ratio AL:HW av. 2.02); genal processes medium-long (ratio VL:GP av. 1.62), and apically bluntly acute (forms oahuensis and tenuis) or rounded (form latus); medium-long setae on vertex and thorax; distal proboscis segment short (length 0.11); hind tibia longer than or subequal to head width (ratio HW:HT av. 0.90) (Fig. 31E–P). Male terminalia (Fig. 32A–C): paramere length subequal to or longer than proctiger (ratio MP:PL av. 0.82), broad at the base and more or less parallel-sided (f. oahuensis), tapering (f. tenuis), or inflated medially (f. latus) before constricting to a short neck below an apex with anteriorly directed hook; distal aedeagus segment shorter than paramere (ratio PL:AEL av. 1.18) with base rounded and slightly inflated, and a shallow hooked apex (ratio AEL:AELH av. 2.57). Female terminalia (Fig. 33A–F): proctiger long, dorsal surface more or less straight or slightly convex, apex acute to bluntly acute, anal ring extremely short (ratio FP:RL av. 7.55); subgenital plate with slight medial bulge ventrally, acute to bluntly acute apically; ovipositor apex lacking serrations, valvulae dorsalis moderately or slightly convex dorsally.

###### Egg.

Short, broad, pigmented brown to dark brown (except tip of pedicel and tail) with surface microsculpturing, either with long pedicel and tail, the pedicel with an inflated tip (forms oahuensis and tenuis), in form latus eggs are more slender, lighter in colour with finer surface microsculpturing, a much shorter pedicel without inflated tip, and an unsclerotized patch at the base of the egg likely in the position where the egg contacts the plant surface (in populations making cone leaf galls, the tail is extremely short and there appears to be no pedicel) (Fig. 33G–I).

###### Immature.

Colour and structure: 5^th^ instar: Cream to orange. Elongate ovoid in outline, wing buds protruding with moderate humeral lobes (Fig. 50A, C). Tarsi with large claws (Fig. 50B). Circumanal ring small (CPW:RW av. 24.40), u-shaped with a single row of sometimes interrupted cells (Fig. 50D). 1^st^ instars have a scaly dorsal surface (Fig. 50F). Chaetotaxy: 2^nd^-5^th^ instars: Head, thorax and abdomen with scattered long to medium-long simple setae. 1^st^ instar (Fig. 50F): Setal arrangement similar to those in the bicoloratus group; marginal sectasetae narrow, anterior margin of the head with simple setae, a single pair of post ocular sectasetae, a single pair of sectasetae on the apices of each wing bud, and the margin of the abdomen with 8-10 pairs of sectasetae.

###### Host plant notes.

Primarily on more pubescent morphotypes.

###### Island.

Oahu.

###### Distribution notes.

The distribution ranges of the three recognized forms overlap, all three are found in both Waianae and Koolau ranges, but rarely at the same collection site suggesting microecological divergence may play a role in explaining this diversity; form oahuensis is the most widespread, form tenuis and form latus are generally less common; however form tenuis appears to be the more common in the southern Waianae and form latus the more common in the southern Koolau, and therefore initial divergence may have taken place allopatrically between the two primary mountain ranges, with subsequent expansion and secondary overlap of ranges.

###### Biology.

Usually galls stems, buds and petioles resulting in irregular swellings (Fig. 50I), but a localized population in the northern Koolau Mnts makes distinct cone galls on leaves resembling those of Pariaconus pyramidalis (Hawaii) but with a different opening mechanism (4-5 valves opening from the apex of the gall on the lower leaf surface, versus a circular suture and trap-door opening on the upper leaf surface in Pariaconus pyramidalis, compare Fig. 50J–L with Fig. 52T–X). See discussion on lability of galling biology. Dense clusters of gall chambers in bud galls can yield >10 immatures per bud.

###### Etymology.

Named for its distribution on the island of Oahu (noun in the genitive case).

###### Comments.

One of the commonest species on Oahu. It can be distinguished most easily from other Oahu species by its typically much larger size and predominantly yellow-green or yellow-brown colour. This species encompasses Trioza iolani sensu Crawford (1918, 1925) and Zimmerman (1948), see comments under Pariaconus iolani (Kirkaldy). Three forms are recognized (Figs 31–33): form oahuensis (based on the type, with variably broad paramere, and shorter, more apically blunt female terminalia), form tenuis (narrower paramere, long slender female terminalia constricted apically), and form latus (with the broadest paramere that is somewhat medially expanded, long female terminalia not apically constricted, and eggs with unsclerotized base), this latter form apparently also includes the populations that produce cone galls on leaves in the northern Koolau Mnts.

###### Type material.

Holotype male (slide mounted, BMNH). See Table 2 for details of type and other material examined for this study.

##### Pariaconus ohiacola

Taxon classificationAnimaliaHemipteraTriozidae

(Crawford, 1918) comb. n.

**Figure 34.**
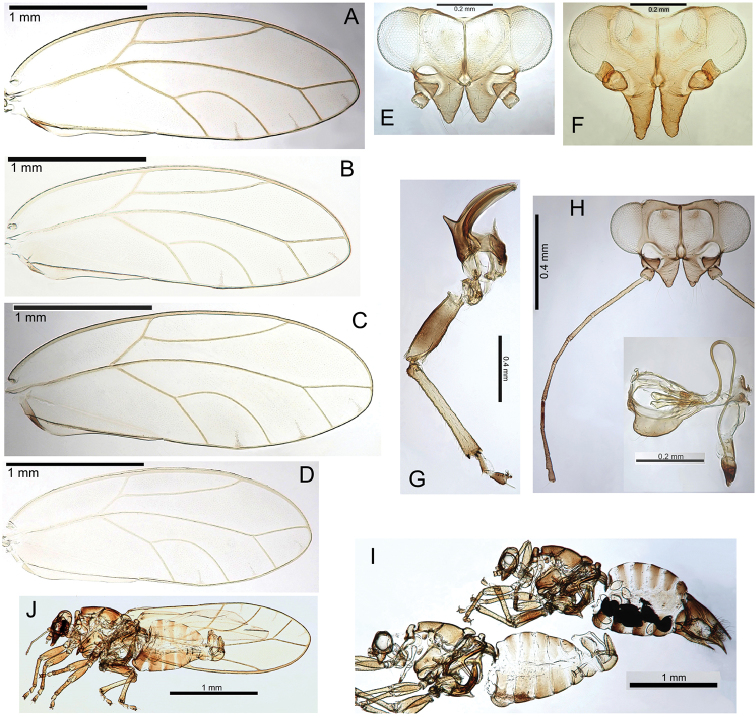
Pariaconus ohiacola. **A, B, C, D** fore wing: **A** form ohiacola **B** form angustipterus **C** form obtusipterus **D** form waianaiensis **E, F** head: **E** form ohiacola **F** form waianaiensis **G, H, I** form ohiacola: **G** hind leg **H** head and antenna with proboscis (inset) **I** male and female **J** male, form waianaiensis.

**Figure 35.**
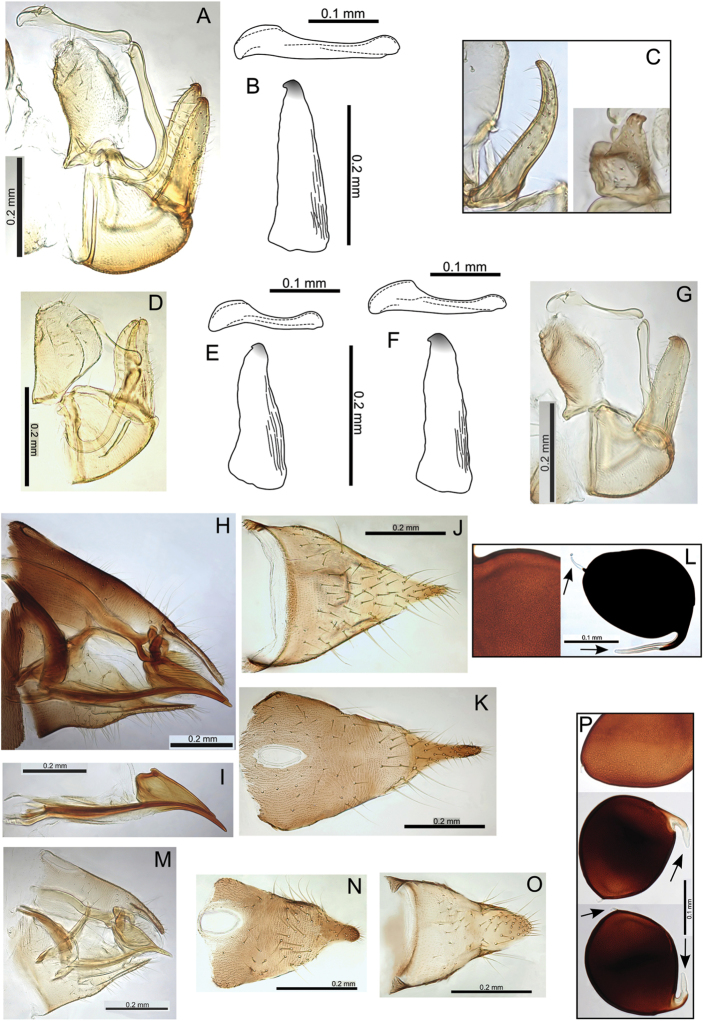
Pariaconus ohiacola. **A, B, C** form ohiacola: **A** male terminalia **B** aedeagus and paramere **C** paramere posterior view (left) dorsal view (right) **D, E** form waianaiensis: **D** male terminalia **E** aedeagus and paramere **F, G** form obtusipterus: **F** aedeagus and paramere **G** male terminalia **H, I, J, K, L** form ohiacola: **H** female terminalia **I** ovipositor **J** female subgenital plate (ventral view) **K** female proctiger (dorsal view) **L** eggs (pedicel and tail indicated, microsculpturing detailed) **M, N, O, P** form waianaiensis: **M** female terminalia **N** female proctiger (dorsal view) **O** female subgenital plate (ventral view) **P** eggs (pedicel and tail indicated, microsculpturing detailed).

Figures 34 , 35 , 50E, G–H, M–R

Trioza ohiacola Crawford, 1918: 442

###### Adult colour.

General body colour red-brown to orange-brown. Females often appear to have a dark abdomen due to darkly pigmented egg load. Fore wing membrane clear or fuscous.

###### Adult structure.

Fore wing apex acute to bluntly acute; spinules distributed in all cells; short to medium-short setae on margins and veins (Fig. 34A–D). Antennae medium-long (av. length 1.02; ratio AL:HW av. 1.76); genal processes medium-long to long (ratio VL:GP av. 1.66), and acute; medium-long setae on vertex and thorax; distal proboscis segment short (av. length 0.08); hind tibia slightly shorter than head width (ratio HW:HT av. 1.05) (Fig. 34E–J). Male terminalia (Fig. 35A–G): paramere longer than proctiger (ratio MP:PL av. 0.87), slender, broader at base and tapering evenly to apex with anteriorly directed hook; distal aedeagus segment shorter or subequal to paramere (ratio PL:AEL av. 1.15) with base rounded and slightly inflated, and a shallow hooked apex (ratio AEL:AELH av. 2.35). Female terminalia (Fig. 35H–K, M–O): proctiger long, dorsal surface convex, apex acute to bluntly rounded, anal ring short (ratio FP:RL av. 4.83); subgenital plate with slight to moderate medial bulge ventrally, acute to bluntly acute apically; ovipositor apex lacking serrations, valvulae dorsalis not strongly convex dorsally. Egg short, broad, pigmented dark brown (except tip of pedicel and tail), surface with dense microsculpturing, extremely long to moderately short pedicel with no or slightly inflated tip, tail medium-long to short (Fig. 35L, P).

###### Immature.

Colour and structure: 2^nd^-5^th^ instars: Orange or orange-red with cream wing buds. Elongate ovoid in outline, wing buds protruding with moderate humeral lobes (Fig. 50R). Tarsi with large claws. Circumanal ring small (CPW:RW av. 21.78), u-shaped with a single row of often interrupted cells (Fig. 50E). 1^st^ instar (Fig. 50Q): yellow-brown with scaly dorsal surface. Chaetotaxy: 2^nd^-5^th^ instars: Head, thorax and abdomen with scattered long to medium-long simple setae. 1^st^ instar (Fig. 50G–H): Margin with broad fan-shaped setae (anterior of head with 5-6 pairs, a single pair post ocular, a single pair on the apices of each wing bud, and 7-8 pairs on the abdomen).

###### Host plant notes.

Mostly associated with glabrous morphotypes.

###### Island.

Oahu.

###### Distribution notes.

A widespread taxon and probably the most commonly encountered on Oahu, but as with Pariaconus oahuensis appears to be undergoing incipient divergence. Forms ohiacola and obtusipterus are the most widespread, found in the Waianae, Aiea, and Koolau mountain regions; form waianaiensis is currently only known from the central Waianae Mnts; form angustipterus is most common in the southern Waianae Mnts, but also occurs in the southern Koolau region.

###### Biology.

Makes flat leaf galls. 1^st^ instars are found in very shallow pits (Fig. 50Q), usually on the lower surface of young leaves that are often still in bud, the leaf tissue around the instar often becomes red or brown. By the 2^nd^ instar there is complete enclosure, generating a flat leaf gall type, often with a slight central depression on the lower surface where the original 1^st^ instar pit was located (Fig. 50O); the 1^st^ instar exuviae are often found with 2^nd^ instars in the gall chamber. The scaly sclerotization on the dorsal surface of 1^st^ instars of the ohialoha group (Fig. 50F) may prevent dehydration during the period when the 1^st^ instar is on the leaf surface before gall enclosure.

###### Comments.

Four forms are recognized (Figs 34–35): form ohiacola (based on the type has fore wing with apex acute, medium long genae, long paramere, long female terminalia that is apically acute, eggs ovoid with long pedicel), form angustipterus (fore wing more narrow with apex acute), form obtusipterus (largest form, with fore wing apex rounded, shorter genae, long female terminalia), and form waianaiensis (smallest form, with fore wing apex bluntly acute, notably long thin genae, paramere slender, short female terminalia, eggs almost round with short pedicel). In some cases these forms correspond to distinct clusters in the DNA analysis (Fig. 3) and may be good candidates for species recognition with further study of specimens and biology. However, there is substantial variation in fore wing shape, genal process length, and female and male terminalia that are intermediate between these forms, with different combinations found within and between populations making the overall observed pattern complex.

###### Type material.

Holotype, male (dry mounted, BPBM). See Table 2 for details of type and other material examined for this study.

##### Pariaconus lanaiensis

Taxon classificationAnimaliaHemipteraTriozidae

(Crawford, 1918) comb. n.

Trioza lanaiensis Crawford, 1918: 443

###### Comments.

No new material was collected during this study. Below is a summary of the description from Crawford (1918) who considered this species closely related to Pariaconus pullatus, yet he also describes it as being “an incipient, not clearly marked, species developing from the Oahuan species [Pariaconus oahuensis]”; he suggests that the divergence of Pariaconus lanaiensis and Pariaconus pullatus may reflect a parallel process to that seen on Oahu between Pariaconus oahuensis and Pariaconus ohiacola (Crawford 1918). Interestingly, Crawford (1918) reports considerable intraspecific variation in size, noting “it is quite possible that in time these variations will break the species into several distinct ones”, and this also reflects the patterns of variation observed on Oahu, Molokai, and Maui.

###### Adult colour and structure.

General body colour yellow to brown. Fore wing membrane clear or slightly fuscous, short setae on margins and veins. Reported size is similar to Pariaconus molokaiensis, but antennae are reported as up to 3× head width; genal processes long (longer than vertex); male paramere longer than proctiger; female terminalia long (subequal in length to abdomen).

###### Immature.

Unknown.

###### Host plant notes.

Metrosideros: “taken on foliage of ohia lehua” (Crawford 1918).

###### Island.

Lanai, Molokai.

###### Distribution notes.

Apparently common on Lanai (Crawford 1918), the distribution on Molokai (according to Crawford 1927) needs confirmation.

###### Biology.

Unknown, but it likely makes enclosed galls, and if Crawford’s hypothesis of parallel divergence to that on Oahu is correct, then this may be a stem galling sister taxon to a leaf galling Pariaconus pullatus.

###### Type material.

Holotype, female (dry mounted, BPBM). See Table 2 for details of type material examined for this study.

##### Pariaconus pullatus

Taxon classificationAnimaliaHemipteraTriozidae

(Crawford, 1918) comb. n.

Trioza pullata Crawford, 1918: 444

###### Comments.

No new material was collected during this study. Below is a summary of the description from Crawford (1918) who considered this, “an incipient species derived from Trioza lanaiensis”. Additional specimens are needed to test Crawford’s hypothesis that it may be a local or seasonal variant of Trioza laniaensis.

###### Adult colour and structure.

Generally body colour dark brown to black, probably the darkest of the ohialoha group. Fore wing membrane clear. Male unknown. Differs from Trioza lanaiensis in shorter antennae (up to 2× head width), and genal processes (subequal to vertex length), and a shorter Rs vein in fore wing.

###### Immature.

Unknown.

###### Host plant notes.

Probably Metrosideros. Original material was collected partly from Cyathodes (Ericaceae) and partly from an undesignated plant.

###### Island.

Lanai.

###### Distribution notes.

Known from two localities on Lanai: “Waiopao” (“Waiopaa, west side” in Zimmerman 1948) 29 Nov. 1916, and “undesignated, Dec. 1916 and Feb. 1917”

###### Biology.

Unknown, but it likely makes enclosed galls, and if Crawford’s hypothesis of parallel divergence to that on Oahu is correct (see comment for Pariaconus lanaiensis), then this may be a leaf galler.

###### Type material.

No type material was found at BPBM.

##### Pariaconus molokaiensis

Taxon classificationAnimaliaHemipteraTriozidae

(Crawford, 1927) comb. n.

Figures 36 , 51I

Trioza molokaiensis Crawford, 1927: 423

###### Adult colour.

General body colour green, or yellow-green to yellow-brown. Females often appear to have a dark abdomen due to darkly pigmented egg load. Fore wing membrane clear.

###### Adult structure.

**Figure 36.**
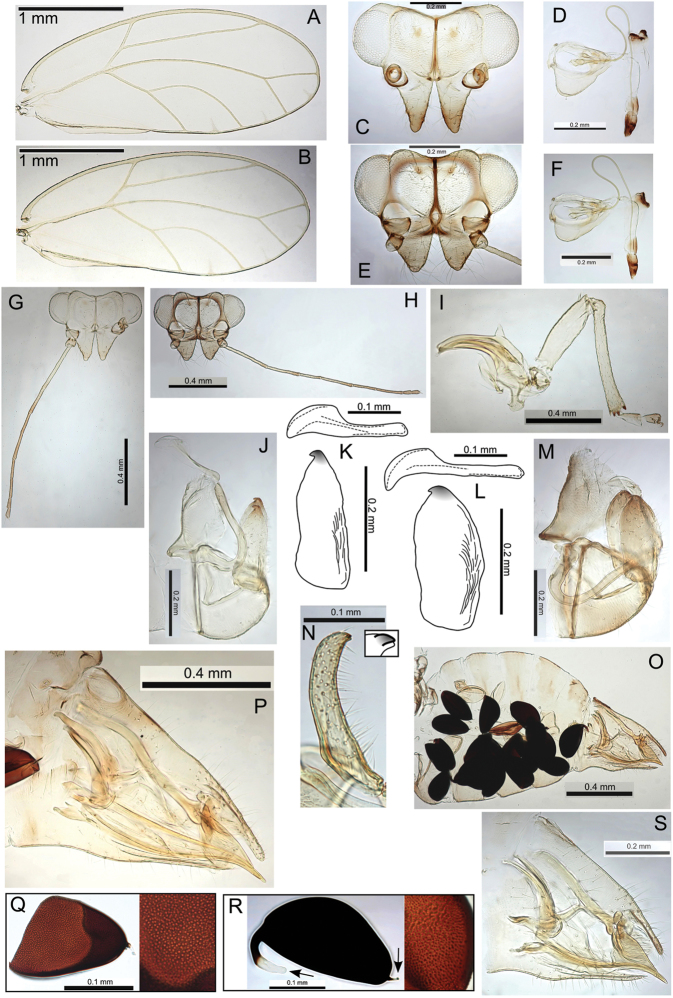
Pariaconus molokaiensis. **A, B** fore wing: **A** form molokaiensis **B** form laka **C, D** form molokaiensis: **C** head **D** proboscis **E, F** form laka: **E** head **F** proboscis **G, H** head and antenna: **G** form molokaiensis **H** form laka **I** hind leg **J, K** form molokaiensis: **J** male terminalia **K** aedeagus and paramere **L, M, N, O, P, Q** form laka: **L** aedeagus and paramere **M** male terminalia **N** paramere (posterior view) with apex detail (inset) **O** female abdomen (with eggs) and terminalia **P** female terminalia **Q** eggs showing microsculpturing **R, S** form molokaiensis: **R** eggs (pedicel and tail indicated, microsculpturing detailed) **S** female terminalia.

Fore wing apex rounded; spinules distributed in all cells, except few or none in r_1_; medium-short setae on margins and veins (Fig. 36A–B). Antennae long (av. length 1.34; ratio AL:HW av. 2.12); genal processes medium-long to long (ratio VL:GP av. 1.26), and acute apically; medium-long setae on vertex and thorax; distal proboscis segment short (length 0.11); hind tibia length subequal to head width (ratio HW:HT av. 0.94) (Fig. 36C–I). Male terminalia (Fig. 36J–N): paramere longer than proctiger (ratio MP:PL av. 0.86), broad at the base and more or less parallel-sided before tapering to apex with anteriorly directed hook; distal aedeagus segment shorter than paramere (ratio PL:AEL av. 1.12) with base rounded, not inflated, and a large shallow hooked apex (ratio AEL:AELH av. 2.16). Female terminalia (Fig. 36O–P, S): proctiger long, dorsal surface slightly to moderately convex, apex bluntly acute, anal ring extremely short (ratio FP:RL av. 6.39); subgenital plate with slight to moderate medial bulge ventrally, acute apically; ovipositor apex lacking serrations, valvulae dorsalis not strongly convex dorsally.

###### Egg.

Short, broad, pigmented brown to dark brown (except tip of pedicel and tail) with surface microsculpturing, medium-long pedicel with slightly inflated tip, tail short (Fig. 36Q–R).

###### Immature.

Unknown.

###### Host plant notes.

Collected from glabrous and pubescent morphotypes.

###### Island.

Molokai.

###### Distribution notes.

Kamakou Preserve (this study), Kamiloloa, Kawela (Crawford 1927).

###### Biology.

Unknown, but morphology suggests it may gall stems/buds and may have made the bud gall in Fig. 51I.

###### Comments.

Two forms are recognized (Fig. 36): form molokaiensis (based on the type has longer genal processes, more slender paramere, and shorter female terminalia), and form laka (with shorter genal processes, broader paramere, and longer female terminalia). Although there are parallels in characteristics and extent of variation to those observed on Oahu for Pariaconus oahuensis, the degree of variation is not as pronounced in Pariaconus molokaiensis.

###### Type material.

Holotype, female (dry mounted, BPBM). See Table 2 for details of type and other material examined for this study.

##### Pariaconus hualani

Taxon classificationAnimaliaHemipteraTriozidae

Percy sp. n.

http://zoobank.org/330DA2DA-152B-479E-A5D2-966C0ABA3038

Figure 37

###### Adult colour.

General body colour dark red to red-brown, or yellow-brown. Females often appear to have a dark abdomen due to darkly pigmented egg load. Fore wing membrane clear.

###### Adult structure.

**Figure 37.**
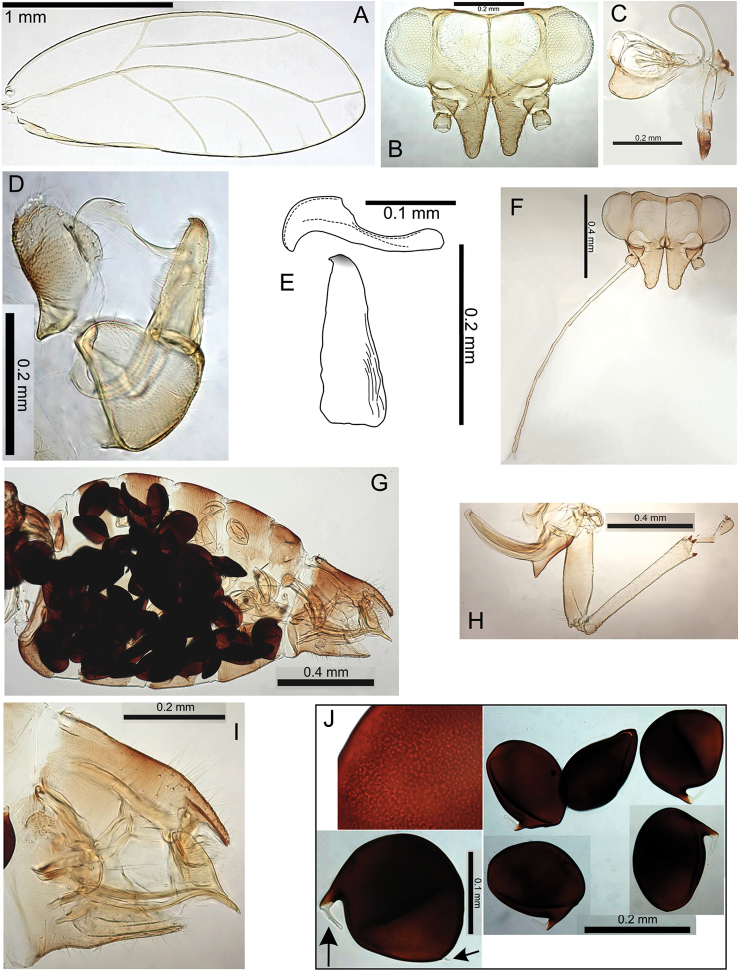
Pariaconus hualani sp. n. **A** fore wing **B** head **C** proboscis **D** male terminalia **E** aedeagus and paramere **F** head and antenna **G** female abdomen (with eggs) and terminalia **H** hind leg **I** female terminalia **J** eggs (pedicel and tail indicated, microsculpturing detailed).

Fore wing apex bluntly acute; spinules distributed in all cells; short to medium-short setae on margins and veins (Fig. 37A). Antennae medium-long (av. length 1.05; ratio AL:HW av. 1.83); genal processes long (ratio VL:GP av. 1.28), and acute or bluntly acute; medium-long setae on vertex, shorter on thorax; distal proboscis segment short (av. length 0.08); hind tibia length subequal to head width (ratio HW:HT av. 1.06) (Fig. 37B–C, F, H). Male terminalia (Fig. 37D–E): paramere longer than proctiger (ratio MP:PL av. 0.92), slender, broader at the base and tapering evenly to apex with anteriorly directed hook; distal aedeagus segment shorter than paramere (ratio PL:AEL av. 1.19) with base somewhat angular and slightly inflated, and a large hooked apex (ratio AEL:AELH av. 2.08). Female terminalia (Fig. 37G, I): proctiger short, dorsal surface convex, apex bluntly acute, anal ring short (ratio FP:RL av. 4.07); subgenital plate with slight medial bulge ventrally, bluntly acute apically; ovipositor apex lacking serrations, valvulae dorsalis moderately convex dorsally.

###### Egg.

Short, broadly ovoid to almost circular, pigmented brown (except tip of pedicel and tail), surface with microsculpturing, short pedicel with slightly inflated tip, tail extremely short (Fig. 37J).

###### Immature.

Unknown.

###### Host plant notes.

Preference unknown; collected from glabrous and pubescent morphotypes.

###### Island.

Molokai.

###### Distribution notes.

Known only from Kamakou Preserve.

###### Biology.

Unknown; but morphology suggests it may be a leaf galler.

###### Etymology.

Named after Hualani, a High Chiefess of Molokai in ancient times (noun in the nominative singular standing in apposition to the generic name).

###### Comments.

Molecular data recovers this taxon as sister to Pariaconus kupua from Maui.

###### Type material.

Holotype male (slide mounted, BMNH). See Table 2 for details of type and other material examined for this study.

##### Pariaconus mauiensis

Taxon classificationAnimaliaHemipteraTriozidae

Percy sp. n.

http://zoobank.org/6BE3239A-184E-40C7-8C12-D795ABEB8FF0

Figure 38

###### Adult colour.

General body colour yellow-brown to green. Females often appear to have a dark abdomen due to darkly pigmented egg load. Fore wing membrane clear, or slightly fuscous.

###### Adult structure.

**Figure 38.**
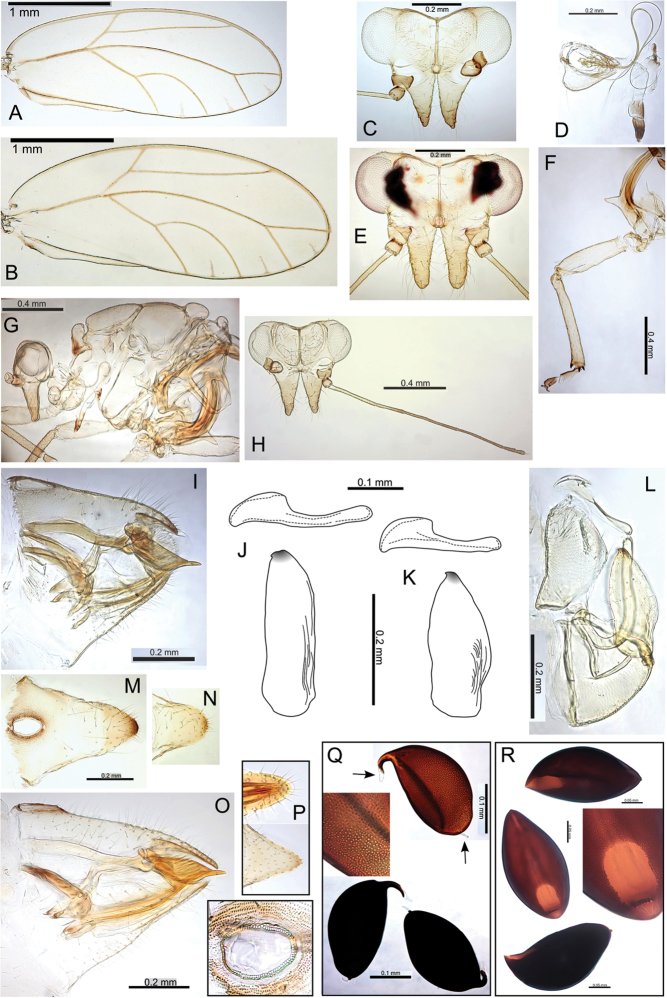
Pariaconus mauiensis sp. n. **A, B** fore wing: **A** form mauiensis **B** form kuula **C, D** form mauiensis: **C** head **D** proboscis **E** head (uncleared ocular tissue), form kuula **F** hind leg **G** head and thorax **H** head and antenna (female) **I** female terminalia, form mauiensis **J** aedeagus and paramere, form kuula **K, L, M, N** form mauiensis: **K** aedeagus and paramere **L** male terminalia **M** female proctiger (dorsal view) **N** female subgenital plate apex (ventral view) **O, P** form kuula: **O** female terminalia with detail of anal ring (inset) **P** apices of female proctiger (above) and subgenital plate (below) **Q, R** eggs (pedicel and tail indicated, microsculpturing detailed): **Q** form mauiensis **R** form kuula.

Fore wing apex rounded; spinules distributed in all cells; short setae on margins and veins (Fig. 38A–B). Antennae long (av. length 1.35; ratio AL:HW av. 2.08); genal processes extremely long, longer than or subequal to vertex length (ratio VL:GP av. 0.93), and acute; long to medium-long setae on vertex and thorax; distal proboscis segment short (av. length 0.10); hind tibia length subequal to head width (ratio HW:HT av. 1.01) (Fig. 38C–H). Male terminalia (Fig. 38I–L): paramere longer than proctiger (ratio MP:PL av. 0.9), broad at the base, and medially expanded before tapering evenly to apex with anteriorly directed hook; distal aedeagus segment shorter than paramere (ratio PL:AEL av. 1.25) with base rounded, not or slightly inflated, and a shallow hooked apex (ratio AEL:AELH av. 2.07). Female terminalia (Fig. 38M–P): proctiger short, dorsal surface more or less straight to convex apically, apex bluntly acute to acute, anal ring short (ratio FP:RL av. 4.65); subgenital plate with slight or no medial bulge ventrally, bluntly acute to acute apically; ovipositor apex lacking serrations, valvulae dorsalis not or moderately convex dorsally.

###### Egg.

Short, broad, pigmented brown to dark brown (except tip of pedicel and tail), surface with coarse microsculpturing, and either with distinct medium-long pedicel with slightly inflated tip, or pedicel obscured and an unsclerotized patch at the base of the egg, tail medium-short (Fig. 38Q–R).

###### Immature.

Unknown.

###### Host plant notes.

Collected from glabrous morphotypes.

###### Island.

Maui.

###### Distribution notes.

Known from east and west Maui; there is some geographical clustering in the molecular data but the east/west divergence of haplotypes is not as distinct as in Pariaconus montgomeri.

###### Biology.

Unknown; but morphology suggests it may gall stems/buds.

###### Etymology.

Named for the distribution on the island of Maui (noun in the genitive case).

###### Comments.

Two forms are recognized (Fig. 38): form mauiensis (based on the type is a smaller form with a shorter, broader paramere, and female terminalia shorter and more apically blunt, known from east Maui), and form kuula (is larger sized, with longer, more slender paramere, and female terminalia longer and apically acute, known from west Maui). There is some variation in the egg type as well; form kuula has an unsclerotized patch at the base of the egg (similar to that found in Pariaconus oahuensis form latus) that suggests the forms of Pariaconus mauiensis may produce different gall types as in Pariaconus oahuensis (e.g. stem galls and cone leaf galls in Pariaconus oahuensis).

###### Type material.

Holotype male (slide mounted, BMNH). See Table 2 for details of type and other material examined for this study.

##### Pariaconus kupua

Taxon classificationAnimaliaHemipteraTriozidae

Percy sp. n.

http://zoobank.org/28468533-0C52-4B0C-B844-CBF81C4B37B4

Figures 39 , 51E–F

###### Adult colour.

General body colour yellow-brown to green. Females often appear to have a dark abdomen due to darkly pigmented egg load. Fore wing membrane clear, or slightly fuscous.

###### Adult structure.

**Figure 39.**
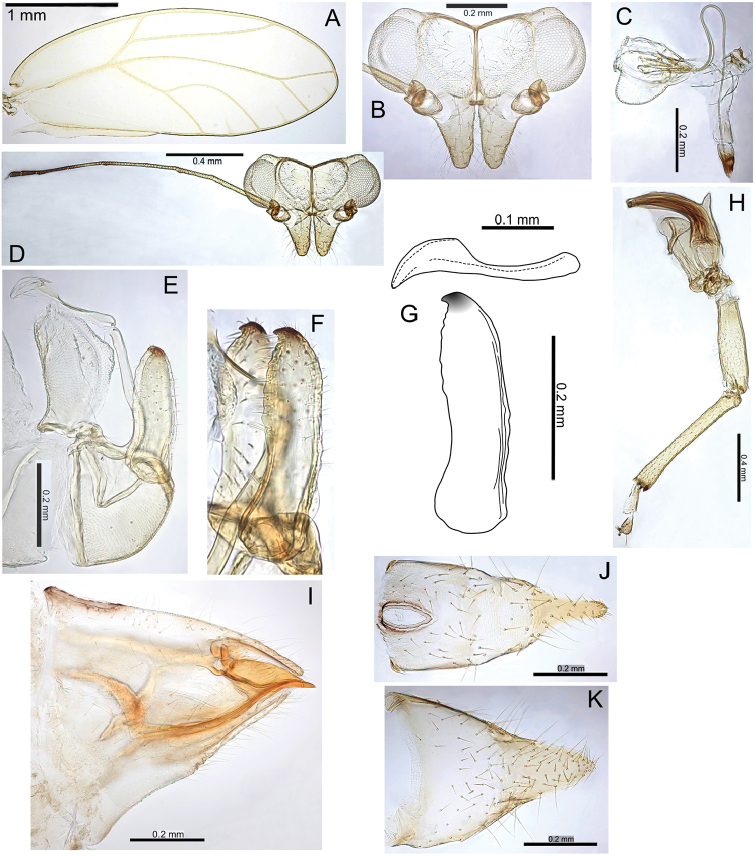
Pariaconus kupua sp. n. **A** fore wing **B** head **C** proboscis **D** head and antenna **E** male terminalia **F** parameres **G** aedeagus and paramere **H** hind leg **I** female terminalia **J** female proctiger (dorsal view) **K** female subgenital plate (ventral view).

Fore wing apex bluntly acute to rounded; spinules sparsely distributed in all cells except r_1_; short setae on margins and veins (Fig. 39A). Antennae long (av. length 1.40; ratio AL:HW av. 2.07); genal processes long, only slightly shorter than vertex (ratio VL:GP av. 1.09), and acute or bluntly acute; long setae on vertex, shorter on thorax; distal proboscis segment short (av. length 0.11); hind tibia longer or subequal to head width (ratio HW:HT av. 0.94) (Fig. 39B–D, H). Male terminalia (Fig. 39E–G): paramere longer than proctiger (ratio MP:PL av. 0.85), slender, more or less parallel-sided before constricting below apex with anteriorly directed hook; distal aedeagus segment shorter than paramere (ratio PL:AEL av. 1.21) with base rounded, not or slightly inflated, and a shallow hooked apex (ratio AEL:AELH av. 2.30). Female terminalia (Fig. 39I–J): proctiger long, dorsal surface convex, apex bluntly acute, anal ring short (ratio FP:RL av. 4.25); subgenital plate with slight medial bulge ventrally, acute apically; ovipositor apex lacking serrations, valvulae dorsalis moderately convex dorsally.

###### Egg.

Short, broadly ovoid to almost circular, pigmented brown (except tip of pedicel and tail), surface with microsculpturing, short pedicel with slightly inflated tip, tail extremely short.

###### Immature.

Colour and structure: 5^th^ instar: Cream to orange. Elongate ovoid in outline, wing buds protruding with moderate humeral lobes (similar to Pariaconus montgomeri in Fig. 51A). Tarsi with large claws. Circumanal ring small (CPW:RW 18.40), u-shaped with a mostly single row of sometimes interrupted cells (Fig. 51F). Chaetotaxy: 5^th^ instar: Head, thorax and abdomen with scattered long to medium-long simple setae (Fig. 51E).

###### Host plant notes.

Collected from glabrous morphotypes.

###### Island.

Maui.

###### Distribution notes.

Known from east and west Maui; the molecular analysis clearly distinguishes eastern from western haplotypes.

###### Biology.

Galls stems and occasionally petioles, resulting in irregular swellings.

###### Etymology.

Named after the kupua, tricksters of the island forests in Hawaiian mythology (noun in the nominative singular standing in apposition to the generic name).

###### Comments.

Molecular data recovers this taxon as sister to Pariaconus hualani from Molokai.

###### Type material.

Holotype male (slide mounted, BMNH). See Table 2 for details of type and other material examined for this study.

##### Pariaconus montgomeri

Taxon classificationAnimaliaHemipteraTriozidae

Percy sp. n.

http://zoobank.org/C2B0DCD5-BD72-4BE5-83D7-490C082E53A3

**Figure 40.**
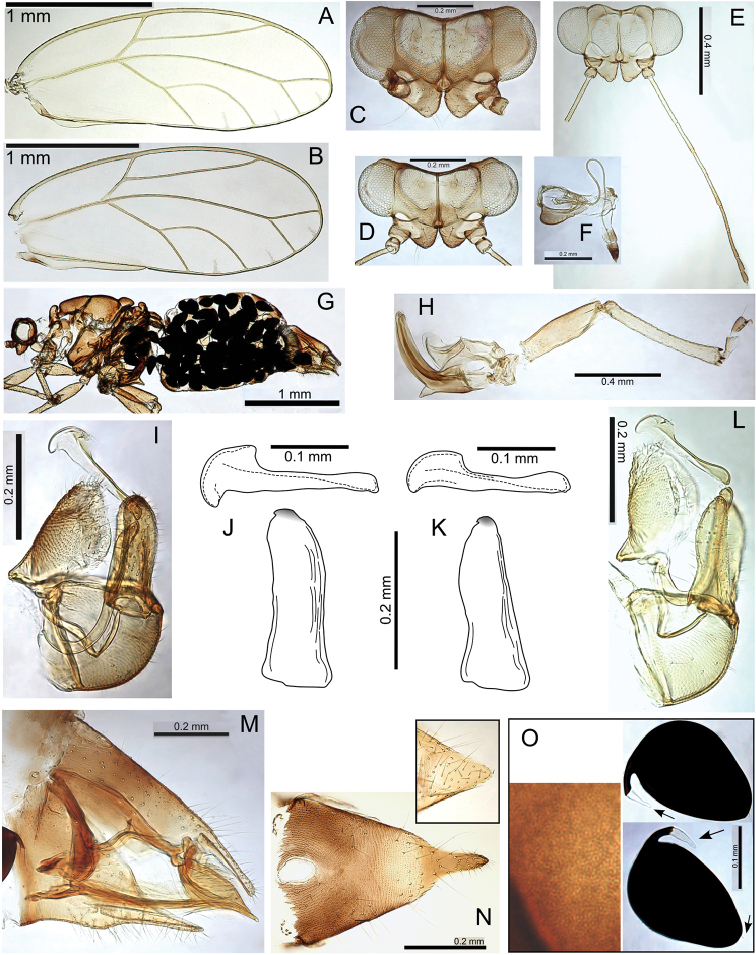
Pariaconus montgomeri sp. n. **A, B** fore wing: **A** form montgomeri **B** form paliuliensis **C, D** head: **C** form montgomeri **D** form paliuliensis **E** head and antenna **F** proboscis **G** female with abdomen full of eggs **H** hind leg **I, J** form paliuliensis: **I** male terminalia **J** aedeagus and paramere **K, L, M, N, O** form montgomeri: **K** aedeagus and paramere **L** male terminalia **M** female terminalia **N** female proctiger (dorsal view) with apex of subgenital plate (ventral view, inset) **O** eggs (pedicel and tail indicated, microsculpturing detailed).

Figures 40 , 51A–D, G–H

###### Adult colour.

Generally body colour orange-red to red-brown. Females often appear to have a dark abdomen due to darkly pigmented egg load. Fore wing membrane clear, or slightly fuscous.

###### Adult structure.

Fore wing apex bluntly acute to rounded; spinules distributed in all cells but few in r_1_; medium-short setae on margins and veins. Antennae medium-long (av. length 1.06; ratio AL:HW av. 1.89); genal processes medium-short (ratio VL:GP av. 2.38), and bluntly acute; medium-short to short setae on vertex and thorax; distal proboscis segment short (av. length 0.08); hind tibia length subequal to head width (ratio HW:HT av. 1.03). Male terminalia: paramere length subequal to or longer than proctiger (ratio MP:PL av. 0.95), slender, broader at the base and more or less parallel-sided or slightly medially expanded before constricting below apex with a small anteriorly directed hook; distal aedeagus segment shorter or subequal to paramere (ratio PL:AEL av. 1.05) with base rounded, not or slightly inflated, and a short, compact, shallow hooked apex (Fig. 40J–K) (ratio AEL:AELH av. 2.67). Female terminalia: proctiger long, dorsal surface slightly to moderately convex, apex acute, anal ring short (ratio FP:RL av. 5.24); subgenital plate with slight to moderate medial bulge ventrally, acute apically; ovipositor apex lacking serrations, valvulae dorsalis moderately convex dorsally.

###### Egg.

Short, broad, pigmented brown to dark brown (except tip of pedicel and tail) with fine surface microsculpturing, medium-long pedicel with slightly inflated tip, tail extremely short or absent (Fig. 40O).

###### Immature.

Colour and structure: 5^th^ instar: Cream to orange. Elongate ovoid in outline, wing buds protruding with moderate humeral lobes (Fig. 51A–B). Tarsi with large claws (Fig. 51C). Circumanal ring large, wide (CPW:RW av. 6.30), and composed of two lateral u-shaped multicellular sections with irregular borders, sometimes reduced or interrupted (Fig. 51D). Chaetotaxy: 5^th^ instar: Head, thorax and abdomen with scattered long to medium-long simple setae.

###### Host plant notes.

Collected from glabrous morphotypes.

###### Island.

Maui.

###### Distribution notes.

Known from east and west Maui; molecular data indicates very distinct eastern and western populations that are characterized by the two recognized forms.

###### Biology.

Makes flat leaf galls (Fig. 51G–H), often with a small central depression (see also Pariaconus ohiacola).

###### Etymology.

Named after Steve Montgomery, an extraordinary field biologist who made a substantial contribution to this study (noun in the genitive case).

###### Comments.

Two forms are recognized (Fig. 40): form montgomeri (based on the type is a slightly larger form, with more sinuous paramere, less developed aedeagus hook, and longer female terminalia, known from west Maui), and form paliuliensis (generally smaller with paramere more straight, aedeagus hook more developed, and shorter female terminalia, known from east Maui).

###### Type material.

Holotype male (slide mounted, BMNH). See Table 2 for details of type and other material examined for this study.

##### Pariaconus hawaiiensis

Taxon classificationAnimaliaHemipteraTriozidae

(Crawford, 1918) comb. n.

**Figure 41.**
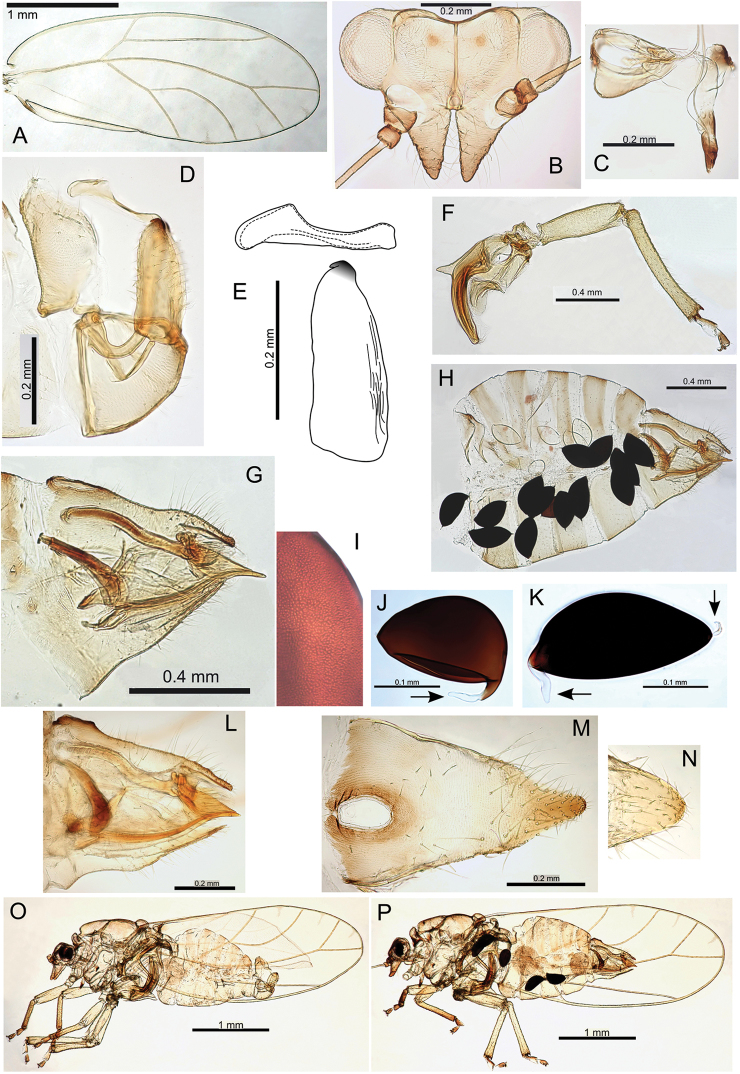
Pariaconus hawaiiensis. **A** fore wing **B** head **C** proboscis **D** male terminalia **E** aedeagus and paramere **F** hind leg **G** female terminalia (Kipuka Alani, Hawaii) **H** female abdomen (with eggs) **I, J, K** eggs (pedicel and tail indicated, microsculpturing detailed) **L** female terminalia (Kona Hema, Hawaii) **M** female proctiger (dorsal view) **N** apex of female subgenital plate (ventral view) **O** male **P** female.

Figures 41 , 52A–C, J, O–R

Trioza hawaiiensis Crawford, 1918: 444

###### Adult colour.

General body colour green or yellow-green, but often with brown on legs, thorax and abdomen. Females often appear to have a dark abdomen due to darkly pigmented egg load. Fore wing membrane clear.

###### Adult structure.

Fore wing apex rounded; spinules with limited distributed in all cells; medium-short to short setae on margins and veins (Fig. 41A). Antennae long (av. length 1.36; ratio AL:HW av. 2.06); genal processes long, subequal or shorter than vertex length (ratio VL:GP av. 1.19), and acute; long to medium-long setae on vertex and thorax; distal proboscis segment short (av. length 0.11); hind tibia longer or subequal to head width (ratio HW:HT av. 0.95) (Fig. 41B–C, F, O–P). Male terminalia (Fig. 41D–E): paramere length subequal to proctiger (ratio MP:PL av. 0.99), broad and more or less parallel-sided basally before tapering evenly to apex with anteriorly directed hook; distal aedeagus segment shorter than paramere (ratio PL:AEL av. 1.18) with base rounded, slightly inflated, and a large shallow hooked apex (ratio AEL:AELH av. 2.17). Female terminalia (Fig. 41H–G, L–N): proctiger short to moderately long, dorsal surface more or less straight, or slightly depressed medially, convex apically, apex bluntly acute, anal ring short (ratio FP:RL av. 4.59); subgenital plate with slight medial bulge ventrally, bluntly acute apically; ovipositor apex lacking serrations, valvulae dorsalis not convex dorsally.

###### Egg.

Short, broad, pigmented brown to dark brown (except tip of pedicel and tail), surface with microsculpturing, medium-long pedicel with slightly inflated tip, tail short (Fig. 41I–K).

###### Immature.

Colour and structure: 5^th^ instar: Cream to orange. Elongate ovoid in outline, wing buds protruding with moderate humeral lobes (Fig. 52A–B). Tarsi with large claws. Circumanal ring small (CPW:RW av. 20.39), u-shaped with a mostly single row of often interrupted cells (Fig. 52C). Chaetotaxy: 5^th^ instar: Head, thorax and abdomen with scattered long to medium-long simple setae (Fig. 52J). 1^st^ instar: Unknown.

###### Host plant notes.

Known from both glabrous and pubescent morphotypes, but mostly associated with pubescent and semi-pubescent types.

###### Island.

Hawaii.

###### Distribution notes.

Widely distributed on the island of Hawaii; there are some distinct clusters in the molecular analysis, with the southern and western populations sister to and nested within members from Kohala (north), and these are distinct from two individuals from the central Saddle Rd area.

###### Biology.

Galls stems and buds, resulting in irregular swellings, and in the case of buds, inhibits bud growth (Fig. 52O–R).

###### Comments.

Individuals from Kona Hema have a notably broader paramere.

###### Type material.

Holotype, male (?) (dry mounted, damaged, abdomen missing, BPBM). See Table 2 for details of type and other material examined for this study.

##### Pariaconus pele

Taxon classificationAnimaliaHemipteraTriozidae

Percy sp. n.

http://zoobank.org/BF151258-53F1-47D2-804A-17F821343BC3

**Figure 42.**
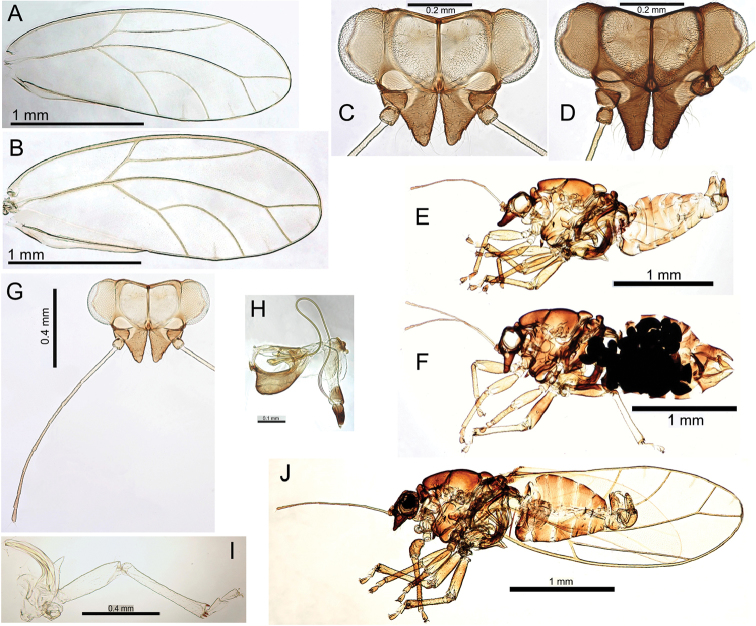
Pariaconus pele sp. n. **A–B** fore wing: **A** form pele **B** form kohalensis **C–D** head: **C** form pele **D** form kohalensis **E–F** form kohalensis: **E** male **F** female with abdomen full of eggs **G** head and antenna **H** proboscis **I** hind leg **J** male, form pele.

**Figure 43.**
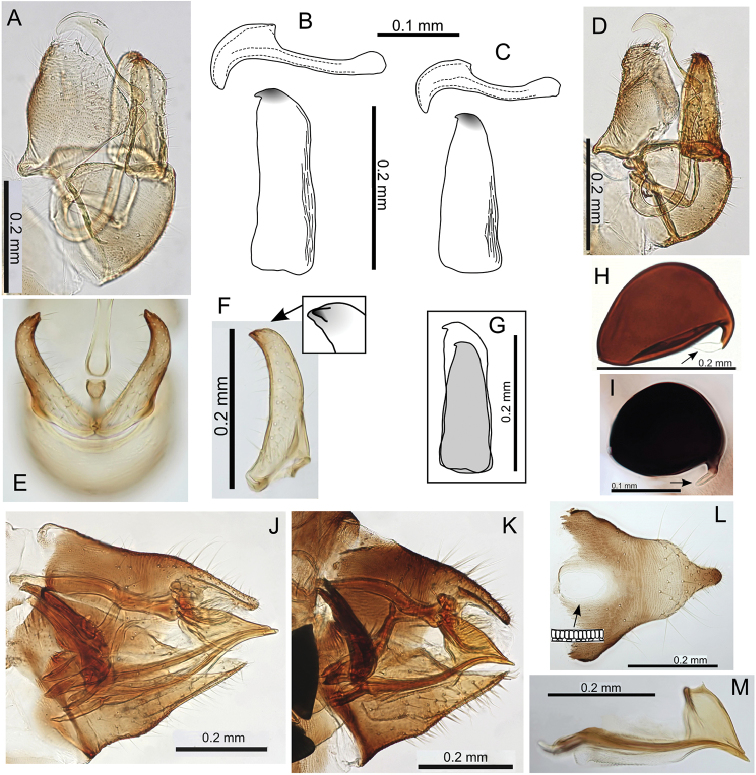
Pariaconus pele sp. n. **A–B** form pele: **A** male terminalia **B** aedeagus and paramere **C, D–F** form kohalensis: **C** aedeagus and paramere **D** male terminalia **E** male terminalia (posterior view) **F** paramere (posterior view) with apex detail (inset) **G** paramere shape variation comparison **H–I** egg (pedicel indicated): **H** form pele **I** form kohalensis **J** female terminalia, form pele **K–M** form kohalensis: **K** female terminalia **L** female proctiger (dorsal view) with anal ring cells detailed **M** ovipositor.

Figures 42 , 43 , 52K–N, X–BB

###### Adult colour.

General body colour dark brown to red-brown, the head and genal processes are often darker or black. Females often appear to have a dark abdomen due to darkly pigmented egg load. Fore wing membrane clear, or slightly fuscous.

###### Adult structure.

Fore wing apex rounded; spinules distributed in all cells, except few or none in r_1_; medium-short setae on margins and veins (Fig. 42A–B). Antennae medium-long (av. length 0.99; ratio AL:HW av. 1.79); genal processes variable medium-long (common form) to long (Kohala form) (ratio VL:GP av. 1.52), bluntly acute to acute; medium-short to short setae on vertex and thorax; distal proboscis segment short (av. length 0.09); hind tibia length subequal to head width (ratio HW:HT av. 1.07) (Fig. 42C–J). Male terminalia (Fig. 43A–G): paramere length subequal to proctiger (ratio MP:PL av. 1.01), broader at the base and tapering gradually to apex with anteriorly directed hook; distal aedeagus segment length subequal to paramere (ratio PL:AEL av. 1.07) with base rounded, slightly inflated, and a large hooked apex (ratio AEL:AELH av. 2.30). Female terminalia (Fig. 43J–M): proctiger medium-long (common form, f. pele) to short (Kohala form, f. kohalensis), dorsal surface slightly to moderately convex, with or without medial depression, apex bluntly acute, anal ring medium-short (ratio FP:RL av. 4.23); subgenital plate with slight to moderate medial bulge ventrally, acute apically; ovipositor apex lacking serrations, valvulae dorsalis moderately convex dorsally.

###### Egg.

Short, broad (almost circular in Kohala form), pigmented brown to dark brown (except tip of pedicel and tail) with surface microsculpturing, medium-long pedicel with slightly inflated tip, tail extremely short or absent (Fig. 43H–I).

###### Immature.

Colour and structure: 5^th^ instar: Cream to orange. Elongate ovoid in outline, wing buds protruding with moderate humeral lobes (Fig. 52K). Tarsi with large claws. Circumanal ring small (CPW:RW av. 23.67), u-shaped with a mostly single row of sometimes interrupted cells (Fig. 52K–L). 1^st^ instar dorsal surface scaly. Chaetotaxy: 5^th^ instar: Head, thorax and abdomen with scattered long to medium-long simple setae, sometimes with clusters of stouter simple setae on the abdomen (Fig. 52K, M). 1^st^ instar (Fig. 52N): Setal arrangement similar to Pariaconus ohiacola but with marginal sectasetae narrow and blunt as in Pariaconus oahuensis (anterior of head with 4-5 pairs, a single pair post ocular, a single pair of sectasetae on the apices of each wing bud, and the abdomen with 7-8 pairs).

###### Host plant notes.

Known from both glabrous and pubescent morphotypes, but mostly associated with glabrous and semi-pubescent forms.

###### Island.

Hawaii.

###### Distribution notes.

The most common species on Hawaii. In addition to a distinct form “Kohala” only known from the Kohala region, within the “common” form there are two groups both of which are widespread: group 1 includes distinct populations from Kona Hema (south west), Kohala (north east), Saddle Rd (central), and Hualalai (north west); group 2 is more mixed with individuals from south, central, east and west, but not from Kohala.

###### Biology.

Makes flat galls on leaves (Fig. 52X–BB), galls often have a small depression or raised plug in the centre (Fig. 52Z–AA). Galls open typically on the lower leaf surface either by irregular cracking, or more rarely by a circular suture around the margin of the gall (Fig. 52Y–BB). This species often co-occurs with Pariaconus hawaiiensis (e.g. Fig. 4I) and/or Pariaconus pyramidalis (e.g. Fig. 52X–Y).

###### Etymology.

Named after Pele, the volcano and fire goddess in Hawaiian mythology (noun in the nominative singular standing in apposition to the generic name).

###### Comments.

Two forms are recognized (Figs 42–43): form pele (based on the type, is the most common form, generally smaller with slightly shorter genae, longer paramere and longer female terminalia, the egg is more ovoid), and form kohalensis (slightly longer genae, shorter paramere and shorter female terminalia, the egg is almost round and the pedicel shorter). Comparison of paramere shape and size is illustrated in Fig. 43G.

###### Type material.

Holotype male (slide mounted, BMNH). See Table 2 for details of type and other material examined for this study.

##### Pariaconus pyramidalis

Taxon classificationAnimaliaHemipteraTriozidae

Percy sp. n.

http://zoobank.org/88EEF065-97CA-428C-9A9F-520C91BA885A

**Figure 44.**
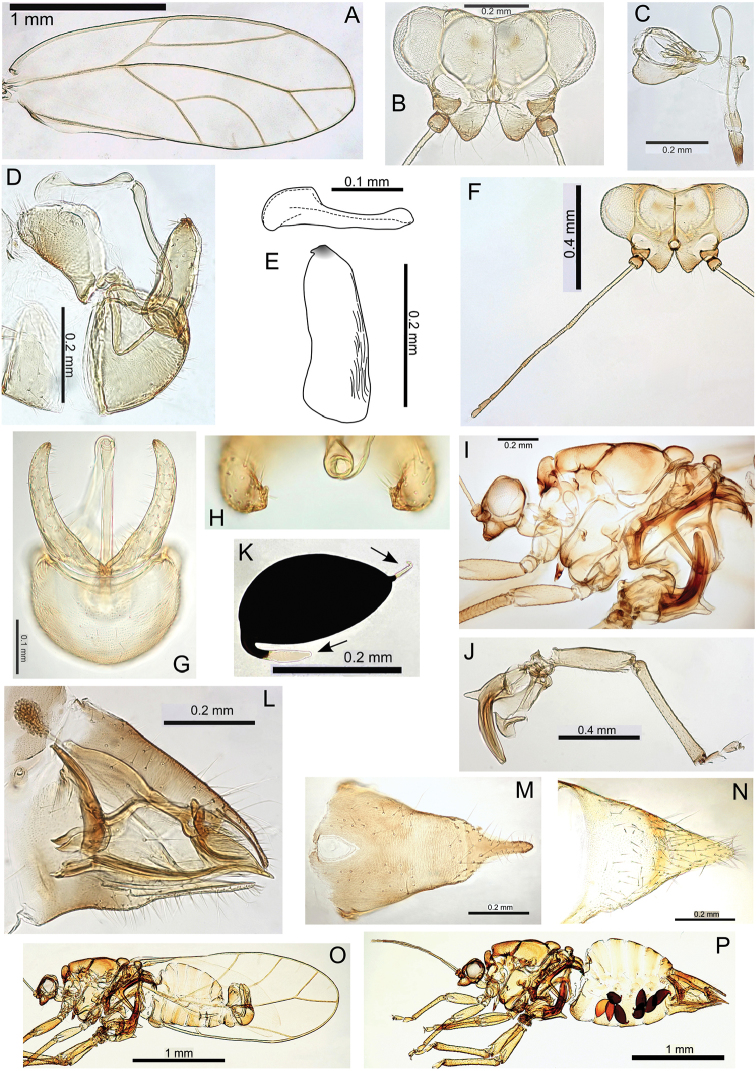
Pariaconus pyramidalis sp. n. **A** fore wing **B** head **C** proboscis **D** male terminalia **E** aedeagus and paramere **F** head and antenna **G** male terminalia (posterior view) **H** paramere apices (dorsal view) **I** head and thorax **J** hind leg **K** egg (pedicel and tail indicated) **L** female terminalia **M** female proctiger (dorsal view) **N** female subgenital plate (ventral view) **O** male **P** female.

Figures 44 , 52D–I, S–Y

###### Adult colour.

General body colour brown or yellow-brown, or yellow-green, the genal processes are often paler than the head. Females often appear to have a dark abdomen due to darkly pigmented egg load. Fore wing membrane clear, or slightly fuscous.

###### Adult structure.

Fore wing apex rounded; spinules distributed in all cells; short setae on margins and veins (Fig. 44A). Antennae medium-long (av. length 1.06; ratio AL:HW av. 1.75); genal processes medium-short (ratio VL:GP av. 1.94), bluntly acute to acute; medium-short to short setae on vertex and thorax; distal proboscis segment short (av. length 0.09); hind tibia length subequal to head width (ratio HW:HT av. 0.97) (Fig. 44B–C, F, H). Male terminalia (Fig. 44D–E): paramere longer than proctiger (ratio MP:PL av. 0.84), broad and more or less parallel-sided basally before tapering to apex with anteriorly directed hook; distal aedeagus segment shorter than paramere (ratio PL:AEL av. 1.17) with base rounded, slightly inflated, and a shallow hooked apex (ratio AEL:AELH av. 2.21). Female terminalia (Fig. 44G): proctiger long, dorsal surface slightly to moderately convex, apex acute, anal ring medium-short (ratio FP:RL av. 6.23); subgenital plate with slight to moderate medial bulge ventrally, acute apically; ovipositor apex lacking serrations, valvulae dorsalis slightly convex dorsally.

###### Egg.

Short, broad, pigmented brown to dark brown (except tip of pedicel and tail) with surface microsculpturing, long pedicel with inflated tip, tail long (Fig. 44I).

###### Immature.

Colour and structure: 5^th^ instar: Cream to orange. Elongate ovoid in outline, wing buds protruding with moderate humeral lobes (similar to Pariaconus hawaiiensis in Fig. 52A). Tarsi with large claws. Circumanal ring small (CPW:RW av. 16.96), u-shaped with patches of single or multiple rows of interrupted cells (Fig. 52D–E), sometimes reduced or absent. Chaetotaxy: 5^th^ instar: Head, thorax and abdomen with scattered long to medium-long simple setae (Fig. 52F, I). 1^st^ instar (Fig. 52H): Setal arrangement similar to Pariaconus oahuensis, with simple setae on anterior margin of head and otherwise narrow, blunt sectasetae (a single pair post ocular, a single pair on the apices of each wing bud, and 7-8 pairs on the abdomen); by the 2^nd^ instar all setae are simple (as is typical of ohialoha group) (Fig. 52G).

###### Host plant notes.

Known from both glabrous and pubescent morphotypes.

###### Island.

Hawaii.

###### Distribution notes.

This species is widely distributed on Hawaii. The molecular data suggests an initial diversification in the south western part of the island and subsequent spread north, west, and east.

###### Biology.

Although predominantly makes cone galls on leaves (Fig. 52S–V), two immatures were dissected from stem galls and one from a flower bud gall that DNA barcoded to this species, suggesting that some lability in galling exists (see Discussion). The cone gall extends from the lower surface of the leaf and the galls typically open on the upper leaf surface by a suture around the margin of the gall resembling a hinged trap door (Fig. 52W, X). The shape of the cone gall can vary depending on the host morphotype, galls on pubescent types tend to be broad and short (Fig. 52U–V), while those on glabrous types tend to be more narrow and elongate (Fig. 52S–T), in both cases gall tissue often becomes reddish in colour. Galls can be scattered across the leaf surface (Fig. 52X–Y) or clustered (Fig. 52V), and in some cases are found clustered linearly along the leaf midrib (Fig. 52S).

###### Etymology.

Named for the shape of the pyramid-like cone gall produced on leaves (adjective in the nominative singular).

###### Comments.

This species is sister to Pariaconus hawaiiensis; the switch from stem/bud galling to cone leaf galling happened in situ on Hawaii, and reflects a parallel process found on Oahu in a localized population of Pariaconus oahuensis, which although normally a stem/bud galler, produces a cone leaf gall in a localized population in the Koolau Mnts. Some lability apparently still exists on Hawaii because a localized population of Pariaconus pyramidalis was found with two individuals dissected from stem galls.

###### Type material.

Holotype male (slide mounted, BMNH). See Table 2 for details of type and other material examined for this study.

**Figure 45.**
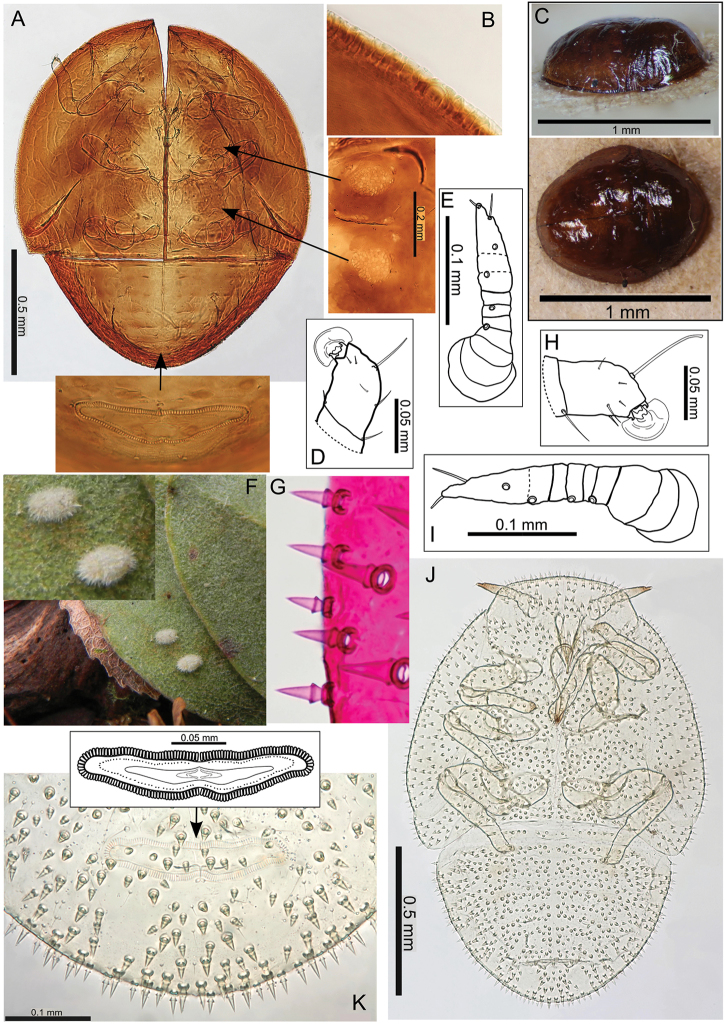
Pariaconus nigricapitus and Pariaconus proboscideus immatures. **A–E** Pariaconus nigricapitus: **A** 5^th^ instar with anal ring detail (inset below) and enlarged cells marking ventral tissue mounds (inset right) **B** detail of fused marginal setae **C** dry mounted specimen showing domed structure (lateral view above, dorsal view below) **D** tarsi **E** antenna **F–K** Pariaconus proboscideus: **F** white, hedgehog-like appearance of live specimens **G** detail of dorsal sectasetae (stained) **H** tarsi **I** antenna **J** 5^th^ instar **K** 5^th^ instar abdomen with anal ring detail (inset).

**Figure 46.**
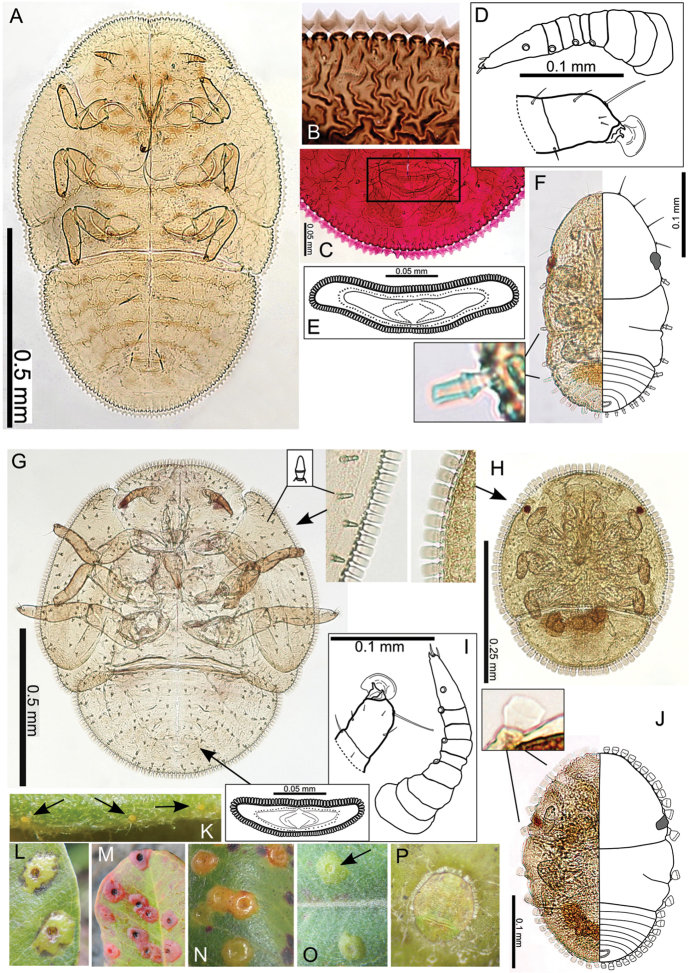
Pariaconus gracilis and Pariaconus minutus immatures. **A–F** Pariaconus gracilis: **A** 4^th^ instar **B** detail of marginal setae (5^th^ instar) **C** 4^th^ instar abdomen indicating position of anal ring (stained) **D** antenna and tarsi **E** anal ring (5^th^ instar) **F** 1^st^ instar with detail of marginal sectasetae (inset) **G–P** Pariaconus minutus: **G** 5^th^ instar with anal ring detail (inset below) and marginal and dorsal sectasetae detail (inset right) (form minutus) **H** 2^nd^ instar with marginal sectasetae detail (inset) **I** tarsi and antenna **J** 1^st^ instar with detail of fan-shaped sectasetae (inset) **K** eggs scattered on upper leaf margin **L, M, N** raised gall tissue around empty pits on upper leaf surface: **L** pale coloured **M** red coloured **N** orange coloured **O** immatures inside pits on upper leaf surface **P** detail of immature seated in pit.

**Figure 47.**
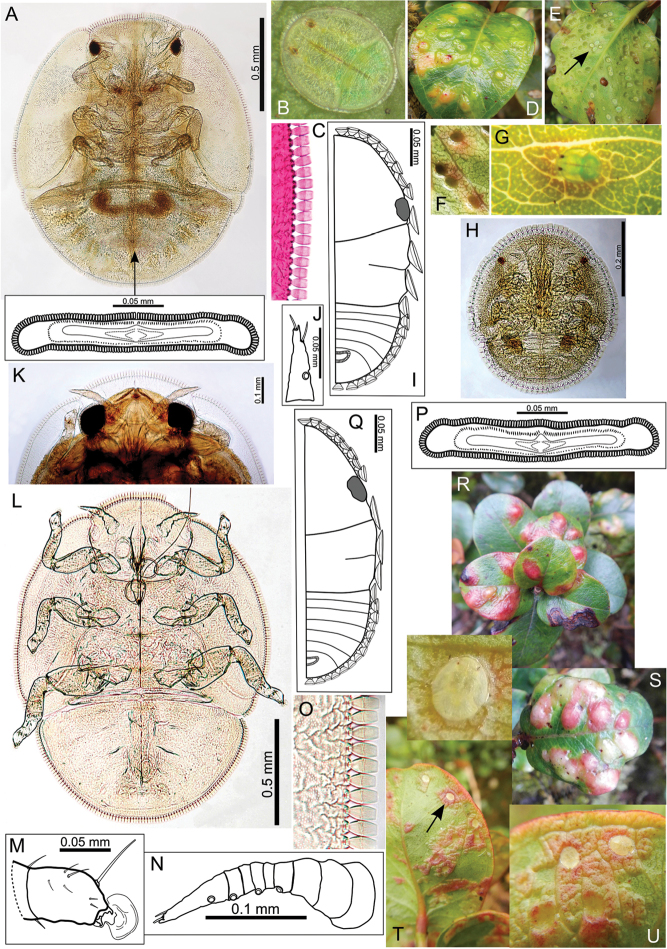
Pariaconus dorsostriatus and Pariaconus namaka immatures. **A–J** Pariaconus dorsostriatus: **A** 5^th^ instar with anal ring detail (inset) **B** detail of immature seated in pit **C** detail of marginal sectasetae (5^th^ instar, stained) **D** raised impressions on upper leaf surface from pit galls on lower leaf surface **E** immatures inside pits on lower leaf surface **F** empty pits on lower leaf surface **G** immature on lower leaf surface **H** 2^nd^ instar **I** 1^st^ instar **J** terminal antennal segment **K–U** Pariaconus namaka: **K** 5^th^ instar (uncleared) showing overhang of sclerotized dorsal surface beyond ventral body **L** 5^th^ instar **M** tarsi **N** antenna **O** detail of marginal sectasetae **P** anal ring (5^th^ instar) **Q** 1^st^ instar **R, S** raised impressions on upper leaf surface from pit galls on lower leaf surface **T** immatures inside pits on lower leaf surface with detail of immature (inset) **U** detail of immatures seated in pits with discoloured raised gall tissue around pits.

**Figure 48.**
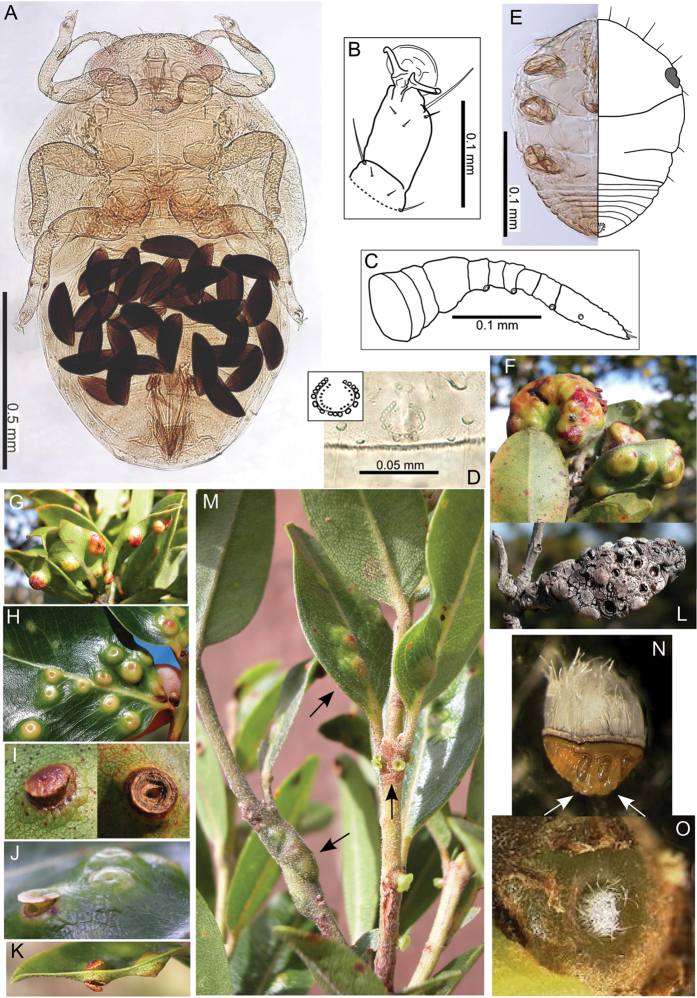
Pariaconus hiiaka and Pariaconus sp. immatures. **A–L** Pariaconus hiiaka: **A** 5^th^ instar (with eggs) **B** tarsi **C** antenna **D** anal ring with detail (inset) **E** 1^st^ instar **F** enclosed leaf galls causing leaf deformation **G, H** variation in shape and colouration of leaf galls **I, J, K** examples of gall opening by hinged circular door **L** severely galled leaf, necrotic and lignified in situ on stem **M** example of three gall types (indicated) on a single branch made by at least two Pariaconus species **N–O** Pariaconus sp. (images by Russell Messing): **N** undescribed species with unusual dorsal waxy filaments and similar ventral tissue mounds (indicated) to other cup/pit gallers **O** thick walled cup gall of undescribed species.

**Figure 49.**
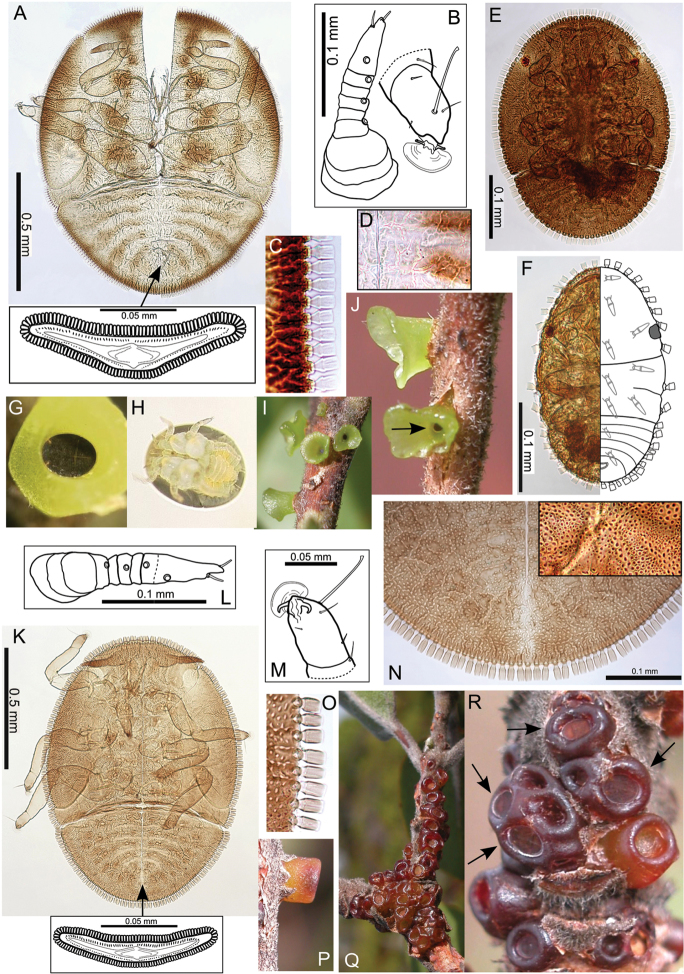
Pariaconus caulicalix and Pariaconus crassiorcalix immatures. **A–J** Pariaconus caulicalix: **A** 5^th^ instar with anal ring detail (inset) **B** antenna and tarsi **C** detail of marginal sectasetae **D** ridged dorsal surface **E** 2^nd^ instar **F** 1^st^ instar (with acute sectasetae on dorsum) **G, H** (images by Russell Messing): **G** immature seated at base of cup gall **H** soft ventral body beneath dark sclerotized dorsal surface **I, J** cup gall tissue extrudes from the plant stem with dark immatures seated in the base (indicated) **K-R** Pariaconus crassiorcalix: **K** 5^th^ instar with anal ring detail (inset) **L** antenna **M** tarsi **N** abdomen with detail of dorsal surface with round tubercle like scales **O** detail of marginal sectasetae **P, Q, R** cup gall tissue extrudes from the plant stem with orange-brown immatures seated in the base (indicated).

**Figure 50.**
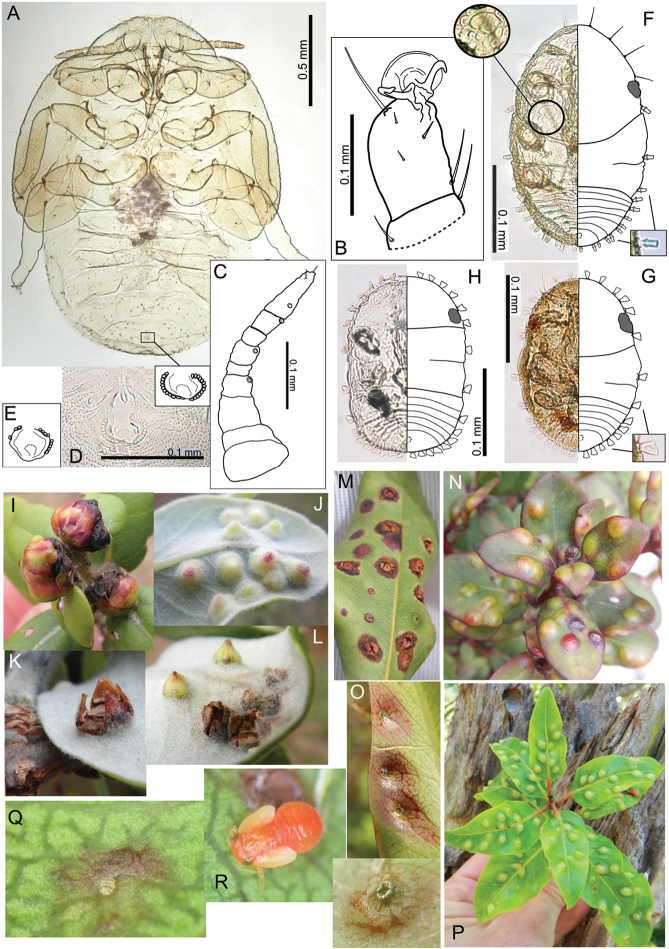
Pariaconus oahuensis and Pariaconus ohiacola immatures. **A–D** Pariaconus oahuensis: **A** 5^th^ instar with anal ring detail (inset) **B** tarsi **C** antenna **D** anal ring **E** Pariaconus ohiacola anal ring **F–H** 1^st^ instar: **F** Pariaconus oahuensis with dorsal tubercles detail (inset left) and marginal sectasetae detail (inset right) **G** Pariaconus ohiacola form ohiacola with marginal fan-shaped setae detail (inset) **H** Pariaconus ohiacola form angustipterus **I–L** Pariaconus oahuensis galls: **I** bud gall **J, K, L** cone galls on leaves opening with valves on lower leaf surface **M–R** Pariaconus ohiacola: **M** flat leaf galls open by valves on lower leaf surface **N** variation in discolouration of gall tissue **O** circular sutures in gall centres from 1^st^ instar pits on lower leaf surface with detail (inset) **P** distribution of flat leaf galls **Q** 1^st^ instar seated in pit before leaf tissue enclosure makes enclosed gall **R** 5^th^ instar dissected from closed flat leaf gall.

**Figure 51.**
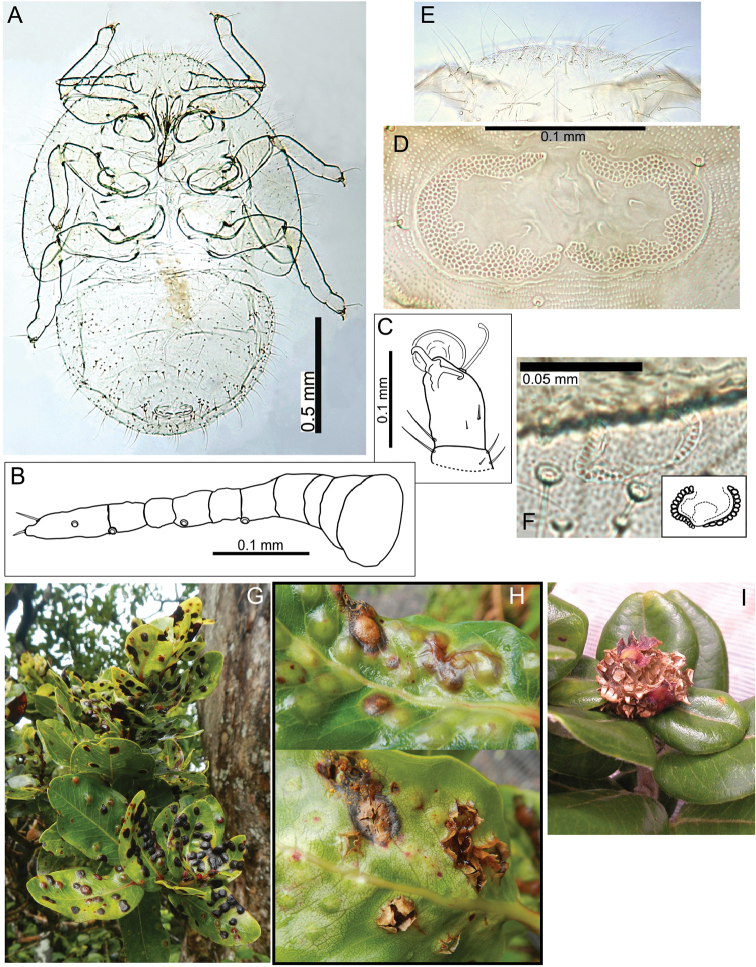
Pariaconus montgomeri and Pariaconus kupua immatures. **A–D** Pariaconus montgomeri: **A** 5^th^ instar **B** antenna **C** tarsi **D** anal ring **E–F** Pariaconus kupua: **E** detail of marginal simple setae **F** anal ring with detail (inset) **G–H** Pariaconus montgomeri: **G** distribution of closed flat leaf galls **H** impression and discolouration on upper leaf surface (above) galls open by valves on lower leaf surface (below) **I** bud gall from Molokai, may be made by Pariaconus molokaiensis.

**Figure 52.**
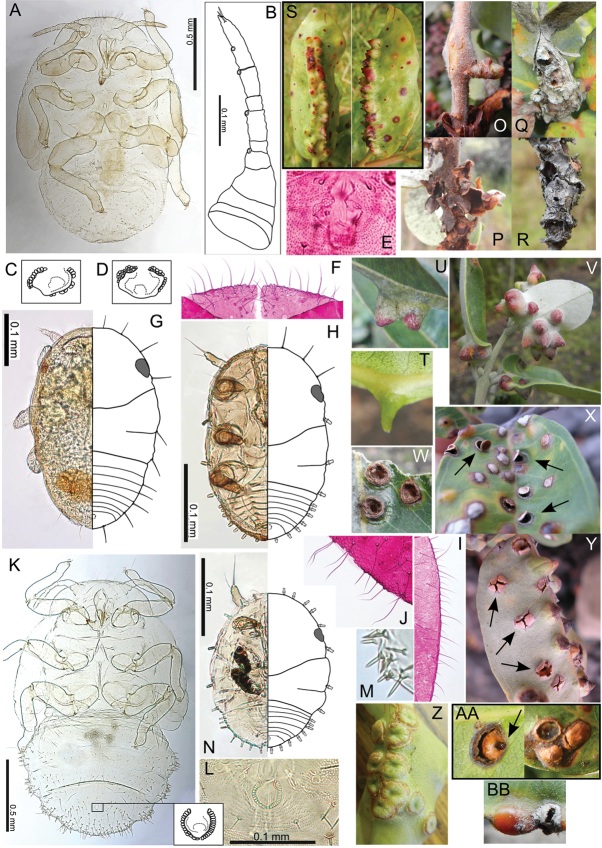
Pariaconus hawaiiensis, Pariaconus pele and Pariaconus pyramidalis immatures. **A–C** Pariaconus hawaiiensis: **A** 5^th^ instar **B** antenna **C** anal ring **D–I** Pariaconus pyramidalis: **D, E** anal ring (E stained) **F** detail of marginal simple setae (head, stained) **G** 2^nd^ instar **H** 1^st^ instar **I** detail of marginal simple setae (wing pad, stained) **J** Pariaconus hawaiiensis, detail of marginal simple setae (wing pad, stained) **K–N** Pariaconus pele: **K** 5^th^ instar with anal ring detail (inset) **L** anal ring **M** detail of marginal simple setae **N** 1^st^ instar **O–R** Pariaconus hawaiiensis: examples of developing and older necrotic and lignified stem and bud galls **S–W** Pariaconus pyramidalis: cone galls on glabrous host morphotypes clustered along the leaf mid-vein **T** detail of narrow cone gall produced on glabrous host morphotype **U, V** broad cone galls produced on more pubescent host morphotypes **W** scars remaining on upper leaf surface from old cone galls **X–Y** single leaf with both Pariaconus pele and Pariaconus pyramidalis galls: **X** upper leaf surface with cone galls opening by hinged circular door (indicated) **Y** lower leaf surface with flat galls opening by valves (indicated) **Z** donut-type gall with central depression produced by Pariaconus pele form kohalensis **AA–BB** Pariaconus pele gall variations: **AA** gall with central plug (indicated), opening with circular suture **BB** gall produced on the leaf margin.

**Figure 53.**
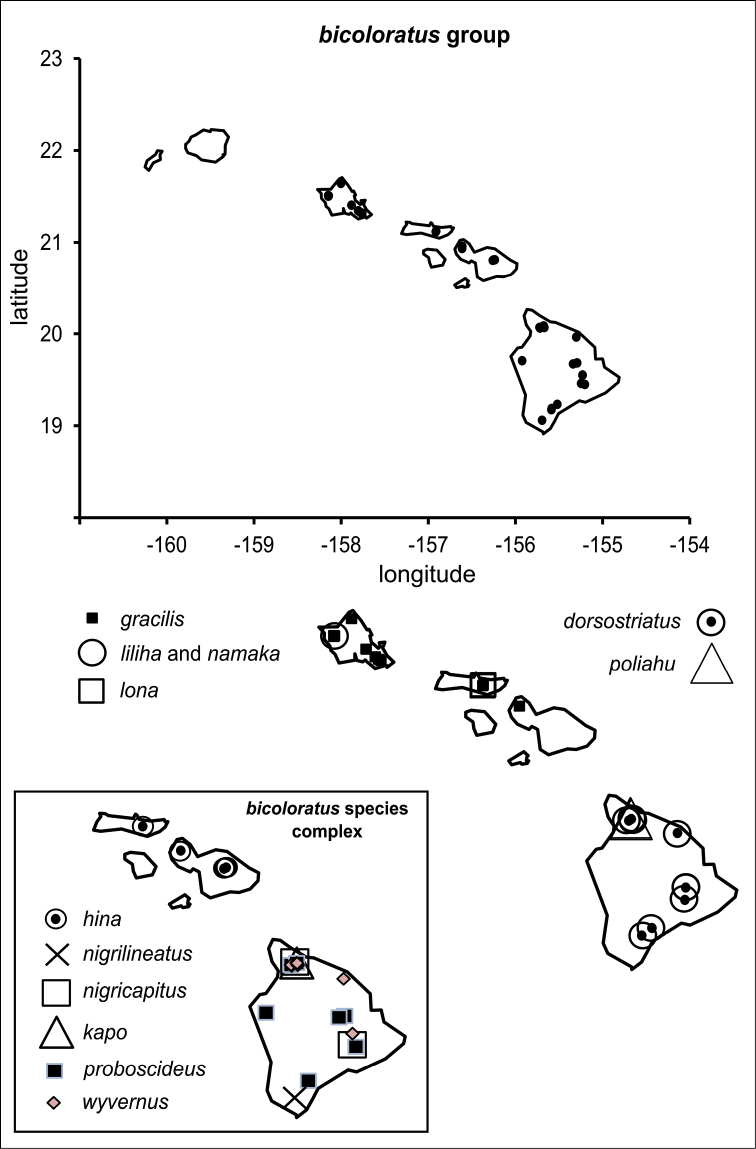
Map indicating sampling sites for bicoloratus group.

**Figure 54.**
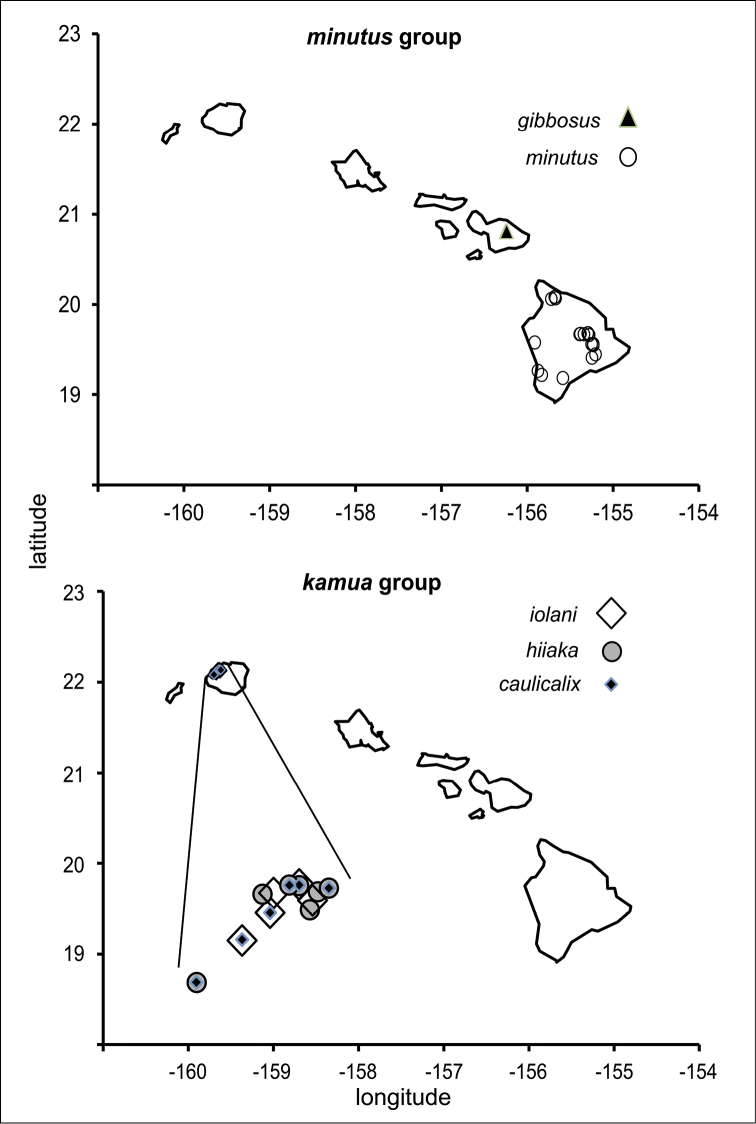
Map indicating sampling sites for minutus and kamua groups (kamua group taxa not mapped here were all found within the same limited geographic range).

**Figure 55.**
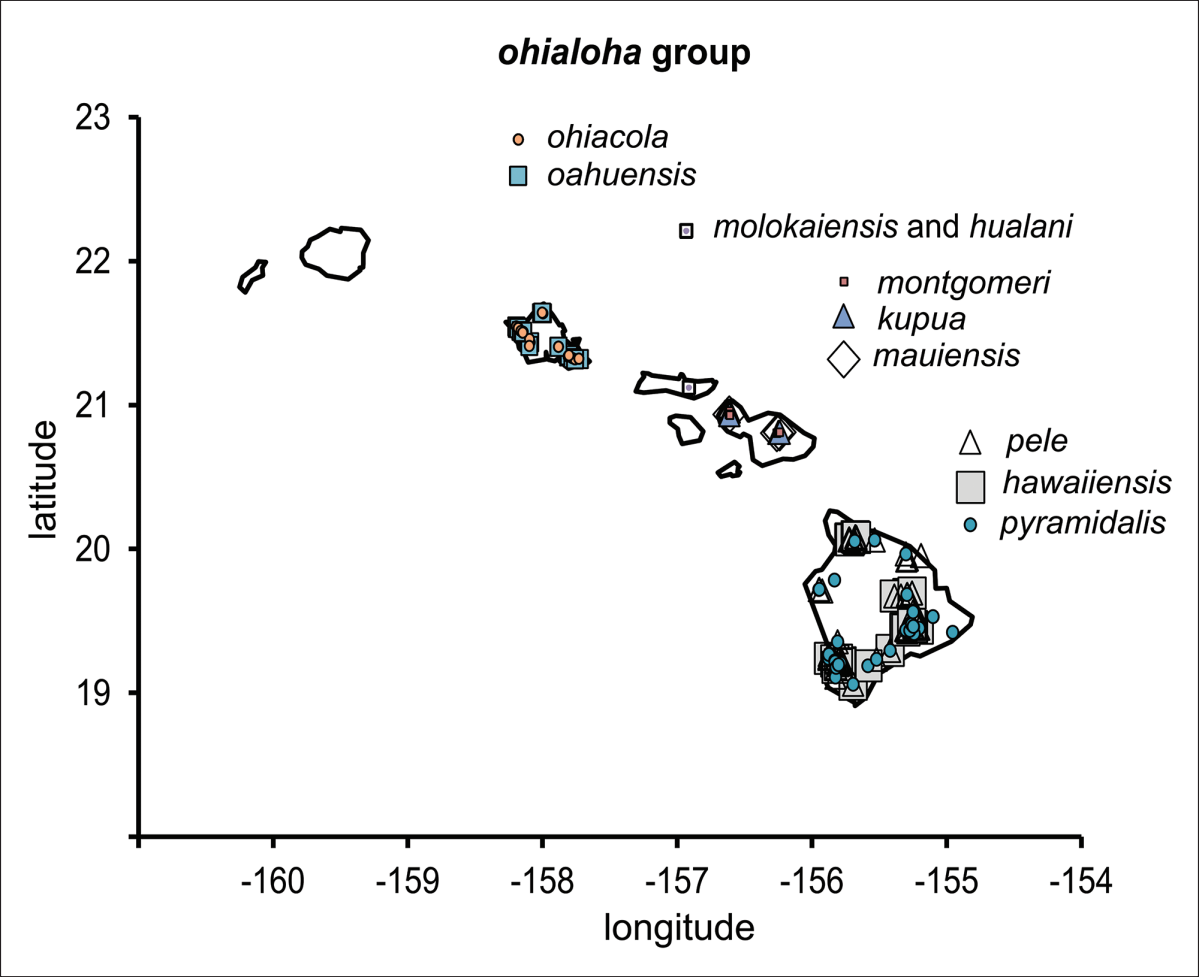
Map indicating sampling sites for ohialoha group.

## Supplementary Material

XML Treatment for Pariaconus

XML Treatment for Pariaconus nigricapitus

XML Treatment for Pariaconus hina

XML Treatment for Pariaconus wyvernus

XML Treatment for Pariaconus nigrilineatus

XML Treatment for Pariaconus kapo

XML Treatment for Pariaconus proboscideus

XML Treatment for Pariaconus poliahu

XML Treatment for Pariaconus lona

XML Treatment for Pariaconus liliha

XML Treatment for Pariaconus gracilis

XML Treatment for Pariaconus dorsostriatus

XML Treatment for Pariaconus namaka

XML Treatment for Pariaconus minutus

XML Treatment for Pariaconus gibbosus

XML Treatment for Pariaconus iolani

XML Treatment for Pariaconus hiiaka

XML Treatment for Pariaconus melanoneurus

XML Treatment for Pariaconus grandis

XML Treatment for Pariaconus caulicalix

XML Treatment for Pariaconus crassiorcalix

XML Treatment for Pariaconus lehua

XML Treatment for Pariaconus elegans

XML Treatment for Pariaconus gagneae

XML Treatment for Pariaconus haumea

XML Treatment for Pariaconus oahuensis

XML Treatment for Pariaconus ohiacola

XML Treatment for Pariaconus lanaiensis

XML Treatment for Pariaconus pullatus

XML Treatment for Pariaconus molokaiensis

XML Treatment for Pariaconus hualani

XML Treatment for Pariaconus mauiensis

XML Treatment for Pariaconus kupua

XML Treatment for Pariaconus montgomeri

XML Treatment for Pariaconus hawaiiensis

XML Treatment for Pariaconus pele

XML Treatment for Pariaconus pyramidalis

## Supplementary materials

Supplementary material 1 Figure S1 Data type: PNG image file Explanation note: Illustration of adult characters and measurements referred to in the text. **A** fore wing length and width (dotted lines), veins R, M and Cu_1_, vein Rs length, cells cu_1_ and m_2_ width (w) and height (h) **B** head width (dotted line), vertex width (solid line), vertex length and genal process length **C** proboscis, distal segment length **D** hind leg femur length, tibia length, proximal and distal tarsus length **E** antennal length (dotted line), 3^rd^ antennal segment length (solid line) **F** male terminalia, proctiger length, subgenital plate height, paramere length, distal aedeagus segment length and apical head length **G** egg length and width **H–I** female terminalia: **H** lateral view, proctiger length, subgenital plate length, anal ring length **I** dorsal view, proctiger length, anal ring length. This dataset is made available under the Open Database License (http://opendatacommons.org/licenses/odbl/1.0/). The Open Database License (ODbL) is a license agreement intended to allow users to freely share, modify, and use this Dataset while maintaining this same freedom for others, provided that the original source and author(s) are credited.Diana M. Percy
