# Supplementary figures and images for: Making the most of your host: the Metrosideros-feeding psyllids (Hemiptera, Psylloidea) of the Hawaiian Islands
Source: Zookeys. 2017 Jan 31;(649):1–163. doi: 10.3897/zookeys.649.10213 (PMC5345378; doi:10.3897/zookeys.649.10213)

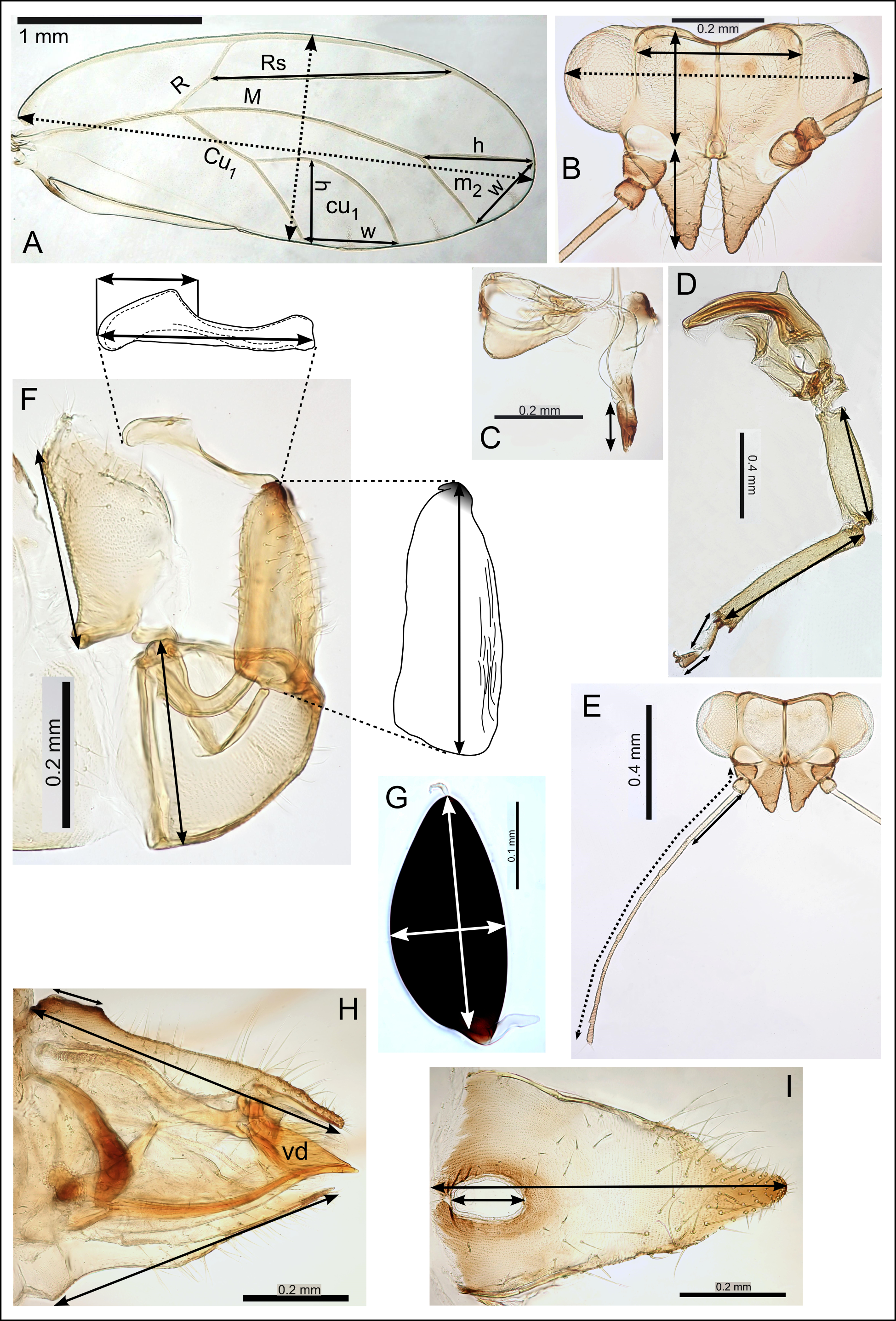

Supplement: Supplementary material 1 — Figure S1 [file zookeys-649-001-s001.png]
